# Mutant Proteomics of Lung Adenocarcinomas Harboring Different *EGFR* Mutations

**DOI:** 10.3389/fonc.2020.01494

**Published:** 2020-08-25

**Authors:** Toshihide Nishimura, Ákos Végvári, Haruhiko Nakamura, Harubumi Kato, Hisashi Saji

**Affiliations:** ^1^Department of Translational Medicine Informatics, St. Marianna University School of Medicine, Kawasaki, Japan; ^2^Department of Chest Surgery, St. Marianna University School of Medicine, Kawasaki, Japan; ^3^Division of Chemistry I, Department of Medical Biochemistry and Biophysics, Karolinska Institutet, Stockholm, Sweden; ^4^Division of Thoracic and Thyroid Surgery, Tokyo Medical University, Tokyo, Japan; ^5^Research Institute of Health and Welfare Sciences, Graduate School, International University of Health and Welfare, Tokyo, Japan

**Keywords:** mutant proteomics, lung adenocarcinoma, EGFR mutation, WGCNA, causal network analysis, disease-related molecular networks, mass spectrometry

## Abstract

Epidermal growth factor receptor *EGFR* major driver mutations may affect downstream molecular networks and pathways, which would influence treatment outcomes of non-small cell lung cancer (NSCLC). This study aimed to unveil profiles of mutant proteins expressed in lung adenocarcinomas of 36 patients harboring representative driver *EGFR* mutations (Ex19del, nine; L858R, nine; no Ex19del/L858R, 18). Surprisingly, the orthogonal partial least squares discriminant analysis performed for identified mutant proteins demonstrated the profound differences in distance among the different *EGFR* mutation groups, suggesting that cancer cells harboring L858R or Ex19del emerge from cellular origins different from L858R/Ex19del-negative cells. Weighted gene coexpression network analysis, together with over-representative analysis, identified 18 coexpressed modules and their eigen proteins. Pathways enriched differentially for both the L858R and Ex19del mutations included carboxylic acid metabolic process, cell cycle, developmental biology, cellular responses to stress, mitotic prophase, cell proliferation, growth, epithelial to mesenchymal transition (EMT), and immune system. The IPA causal network analysis identified the highly activated networks of *PARPBP, HOXA1*, and *APH1* under the L858R mutation, whereas those of *ASGR1, APEX1, BUB1*, and *MAPK10* were highly activated under the Ex19del mutation. Interestingly, the downregulated causal network of osimertinib intervention showed the highest significance in overlap *p*-value among most causal networks predicted under the L858R mutation. We also identified the causal network of MAPK interacting serine/threonine kinase 1/2 (*MNK1/2*) highly activated differentially under the L858R mutation. Tumor-suppressor *AMOT*, a component of the Hippo pathways, was highly inhibited commonly under both L858R and Ex19del mutations. Our results could identify disease-related protein molecular networks from the landscape of single amino acid variants. Our findings may help identify potential therapeutic targets and develop therapeutic strategies to improve patient outcomes.

## Highlights

- The first study to perform mutant proteomic analysis of clinical tissue specimens obtained from patients of lung adenocarcinoma with *EGFR* oncogenic driver mutations.
- Surprisingly, the OPLS discriminant analysis revealed profound differences among the profiles of mutant proteins identified under the different *EGFR* mutation statuses, which were never seen before.
- Weighted gene coexpression network analysis (WGCNA) screened by the over-representative test identified 18 significant network modules under the respective *EGFR* mutation statuses.
- Interestingly, the downregulated causal network of osimertinib intervention and highly activated *MNK1/2* were associated with L858R-positive lung adenocarcinoma. Upstream regulators and causal networks predicted suggested a close link to *EGFR* mutation-positive cancers, mainly NSCLC.

## Introduction

The discovery of somatic mutations in the tyrosine kinase domain of the epidermal growth factor receptor (EGFR) (1, 2) drastically changed the therapeutic perspective of non-small-cell lung cancer (NSCLC). The representative *EGFR* oncogenic mutations are in-frame deletions in exon 19 (Ex19del) (44.8%) and a point mutation at Leu-858 substituted with arginine (L858R) (39.8%) (3). Personalized and/or precision medicine (PM) have been successful by targeting those mutations with tyrosine kinase inhibitors (TKIs) gefitinib, erlotinib, and afatinib. Because most patients, however, suffer from drug resistance after a year of treatment, therapeutic strategies have been challenged to improve the survival benefit of first-line treatment. The efficacy of the first- and second-generation EGFR-TKIs is limited by the result of drug resistance conferred by another mutation involving the substitution of threonine 790 with methionine (T790M) (4).

Osimertinib is an irreversible third-generation EGFR-TKI that is selective for sensitizing EGFR and T790M mutations. The randomized phase III AURA3 trial demonstrated that the efficacy of osimertinib was significantly greater than that of platinum therapy plus pemetrexed in patients with T790M-positive advanced NSCLC (5). Recently, osimertinib was recommended as first-line treatment for patients with *EGFR*-mutant NSCLC according to the FLAURA trial that reported significantly better PFS and OS with osimertinib than with first-generation EGFR-TKIs (gefitinib or erlotinib) (6, 7).

Numerous studies have been reported regarding *EGFR* mutations and their disease-related downstream signaling pathways (8–12) and EGFR-TKIs resistance (13–15). Hyperactivation of STAT3 enhances carcinogenesis in various cancers (16, 17) and drives drug resistance in response to EGFR TKIs (18). Chromosomal instability was found to be significantly increased during TKI treatment in T790M-negative patients and resulting co-acquired alterations and genomic evolution are primarily responsible for resistance to the first-generation TKIs (19). Low-frequency *EGFR* mutations in NSCLC, including point mutations, deletions, insertions, and duplications within exons 18–25, are also associated with poor responses to EGFR TKIs (20).

Today, the field of proteomics is strongly dominated by mass spectrometry (MS)–based methodologies, largely due to that modern mass spectrometers offer high mass resolution and accuracy required for correct protein identification. The most successful approach is “shotgun” proteomics that employs proteases (often trypsin) to enzymatically cleave proteins resulting in peptides, which are more convenient to separate and sequence (21). The most reliable protein identification strategy in shotgun proteomics is based on tandem (MS/MS) mass spectra generation of tryptic peptides by fragmentation and their consecutive search against databases of canonical/consensus sequences. MS-based proteomics has been extensively applied to investigate EGFR regulations, including phosphorylation, ubiquitination, and protein–protein interactions as well as post-translational modifications (22, 23). Zhang et al. performed quantitative phosphoproteomics to unveil global phosphorylation changes upon the erlotinib treatment of *EGFR* mutation-positive lung adenocarcinoma cells (24, 25).

Unfortunately, mutant proteins, those products of non-synonymous single nucleotide polymorphisms (nsSNP), are overlooked in general MS-based proteomic data because these proteoforms are excluded in canonical protein databases (26). Although, the high number of nsSNPs, estimated to be >3 million, suggests that single amino acid variants (SAAVs) are widely distributed in the human proteome (27), only a couple of mutant proteins have been detected at expression level in human samples (28). Cancerous diseases are often characterized by high mutation rates (29) that are tightly associated with the physiological and pathological traits of individuals (30), whereas the allele-specific gene expressions in the heterozygous state are also associated with various traits of individuals (31, 32).

Because many of these mutant proteins are exclusively expressed in cancer cells (33), they can be identified as lead candidates of optimal disease biomarkers. The qualitative and quantitative analyses of these proteoforms, thus, can provide novel diagnostic and prognostic values.

A laser microdissection (LMD) technique enables the collection of target cells of a certain type from sections of formalin-fixed paraffin-embedded (FFPE) cancer tissue (34, 35). Label-free spectral counting and identification-based semiquantitative shotgun proteomic analysis of microdissected target cancerous cells of a certain type were used that characterized lung adenocarcinoma (35).

A pivotal challenge is to understand how the major driver mutations—*EGFR* L858R and Ex19del—affect disease-related downstream networks together with other upstream driver mutation crosstalk, which plays a central role in the context of lung cancer progression, malignancy, and outcome and/or resistance regarding TKI therapies (28). We performed mutant proteomic analysis and applied the weighted gene correlation network analysis (WGCNA), which is an unsupervised gene-clustering method based on the correlation network of gene expression (36–38) as well as spectral counting-based comparative analysis. The main aim of this study was to identify the key modules and networks of mutant proteins associated with the *EGFR* mutations L858R and Ex19del. To our knowledge, this is the first proteomics study performed to identify mutant proteoforms expressed in clinical tissue specimens.

## Materials and Methods

### FFPE Tissue Specimens and Sample Preparation

Among 974 patients who underwent surgical lung cancer resection at St. Marianna University Hospital between 2000 and 2014, only 674 (69.3%) had tumors that were histologically confirmed adenocarcinomas. Pathological specimens were reviewed by pathologists to confirm that they satisfied the 2015 WHO classification of lung tumors (histological criteria) (39). For tissue microdissection, 10-μm-thick sections from the FFPE tumor blocks were cut onto DIRECTOR slides (OncoPlex Diagnostics Inc., Rockville, MD, USA). The sections were deparaffinized and stained only with hematoxylin using standard histological methods prior to dissection. Microdissection was performed using a Leica LMD7 Microdissection Microscope (Leica, Wetzlar, Germany). A total area of 4 mm^2^ with about 15,000 tumor cells was transferred from the FFPE sections via laser dissection directly into the cap of a 200-μL low-binding tube. Proteins were extracted and digested with trypsin using Liquid Tissue MS Protein Prep kits (OncoPlex Diagnostics, Inc.). The procedures have been described in detail elsewhere (34, 35, 38).

### Liquid Chromatography-Tandem Mass Spectrometry

Digested protein samples were used for liquid chromatography-tandem mass spectrometry (LC-MS/MS) analysis on a Q-Exactive Orbitrap mass spectrometer (Thermo-Fisher Scientific, Bremen, Germany) equipped with an LC system operated at 500 nL/min via a nano-ESI device (AMR Inc., Tokyo, Japan). The gradient was 110 min long and a 5-μL sample was injected in each analysis.

All LC-MS/MS data were acquired using Xcalibur, version 2.8 SP1 (Thermo Fisher Scientific) in high-resolution data-driven analysis (DDA) mode with the survey scan (MS in the mass range *m/z* 400–1,600) acquired in the Orbitrap at 70,000 resolution (at *m/z* 200) in profile mode. The survey scan was followed by the top 10 higher energy collision-induced dissociation (HCD) MS/MS spectra, acquired in centroid mode in the Orbitrap at 17,500 resolution.

For MS/MS acquisition of top 10 precursors, the following settings were used: minimal signal threshold = 1,700; isolation width = 1.6 *m/z*; normalized collision energy = 27%. Monoisotopic precursor selection, charge-state screening, and charge-state rejection were enabled with rejection of singly charged and unassigned charge states. Dynamic exclusion was enabled to remove selected precursor ions (±10 ppm) for 15 s after MS/MS acquisition. The expression levels of identified mutant proteins were assessed by spectral count-based protein quantification. Fold changes of expressed proteins in the base 2 logarithmic scale (*R*_*SC*_) (40) were calculated using the spectral count (*SpC*) that is the number of MS/MS spectra assigned to each mutant protein.

### Identification of Mutant Proteoforms

**Figure 1:**
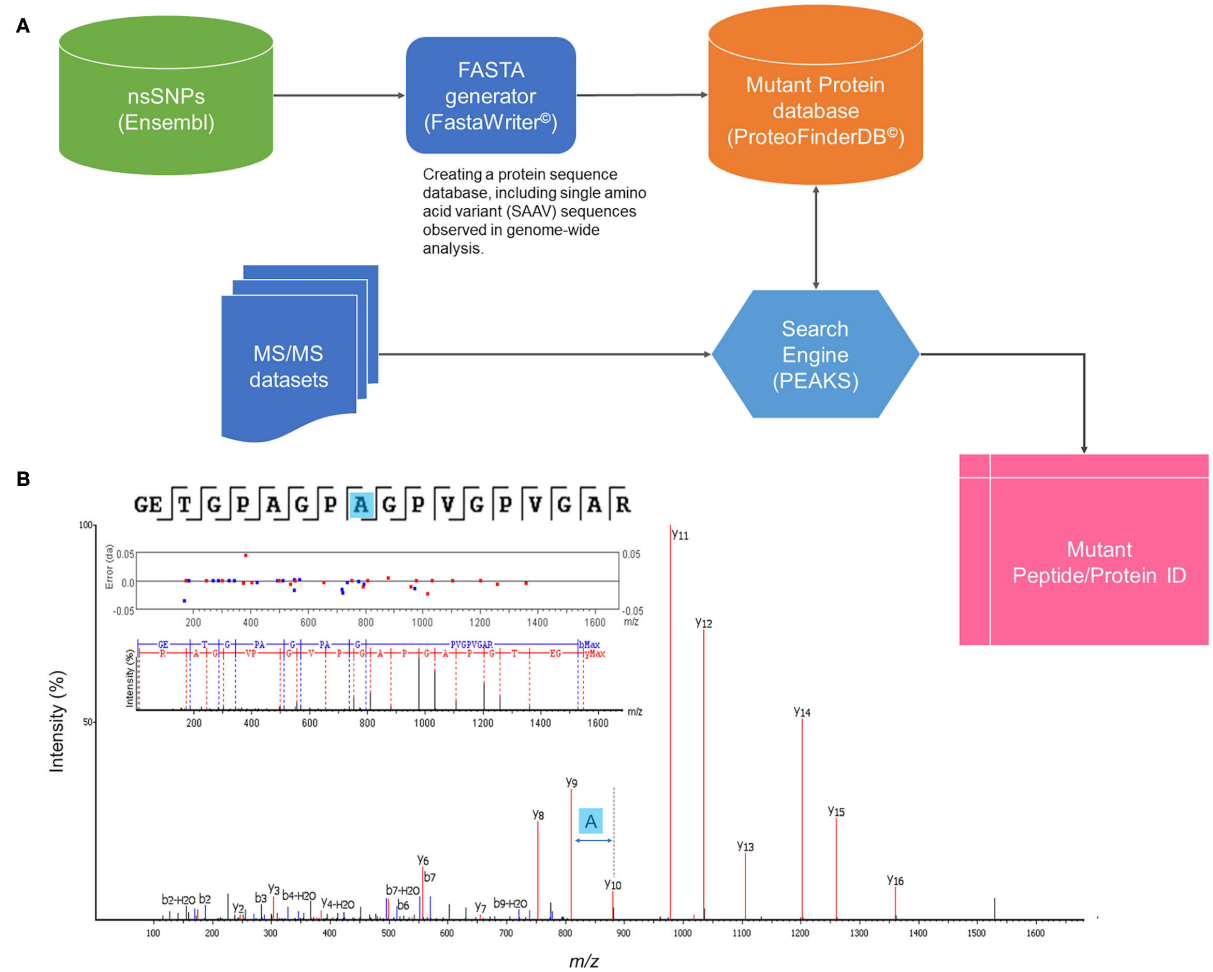
General workflow of mutant proteomics. **(A)** Identification of SAAVs by tandem mass (MS/MS) spectra searching a specialized protein database, ProteFinder (PFdb), which includes 1.9 million SAAVs, all human consensus sequences, splicing variants, and 105 common contaminant proteins. PFdb is verified in PEAKS (Biosystems Inc.) and used together with its decoy databases. **(B)** MS-based sequencing of a SAAV of collagen a-1(I) chain (COL1A1–P02452) at the point mutation, T1075A.

A strategy to identify mutant proteoforms in lung cancer samples was designed using high-quality shotgun proteomics tandem mass spectra. The central component of the approach was a unique set of protein sequences, which included SAAV sequences translated from known genomic studies. Using a custom-made software tool (FastaWriter v1.4.0), a new database of mutant protein sequences (ProteoFinder v17.04.12) was generated to include a mutation in each new entry that, thus, differed in amino acid from the consensus protein. Titin (Q8WZ42) was removed from the database to decrease the size of the database as 21,045 mutations were registered only on this protein. The resulting *in silico* derived proteoforms (total number of searchable mutations of 1,899,031) were denoted following the neXtProt nomenclature, including the access codes but adding information also about the position of the mutation (such as NX_P07288-S132L). These SAAV sequences were then shortened to reduce redundancy, keeping only the part of the protein sequence where the amino acid exchange took place surrounded by two additional tryptic peptides at both N- and C-termini. The new database entries were rendered as a combination of consensus (neXtProt database 2017-04-12 release) and mutant proteoform sequences in standardized *fasta* format. Figure 1 illustrates a general workflow of identification of SAAVs by tandem mass spectra searching a specialized protein database, ProteFinder (PFdb), and MS-based sequencing of a mutant peptide is exemplified.

The MS raw files of 108 runs (36 patient samples as triplicate) were imported into PEAKS Studio v8.5 (Bioinformatics Solutions Inc., Waterloo, Canada) (41) for database searching against the PF v17.04.12 database, appended with contaminant sequences (cRAP). PEAKS database searches were performed with a precursor ion error tolerance of 10 ppm, fragment ion error tolerance of 0.05 Da, fixed carbamidomethyl cysteine, and modifications of oxidation (M), deamidation (NQ), and acetylation (N-term) were set dynamically. Trypsin was specified as the enzyme, allowing for two missed cleavages. The technical triplicates were searched together, generating a single combined result file of each biological sample.

The search results were further filtered for hits with mutant specific tryptic peptides removing all multiple protein identifications while multi-isoform hits with the same amino acid change were included in the final list. Non-tryptic peptides with the mutation were not considered as reliable identification and were excluded in the additional filtering steps. Isobaric amino acid mutations, i.e., exchange of Ile to Leu and Leu to Ile, were registered for future experimental verification and kept as potentially valid identifications. The summary of each search, including score distributions and statistical data, which are available as PDF files (e.g., AZ0x_summary.pdf) in Search summaries in Supplementary Information File 1.

The technical triplicates together have resulted in rich data with an MS/MS spectra range of 44,798–143,118, providing a peptide sequence match (PSM) range of 10,464–51,133, peptide sequence range of 7,703–19,974, and protein group range of 1,221–2,266. The identified mutant protein sequences were between 252 and 964 after filtration, which is presented in Supplementary Information File 2. The protein sequences carrying amino acid variants were registered, and the presence of each mutant protein was indicated in the technical replicates as well as their scan numbers.

### WGCNA

The similarity among protein expression patterns for all protein pairs was calculated according to their pairwise Pearson's correlation coefficient, i.e., the similarity between proteins *i* and *j* was defined as (1 – *r*_*i, j*_)/2, where *r*_*i, j*_ is the correlation of the protein expression patterns between the two proteins *i* and *j*. An adjacency matrix was then computed by increasing the similarity matrix up to the power of 10 to generate a coexpression network with scale-free properties. Subsequently, from the resultant scale-free coexpression network, we generated a topological overlap matrix (TOM) that considers topological similarities between a pair of proteins in the network. Using the dissimilarity according to TOM (1 – TOM), we conducted hierarchical clustering to generate a tree that clustered proteins in its branches. Dynamic tree cutting was used to trim the branches to identify protein modules. A protein module was summarized by the top hub protein (referred to as eigen-protein) with the highest connectivity in the module. To identify the protein modules associated with clinical traits, we calculated the correlation coefficients between the eigen-proteins and clinical traits. WGCNA was conducted using a Garuda Platform gadget (The Systems Biology Institute, Tokyo, Japan) that implemented the WGCNA pipeline based on the WGCNA R-package (36).

### Protein–Protein Interaction Network Construction and Functional Enrichment

To construct a protein interaction network for a protein module, we used the STRING database (version 11.0) (42), which accumulates information on protein–protein interactions from various other databases, such as IntAct, Reactome, DIP, BioGRID, MINT, KEGG, NCI/Nature PID, the Interactive Fly, and BioCyc. STRING networks were constructed under the criteria for linkage only with experiments, databases, text mining, and coexpression using the default settings, i.e., a medium confidence score of 0.400, a network depth of 0 or 50 interactions. Subsequently, protein networks imported from the STRING database were visualized using *Cytoscape* version 3.7.2. Functional enrichment results were obtained for canonical pathways considering *p* < 0.05 to be statistically significant.

### Comparative Analysis of the Causal Networks and Pathways Predicted by IPA

Canonical pathways, upstream regulators, and causal networks were predicted using the ingenuity pathway analysis (IPA) software (43). Mutant protein expression data (quantile-normalized for selected modules) were used as input data sets. Comparative analysis of the predicted causal networks (*p*-value < 0.05) was performed to elucidate networks associated with the three clinical traits: Ex19del, L858R, and no Ex19del/L858R mutations, where activation and inhibition of a predicted network were defined by *z*-values >2.0 and < −2.0, respectively, and upregulation and downregulation were defined by *z*-values >1.0 and < −1.0, respectively.

## Results

### Mutant Proteome Data Sets of Lung Adenocarcinoma

MS-based proteomic analysis was conducted for 36 FFPE tissue specimens of lung adenocarcinoma (35 involved the acinar subtype and one involved the papillary subtype). These specimens were selected for their preserved condition, tumor area, and well-clarified pathological diagnosis and *EGFR* mutation status (nine specimens of the clinical trait M1: L858R mutation, nine specimens of the clinical trait M2: Ex19del mutation, and 18 specimens of the clinical trait NM: no Ex19del or L858R mutation; see Table 1). Pre-surgical treatment was not performed in any of the cases.

**Table 1:** Clinicopathological information of the 36 patients.

**Variable**	**Category**	**No. patients**	**%**
**Gender**			
	Female	16	44.4
	Male	20	55.6
**AGE (years)**			
	Median (range)	67.9 (22–82)	
**Smoking index (Brinkmann Index, BI)**			
	**Female**		
	BI = 0	12	75
	0 < BI ≤ 400	0	0
	400 < BI ≤ 600	0	0
	600 < BI ≤ 1,200	4	25
	BI > 1,200	0	0
	**Male**		
	BI = 0	3	15
	0 < BI ≤ 400	4	20
	400 < BI ≤ 600	0	0
	600 < BI ≤ 1,200	10	50
	BI > 1,200	3	15
**Histologic type**			
	Adenocarcinoma	36	100
**Subtype**			
	Acinar	35	97.2
	Papillary	1	2.8
**Surgical method**			
	Radical lobectomy	24	66.7
	Limited resection	12	33.3
**Tumor size on CT**			
	T1a (≤1 cm)	1	2.8
	T1b (1–2 cm)	11	30.6
	T1c (2–3 cm)	11	30.6
	T2a (3–4 cm)	4	11.1
	T2b (4–5 cm)	7	19.4
	T3 (5–7 cm)	2	5.6
	T4 (>7 cm)	0	0
**Clinical stage**			
	cIA	21	58.3
	cIIA	2	5.6
	cIB	9	25
	cIIB	1	2.8
	cIV	3	8.3
***EGFR*** **mutation status**			
	**Positive**		
	L858R	9	25
	Ex19 E746-A750 del	9	25
	**Negative**		
	Neither L858R nor Ex19del	18	50

**Figure 2:**
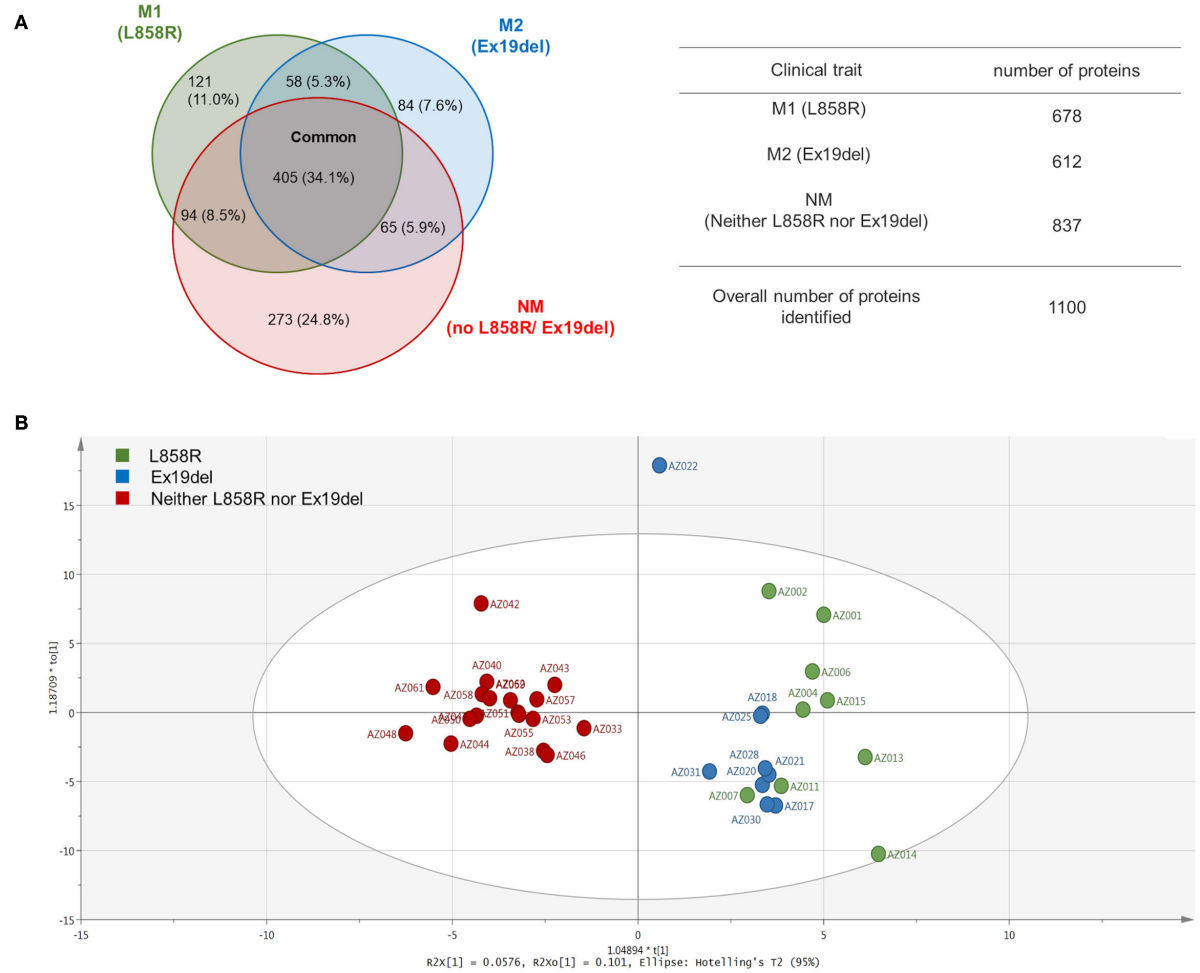
Venn map and orthogonal partial least square-discriminant analysis (OPLS-DA) of the identified proteins. **(A)** Venn map of the identified proteins. **(B)** OPLS-DA of the expressed proteins including their spectral counts for patients.

A total of 1,100 mutant proteins were identified, in which M1, M2, and NM were 678, 612, and 837, respectively, and 405 (34.1%) were expressed commonly (Figure 2A). The proportion of mutant proteins unique to the L858R mutation was 121 (11.0%), and that to the Ex19del mutation was 84 (7.8%), whereas the proportion of proteins expressed in only no *EGFR* mutation cases was 273 (24.8%). GO analysis using PANTHER Ver. 14.1 (44) exhibited mostly similar profiles in gene hits for all the traits (M1, L858R mutation; M2, Ex19Del mutation; NM, no Ex19del or L858R mutation; see Figure S1). Mutation proteins with high hits in GO biological process included cellular process (GO:0009987), localization (GO:0051179), cellular component organization or biogenesis (GO:0071840), biological regulation (GO:0065007), metabolic process (GO:0008152), and response to stimulus (GO:0050896). Those in the GO protein class included cytoskeletal protein (PC00085), nucleic acid-binding protein (PC00171), and metabolite interconversion enzyme (PC00262).

An orthogonal partial least square-discriminant analysis (OPLS-DA) (45) was applied to identified mutant proteins and interestingly exhibited profound differences in distance among the *EGFR* mutation statuses (Figure 2B), whereas a conventional hierarchical clustering of patients according to mutant protein abundance failed to show a clear separation among the three clinical traits. Surprisingly, clear differentiation was found between the NM group and the M1 and M2 groups. The data points of the M1 group appeared to be to some extent scattered, whereas those of the M2 group clustered closely. These findings seem to unveil the mutant proteome landscape correlating with the *EGFR* mutation type in lung adenocarcinoma.

### Identification of Key Mutant Protein Modules by WGCNA

**Figure 3:**
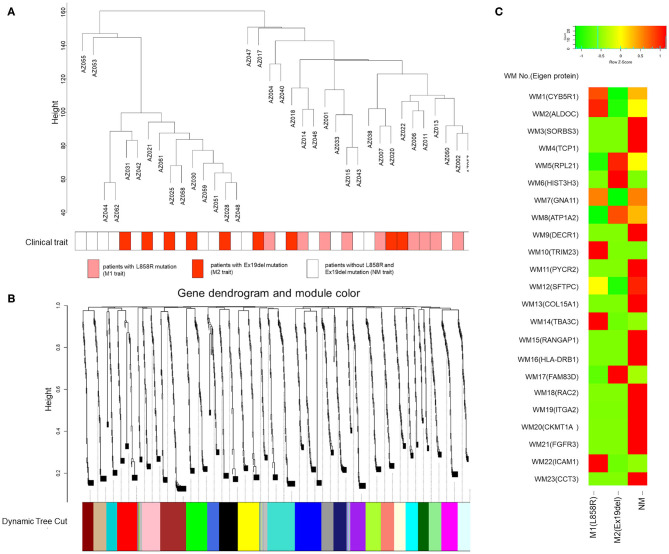
Gene modules identified by weighted gene coexpression network analysis (WGCNA). **(A)** Patient clustering according to mutant protein abundance with the *EGFR* mutation profiles. The red, orange, and white cells below the patients indicate the *EGFR* mutation types, i.e., Ex19del mutation, L858R mutation, and no *EGFR* mutation, respectively. **(B)** Gene dendrogram obtained by clustering dissimilarity according to topological overlap with the corresponding module. The colored rows correspond with the 23 modules identified by dissimilarity according to topological overlap. **(C)** Heat map for the proteome abundance of eigen proteins in the 23 mutant protein modules by WGCNA. The rows and columns are the mutant protein modules and *EGFR* mutation types, respectively. The red and green colors indicate high and low mutant protein abundances, respectively, of an eigen protein in a mutant protein module. The names of the eigen proteins in the protein modules are indicated in parentheses.

A weighted gene coexpression network was constructed in which all the identified mutant proteins were clustered, and we found 23 mutant protein modules (Figures 3A,B). A spectral counting-based heat map (46) for eigen-proteins in the modules is shown in Figure 3C. In the WGCNA, a soft threshold power of 15 was selected to define the adjacency matrix according to the criteria of approximate scale-free topology with a minimum module size of 30 and a module detection sensitivity *deepSplit* of 4. The clinical traits for patients were set according to the *EGFR* mutation status with M1, M2, and NM traits corresponding to L858R mutation, Ex19del mutation, and neither Ex19del/L858R mutation, respectively. The correlations between resultant modules and clinical traits were determined to identify mutant protein modules whose expressions were up- or downregulated in L858del, Ex19del, or no Ex19del/L858R mutation samples (Figure S2).

Among the 23 modules, only the WM6 module was moderately correlated with the *EGFR* Ex19del mutation status (*r* = 0.41, *p* < 0.05). Most of the other WGCNA modules were not statistically significant. However, several modules seem to be characteristic to the clinical traits (Figure S2). The WM10, WM12, WM14, and WM22 modules seem to be characteristic to the L858R mutation status (*r* = 0.3, *p* = 0.08). The WM17 module showed a positive correlation with the Ex19del mutation status (*r* = 0.3, *p* = 0.08). We could find no modules characteristic to the NM trait (no L858R or Ex19del mutations).

### WGCNA Modules Screened by ORA and Functional Enrichment Analysis

The computational WGCNA framework (36) has been proven to be powerful in identifying coexpression protein modules (37, 38). However, it should be noted that traditional trait analysis of the correlations between eigen components of WGCNA modules and clinical traits might overlook important modules for investigating molecular mechanisms differentially behind a disease. Especially for clinical traits quite close to each other, difficulties would be sometimes encountered to attain identification of key WGCNA modules with a high significance. Multiple correction testing, such as Bonferroni, Benjamini-Hochberg, etc., would result in that none of the modules associated with M1 or M2 remains significant. Statistical over-representative analysis (ORA) would help to evaluate potential key WGCNA modules with identified proteins uniquely expressed and upregulated to each trait.

**Figure 4:**
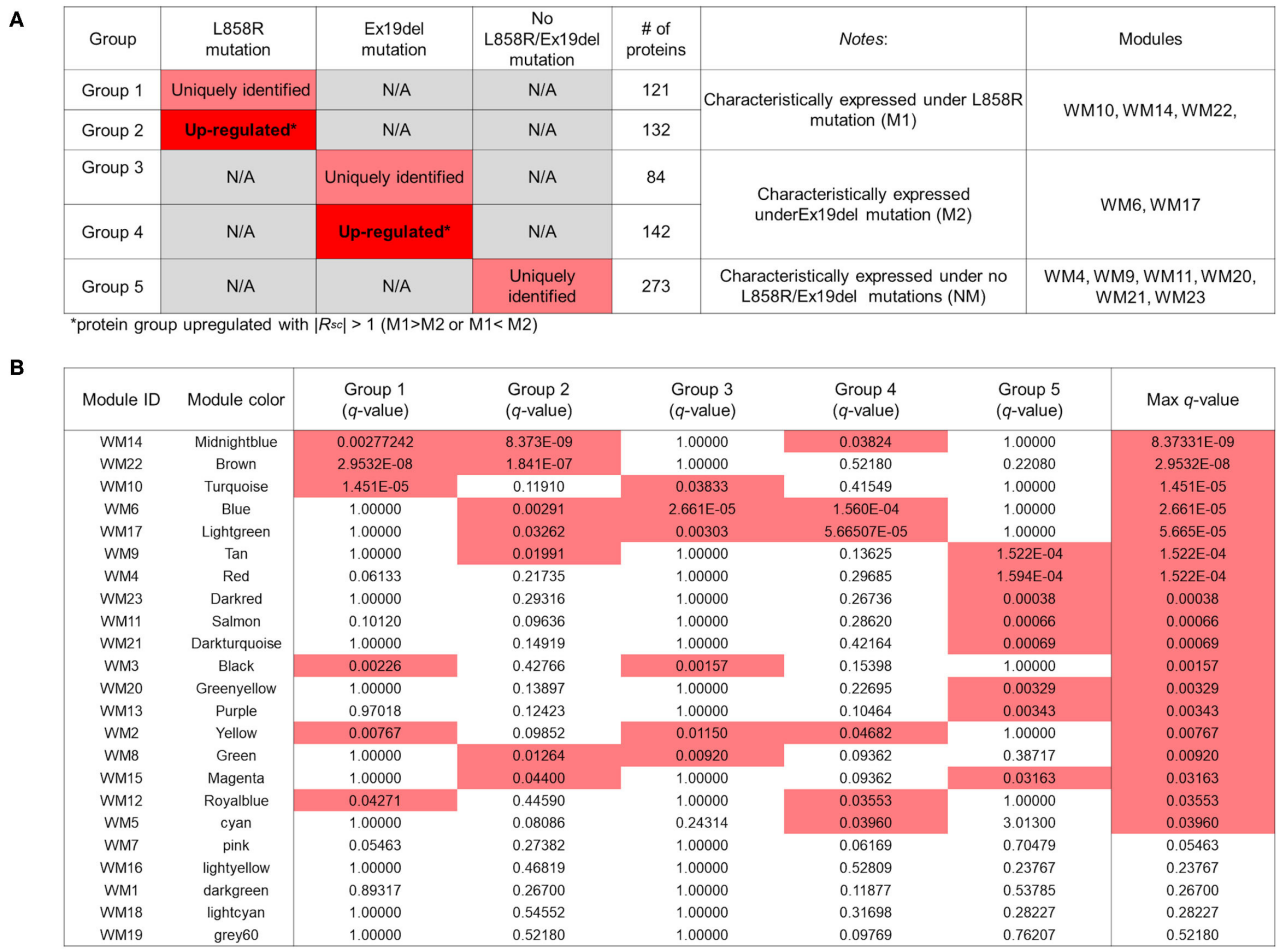
Overlapping proteins unique to the clinical traits and/or upregulated under the M1 or M2 traits and those from weighted gene coexpression network analysis (WGCNA). **(A)** Results of identified proteins and spectral-counting based semiquantitative comparison. Each row represents results for each protein group. The red and pink cells in the “L858R mutation” and “Ex19del mutation” columns indicate that the proteins in the group are uniquely expressed and significantly upregulated, respectively, in samples with the mutations [Upregulated with *|Rsc|* > 1 (M1 > M2 or M1 < M2)]. The fourth column shows the number of proteins in each protein group. The fifth column provides notes for each protein group. The WGCNA modules with significant overlap with each protein group are listed in the sixth column (“Modules” column). **(B)** Overlap in proteins between the groups by the protein expression profiles and the modules by WGCNA. Each row in the embedded table represents overlap analysis results for each module. The first and second columns in the table represent module ID and color name of the module. The third through eighth columns indicate the *q*-values for overlap in proteins between a module by WGCNA and the five protein groups. In the six columns, significant *q*-values are highlighted in red. The eighth column represents the value of the most significant *q*-value (max *q*-value) in each module. The 18 modules with max *q*-values <0.05 are listed in order.

We conducted an ORA-based screening of WGCNA mutant protein modules to further identify key protein modules to investigate the differential disease mechanisms associated with the *EGFR* L858R and Ex19del mutation statuses; 121, 84, and 273 mutant proteins identified were expressed uniquely to the respective traits: M1, M2, and NM (Figure 3A); 132 and 142 mutant proteins were upregulated differentially to M1 and M2 with |*R*_*SC*_| >1 (higher than 2-fold change) in the comparison between M1 and M2 (Figure S3). The overlaps between the WGCNA-derived protein modules and identification-based significantly expressed proteins were then assessed using the over-representation test. We confirmed that five WGCNA modules overlapped significantly (maximum *q*-value among the groups <0.05) with protein groups unique to each trait and/or highly upregulated to M1 or M2 (Figures 4A,B).

**Figure 5:**
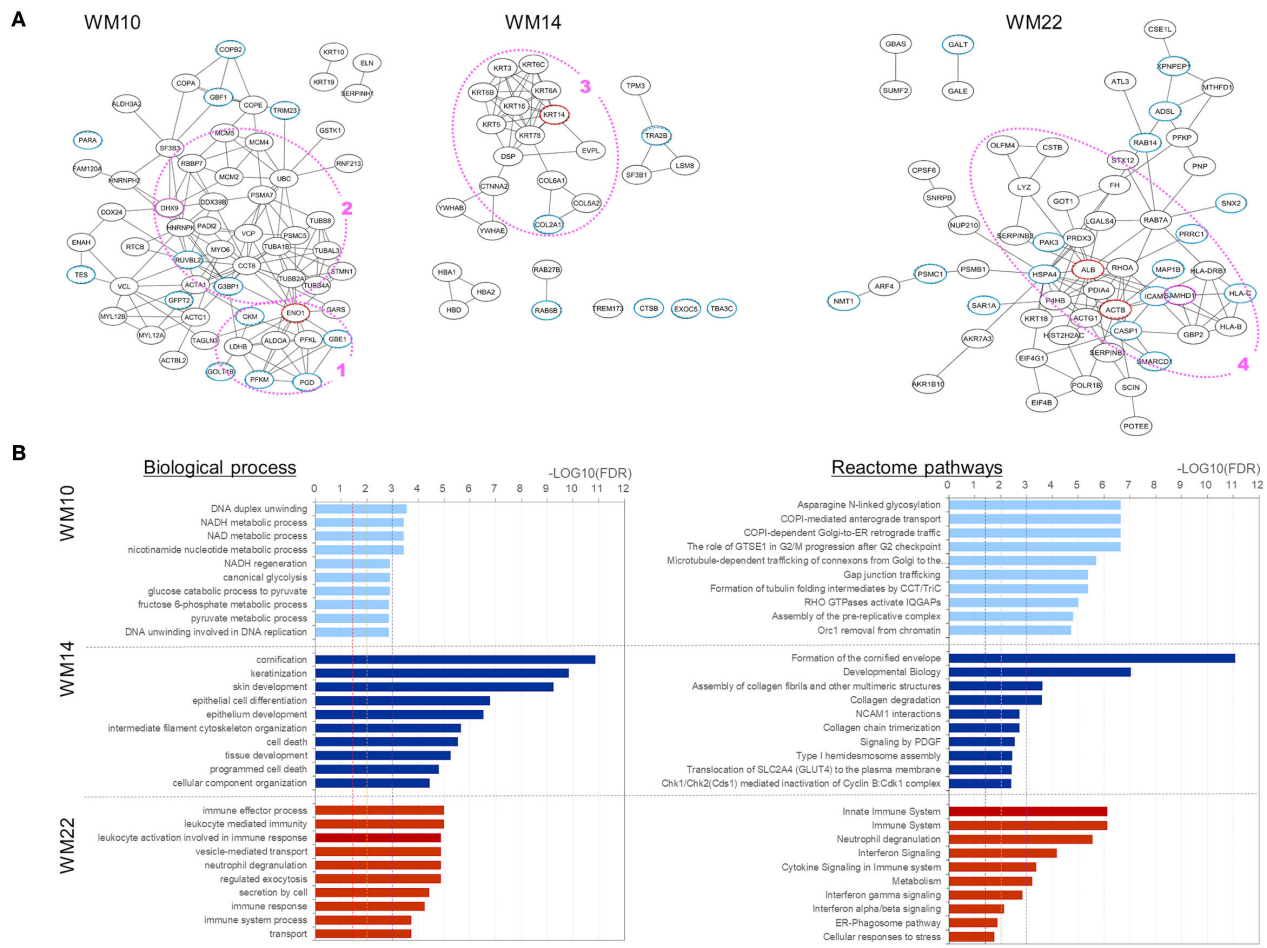
Analysis results for three protein modules (WM10, WM14, and WM22) that overlap with proteins uniquely and upregulated under the L858R mutation, respectively. **(A)** Protein interaction networks for the three WGCNA modules. Dotted circle nodes in blue and red represent eigen-proteins and hub proteins, respectively, for each module. **(B)** Pathway enrichment analysis using Go Biological Process and Reactome pathway databases for the three protein modules. The vertical axis shows the pathway names, and the bars on the horizontal axis represent the –log10 (*p*-value) of the corresponding pathway. The different colors of the bars are following the corresponding modules. Dashed lines in red, orange, and magenta indicate *p*-values <0.05, <0.01, and <0.001, respectively.

**Figure 6:**
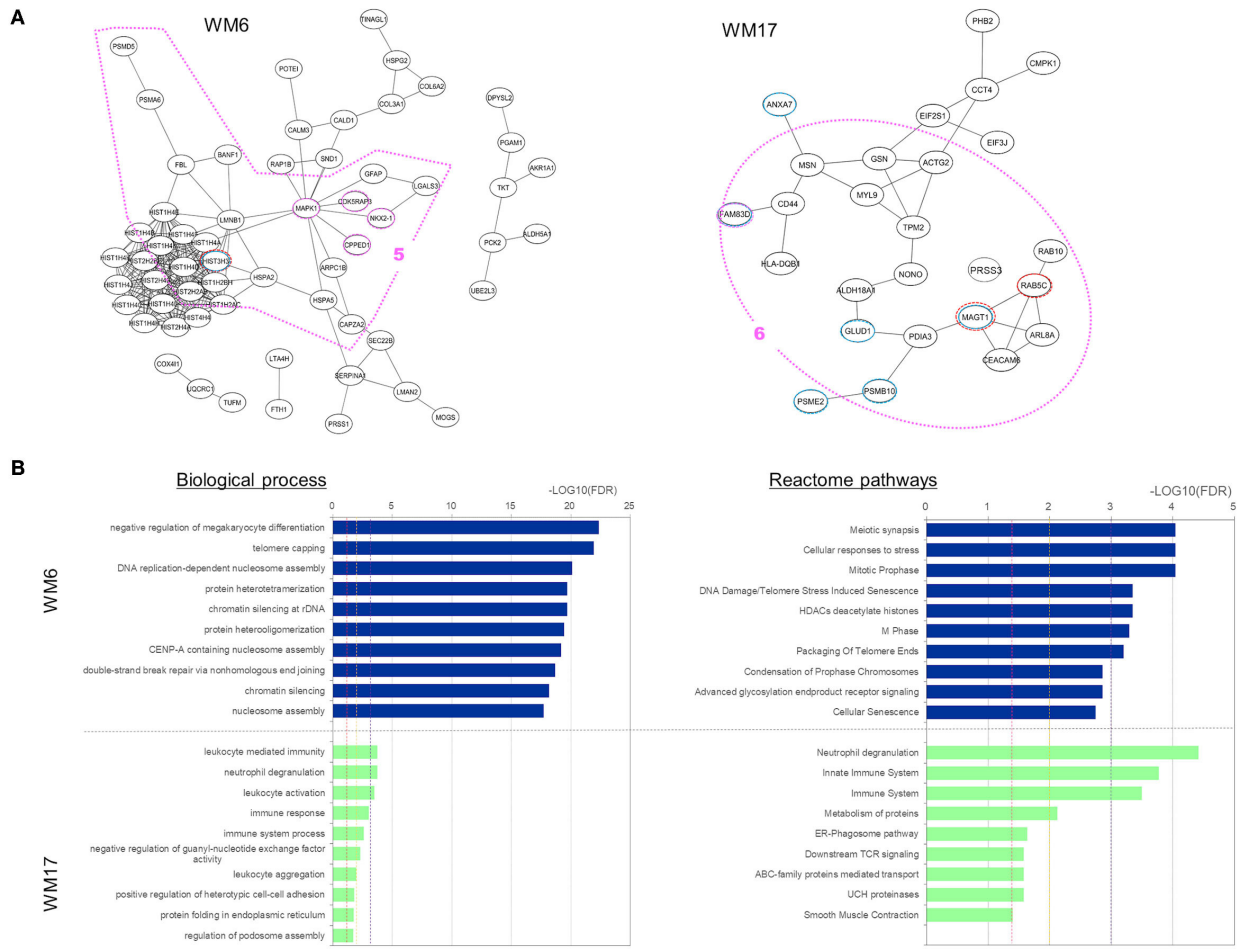
Analysis results for three protein modules (WM6 and WM17) that overlap with proteins uniquely expressed and upregulated under the Ex19del mutation. **(A)** Protein interaction networks for the three WGCNA modules. Dotted circle nodes in blue and red represent eigen-proteins and hub proteins, respectively, for each module. **(B)** Pathway enrichment analysis using Go Biological Process and Reactome pathway databases for the three protein modules. The vertical axis shows the pathway names, and the bars on the horizontal axis represent the –log10 (*p*-value) of the corresponding pathway. The different colors of the bars are following the corresponding modules. Dashed lines in red, orange, and magenta indicate *p* < 0.05, <0.01, and <0.001, respectively.

To characterize those five modules, we analyzed the biological connectivity among the proteins in each module by mapping the module proteins in the human protein–protein interaction (PPI) network and among the biological pathways by pathway enrichment analysis (Figures 5, 6).

Three WGCNA modules—WM10, WM14, and WM22—significantly overlapped with protein groups uniquely identified and highly upregulated under the L858R mutation (Figure 4). The enriched pathways of the WM10 mutant protein module involved DNA duplex unwinding, canonical glycolysis, glucose catabolic process to pyruvate, DNA unwinding involved in DNA replication, COPI-dependent Golgi-to-ER retrograde traffic, the role of GTSE1 in G2/M progression after G2 checkpoint, and formation of tubulin folding intermediates by CCT/TriC (Figure 5B). The hub protein alpha-enolase (also known as MBP-1) encoded by ENO1 is involved in the subnetworks related to the carboxylic acid metabolic process (as indicated by the pink dotted line 1 in Figure 5A) and is associated with glycolysis, growth control, and hypoxia tolerance. MBP-1 binds to the myc promoter and acts as a transcriptional repressor and so maybe a tumor suppressor. The cell cycle–related subnetwork is denoted by the pink dotted line 2 in Figure 5A. DHX9 encodes ATP-dependent RNA helicase A [also known as nuclear DNA helicase II (NDH II) or leukophysin (LKP)] participates in multiple processes of gene regulation, including transcription, translation, and DNA replication, and plays important roles at the maintenance of genomic stability. DHX9 has been reported to be overexpressed in various types of malignant tumors and might be a potential therapeutic target for the treatment of NSCLC (47).

The enriched pathways of the WM14 module include epithelial cell differentiation, tissue development, cell death, programmed cell death, developmental biology, and collagen degradation (Figure 5). The hub protein KRT14 is associated with developmental biology, which subnetwork is indicated by the pink dotted line 3 in Figure 5A. The enriched pathways of the WM22 module involve the immune effector process, immune response, cytokine signaling in immune system, and cellular responses to stress (Figure 5B). The subnetworks related mostly to the immune system (the pink dotted line 4 in Figure 5A), in which sterile alpha motif and HD domain-containing protein 1 (SAMHD1), a deoxyribonucleoside triphosphate triphosphohydrolase is known to play roles in defense response to the virus and cellular response to DNA damage stimulus, and is dysregulated in breast and other cancers (48). Frequently mutated SAMHD1 found in colon cancers was suggested to be involved in tumorigenesis with defective mismatch repair (MMR) (49) and also act as a resistance factor for anticancer drugs (50).

Two WGCNA modules—WM6 and WM17—significantly overlapped with protein groups uniquely identified and highly upregulated under the Ex19del mutation (Figure 4). The enriched pathways of the WM6 module involved DNA replication-dependent nucleosome assembly, chromatin silencing, double-strand break repair via non-homologous end joining, cellular responses to stress, DNA damage/telomere stress-induced senescence, and M phase (Figure 6B). The hub protein is the mutant H3.1t encoded by the mutant HIST3H3. Histone H3.1t protein (also known as H3t) itself is a core component of the nucleosome and plays a central role in transcription regulation, DNA repair, DNA replication, and chromosomal stability. The subnetworks related to both cellular responses to stress and mitotic prophase are indicated by the pink dotted line 5 in Figure 6A. Calcineurin-like phosphoesterase domain containing 1 (CPPED1, also known as CSTP1) blocks cell cycle progression and promoting cell apoptosis by dephosphorylating AKT family kinase (51). CDK5RAP3 itself encodes CDK5 regulatory subunit associated protein C53 (Cdk5rap3, also known as C53 and LZAP) that is a probable tumor suppressor involved in signaling pathways governing transcriptional regulation and cell cycle progression. Its specific mutant protein was reported to prevent apoptosis-induced cleavage of nuclear substrates, including nuclear shrinkage, chromatin condensation, and DNA fragmentation (52). The homeobox protein Nkx-2.1 [also known as thyroid transcription factor-1 (TTF-1)] has a role in lung development and surfactant homeostasis and is highly expressed in both small-cell lung carcinoma (SCLC) and lung adenocarcinoma (53, 54). Based on a quantitative real-time RT-PCR study of the NSCLC cell lines, Zu et al. (55) concluded that TTF-1 may serve as a tumor suppressor because of its inverse correlation with Ki-67 proliferative activity and increase of cellular apoptosis.

The enriched pathways of the WM17 module involved neutrophil degranulation, immune response, and immune system (Figure 6B). The hub protein is the RAS-related protein Rab-5C (also known as L1880 or RAB5L). Rab-5C itself is one of the three isoforms of Rab-5, which is a master regulator of the endocytic pathway. The subnetworks related mostly to the immune system process are indicated in the pink dotted line 6 in Figure 6A. Protein FAM83D (also known as spindle protein CHICA), a probable proto-oncogene, plays a role in cell proliferation, growth, migration, and epithelial to mesenchymal transition (EMT) (56). Elevated FAM83D expressions were reported in several cancers including metastatic lung adenocarcinomas (57). Recently, Shi et al. suggested its oncogenic activity by regulating cell cycle in lung adenocarcinoma (58).

### Comparative Analysis of Causal Networks Predicted by IPA

**Figure 7:**
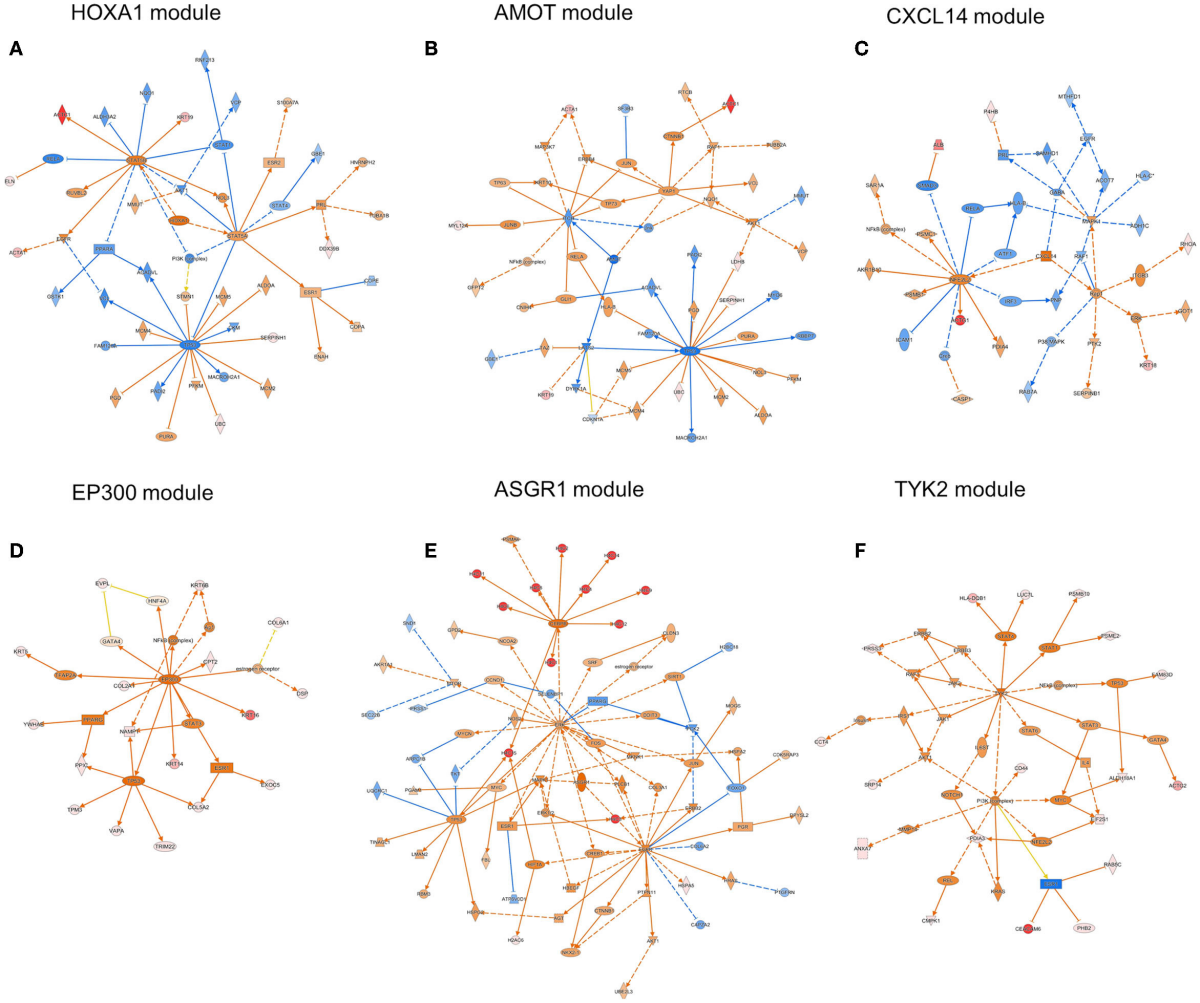
The representative modules of master and participating regulators with the target mutant proteins differentially significant under the *EGFR* L858 or Ex19del mutation status, which were obtained by using IPA software. The modules of **(A)** *HOXA1*, **(C)** *CXCL14*, and **(D)** *EP300* were predicted to be highly activated and **(B)** *AMOT* highly inhibited in association with the L858R mutation status. The modules of **(E)** *ASGR1* and **(F)** *TYK2* were highly and/or differentially activated on the Ex19del mutation status. Node shapes indicate molecular types: triangle, kinase; square (dashed), growth factor; rectangle (horizontal), ligand-dependent nuclear receptor; rectangle (vertical), ion channel; diamond (vertical), enzyme; diamond (horizontal), peptidase; trapezoid, transporter; oval (horizontal), transcription regulator; oval (vertical), transmembrane receptor; double circle, complex; circle, other. Red or light red colors indicate highly or moderately increased expression of a mutant protein in the data set. Orange or light orange colors indicate the extent of confidence for predicted activation and the blue and light blue for predicted inhibition. Lines denote predicted relationships. A solid or dashed line indicates direct or indirect interaction, respectively. Orange indicates leading to activation; blue, leading to inhibition; yellow, findings inconsistent with the state of a downstream molecule; gray, an effect not predicted.

The ORA-based screening of the WGCNA modules was performed to capture clinically important modules and their upstream regulators, which reflect the disease mechanisms affected differentially under the different driver *EGFR* mutations in lung adenocarcinoma. Both upstream regulators and causal networks using IPA (http://www.ingenuity.com) software (43) were performed especially for the two selected modules, WM 10 and WM6, which were significantly associated with the Ex19del and L858R mutation (Figures 5, 6). Causal networks predicted for these mutant protein modules included chemical drugs, transcriptional regulators, transmembrane receptors, growth factors, kinases, transporters, etc. Table 2 summarizes top causal networks significant to each module (|z-value| > 1.5) representative under the *EGFR* L858R or Ex19del mutation status in the order of higher overlap significance, *p*-value. Figure 7 presents the representative modules of master and participating regulators with the target mutant proteins differentially significant to the *EGFR* L858 or Ex19del mutation status.

**Table 2:** The top master regulators of causal networks predicted using the ingenuity pathway analysis (IPA) for the WM10 and WM6 modules, which are representatively characteristic under the L858R and Ex19del mutation statuses, respectively.

**Module ID (module color)**	**Master regulator**	**Molecule type**	**Depth**	**Predicted activation state**	**Activation *z*-score**	***p*-value of overlap**	**Network bias-corrected *p*-value**	**Participating regulators^a^**	**Target molecules in dataset^b^**
WM 10 (turquoise)	Osimertinib	Chemical drug	3	Downregulated highly under M1 and NM	−1.667	3.84E-12	1.00E-04	*AGT, Akt, AKT1, BLK, Creb, CTNNB1, EGFR, ERBB2, ERBB3, ERBB4, ERK1/2, ESR1, FOS, FOXO1, HBEGF, HIF1A, HRAS, JUN, KRAS, MAP2K1, MTOR, NFkB (complex), NRG1, osimertinib, PSEN1, RAF1, RB1, ICTOR, SRC, STAT1, STAT3, STA T5B, STAT6, TNK2, TP53, TSC2*	*ACADVL, ACTA1, ACTC1, ALDH3A2, ALDOA, COPA, COPB2, COPE, DDX39B, ENAH, ENO1, FAM120A, G3BP1, GBE1, GFPT2, GSTK1, HNRNPK, KRT10, LDHB, MACROH2A1, MCM2, MCM4, MCM5, MMUT, NIPSNAP1, PADI2, PDXDC1, PFKL, PFKM, PGD, PRPH, PSMC5, PURA, RBBP7, RNF213, RTCB, RUVBL2, SERPINH1, SF3B3, STMN1, TES, TMEM109, TUBB2A, TUBB4A, VCL, VCP*
	*NEUROG1*	Transcription regulator	3	Downregulated highly under M1 and NM	−1.89	4.35E-11	1.00E-04	*Akt, AKT1, Cdc42, CHUK, ERK1/2, HRAS, MAP2K1/2, MRTFA, NEUROG1, PI3K(complex), RAF1, RELA, RHOA, ROCK2, SRF, STAT, STAT3, STAT4, STAT5A, STAT5B, STAT6, TP53*	*ACADVL, ACTA1, ACTC1, ALDH3A2, ALDOA, COPB2, ELN, ENAH, ENO1, FAM120A, GBE1, GFPT2, GSTK1, KRT10, LDHB, MACROH2A1, MCM2, MCM4, MCM5, MMUT, MYO6, NOL3, PADI2, PFKM, PGD, PURA, RBBP7, RTCB, RUVBL2, SERPINH1, STMN1, TES, TUBB2A, UBC, VCP*
	*PARPBP*	Other	3	Activated highly under M1	2.449	4.52E-11	1.00E-04	*AKT1, ESR1, ESR2, estrogen receptor, NFkB (complex), PARPBP, PI3K (complex), PRL, STAT1, STAT4, STAT5A, STAT5B, TP53*	*ACADVL, ACTC1, ALDH3A2, ALDOA, CKM, COPA, COPE, DDX39B, ELN, ENAH, FAM120A, GBE1, GFPT2, HNRNPH2, KRT19, MACROH2A1, MCM2, MCM4, MCM5, MMUT, NOL3, PADI2, PFKM, PGD, PURA, RBBP7, RNF213, RUVBL2, S100A7A, SERPINH1, STMN1, TUBA1B, UBC, VCL, VCP*
	*HOXA1*	Transcription regulator	3	Activated highly under M1	2.646	5.38E-11	1.00E-04	*AKT1, EGFR, ESR1, ESR2, HOXA1, PI3K (complex), PPARA, PRL, RELA, STAT1, STAT4, STAT5A, STAT5B, TP53*	*ACADVL, ACTA1, ACTC1, ALDH3A2, ALDOA, CKM, COPA, COPE, DDX39B, ELN, ENAH, FAM120A, GBE1, GSTK1, HNRNPH2, KRT19, MACROH2A1, MCM2, MCM4, MCM5, MMUT, NOL3, NQO1, PADI2, PFKM, PGD, PURA, RNF213, RUVBL2, S100A7A, SERPINH1, STMN1, TUBA1B, UBC, VCL, VCP*
	*AMOT*	Other	3	Inhibited highly under M1 and M2	−2.646	7.02E-11	1.00E-04	*AKT1, AMOT, CDKN1A, CTNNB1, DYRK1A, ERBB4, GLI1, ITCH, Jnk, JUN, JUNB, LATS2, MAP3K7, NFkB (complex), RAF1, RELA, TAZ, TP53, TP63, TP73, YAP1*	*ACADVL, ACTA1, ACTC1, ALDOA, CNIH4, FAM120A, GBE1, GFPT2, HLA-, KRT10, KRT19, LDHB, MACROH2A1, MCM2, MCM4, MCM5, MMUT, MYL12A, MYO6, NOL3, NQO1, PADI2, PFKM, PGD, PURA, RBBP7, RTCB, SERPINH1, SF3B3, TUBB2A, UBC, VCL, VCP*
	*IP6K2*	Kinase	2	Inhibited highly under M1 and M2	−2.236	8.93E-11	1.00E-04	*Akt, AKT1, IP6K2, MAP3K7, STK11, TP53*	*ACADVL, ACTA1, ALDOA, CKM, ENO1, FAM120A, GSTK1, HLA-B, LDHB, MACROH2A1, MCM2, MCM4, MCM5, MMUT, MYO6, NOL3, NQO1, PADI2, PFKM, PGD, PURA, RBBP7, SERPINH1, STMN1, UBC, VCL, VCP*
	*APH-1*	Group	3	Activated highly under M1	2	9.86E-11	1.00E-04	*Akt, AKT1, APH-1, APH1A, APH1B, NOTCH1, PSEN1, PSEN2, Secretase gamma, TP53*	*ACADVL, ALDOA, CKM, ENO1, FAM120A, GSTK1, HLA-B, LDHB, MACROH2A1, MCM2, MCM4, MCM5, MMUT, MYO6, NOL3, NQO1, PADI2, PDXDC1, PFKM, PGD, PURA, RBBP7, SERPINH1, STMN1, UBC, VCL, VCP*
WM6 (blue)	*ASGR1*	Transmembrane receptor	3	Activated highly to M2	3.606	1.90E-11	1.00E-04	*AGT, AKT1, ASGR1, CCND1, CEBPB, CREB1, CTNNB1, DDIT3, EGFR, ERBB2, ERK, ERK1/2, ESR1, estrogen receptor, FOS, FOXO1, HBEGF, HIF1A, HRAS, JUN, MKNK1, MTOR, MYC, MYCN, NCOA2, NOS2, PGR, PLCB1, PPARG, PTPN11, SIRT1, SRF, TP53*	*AKR1A1, ARPC1B, ATP6V0D1, CAPZA2, CDK5RAP3, CLDN3, COL3A1, COL6A2, DPYSL2, FBL, GPD2, H2AC6, H2BC18, H4C1, H4C11, H4C12, H4C14, H4C15, H4C2, H4C3, H4C4, H4C6, H4C8, H4C9, HSPA2, HSPA5, HSPG2, LMAN2, MAPK1, MOGS, NKX2-1, PCK2, PGAM1, PRSS1, PSMA6, PTGFRN, RBM3, SEC22B, SELENBP1, SND1, TINAGL1, TKT, UBE2L3, UQCRC1*
	*CEBPB*	Transcription regulator	1	Activated commonly	3.317	1.78E-10	1.00E-04	*CEBPB*	*GFAP, H4C1, H4C11, H4C12, H4C14, H4C15, H4C2, H4C4, H4C6, H4C8, H4C9, PSMA6, SERPINA1*
	*APEX1*	Enzyme	2	Activated highly to M2	2	1.42E-06	9.00E-03	*APEX1, c-Src, HIF1A, SIRT1, TDG, temozolomide, TP53*	*ARPC1B, COL3A1, COL6A2, COX4I1, FTH1, GFAP, H2AC6, H2BC18, H4C15, HSPA5, HSPG2, LGALS3, LMAN2, MAPK1, NKX2-1, PCK2, RAP1B, RBM3, TINAGL1, TKT, UQCRC1*
	*Cbp/p300*	Group	2	Activated under both M1 and M2	2	1.74E-06	1.99E-02	*Cbp/p300, CTNNB1, EP300, ESR1, estrogen receptor, NCOA2, NCOA3, NFE2L2, NR3C2, PPARG, SMAD3, TP53*	*AKR1A1, ARPC1B, ATP6V0D1, CALD1, CLDN3, COL3A1, COL6A2, COX4I1, FTH1, GPD2, H4C15, H4C3, H4C6, HSPA5, HSPG2, LGALS3, LMAN2, MAPK1, MOGS, NKX2-1, PCK2, PSMA6, PSMD5, RBM3, SERPINA1, TINAGL1, UQCRC1*
	*BUB1*	Kinase	3	Activated highly to M2 but inhibited under NM	1.732	2.51E-07	3.80E-03	*APC, AURKB, BUB1, CTNNB1, GFAP, MTOR, PKM, TP53*	*AKR1A1, ARPC1B, COL3A1, COL6A2, COX4I1, FTH1, GFAP, H4C15, HSPA5, HSPG2, LGALS3, LMAN2, MAPK1, NKX2-1, PCK2, RBM3, SEC22B, SERPINA1, SND1, TINAGL1, TKT, UQCRC1*
	*MAPK10*	Kinase	2	Activated highly under M2 but inhibited under NM	1.732	1.40E-05	4.76E-02	*APP, CCND1, CDKN1A, JUN, L-serine, MAPK10, MAPKAPK3, NR3C1, TP53*	*ARPC1B, COL3A1, COL6A2, DPYSL2, FTH1, GFAP, H4C15, HSPA5, HSPG2, LGALS3, LMAN2, MAPK1, MOGS, PCK2, PRSS1, RBM3, SELENBP1, TINAGL1, TKT, TUFM, UQCRC1*
	*HSF1*	Transcription regulator	2	Inhibited commonly	−2.887	1.72E-11	1.00E-04	*CEBPB, EIF2A, ERK1/2, HSF1*	*COL3A1, COL6A2, GFAP, H4C1, H4C11, H4C12, H4C14, H4C15, H4C2, H4C4, H4C6, H4C8, H4C9, HSPA5, MAPK1, PCK2, PSMA6, SERPINA1*
	*HNRNPK*	Transcription regulator	2	Inhibited commonly	−2.887	1.06E-10	1.00E-04	*CEBPB, ERK1/2, HNRNPK, MAP2K1/2*	*COL3A1, GFAP, H4C1, H4C11, H4C12, H4C14, H4C15, H4C2, H4C4, H4C6, H4C8, H4C9, HSPA5, MAPK1, PSMA6, SERPINA1*
	*TGFB1*	Growth factor	2	Upregurated under M2	1.342	1.31E-07	8.20E-03	*AKT1, EGFR, ERBB2, FOXO1, HIF1A, IKBKB, JUN, MTOR, MYB, MYC, NR2C2, RICTOR, SRF, STAT4, STK11, TGFB1, TP53*	*ARPC1B, ATP6V0D1, CALD1, CAPZA2, CDK5RAP3, CLDN3, COL3A1, COL6A2, DHRS2, DPYSL2, FBL, FTH1, GFAP, H2AC6, H4C15, H4C3, HBG2, HSPA5, HSPG2, LGALS3, LMAN2, LMNB1, TA4H, MAPK1, MOGS, NKX2-1, PGAM1, PSMA6, RBM3, SEC22B, SELENBP1, SERPINA1, SND1, SYPL1, TINAGL1, TUFM, UBE2L3, UQCRC1*

a *Participating regulators are regulators through which the upstream regulator molecule controls the expression of target molecules in the dataset*.

b *Target molecules in the dataset are molecules in our dataset whose expression is potentially controlled by an upstream regulator*.

Regarding the WM10 module associated with the M1 trait, the *EGFR* L858R mutation, *PARPBP, HOXA1*, and *APH-1* were highly activated or upregulated under the *EGFR* L858R mutation, whereas *AMOT* was highly inhibited under both L858R and Ex19del mutations. *PARPBP* encodes poly (ADP-ribose) polymerase-1 (PARP-1) binding protein, which plays a central role in DNA repair and the maintenance of genomic stability, regulating DNA repair, and negatively double-strand break repair via homologous recombination. Xu et al. reported that PARPBP expression was enhanced in lung adenocarcinoma tissues and correlated with poor prognosis in lung adenocarcinoma patients (59) and also that its high expression was closely correlated with pathologic stages, suggesting its utility as an independent predictor in lung adenocarcinoma patients. *HOXA1* encodes homeobox protein Hox-1F, a member of the Homeobox (HOX) transcription factor family. HOXA1 mRNA and protein expression levels were significantly upregulated in breast cancer, and its overexpression was associated with poor prognosis and tumor progression in breast cancer patients (60). Anterior pharynx defective 1 (*APH1*) is the group *APH1A* and *APH1B*, which are the members of the gamma-secretase complex, comprising presenilin (*PSEN1* and *PSEN2*), anterior pharynx defective 1 (*APH1*), presenilin enhancer 2 (*PEN2*), and nicastrin. Gamma secretase substrates are known to include the four well-characterized mammalian Notch receptors (Notch1-4) and the five canonical transmembrane Notch ligands. Aberrant Notch activation drives development, tumorigenesis, and progression of lung cancer and is known to participate in resistance to anti-VEGF therapy (61). The inhibition of Notch activation by gamma-secretase inhibitors (GSIs) then could benefit NSCLC patients (62). Angiomotin (AMOT) and its related proteins, scaffold proteins, AMOT family proteins, were identified to have a strong interaction with the transcription factors Yes-associated protein (YAP) and TAZ (transcriptional coactivator with PDZ-binding motif) by tandem affinity purification (TAP) and mass spectrometry (63). Scaffold proteins angiomotin negatively regulated the transcription factors YAP and TAZ by preventing their nuclear translocation, suggesting a tumor-suppressing role of AMOT family proteins as components of the Hippo pathway. However, Hong reported the controversial results that AMOT may promote nuclear translocation of YAP and act as a transcriptional cofactor of the YAP-TEAD complex to facilitate the proliferation of epithelial cells and cancer development (64). It has been pointed out that the functional roles of AMOTs in different cancer types are controversial, highly depending on cell context (65).

Interestingly, among all causal networks predicted from the WM10 module, the downregulation of osimertinib intervention showed the highest significance in overlap *p*-value, in which *EGFR, ERBB2, ERBB3, ERBB4, BLK*, and *TNK* and their downstream pathways were maintained. In this study, we used FFPE tissue specimens collected from lung adenocarcinoma patients who did not receive any EGFR tyrosine kinase inhibitors, such as osimertinib. It has been reported that L858R-positive patients of NSCLC had a poor prognosis and difference in therapeutic outcome compared to Ex19del-positive patients (6). Moreover, the comparative IPA analysis predicted the *MNK1/2* causal network highly and differentially activated under the L858R mutation status (Table S1), which have been targeted by several chemical drug inhibitors for *EGFR* mutation-positive lung cancers. Those inhibitors, including dacomitinib, tomivosertib, BAY1143269, and ETC-1907206, have been developed for various types of *EGFR* mutation-positive cancers mainly including NSCLCs and various types of clinical trials are currently undergoing. Other top causal networks activated differentially under the *EGFR* L858R mutation included *max-myc* (complex), *MYC, F8, STK11*, and *RAD21*.

For the WM6 module associated with the M2 trait, the *EGFR* Ex19del mutation, *ASGR1* and *APEX1* were highly activated, and *BUB1, MAPK10*, and *TGFB1* were upregulated. *CEBPB* was activated commonly under all the traits, whereas *Cbp/p300* was activated under both Ex19del and L858R mutations. *ASGR1* encodes a subunit of the asialoglycoprotein receptor (*ASGR*) expressed in the extracellular region and a complex of the receptor and binding ligand is internalized. *ASGR* has been suggested to promote cancer metastasis by activating the EGFR–ERK pathway through interactions with counter-receptors on cancer cells, responding to endogenous lectins in the tumor microenvironment (66). *APEX1* (also known as *APE1, APX, HAP1*, and *REF1*) and encodes DNA-apurinic/apyrimidinic (AP) site endonuclease (protein names, such as APEN, APE-1, and REF-1), which plays a central role in the cellular response to oxidative stress, in which its two major activities are DNA repair and redox regulation of transcriptional factors. The elevated levels of APEX1 have been reported in several cancers, including lung cancer (67), and also to be associated with resistance to chemotherapy and radiotherapy in some cancers (68). *MAPK10* encodes mitogen-activated protein kinase 10 (also known as stress-activated protein kinase JNK3), which is involved in a wide variety of cellular processes, including stress response, proliferation, differentiation, transcription regulation, and development. *MAPK10* functions as a tumor suppressor and the deletion of this proapoptotic gene would favor the survival and proliferation of cancer cells (69). *BUB1* encodes mitotic checkpoint serine/threonine-protein kinase BUB1 or budding uninhibited by benzimidazoles 1 (Bub1), which is required for chromosome alignment and resolution of spindle attachment errors but does not play a major role in the spindle assembly checkpoint (SAC) activity. Overexpression of Bub1 in breast cancer is associated with a poor clinical prognosis (70). Recent tumor xenograft studies suggested that the Bub1 kinase inhibitor BAY 1816032 in combination with taxanes or PARP inhibitors enhanced their efficacy and suppressed the development of therapy resistance (71). *CEBPB* encodes CCAAT/enhancer-binding protein beta (C/EBP beta), which is important in the regulation of genes involved in immune and inflammatory responses. C/EBP beta induces elevated IL-6 expression levels frequently observed in human lung adenocarcinomas (72) and interacts with peroxisome proliferator-activated receptor-gamma (*PPARG*) involved in pathways of transcriptional misregulation in cancer (73). The study using the inducible EGFR T790M-L858R transgenic mouse models suggested that C/EBP beta is dispensable for lung tumorigenesis in *EGFR*-driven murine lung cancer (73).

## Discussion

Outcomes of lung adenocarcinoma patients receiving EGFR TKIs were reported to be affected depending on the types of *EGFR* gatekeeper mutation (6, 74), which are serious clinical challenges. Targeting disease-associated dual core networks rather than targeting a single protein (gene) as in conventional approaches is expected to greatly improve the outcomes of individual patients, such as efficacy and safety, in line with the concept of precision medicine. Such a concept, so-called network pharmacology, was first proposed by Hopkins (75), which aims to induce synthetic lethality by targeting dual hub molecules involved in different disease core networks. We have first conducted a mutant proteomic analysis for clinical tissue specimens of 36 lung adenocarcinoma patients who harbored distinct *EGFR* mutations, Ex21 L858R, Ex19del, and no L858R/Ex19del. Disease-related network modules are elucidated from mutant protein expression data sets, which would be potentially associated with the activation of downstream and/or upstream networks affected under distinct *EGFR* mutations. In particular, this study focuses on influence in disease-related networks of lung adenocarcinoma, which would take place under the L858R mutation. Our analytical workflow combining WGCNA with ORA-screening identified several mutant protein modules significantly overlapping with upregulated mutant proteins under the *EGFR* L858R mutation.

Our goal with the present study was to apply an unbiased bioinformatic method to characterize the mutant profiles of detectable SAAVs after filtering with stringent criteria of database identifications in pathologically well-described patient samples. Mass spectrometry-based proteomic data is widely recognized as an information-rich source of uniquely expressed proteoforms, but tandem mass spectra interpretation is dependent on fragmentation efficiency and identification strategies. Because the number of subjects was limited in each patient group, we presented quality control data in the Supplementary Material demonstrating the overall homogeneity of the mass spectrometric data due to low technical variability of sample preparation and data acquisition. Careful interpretation of the findings highlighted potential differences between phenotypes, which suggests that different oncogenic driver *EGFR* mutations would affect activation or inactivation of their downstream disease-related molecular networks, which are often associated with protein mutations.

Surprisingly, the OPLS-DA performed for identified mutant proteins demonstrated profound differences in distance among the different *EGFR* mutation groups, L858R, Ex19del, and no L858R/Ex19del, suggesting that cancer cells harboring L858R or Ex19del emerge from cellular origins differently from L858R/Ex19del-negative cells (Figure 2B). Aberrant cells would, thus, emerge as a subpopulation of tumor cells of genetic intratumor heterogeneity, which would rapidly grow and predominantly survive by disrupting the tumor environment. To confirm our observation, a further large-scale investigation with genomic alteration analysis by next-generation sequencing (NGS) is required.

**Figure 8:**
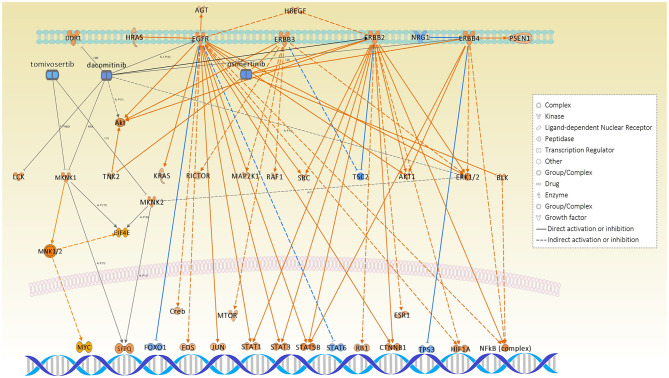
The causal networks of downregulated osimertinib intervention and activated *MNK1/2*, which were predicted to be significant under L858R mutation status, illustrated together with the related inhibitors: dacomitinib and tomivosertib.

The pathways of the carboxylic acid metabolic process, cell cycle, developmental biology, and immune system were centrally associated under the L858R mutation. The top IPA causal networks predicted for the representative mutant protein module-WM10 were associated with the regulation of DNA repair, cancer development, tumorigenesis, and maintenance of genomic stability as well as therapeutic resistance. Interestingly, the downregulation of osimertinib intervention showed the highest significance rank in overlap among all causal networks predicted from the WM10 module (Table 2). This finding might suggest the potential usefulness of osimertinib to be revisited for the L858R-positive patients of lung adenocarcinoma. Both the causal networks of osimertinib intervention and *MNK1/2* identified significantly and differentially, respectively, may evidence disease mechanisms associated with *EGFR* mutation-positive lung adenocarcinoma (Figure 8).

The pathways of cellular responses to stress, mitotic prophase, cell proliferation, growth, migration, epithelial to mesenchymal transition (EMT), and immune system process were mostly involved under the Ex19del mutation. The IPA causal networks elucidated for the representative mutant protein module, WM6, seem to be associated dominantly with the EGFR–ERK pathway. The pathways related to the Hippo pathway and tumorigenesis were commonly involved under both L858R and Ex19del mutations.

The limitations of this study are as follows: first, the number of patients examined is limited to be 36, which was attributed to collect the homogeneous tumor-derived samples with the best effort. Second, genomic alteration analysis was not conducted for the same samples.

In conclusion, we successfully applied WGCNA combined with ORA-based protein screening to clinical mutant proteomic data sets from 36 patients of lung adenocarcinoma. The proteomic discovery method detecting mutant proteoforms has revealed specific profiles distinguishing the phenotypically characterized patient groups. Our results could confirm the usefulness of mutant proteomics to identify activated or inactivated disease-related mutant protein networks affected under distinct *EGFR* mutations. Verification and quantitative analysis of these molecular features in an independent patient cohort are yet to be undertaken by either using targeted proteomics or RNAseq and combining the resulting data in a systems biology approach. Additionally, our findings may help in the development of therapeutic strategies to improve patient outcomes. Differences in mutant proteomes between L858R and Ex19del mutation cells help to demonstrate the difference in efficacy of various EGFR-TKIs. Further verifications with a greater number of patient samples and targeted analysis of mutant proteoforms throughout the cohorts are planned in follow-up studies to achieve a better understanding of the expression profiles of SAAVs in phenotypic groups and establish a relationship between the detected networks in connection to disease progression.

## Data Availability Statement

The unfiltered mass spectrometry data sets generated and analyzed in this study have been deposited in the ProteomeXchange (http://proteomecentral.proteomexchange.org) and jPOST with the data set identifiers PXD015862 and JPST000687, respectively.

## Ethics Statement

FFPE tumor tissue blocks from 36 surgical specimens of lung adenocarcinomas with known *EGFR* mutation statuses were obtained without patient identifiers from St. Marianna University School of Medicine Hospital. Informed consent was obtained from all participating subjects, and the protocol was approved by the institutional review board of St. Marianna University School of Medicine (approval no. 3569) and was conducted in accordance with the Helsinki Declaration.

## Author Contributions

TN and ÁV conceptualized this study, designed the bioinformatics methodology, wrote the main manuscript text, prepared Figures 1–7 and Supplementary Information Files, and wrote the first draft of the manuscript. ÁV performed the identification of mutant protein from the clinical proteome raw data sets. TN analyzed the mutant proteome data sets by using weighted gene coexpression network analysis (WGCNA), over representative analysis, and the ingenuity pathway analysis software. TN, HN, HK, and HS initiated and managed the collaboration. All authors reviewed the manuscript.

## Conflict of Interest

The authors declare that the research was conducted in the absence of any commercial or financial relationships that could be construed as a potential conflict of interest.

## Supplementary Material

Supplementary Information File 1 Search summaries, including score distributions and statistical data are presented using the PEAKS reporting function.

Supplementary Information File 2 The complete lists of identified mutant protein sequences after filtration.

Figure S1 Geneontology (GO) analysis of the identified proteins to the three traits-M1 (L858R), M2 (Ex19del), and NM (no L858R/Ex19del). **(A)** GO Biological process. 1, developmental process (GO:0032502); 2, multicellular organismal process (GO:0032501); 3, cellular process (GO:0009987); 4, reproduction (GO:0000003); 5, cell population proliferation (GO:0008283); 6, localization (GO:0051179); 7, reproductive process (GO:0022414); 8, multiorganism process (GO:0051704); 9, biological adhesion (GO:0022610); 10, immune system process (GO:0002376); 11, cellular component organization or biogenesis (GO:0071840); 12, biological regulation (GO:0065007); 13, growth (GO:0040007); 14, signaling (GO:0023052); 15, metabolic process (GO:0008152); 16, response to stimulus (GO:0050896); 17, pigmentation (GO:0043473); 18, behavior (GO:0007610); 19, locomotion (GO:0040011). **(B)** GO Molecular function. 1, translation regulator activity (GO:0045182); 2, transcription regulator activity (GO:0140110); 3, molecular transducer activity (GO:0060089); 4, binding (GO:0005488); 5, structural molecule activity (GO:0005198); 6, molecular function regulator (GO:0098772); 7, catalytic activity (GO:0003824); 8, transporter activity (GO:0005215). **(C)** GO Cellular component. 1, synapse part (GO:0044456); 2, membrane part (GO:0044425); 3, membrane (GO:0016020); 4, synapse (GO:0045202); 5, organelle part (GO:0044422); 6, extracellular region part (GO:0044421); 7, cell junction (GO:0030054); 8, membrane-enclosed lumen (GO:0031974); 9, protein-containing complex (GO:0032991); 10, supramolecular complex (GO:0099080); 11, extracellular region (GO:0005576); 12, cell (GO:0005623); 13, cell part (GO:0044464); 14, organelle (GO:0043226). **(D)** GO Protein class. 1, extracellular matrix protein (PC00102); 2, cytoskeletal protein (PC00085); 3, transporter (PC00227); 4, scaffold/adaptor protein (PC00226); 5, cell adhesion molecule (PC00069); 6, nucleic acid binding protein (PC00171); 7, intercellular signal molecule (PC00207); 8, protein-binding activity modulator (PC00095); 9, calcium-binding protein (PC00060); 10, gene-specific transcriptional regulator (PC00264); 11, defense/immunity protein (PC00090); 12, translational protein (PC00263); 13, metabolite interconversion enzyme (PC00262); 14, protein modifying enzyme (PC00260); 15, chromatin/chromatin-binding, or -regulatory protein (PC00077); 16, transfer/carrier protein (PC00219); 17, membrane traffic protein (PC00150); 18, chaperone (PC00072); 19, cell junction protein (PC00070); 20, structural protein (PC00211); 21, storage protein (PC00210); 22, transmembrane signal receptor (PC00197).

Figure S2 Relationship between module eigen proteins and the L858R and Ex19del mutations in the *EGFR* gene. Each row in the embedded table represents weighted gene coexpression network analysis results for each module. The first and second columns in the table represent module ID and color name of the module. The third column represents the number of proteins in each module. The fourth, fifth, and sixth (seventh, eighth, and ninth) columns indicate the correlation coefficients (*p*-values of the correlation coefficients) between the corresponding modules and the clinical traits. The table is color-coded by correlation coefficient according to the color legend on the right side of the figure. The intensity and direction of the correlations are indicated on the right side of the heat map (red, positive correlation; blue, negative correlation). *p*-values (<0.10) are highlighted in red.

Figure S3 *R*_*SC*_ values between M1 and M2 calculated for proteins identified (X-axis). Mutant proteins upregulated with 2-fold changes for M1 (*R*_*SC*_ ≥ 1) and M2 (*R*_*SC*_ ≤ −1) are denoted.

Table S1 The comparative analysis results of causal networks predicted for mutant proteins expressed commonly (see Venn map in Figure 2A). *MNK1/2, Max-Myc*, MYC, *XBP1, BTG2, F8, STK11*, and *RAD21* were highly activated (*z*-score > 2.5) and differentially under M1 (L858R).

## References

[1] Lynch TJ Bell DW Sordella R Gurubhagavatula S Okimoto RA Brannigan BW. Activating mutations in the epidermal growth factor receptor underlying responsiveness of non-small-cell lung cancer to gefitinib. N Engl J Med. (2004) 350:2129–39. 10.1056/NEJMoa040938 15118073

[2] Paez JG Jänne PA Lee JC Tracy S Greulich H Gabriel S. EGFR mutations in lung cancer: correlation with clinical response to gefitinib therapy. Science. (2004) 304:1497–500. 10.1126/science.1099314 15118125

[3] Kobayashi Y Mitsudomi T. Not all epidermal growth factor receptor mutations in lung cancer are created equal: perspectives for individualized treatment strategy. Cancer Sci. (2016) 107:1179–86. 10.1111/cas.12996 27323238 PMC5021039

[4] Kobayashi S Boggon TJ Dayaram T Jänne PA Kocher O Meyerson M. EGFR mutation and resistance of non-small-cell lung cancer to gefitinib. N Engl J Med. (2005) 352:786–92. 10.1056/NEJMoa044238 15728811

[5] Mok TS Wu Y-L Ahn M-J Garassino MC Kim HR Ramalingam SS. Osimertinib or platinum-pemetrexed in EGFR T790M-positive lung cancer. N Engl J Med. (2017) 376:629–40. 10.1056/NEJMoa1612674 27959700 PMC6762027

[6] Soria JC Ohe Y Vansteenkiste J Reungwetwattana T Chewaskulyong B Lee KH. Osimertinib in untreated EGFR-mutated advanced non-small-cell lung cancer. N Engl J Med. (2018) 378:113–25. 10.1056/NEJMoa1713137 29151359

[7] Ramalingam SS Vansteenkiste J Planchard D Cho BC Gray JE Ohe Y. Osimertinib vs comparator EGFR-TKI as first-line treatment for EGFRm advanced NSCLC (FLAURA): final overall survival analysis. Ann Oncol. (2019) 30:v914–15. 10.1093/annonc/mdz394.07630508196

[8] Forcella M Oldani M Epistolio S Freguia S Monti E Fusi P. Non-small cell lung cancer (NSCLC), EGFR downstream pathway activation and TKI targeted therapies sensitivity: effect of the plasma membrane-associated NEU3. PLoS ONE. (2017) 12:e0187289. 10.1371/journal.pone.0187289 29088281 PMC5663482

[9] Torres AF Nogueira C Magalhaes J Costa IS Aragao A Neto AG. Expression of EGFR and molecules downstream to PI3K/Akt, Raf-1-MEK-1-MAP (Erk1/2), and JAK (STAT3) pathways in invasive lung adenocarcinomas resected at a single institution. Anal Cell Pathol (Amst). (2014) 2014:352925. 10.1155/2014/352925 25763322 PMC4334032

[10] Azevedo AP Silva S Rueff J Non-receptor Tyrosine Kinases Role and Significance in Hematological Malignancies. (2019). 10.5772/intechopen.84873

[11] Han G Feng J Peng M Verma V Bi J Song Q EGFR overexpression and mutations lead to a change in biological characteristics of human lung adenocarcinoma cells. Int J Radiation Oncol. (2017) 99:E594 10.1016/j.ijrobp.2017.06.2031

[12] Wee P Wang Z. Epidermal growth factor receptor cell proliferation signaling pathways. Cancers. (2017) 9:52. 10.3390/cancers9050052 28513565 PMC5447962

[13] Morgillo F Della Corte CM Fasano M Ciardiello F. Mechanisms of resistance to EGFR-targeted drugs: lung cancer. ESMO Open. (2016) 1:e000060. 10.1136/esmoopen-2016-000060 27843613 PMC5070275

[14] Liu Q Yu S Zhao W Qin S Chu Q Wu K. EGFR-TKIs resistance via EGFR-independent signaling pathways. Mol Cancer. (2018) 17:53. 10.1186/s12943-018-0793-1 29455669 PMC5817859

[15] Peng S Wang R Zhang X Ma Y Zhong L Li K. EGFR-TKI resistance promotes immune escape in lung cancer via increased PD-L1 expression. Mol Cancer. (2019) 18:165. 10.1186/s12943-019-1073-4 31747941 PMC6864970

[16] Yu H Pardoll D Jove R. STATs in cancer inflammation and immunity: a leading role for STAT3. Nat Rev Cancer. (2009) 9:798–809. 10.1038/nrc2734 19851315 PMC4856025

[17] Yan S Li Z Thiele CJ. Inhibition of STAT3 with orally active JAK inhibitor, AZD1480, decreases tumor growth in neuroblastoma and pediatric sarcomas *in vitro* and *in vivo*. Oncotarget. (2013) 4:433–45. 10.18632/oncotarget.930 23531921 PMC3717306

[18] Wu K Chang Q Lu Y Qiu P Chen B Thakur C. Gefitinib resistance resulted from STAT3-mediated Akt activation in lung cancer cells. Oncotarget. (2013) 4:2430–8. 10.18632/oncotarget.1431 24280348 PMC3926838

[19] Jin Y Bao H Le X Fan X Tang M Shi X. Distinct co-acquired alterations and genomic evolution during TKI treatment in non-small-cell lung cancer patients with or without acquired T790M mutation. Oncogene. (2020) 39:1846–59. 10.1038/s41388-019-1104-z 31754213

[20] Harrison PT Vyse S Huang PH. Rare epidermal growth factor receptor (EGFR) mutations in non-small cell lung cancer. Semin Cancer Biol. (2020) 61:167–79. 10.1016/j.semcancer.2019.09.015 31562956 PMC7083237

[21] Yates JR. The revolution and evolution of shotgun proteomics for large-scale proteome analysis. J Am Chem Soc. (2013) 135:1629–40. 10.1021/ja3094313 23294060 PMC3751590

[22] Tong J Taylor P Moran MF. Proteomic analysis of the epidermal growth factor receptor (EGFR) interactome and post-translational modifications associated with receptor endocytosis in response to EGF and stress. Mol Cell Proteomics. (2014) 13:1644–58. 10.1074/mcp.M114.038596 24797263 PMC4083106

[23] Putri DU Chiumia FK Jheng YT Han CL The role of proteomics for dissecting aberrant molecular signaling pathways upon Egfr-Tki treatments in non-small cell lung cancer. Proteomics Bioinform Curr Res. (2019) 1:4–16.

[24] Zhang X Belkina N Jacob HK Maity T Biswas R Venugopalan A. Identifying novel targets of oncogenic EGF receptor signaling in lung cancer through global phosphoproteomics. Proteomics. (2015) 15:340–55. 10.1002/pmic.201400315 25404012 PMC6461560

[25] Zhang X Maity T Kashyap MK Bansal M Venugopalan A Singh S. Quantitative tyrosine phosphoproteomics of epidermal growth factor receptor (EGFR) tyrosine kinase inhibitor-treated lung adenocarcinoma cells reveals potential novel biomarkers of therapeutic response. Mol Cell Proteomics. (2017) 16:891–910. 10.1074/mcp.M117.067439 28331001 PMC5417828

[26] Végvári Á Mutant proteogenomics. In: Végvári Á editor. Proteogenomics. Cham: Springer (2016). p. 77–91. 10.1007/978-3-319-42316-6_6

[27] The International HapMap Consortium. A second generation human haplotype map of over 3.1 million SNPs. Nature. (2007) 449:851–61. 10.1038/nature06258 17943122 PMC2689609

[28] Nishimura T Nakamura H Végvári Á Marko-Varga G Furuya N Saji H. Current status of clinical proteogenomics in lung cancer. Expert Rev Proteomics. (2019) 16:761–72. 10.1080/14789450.2019.1654861 31402712

[29] Wood LD Parsons DW Jones S Lin J Sjöblom T Leary RJ. The genomic landscapes of human breast and colorectal cancers. Science. (2007) 318:1108–13. 10.1126/science.1145720 17932254

[30] Sun T Zhou Y Yang M Hu Z Tan W Han X. Functional genetic variations in cytotoxic T-lymphocyte antigen 4 and susceptibility to multiple types of cancer. Cancer Res. (2008) 68:7025–34. 10.1158/0008-5472.CAN-08-0806 18757416

[31] Yan H Yuan W Velculescu VE Vogelstein B Kinzler KW. Allelic variation in human gene expression. Science. (2002) 297:1143. 10.1126/science.1072545 12183620

[32] Végvári A Sjödin K Rezeli M Malm J Lilja H Laurell T. Identification of a novel proteoform of prostate specific antigen (SNP-L132I) in clinical samples by selective reaction monitoring. Mol Cell Proteomics. (2013) 12:2761–73. 10.1074/mcp.M113.028365 23842001 PMC3790289

[33] Wang Q Chaerkady R Wu J Hwang HJ Papadopoulos N Kopelovich L. Mutant proteins as cancer-specific biomarkers. Proc Natl Acad Sci USA. (2011) 108:2444–9. 10.1073/pnas.1019203108 21248225 PMC3038743

[34] Kawamura T Nomura M Tojo H Fujii K Hamasaki H Mikami S. Proteomic analysis of laser microdissected paraffin-embedded tissues: (1) Stage-related protein candidates upon non-metastatic lung adenocarcinoma. J Proteomics. (2010) 73:1089–99. 10.1016/j.jprot.2009.11.011 19948256

[35] Fujii K Miyata Y Takahashi I Koizumi H Saji H Hoshikawa M. Differential proteomic analysis between small cell lung carcinoma (SCLC) and pulmonary carcinoid tumors reveals molecular signatures for malignancy in lung cancer. Proteomics Clin Appl. (2018) 12:e1800015. 10.1002/prca.201800015 29888431

[36] Langfelder P Horvath S. WGCNA: an R package for weighted correlation network analysis. BMC Bioinformatics. (2008) 9:559. 10.1186/1471-2105-9-559 19114008 PMC2631488

[37] Tang Y Ke ZP Peng YG Cai PT. Coexpression analysis reveals key gene modules and pathways of human coronary heart disease. J Cell Biochem. (2018) 119:2102–9. 10.1002/jcb.26372 28857241

[38] Nakamura H Fujii K Gupta V Hata H Koizumu H Hoshikawa M. Identification of key modules and hub genes for small-cell lung carcinoma and large-cell neuroendocrine lung carcinoma by weighted gene co-expression network analysis of clinical tissue-proteomes. PLoS ONE. (2019) 14:e0217105. 10.1371/journal.pone.0217105 31166966 PMC6550379

[39] Travis WD Brambilla E Nicholson AG Yatabe Y Austin JHM Beasley MB. The 2015 World Health Organization classification of lung tumors: impact of genetic, clinical and radiologic advances since the 2004 classification. J Thorac Oncol. (2015) 10:1243–60. 10.1097/JTO.0000000000000630 26291008

[40] Old WM Meyer-Arendt K Aveline-Wolf L Pierce KG Mendoza A Sevinsky JR. Comparison of label-free methods for quantifying human proteins by discovery proteomics. Mol Cell Proteomics. (2005) 4:1487–502. 10.1074/mcp.M500084-MCP200 15979981

[41] Zhang J Xin L Shan B Chen W Xie M Yuen D. PEAKS DB: *de novo* sequencing assisted database search for sensitive and accurate peptide identification. Mol Cell Proteomics. (2012) 11:M111.010587. 10.1074/mcp.M111.010587 22186715 PMC3322562

[42] Szklarczyk D Gable AL Lyon D Junge A Wyder S Huerta-Cepas J. et al. STRING v11:protein-protein association networks with increased coverage, supporting functional discovery in genome-wide experimental datasets. Nucleic Acids Res. (2019) 47:D607–13. 10.1093/nar/gky1131 30476243 PMC6323986

[43] Krämer A Green J Pollard J Jr. Tugendreich S. Causal analysis approaches in ingenuity pathway analysis. Bioinformatics. (2014) 30:523–30. 10.1093/bioinformatics/btt703 24336805 PMC3928520

[44] Mi H Muruganujan A Huang X Ebert D Mills C Guo X. Protocol update for large-scale genome and gene function analysis with the PANTHER classification system (v.14.0). Nat Protoc. (2019) 14:703–21. 10.1038/s41596-019-0128-8 30804569 PMC6519457

[45] Bylesjö M Rantalainen M Cloarec O Nicholson JK Holmes E Trygg J OPLS discriminant analysis: combining the strengths of PLS-DA and SIMCA classification. J Chemometrics. (2006) 20:341–51. 10.1002/cem.1006

[46] Khomtchouk BB Hennessy JR Wahlestedt C. shinyheatmap: Ultra fast low memory heatmap web interface for big data genomics. PLoS ONE. (2017) 12:e0176334. 10.1371/journal.pone.0176334 28493881 PMC5426587

[47] Yan X Chang J Sun R Meng X Wang W Zeng L. DHX9 inhibits epithelial-mesenchymal transition in human lung adenocarcinoma cells by regulating STAT3. Am J Transl Res. (2019) 11:4881–94. 31497206 PMC6731401

[48] Thompson E SAMHD1 is a novel biomarker and therapeutic target for radiation therapy and PARP inhibition in breast cancer. Cancer Res. (2019) 79:Abstract nr 3497. 10.1158/1538-7445.AM2019-3497

[49] Rentoft M Lindell K Tran P Chabes AL Buckland RJ Watt DL. Heterozygous colon cancer-associated mutations of SAMHD1 have functional significance. Proc Natl Acad Sci USA. (2016) 113:4723–28. 10.1073/pnas.1519128113 27071091 PMC4855590

[50] Herold N Rudd SG Sanjiv K Kutzner J Myrberg IH Paulin CBJ. With me or against me: tumor suppressor and drug resistance activities of SAMHD1. Exp Hematol. (2017) 52:32–9. 10.1016/j.exphem.2017.05.001 28502830

[51] Zhuo DX Zhang XW Jin B Zhang Z Xie BS Wu CL. CSTP1, a novel protein phosphatase, blocks cell cycle, promotes cell apoptosis, and suppresses tumor growth of bladder cancer by directly dephosphorylating Akt at Ser473 site. PLoS ONE. (2013) 8:e65679. 10.1371/journal.pone.0065679 23799035 PMC3684612

[52] Wu J1 Jiang H Luo S Zhang M Zhang Y Sun F. Caspase-mediated cleavage of C53/LZAP protein causes abnormal microtubule bundling and rupture of the nuclear envelope. Cell Res. (2013) 23:691–704. 10.1038/cr.2013.36 23478299 PMC3641598

[53] Tan D Li Q Deeb G Ramnath N Slocum HK Brooks J. Thyroid transcription factor-1 expression prevalence and its clinical implications in non-small cell lung cancer: a high-throughput tissue microarray and immunohistochemistry study. Hum Pathol. (2003) 34:597–604. 10.1016/S0046-8177(03)00180-1 12827614

[54] Tanaka H Yanagisawa K Shinjo K Taguchi A Maeno K Tomida S. Lineage-specific dependency of lung adenocarcinomas on the lung development regulator TTF-1. Cancer Res. (2007) 67:6007–11. 10.1158/0008-5472.CAN-06-4774 17616654

[55] Zu YF Wang XC Chen Y Wang JY Liu X Li X. Thyroid transcription factor 1 represses the expression of Ki-67 and induces apoptosis in non-small cell lung cancer. Oncol Rep. (2012) 28:1544–50. 10.3892/or.2012.2009 22940844 PMC3583567

[56] Wang Z Liu Y Zhang P Zhang W Wang W Curr K. FAM83D promotes cell proliferation and motility by downregulating tumor suppressor gene FBXW7. Oncotarget. (2013) 4:2476–86. 10.18632/oncotarget.1581 24344117 PMC3926842

[57] Inamura K Shimoji T Ninomiya H Hiramatsu M Okui M Satoh Y. A metastatic signature in entire lung adenocarcinomas irrespective of morphological heterogeneity. Hum Pathol. (2007) 38:702–9. 10.1016/j.humpath.2006.11.019 17376511

[58] Shi R Sun J Sun Q Zhang Q Xia W Dong G. Upregulation of FAM83D promotes malignant phenotypes of lung adenocarcinoma by regulating cell cycle. Am J Cancer Res. (2016) 6:2587–98. 27904773 PMC5126275

[59] Xu D Tao Z Tang X He JK. Poly (ADP-ribose) polymerase-1 binding protein facilitates lung adenocarcinoma cell proliferation and correlates with poor prognosis. Ann Clin Lab Sci. (2019) 49:574–80. 31611199

[60] Liu J Liu J Lu X. HOXA1 upregulation is associated with poor prognosis and tumor progression in breast cancer. Exp Ther Med. (2019) 17:1896–902. 10.3892/etm.2018.7145 30783466 PMC6364196

[61] Li JL Sainson RC Oon CE Turley H Leek R Sheldon H. DLL4-Notch signaling mediates tumor resistance to anti-VEGF therapy *in vivo*. Cancer Res. (2011) 71:6073–83. 10.1158/0008-5472.CAN-11-1704 21803743

[62] Pine SR. Rethinking gamma-secretase inhibitors for treatment of non-small-cell lung cancer: is notch the target? Clin Cancer Res. (2018) 24:6136–41. 10.1158/1078-0432.CCR-18-1635 30104200 PMC6295228

[63] Zhao B1 Li L Lu Q Wang LH Liu CY Lei Q. Angiomotin is a novel Hippo pathway component that inhibits YAP oncoprotein. Genes Dev. (2011) 25:51–63. 10.1101/gad.2000111 21205866 PMC3012936

[64] Hong W. Angiomotin'g YAP into the nucleus for cell proliferation and cancer development. Sci. Signal. (2013) 6:pe27. 10.1126/scisignal.2004573 24003252

[65] Huang T Zhou Y Zhang J Cheng ASL Yu J To KF. The physiological role of Motin family and its dysregulation in tumorigenesis. J Transl Med. (2018) 16:98. 10.1186/s12967-018-1466-y 29650031 PMC5898069

[66] Ueno S Mojic M Ohashi Y Higashi N Hayakawa Y Irimura T. Asialoglycoprotein receptor promotes cancer metastasis by activating the EGFR-ERK pathway. Cancer Res. (2011) 71:6419–27. 10.1158/0008-5472.CAN-11-1773 21868757

[67] Kakolyris S Giatromanolaki A Koukourakis M Kaklamanis L Kanavaros P Hickson ID. Nuclear localization of human AP endonuclease 1 (HAP1/Ref-1) associates with prognosis in early operable non-small cell lung cancer (NSCLC). J Pathol. (1999) 189:351–7. 10.1002/(SICI)1096-9896(199911)189:3<351::AID-PATH435>3.0.CO;2-1 10547596

[68] Tell G Quadrifoglio F Tiribelli C Kelley MR. The many functions of APE1/Ref-1: not only a DNA repair enzyme. Antioxid Redox Signal. (2009) 11:601–20. 10.1089/ars.2008.2194 18976116 PMC2811080

[69] Xie Y Liu Y Fan X Zhang L Li Q Li S. MicroRNA-21 promotes progression of breast cancer via inhibition of mitogen-activated protein kinase10 (MAPK10). Biosci Rep. (2019). 10.1042/BSR20181000. [Epub ahead of print]. 31375553

[70] Takagi K Miki Y Shibahara Y Nakamura Y Ebata A Watanabe M. BUB1 immunolocalization in breast carcinoma: its nuclear localization as a potent prognostic factor of the patients. Horm Cancer. (2013) 4:92–102. 10.1007/s12672-012-0130-x 23288590 PMC10358026

[71] Siemeister G Mengel A Fernández-Montalván AE Bone W Schröder J Zitzmann-Kolbe S. Inhibition of BUB1 kinase by BAY 1816032 sensitizes tumor cells toward taxanes, ATR, and PARP inhibitors *in vitro* and *in vivo*. Clin Cancer Res. (2019) 25:1404–14. 10.1158/1078-0432.CCR-18-0628 30429199

[72] Meng X Lu P Bai H Xiao P Fan Q. Transcriptional regulatory networks in human lung adenocarcinoma. Mol Med Rep. (2012) 6:961–6. 10.3892/mmr.2012.1034 22895549

[73] Cai Y Hirata A Nakayama S VanderLaan PA Levantini E Yamamoto M. CCAAT/enhancer binding protein β is dispensable for development of lung adenocarcinoma. PLoS ONE. (2015) 10:e0120647. 10.1371/journal.pone.0120647 25767874 PMC4358974

[74] Saito H Fukuhara T Furuya N Watanabe K Sugawara S Iwasawa S. Erlotinib plus bevacizumab versus erlotinib alone in patients with EGFR-positive advanced non-squamous non-small-cell lung cancer (NEJ026): interim analysis of an open-label, randomized, multicentre, phase 3 trial. Lancet Oncol. (2019) 20:625–35. 10.1016/S1470-2045(19)30035-X 30975627

[75] Hopkins AL. Network pharmacology: the next paradigm in drug discovery. Nat Chem Biol. (2008) 4:682–90. 10.1038/nchembio.118 18936753

