# Supplementary material for: Mutant Proteomics of Lung Adenocarcinomas Harboring Different EGFR Mutations
Source: Front Oncol. 2020 Aug 25;10:1494. doi: 10.3389/fonc.2020.01494 (PMC7477350; doi:10.3389/fonc.2020.01494)
Supplement: Supplementary Information File 1 — Search summaries, including score distributions and statistical data are presented using the PEAKS reporting function. [file Data_Sheet_1.PDF]

1. Notes

2. Result Statistics

Figure 1. False discovery rate (FDR) curve. X axis is the number of peptide-spectrum matches (PSM) being kept. Y axis is the corresponding FDR.

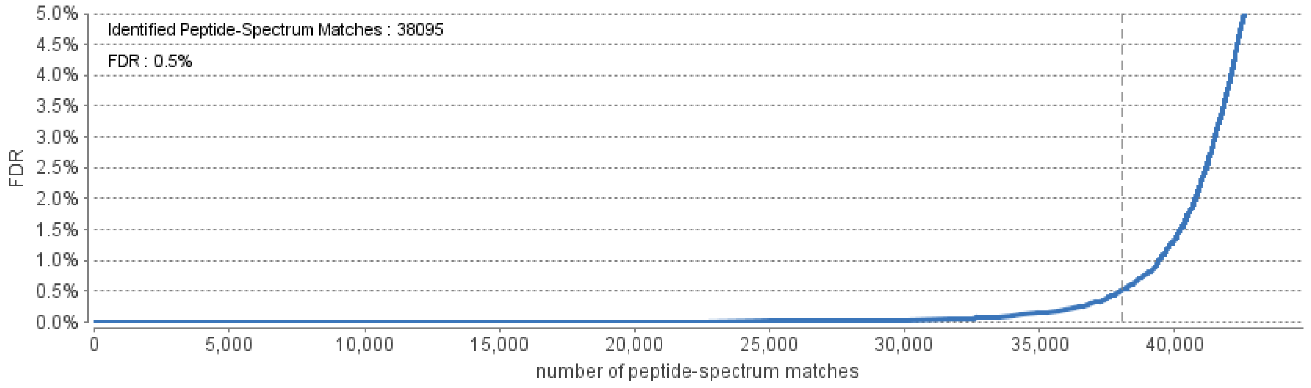

Figure 2. PSM score distribution. **(a)** Distribution of PEAKS peptide score; **(b)** Scatterplot of PEAKS peptide score versus precursor mass error.

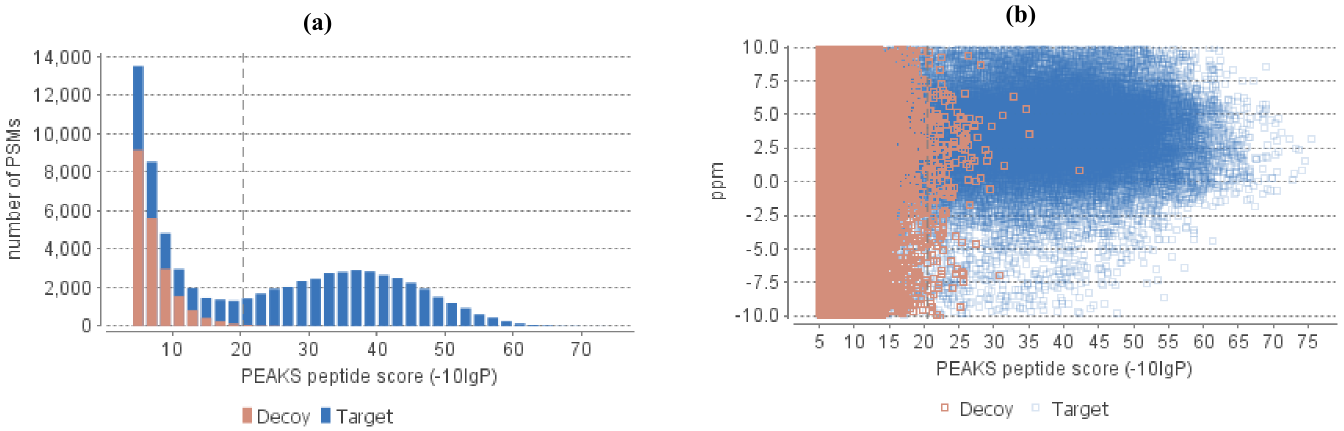

Figure 3. De novo result validation. Distribution of residue local confidence: **(a)** Residues in de novo sequences validated by confident database peptide assignment; **(b)** Residues in "de novo only" sequences.

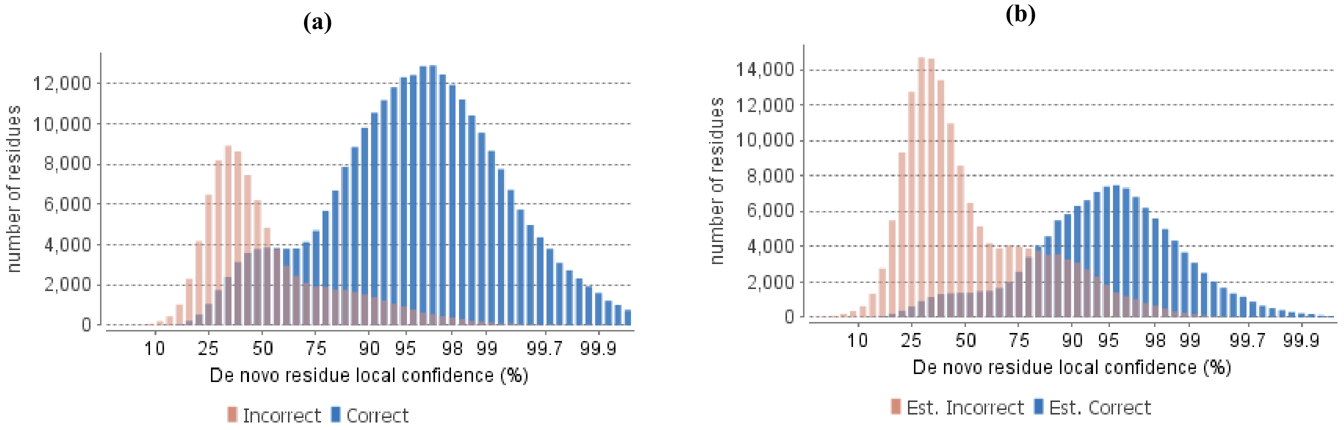

Table 1. Statistics of data.

# of MS scans	25619

Table 4. PTM profile.

Name	ΔMass	Position	#PSM	-10lgP	Area	AScor[illegible]

# of MS/MS scans 134251

Table 2. Result filtration parameters.

Peptide -10lgP	≥20.4
Peptide Ascore	≥0
Protein -10lgP	≥20
Proteins unique peptides	≥0
De novo ALC Score	≥50%

Table 3. Statistics of filtered result.

Peptide-Spectrum Matches	38095
Peptide sequences	14272
Protein groups	2121
Proteins	3620
Proteins (#Unique Peptides)	1554 (>2); 450 (=2); 1302 (=1);
FDR (Peptide-Spectrum Matches)	0.5%
FDR (Peptide Sequences)	1.0%
De Novo Only Spectra	21870

Deamidation	.98	NQ	5931	75.41	1.31E7	32.9
Oxidation	15.99	M	3317	74.19	7.88E7	1000.0
Acetylation	42.01	N-term	903	59.82		1000.0
Carbamidomethyl	57.02	C	155	62.44	5.2E6	1000.0

3. Experiment Control

Figure 4. Precursor mass error of peptide-spectrum matches (PSM) in filtered result. **(a)** Distribution of precursor mass error in ppm; **(b)** Scatterplot of precursor m/z versus precursor mass error in ppm.

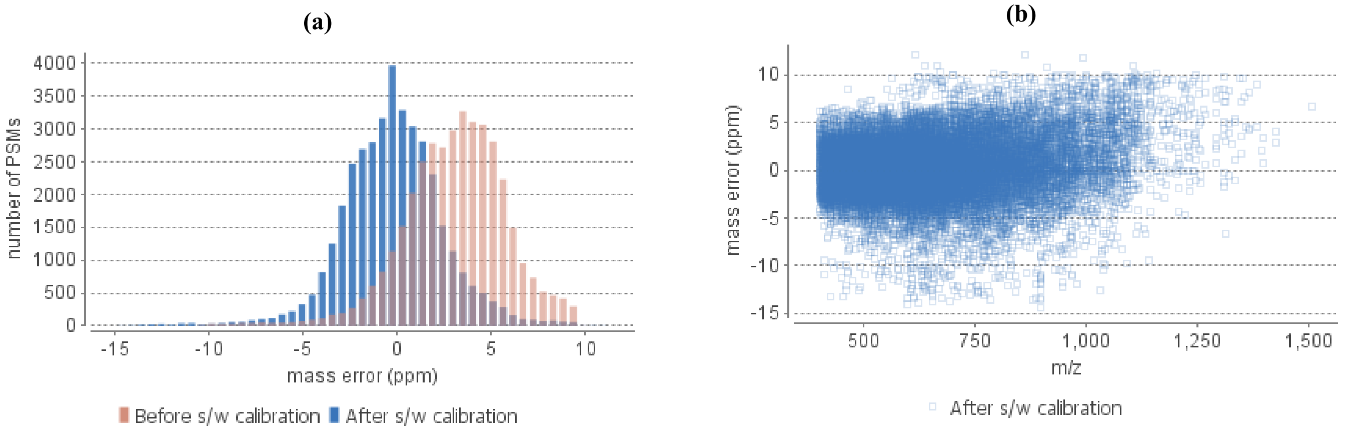

Table 5. Number of identified peptides in each sample by the number of missed cleavages

Missed Cleavages	0	1	2	3	4+
AZ001_01	3921	806	83	0	0
AZ001_02	3487	703	83	0	0
Sample 3	4341	771	77	0	0

4. Other Information

Table 6. Search parameters.

Search Engine Name: PEAKS
Parent Mass Error Tolerance: 10.0 ppm
Fragment Mass Error Tolerance: 0.05 Da
Precursor Mass Search Type: monoisotopic
Enzyme: Trypsin
Max Missed Cleavages: 2
Non-specific Cleavage: one
Fixed Modifications:

Table 7. Instrument parameters.

Fractions: AZ001_01.raw, AZ001_02.raw, AZ001_03.raw
Ion Source: ESI(nano-spray)
Fragmentation Mode: high energy CID (y and b ions)
MS Scan Mode: FT-ICR/Orbitrap
MS/MS Scan Mode: FT-ICR/Orbitrap

Carbamidomethylation: 57.02
Variable Modifications:
Deamidation (NQ): 0.98
Oxidation (M): 15.99
Acetylation (N-term): 42.01
Max Variable PTM Per Peptide: 2
Database: PF_all
Taxon: All
Searched Entry: 1941073
FDR Estimation: Enabled
Merge Options: no merge
Precursor Options: corrected
Charge Options: no correction
Filter Options: no filter
Process: true

1. Notes

2. Result Statistics

Figure 1. False discovery rate (FDR) curve. X axis is the number of peptide-spectrum matches (PSM) being kept. Y axis is the corresponding FDR.

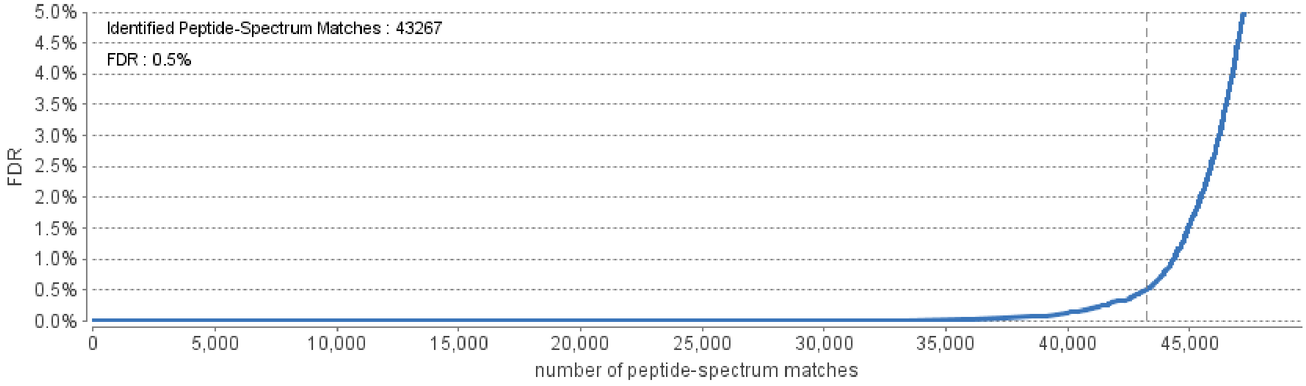

Figure 2. PSM score distribution. **(a)** Distribution of PEAKS peptide score; **(b)** Scatterplot of PEAKS peptide score versus precursor mass error.

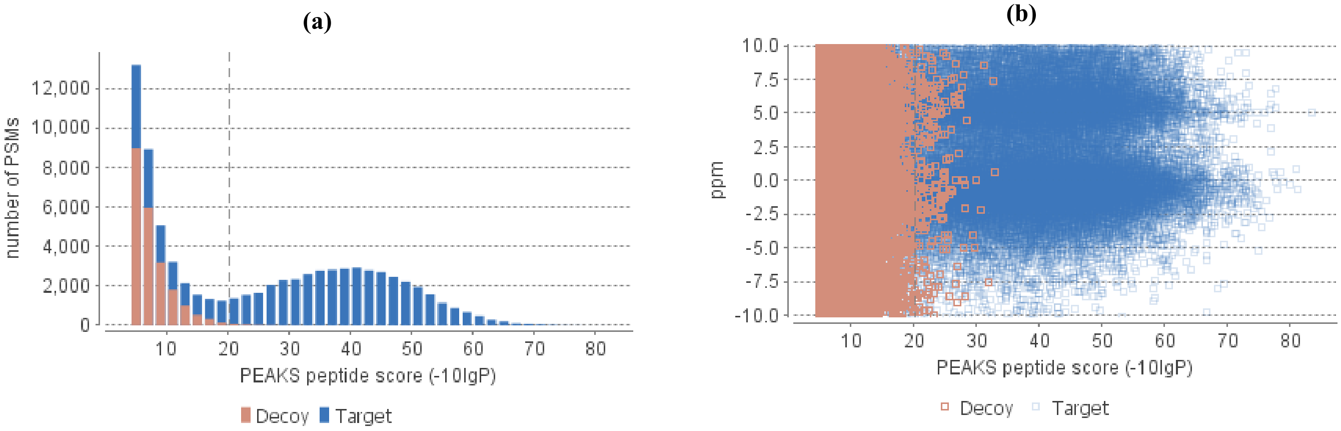

Figure 3. De novo result validation. Distribution of residue local confidence: **(a)** Residues in de novo sequences validated by confident database peptide assignment; **(b)** Residues in "de novo only" sequences.

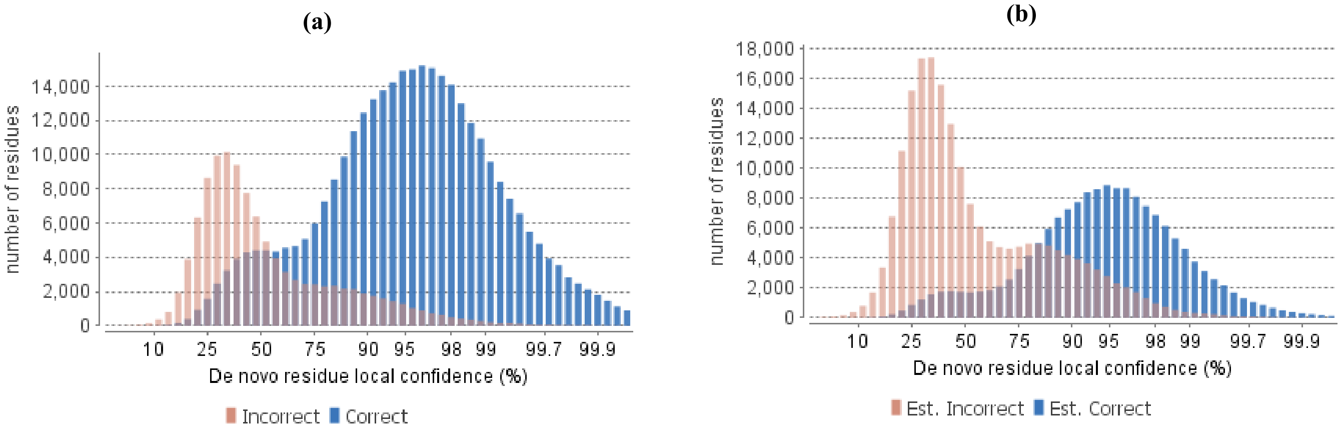

Table 1. Statistics of data.

# of MS scans	24585

Table 4. PTM profile.

Name	ΔMass	Position	#PSM	-10lgP	Area	AScore

# of MS/MS scans 138684

Table 2. Result filtration parameters.

Peptide -10lgP	≥20.2
Peptide Ascore	≥0
Protein -10lgP	≥20
Proteins unique peptides	≥0
De novo ALC Score	≥50%

Table 3. Statistics of filtered result.

Peptide-Spectrum Matches	43267
Peptide sequences	16343
Protein groups	2184
Proteins	3580
Proteins (#Unique Peptides)	1690 (>2); 461 (=2); 1166 (=1);
FDR (Peptide-Spectrum Matches)	0.5%
FDR (Peptide Sequences)	1.0%
De Novo Only Spectra	26364

Deamidation	.98	NQ	8984	83.39		31.3[illegible]
Oxidation	15.99	M	3442	75.91	2.08E8	1000.0[illegible]
Acetylation	42.01	N-term	1155	64.27	3.45E6	1000.0[illegible]
Carbamidomethyl	57.02	C	267	60.86	1.39E7	1000.0[illegible]

3. Experiment Control

Figure 4. Precursor mass error of peptide-spectrum matches (PSM) in filtered result. **(a)** Distribution of precursor mass error in ppm; **(b)** Scatterplot of precursor m/z versus precursor mass error in ppm.

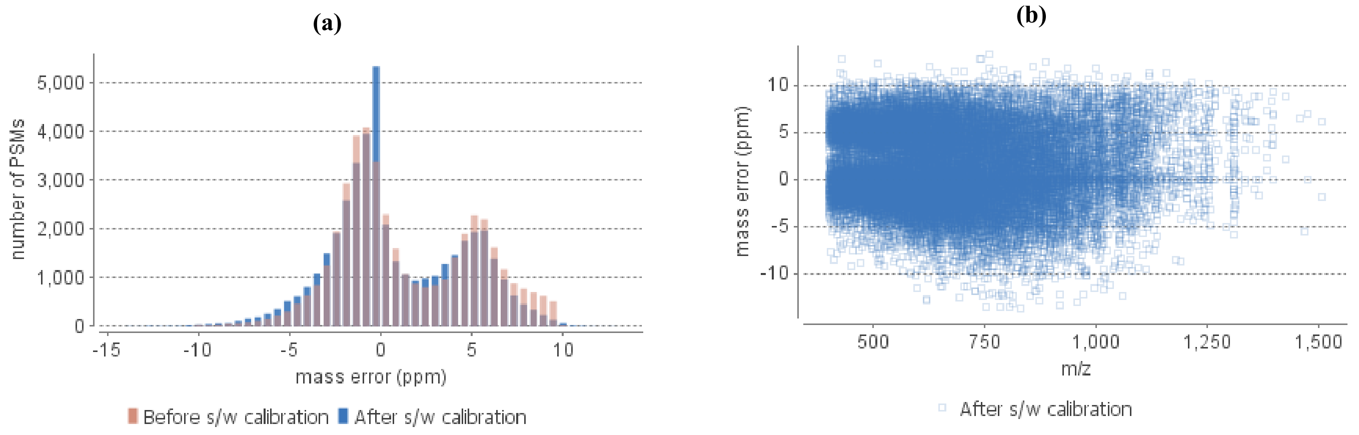

Table 5. Number of identified peptides in each sample by the number of missed cleavages

Missed Cleavages	0	1	2	3	4+
AZ002_01	5241	1048	126	0	0
AZ002_02	3999	748	78	0	0
AZ002_03	4263	735	105	0	0

4. Other Information

Table 6. Search parameters.

Search Engine Name: PEAKS
Parent Mass Error Tolerance: 10.0 ppm
Fragment Mass Error Tolerance: 0.05 Da
Precursor Mass Search Type: monoisotopic
Enzyme: Trypsin
Max Missed Cleavages: 2
Non-specific Cleavage: one
Fixed Modifications:

Table 7. Instrument parameters.

Fractions: AZ002_01.raw, AZ002_02.raw, AZ002_03.raw
Ion Source: ESI(nano-spray)
Fragmentation Mode: high energy CID (y and b ions)
MS Scan Mode: FT-ICR/Orbitrap
MS/MS Scan Mode: FT-ICR/Orbitrap

Carbamidomethylation: 57.02
Variable Modifications:
Deamidation (NQ): 0.98
Oxidation (M): 15.99
Acetylation (N-term): 42.01
Max Variable PTM Per Peptide: 2
Database: PF_all
Taxon: All
Searched Entry: 1941073
FDR Estimation: Enabled
Merge Options: no merge
Precursor Options: corrected
Charge Options: no correction
Filter Options: no filter
Process: true

1. Notes

2. Result Statistics

Figure 1. False discovery rate (FDR) curve. X axis is the number of peptide-spectrum matches (PSM) being kept. Y axis is the corresponding FDR.

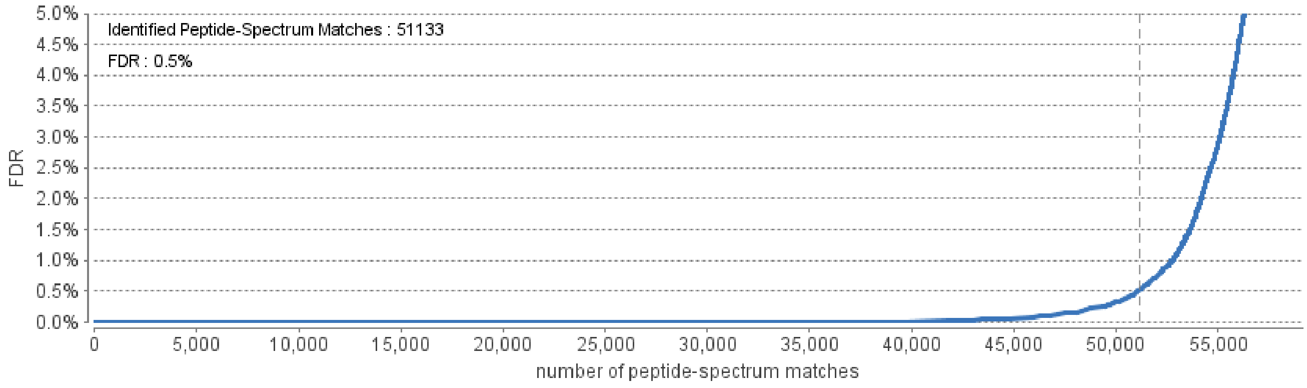

Figure 2. PSM score distribution. **(a)** Distribution of PEAKS peptide score; **(b)** Scatterplot of PEAKS peptide score versus precursor mass error.

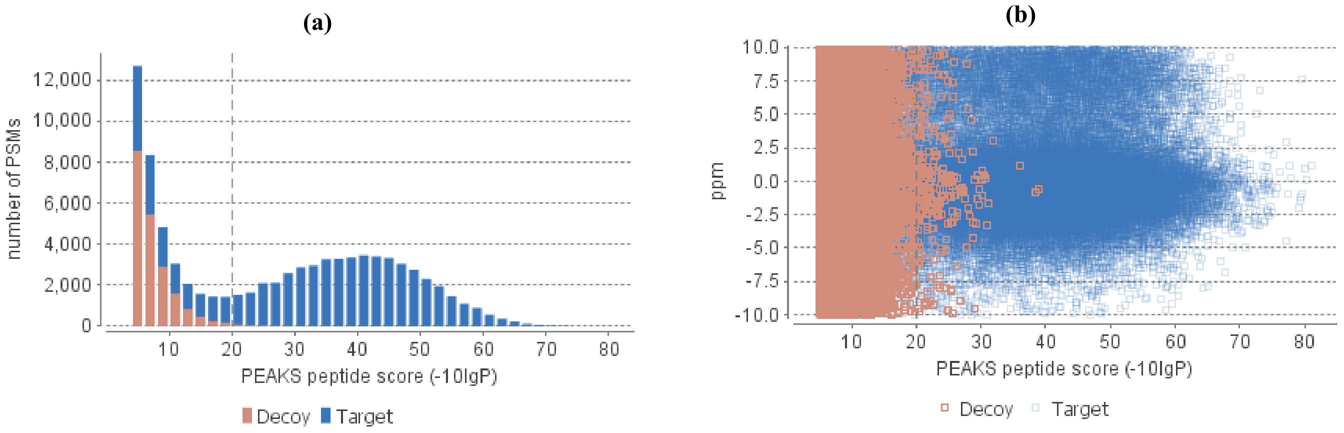

Figure 3. De novo result validation. Distribution of residue local confidence: **(a)** Residues in de novo sequences validated by confident database peptide assignment; **(b)** Residues in "de novo only" sequences.

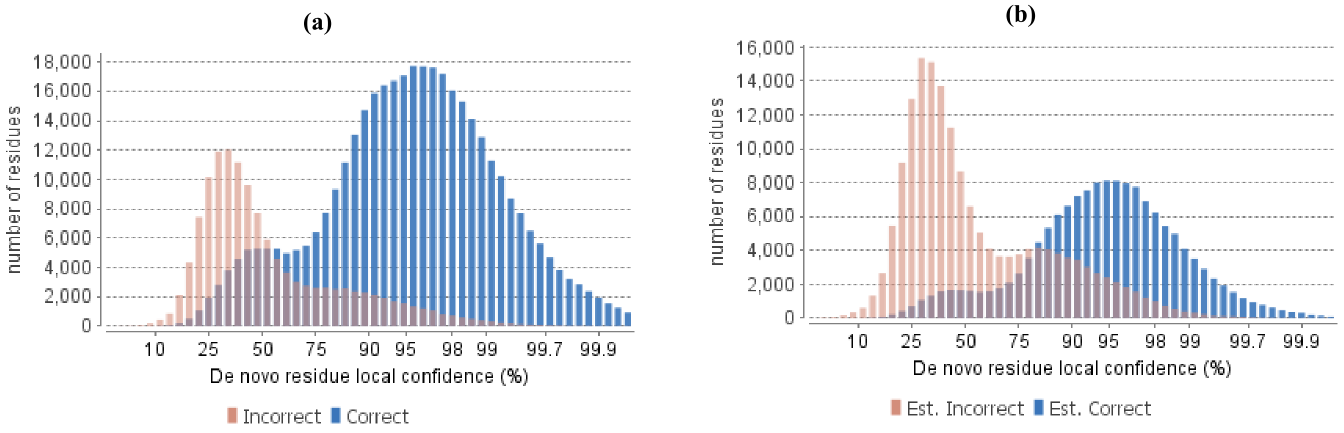

Table 1. Statistics of data.

# of MS scans	24102

Table 4. PTM profile.

Name	ΔMass	Position	#PSM	-10lgP	Area	AScor

# of MS/MS scans 140683

Table 2. Result filtration parameters.

Peptide -10lgP	≥20.1
Peptide Ascore	≥0
Protein -10lgP	≥20
Proteins unique peptides	≥0
De novo ALC Score	≥50%

Table 3. Statistics of filtered result.

Peptide-Spectrum Matches	51133
Peptide sequences	19974
Protein groups	2159
Proteins	3488
Proteins (#Unique Peptides)	1852 (>2); 333 (=2); 1067 (=1);
FDR (Peptide-Spectrum Matches)	0.5%
FDR (Peptide Sequences)	0.9%
De Novo Only Spectra	23265

Deamidation	.98	NQ	12103	81.02	7.47E7	18.0(
Oxidation	15.99	M	3878	74.64	1.68E8	1000.0(
Acetylation	42.01	N-term	1158	69.48	6.95E6	1000.0(
Carbamidomethyl	57.02	C	297	63.48		1000.0(

3. Experiment Control

Figure 4. Precursor mass error of peptide-spectrum matches (PSM) in filtered result. **(a)** Distribution of precursor mass error in ppm; **(b)** Scatterplot of precursor m/z versus precursor mass error in ppm.

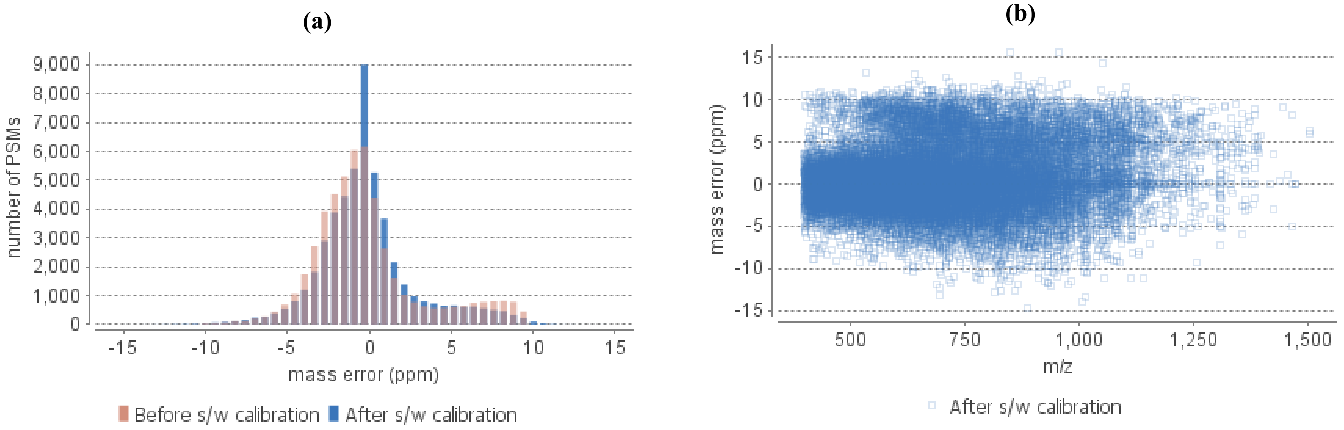

Table 5. Number of identified peptides in each sample by the number of missed cleavages

Missed Cleavages	0	1	2	3	4+
AZ004_01	5607	1139	125	0	0
AZ004_02	5899	1167	122	0	0
AZ004_03	4988	833	94	0	0

4. Other Information

Table 6. Search parameters.

Search Engine Name: PEAKS
Parent Mass Error Tolerance: 10.0 ppm
Fragment Mass Error Tolerance: 0.05 Da
Precursor Mass Search Type: monoisotopic
Enzyme: Trypsin
Max Missed Cleavages: 2
Non-specific Cleavage: one
Fixed Modifications:

Table 7. Instrument parameters.

Fractions: AZ004_01.raw, AZ004_02.raw, AZ004_03.raw
Ion Source: ESI(nano-spray)
Fragmentation Mode: high energy CID (y and b ions)
MS Scan Mode: FT-ICR/Orbitrap
MS/MS Scan Mode: FT-ICR/Orbitrap

Carbamidomethylation: 57.02
Variable Modifications:
Deamidation (NQ): 0.98
Oxidation (M): 15.99
Acetylation (N-term): 42.01
Max Variable PTM Per Peptide: 2
Database: PF_all
Taxon: All
Searched Entry: 1941073
FDR Estimation: Enabled
Merge Options: no merge
Precursor Options: corrected
Charge Options: no correction
Filter Options: no filter
Process: true

1. Notes

2. Result Statistics

Figure 1. False discovery rate (FDR) curve. X axis is the number of peptide-spectrum matches (PSM) being kept. Y axis is the corresponding FDR.

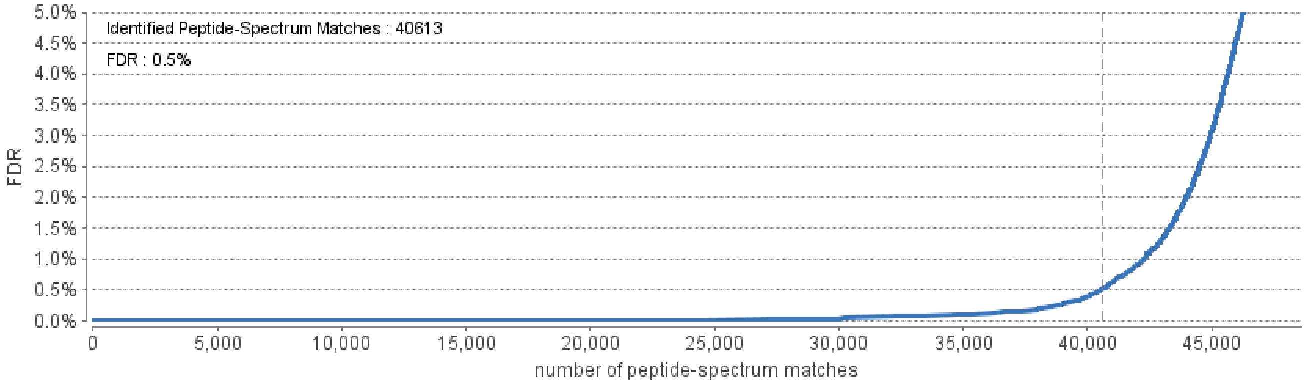

Figure 2. PSM score distribution. **(a)** Distribution of PEAKS peptide score; **(b)** Scatterplot of PEAKS peptide score versus precursor mass error.

(a)

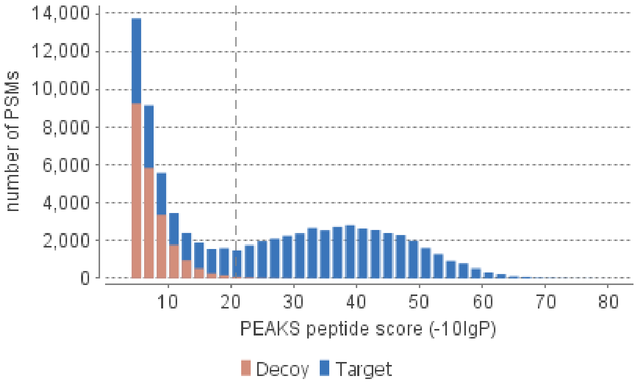

(b)

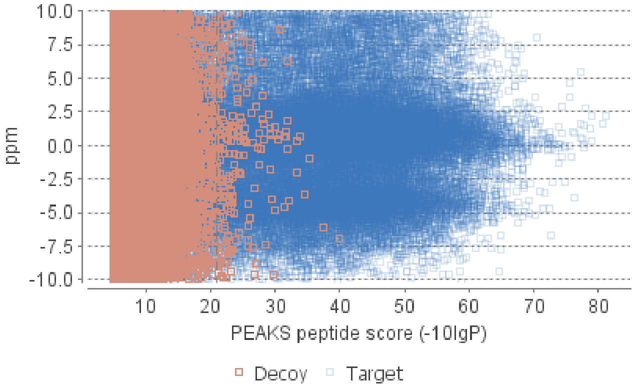

Figure 3. De novo result validation. Distribution of residue local confidence: **(a)** Residues in de novo sequences validated by confident database peptide assignment; **(b)** Residues in "de novo only" sequences.

(a)

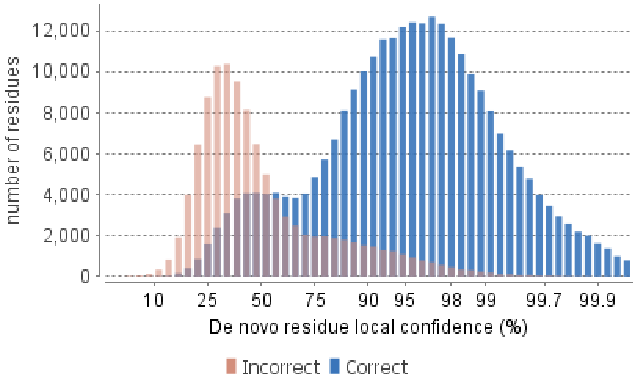

(b)

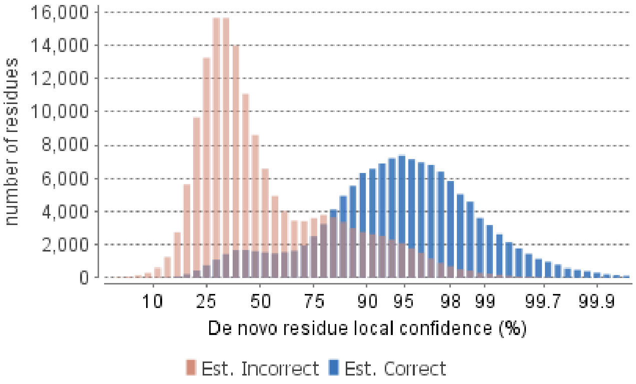

Table 1. Statistics of data.

# of MS scans	24814

Table 4. PTM profile.

Name	ΔMass	Position	#PSM	-10lgP	Area	AScor[illegible]

# of MS/MS scans 138392

Deamidation	.98	NQ	8308	79.07	5.18E7	32.9[illegible]
Oxidation	15.99	M	3822	77.17		61.5[illegible]
Acetylation	42.01	N-term	1161	69.82	5.81E6	1000.0[illegible]
Carbamidomethyl	57.02	C	204	66.53		1000.0[illegible]

Table 2. Result filtration parameters.

Peptide -10lgP	≥20.9
Peptide Ascore	≥0
Protein -10lgP	≥20
Proteins unique peptides	≥0
De novo ALC Score	≥50%

Table 3. Statistics of filtered result.

Peptide-Spectrum Matches	40613
Peptide sequences	15048
Protein groups	2128
Proteins	3625
Proteins (#Unique Peptides)	1652 (>2); 426 (=2); 1198 (=1);
FDR (Peptide-Spectrum Matches)	0.5%
FDR (Peptide Sequences)	1.1%
De Novo Only Spectra	22771

3. Experiment Control

Figure 4. Precursor mass error of peptide-spectrum matches (PSM) in filtered result. **(a)** Distribution of precursor mass error in ppm; **(b)** Scatterplot of precursor m/z versus precursor mass error in ppm.

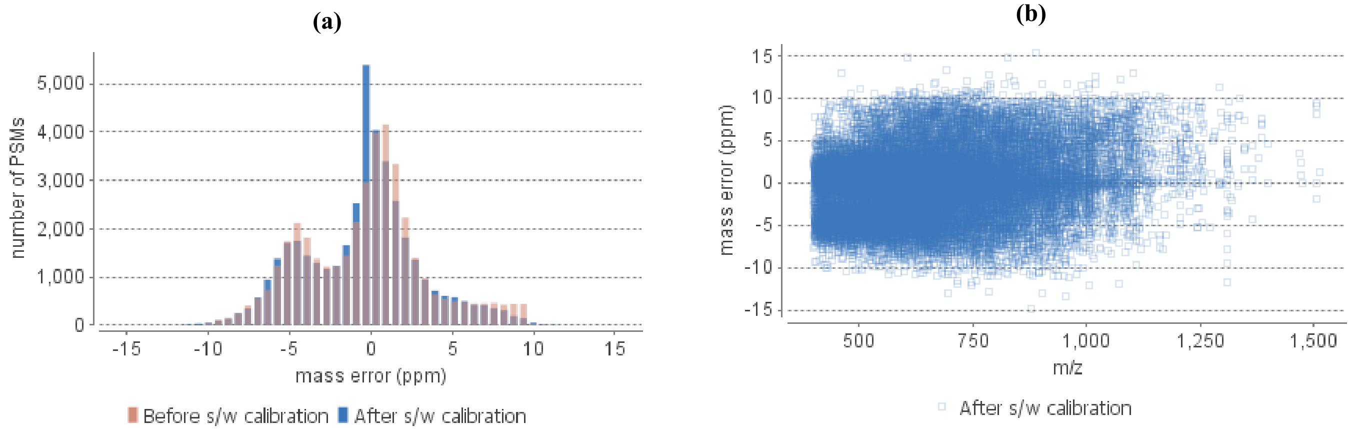

Table 5. Number of identified peptides in each sample by the number of missed cleavages

Missed Cleavages	0	1	2	3	4+
AZ006_01	5086	708	61	0	0
AZ006_02	4102	701	55	0	0
AZ006_03	3686	597	52	0	0

4. Other Information

Table 6. Search parameters.

Search Engine Name: PEAKS
Parent Mass Error Tolerance: 10.0 ppm
Fragment Mass Error Tolerance: 0.05 Da
Precursor Mass Search Type: monoisotopic
Enzyme: Trypsin
Max Missed Cleavages: 2
Non-specific Cleavage: one
Fixed Modifications:

Table 7. Instrument parameters.

Fractions: AZ006_01.raw, AZ006_02.raw, AZ006_03.raw
Ion Source: ESI(nano-spray)
Fragmentation Mode: high energy CID (y and b ions)
MS Scan Mode: FT-ICR/Orbitrap
MS/MS Scan Mode: FT-ICR/Orbitrap

Carbamidomethylation: 57.02
Variable Modifications:
Deamidation (NQ): 0.98
Oxidation (M): 15.99
Acetylation (N-term): 42.01
Max Variable PTM Per Peptide: 2
Database: PF_all
Taxon: All
Searched Entry: 1941073
FDR Estimation: Enabled
Merge Options: no merge
Precursor Options: corrected
Charge Options: no correction
Filter Options: no filter
Process: true

1. Notes

2. Result Statistics

Figure 1. False discovery rate (FDR) curve. X axis is the number of peptide-spectrum matches (PSM) being kept. Y axis is the corresponding FDR.

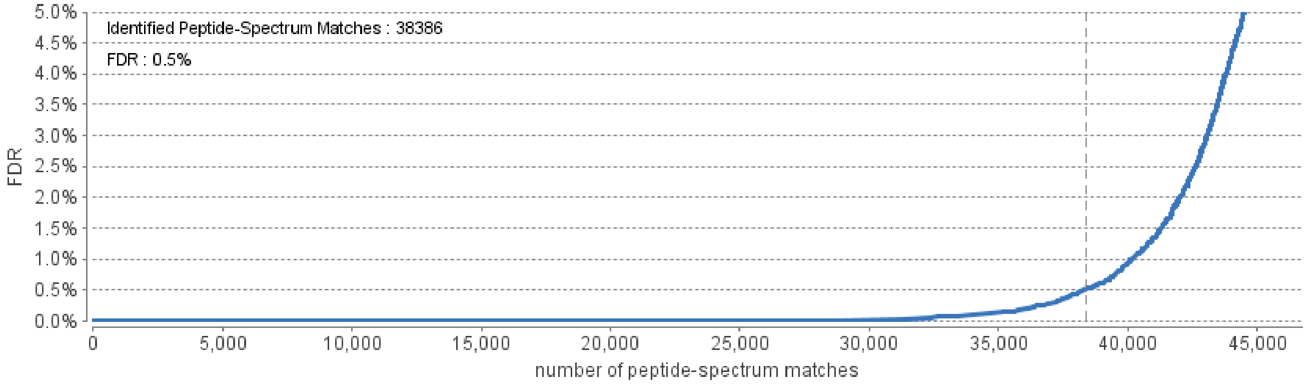

Figure 2. PSM score distribution. **(a)** Distribution of PEAKS peptide score; **(b)** Scatterplot of PEAKS peptide score versus precursor mass error.

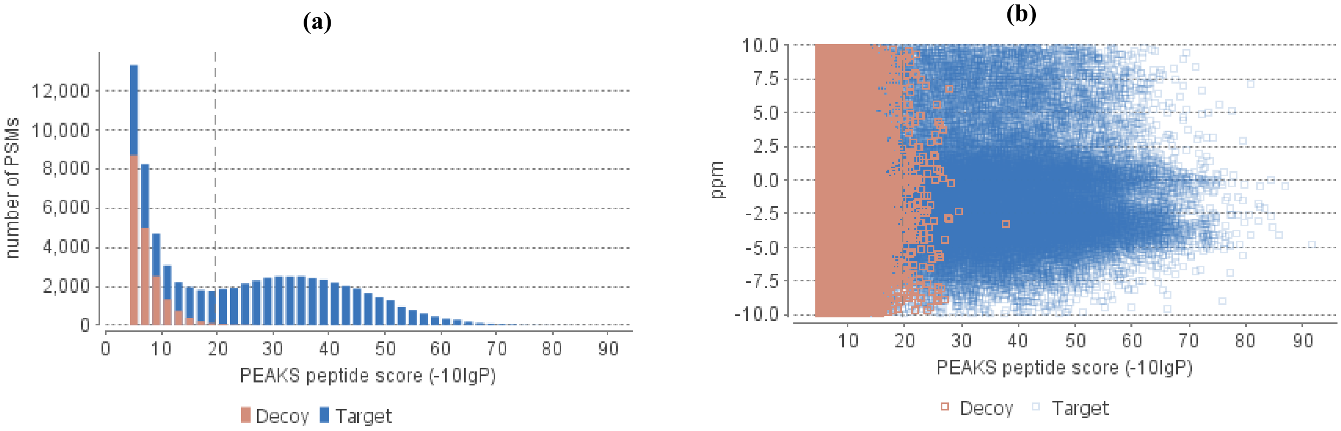

Figure 3. De novo result validation. Distribution of residue local confidence: **(a)** Residues in de novo sequences validated by confident database peptide assignment; **(b)** Residues in "de novo only" sequences.

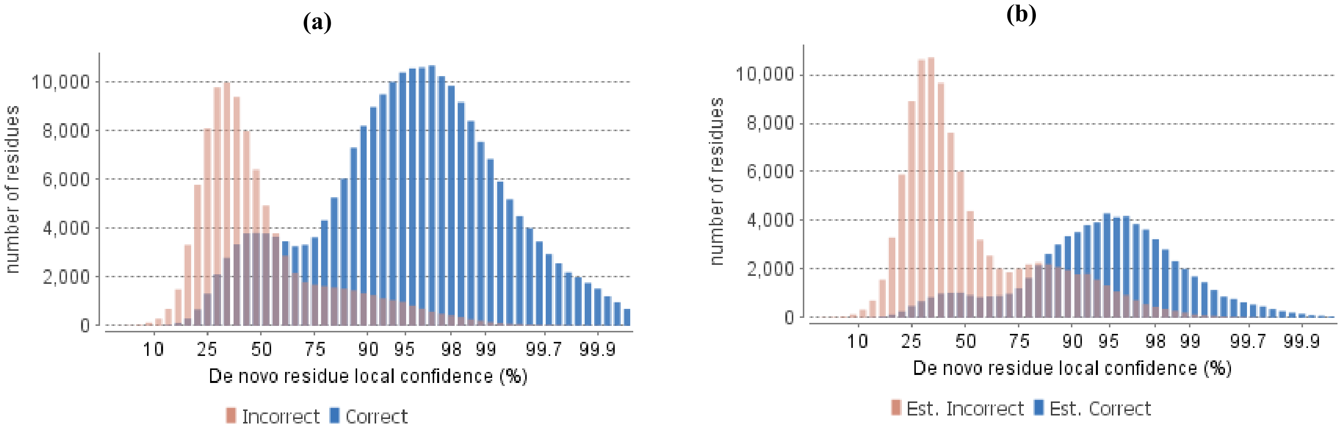

Table 1. Statistics of data.

# of MS scans	27074

Table 4. PTM profile.

Name	ΔMass	Position	#PSM	-10lgP	Area	AScore

# of MS/MS scans 129639

Deamidation	.98	NQ	8129	91.57		129.8[illegible]
Oxidation	15.99	M	3800	80.85	1.11E8	1000.0[illegible]
Acetylation	42.01	N-term	720	61.01		1000.0[illegible]
Carbamidomethyl	57.02	C	97	67.35	3.47E6	1000.0[illegible]

Table 2. Result filtration parameters.

Peptide -10lgP	≥19.7
Peptide Ascore	≥0
Protein -10lgP	≥20
Proteins unique peptides	≥0
De novo ALC Score	≥50%

Table 3. Statistics of filtered result.

Peptide-Spectrum Matches	38386
Peptide sequences	14049
Protein groups	1862
Proteins	3313
Proteins (#Unique Peptides)	1499 (>2); 367 (=2); 1128 (=1);
FDR (Peptide-Spectrum Matches)	0.5%
FDR (Peptide Sequences)	1.1%
De Novo Only Spectra	14985

3. Experiment Control

Figure 4. Precursor mass error of peptide-spectrum matches (PSM) in filtered result. **(a)** Distribution of precursor mass error in ppm; **(b)** Scatterplot of precursor m/z versus precursor mass error in ppm.

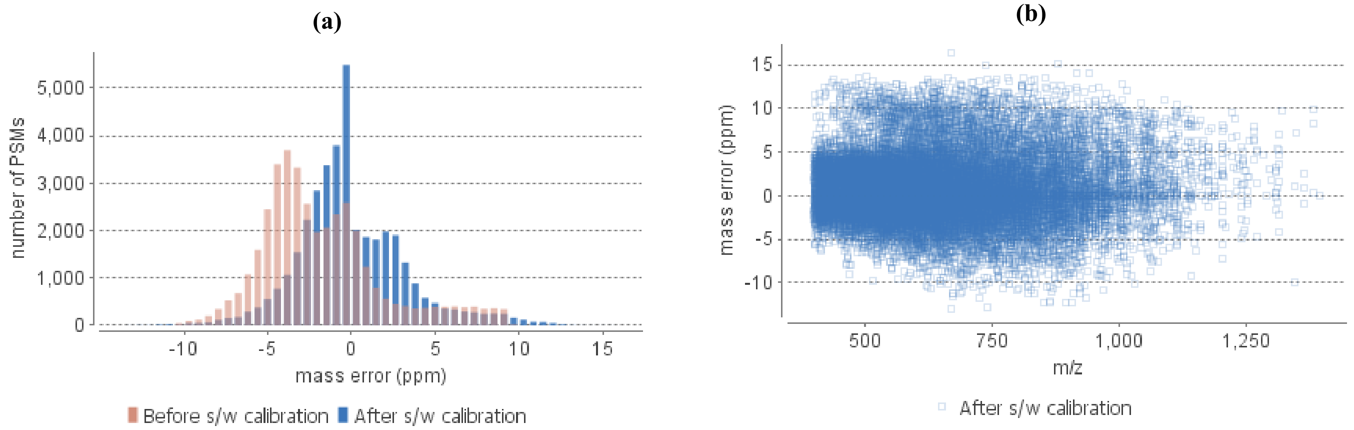

Table 5. Number of identified peptides in each sample by the number of missed cleavages

Missed Cleavages	0	1	2	3	4+
AZ007_01	3470	556	44	0	0
AZ007_02	3896	575	47	0	0
AZ007_03	4735	679	47	0	0

4. Other Information

Table 6. Search parameters.

Search Engine Name: PEAKS
Parent Mass Error Tolerance: 10.0 ppm
Fragment Mass Error Tolerance: 0.05 Da
Precursor Mass Search Type: monoisotopic
Enzyme: Trypsin
Max Missed Cleavages: 2
Non-specific Cleavage: one
Fixed Modifications:

Table 7. Instrument parameters.

Fractions: AZ007_01.raw, AZ007_02.raw, AZ007_03.raw
Ion Source: ESI(nano-spray)
Fragmentation Mode: high energy CID (y and b ions)
MS Scan Mode: FT-ICR/Orbitrap
MS/MS Scan Mode: FT-ICR/Orbitrap

Carbamidomethylation: 57.02
Variable Modifications:
Deamidation (NQ): 0.98
Acetylation (N-term): 42.01
Oxidation (M): 15.99
Max Variable PTM Per Peptide: 3
Database: PF_all
Taxon: All
Searched Entry: 1941073
FDR Estimation: Enabled
Merge Options: no merge
Precursor Options: corrected
Charge Options: no correction
Filter Options: no filter
Process: true

1. Notes

2. Result Statistics

Figure 1. False discovery rate (FDR) curve. X axis is the number of peptide-spectrum matches (PSM) being kept. Y axis is the corresponding FDR.

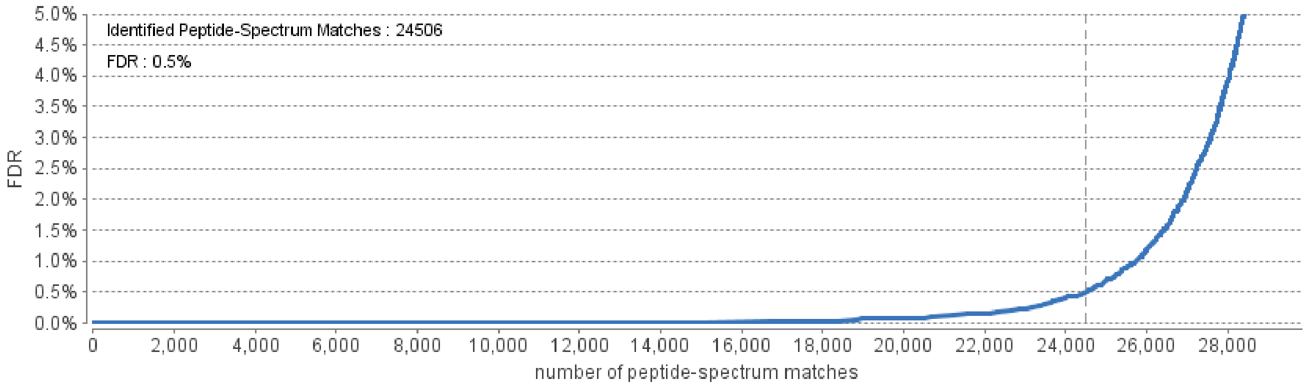

Figure 2. PSM score distribution. **(a)** Distribution of PEAKS peptide score; **(b)** Scatterplot of PEAKS peptide score versus precursor mass error.

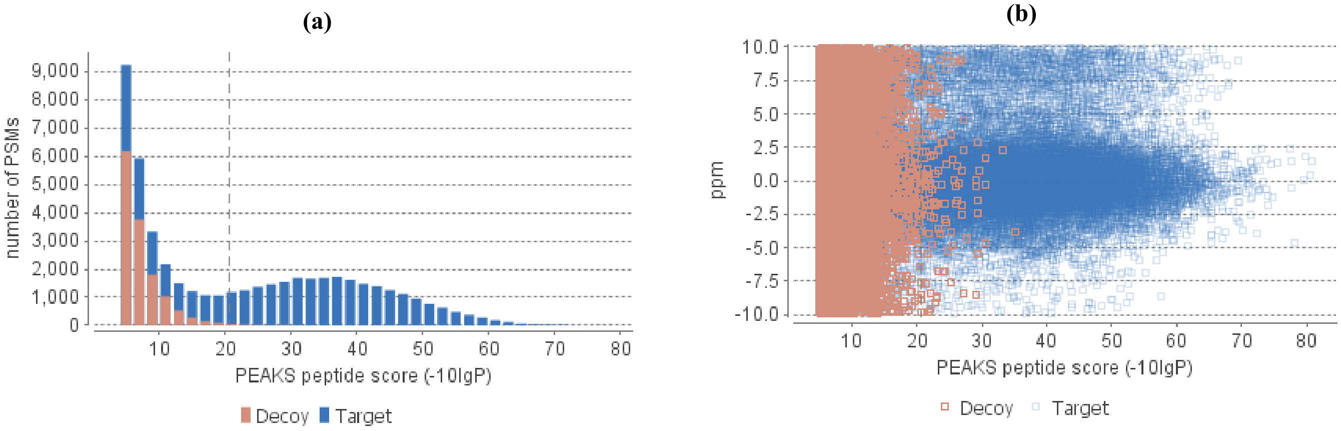

Figure 3. De novo result validation. Distribution of residue local confidence: **(a)** Residues in de novo sequences validated by confident database peptide assignment; **(b)** Residues in "de novo only" sequences.

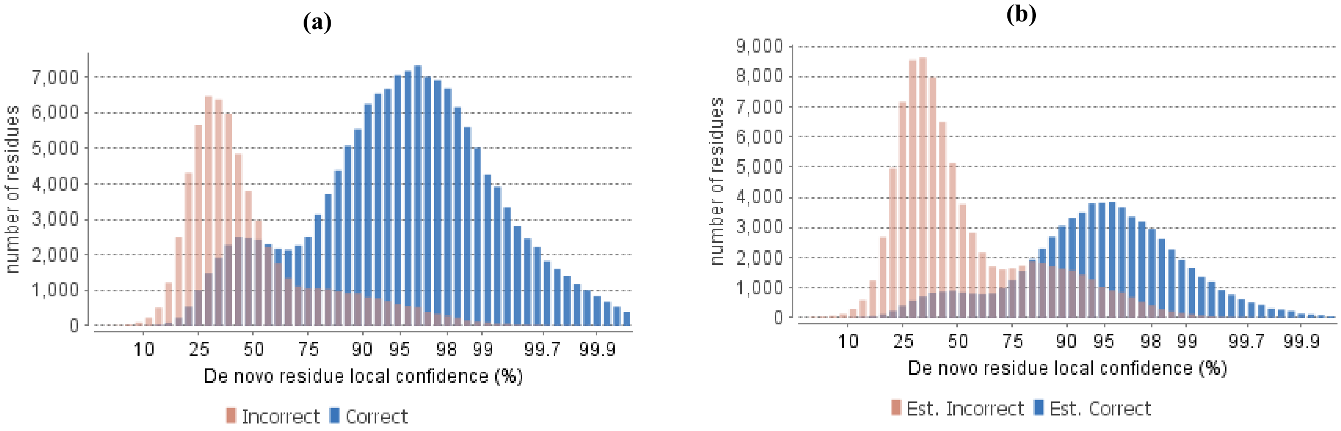

Table 1. Statistics of data.

# of MS scans	17573

Table 4. PTM profile.

Name	ΔMass	Position	#PSM	-10lgP	Area	AScore

# of MS/MS scans 88053

Deamidation	.98	NQ	4659	80.74	2.08E7	32.9[illegible]
Oxidation	15.99	M	2291	80.52	1.35E8	1000.0[illegible]
Acetylation	42.01	N-term	572	63.01	1.85E6	1000.0[illegible]
Carbamidomethyl	57.02	C	79	65.87	1.22E6	1000.0[illegible]

Table 2. Result filtration parameters.

Peptide -10lgP	≥20.6
Peptide Ascore	≥0
Protein -10lgP	≥20
Proteins unique peptides	≥0
De novo ALC Score	≥50%

Table 3. Statistics of filtered result.

Peptide-Spectrum Matches	24506
Peptide sequences	12518
Protein groups	1841
Proteins	3405
Proteins (#Unique Peptides)	1479 (>2); 481 (=2); 1172 (=1);
FDR (Peptide-Spectrum Matches)	0.5%
FDR (Peptide Sequences)	0.8%
De Novo Only Spectra	12921

3. Experiment Control

Figure 4. Precursor mass error of peptide-spectrum matches (PSM) in filtered result. **(a)** Distribution of precursor mass error in ppm; **(b)** Scatterplot of precursor m/z versus precursor mass error in ppm.

(a)

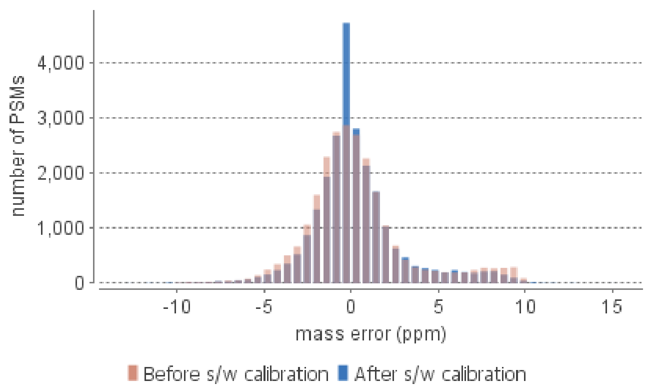

(b)

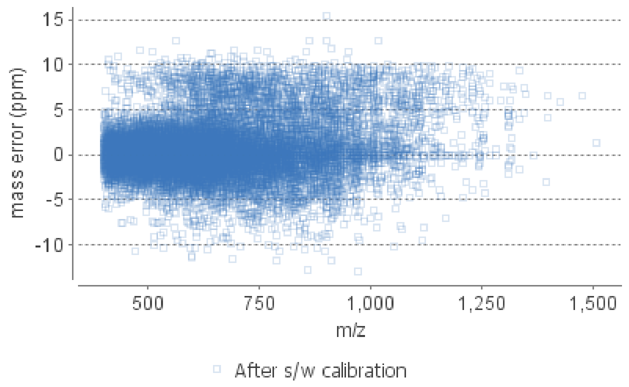

Table 5. Number of identified peptides in each sample by the number of missed cleavages

Missed Cleavages	0	1	2	3	4+
AZ011_01	5140	669	46	0	0
AZ011_02	5759	843	61	0	0

4. Other Information

Table 6. Search parameters.

Search Engine Name: PEAKS
Parent Mass Error Tolerance: 10.0 ppm
Fragment Mass Error Tolerance: 0.05 Da
Precursor Mass Search Type: monoisotopic
Enzyme: Trypsin
Max Missed Cleavages: 2
Non-specific Cleavage: one
Fixed Modifications:
  Carbamidomethylation: 57.02
Variable Modifications:

Table 7. Instrument parameters.

Fractions: AZ011_01.raw, AZ011_02.raw, AZ011_03.raw
Ion Source: ESI(nano-spray)
Fragmentation Mode: high energy CID (y and b ions)
MS Scan Mode: FT-ICR/Orbitrap
MS/MS Scan Mode: FT-ICR/Orbitrap

Deamidation (NQ): 0.98
Acetylation (N-term): 42.01
Oxidation (M): 15.99
Max Variable PTM Per Peptide: 3
Database: PF_all
Taxon: All
Searched Entry: 1941073
FDR Estimation: Enabled
Merge Options: no merge
Precursor Options: corrected
Charge Options: no correction
Filter Options: no filter
Process: true

1. Notes

2. Result Statistics

Figure 1. False discovery rate (FDR) curve. X axis is the number of peptide-spectrum matches (PSM) being kept. Y axis is the corresponding FDR.

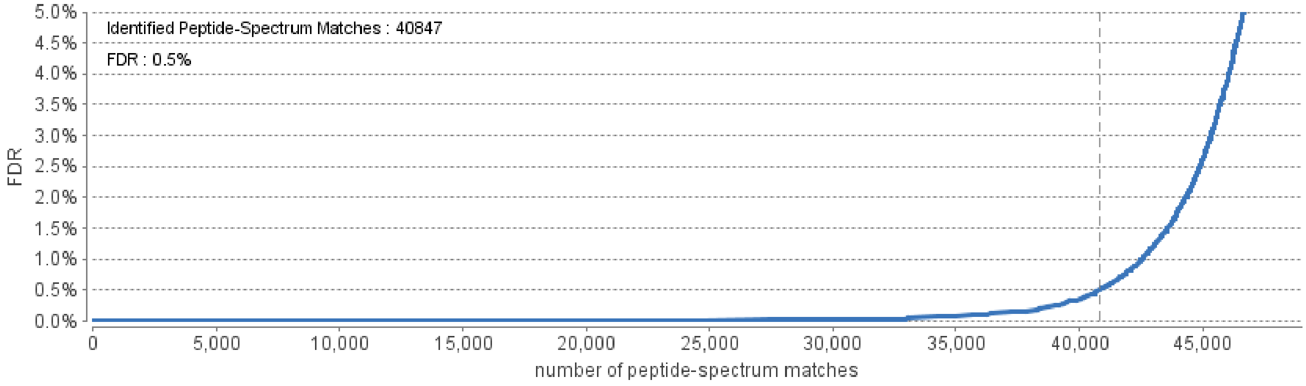

Figure 2. PSM score distribution. **(a)** Distribution of PEAKS peptide score; **(b)** Scatterplot of PEAKS peptide score versus precursor mass error.

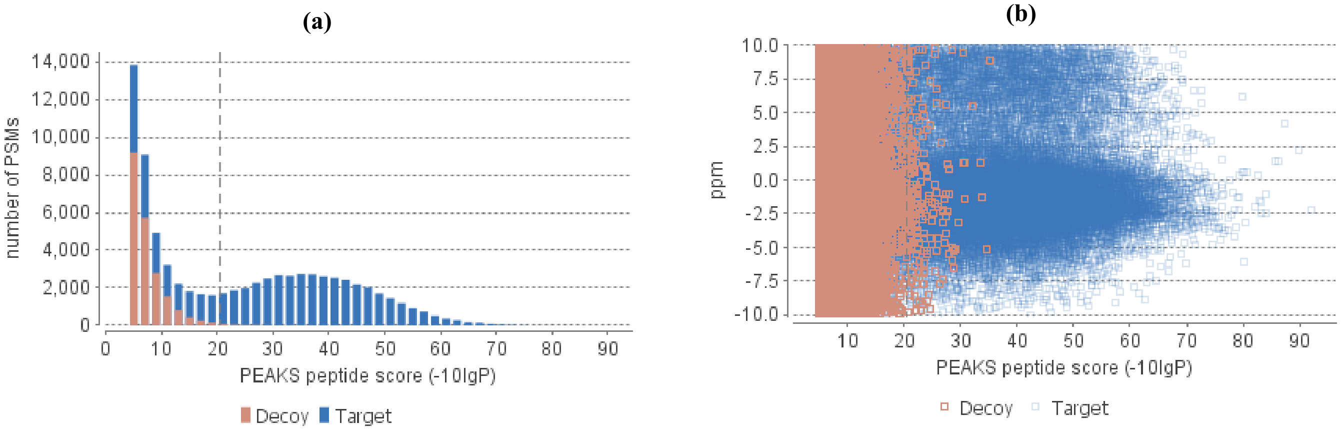

Figure 3. De novo result validation. Distribution of residue local confidence: **(a)** Residues in de novo sequences validated by confident database peptide assignment; **(b)** Residues in "de novo only" sequences.

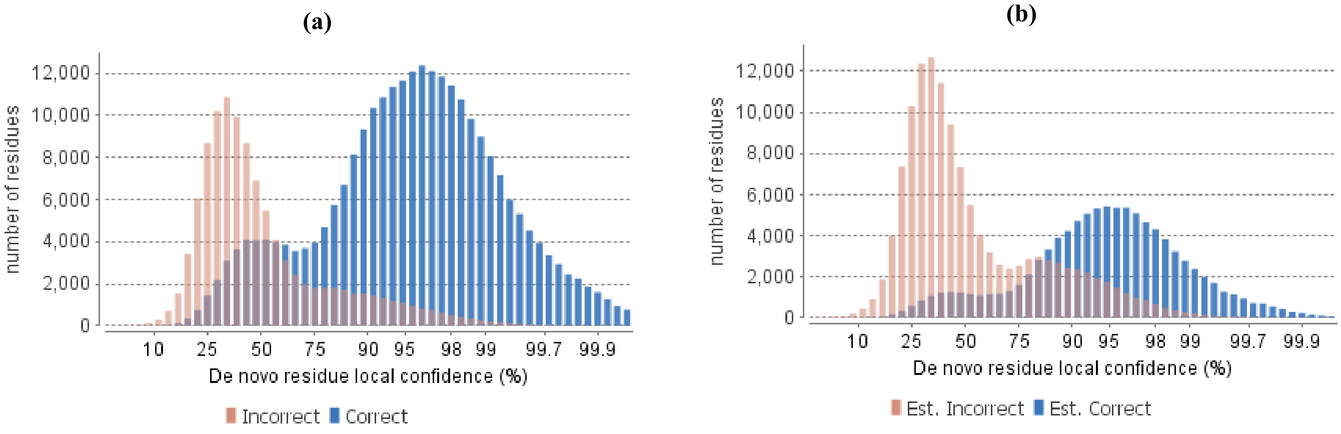

Table 1. Statistics of data.

# of MS scans	25431

Table 4. PTM profile.

Name	ΔMass	Position	#PSM	-10lgP	Area	AScore

# of MS/MS scans 135634

Deamidation	.98	NQ	9312	91.97	4.68E6	47.0[illegible]
Oxidation	15.99	M	4045	91.97	4.68E6	1000.0[illegible]
Acetylation	42.01	N-term	911	65.52	4.19E6	1000.0[illegible]
Carbamidomethyl	57.02	C	178	63.83		1000.0[illegible]

Table 2. Result filtration parameters.

Peptide -10lgP	≥20.4
Peptide Ascore	≥0
Protein -10lgP	≥20
Proteins unique peptides	≥0
De novo ALC Score	≥50%

Table 3. Statistics of filtered result.

Peptide-Spectrum Matches	40847
Peptide sequences	15713
Protein groups	1981
Proteins	3465
Proteins (#Unique Peptides)	1568 (>2); 427 (=2); 1138 (=1);
FDR (Peptide-Spectrum Matches)	0.5%
FDR (Peptide Sequences)	1.0%
De Novo Only Spectra	18457

3. Experiment Control

Figure 4. Precursor mass error of peptide-spectrum matches (PSM) in filtered result. **(a)** Distribution of precursor mass error in ppm; **(b)** Scatterplot of precursor m/z versus precursor mass error in ppm.

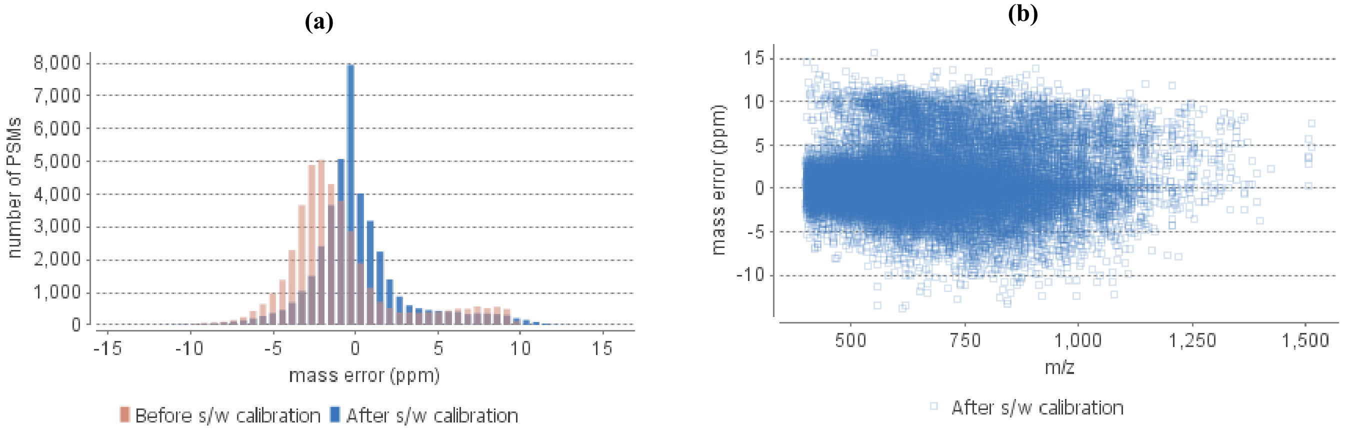

Table 5. Number of identified peptides in each sample by the number of missed cleavages

Missed Cleavages	0	1	2	3	4+
AZ013_01	4394	678	66	0	0
AZ013_02	4834	661	48	0	0
AZ013_03	4352	628	52	0	0

4. Other Information

Table 6. Search parameters.

Search Engine Name: PEAKS
Parent Mass Error Tolerance: 10.0 ppm
Fragment Mass Error Tolerance: 0.05 Da
Precursor Mass Search Type: monoisotopic
Enzyme: Trypsin
Max Missed Cleavages: 2
Non-specific Cleavage: one
Fixed Modifications:

Table 7. Instrument parameters.

Fractions: AZ013_01.raw, AZ013_02.raw, AZ013_03.raw
Ion Source: ESI(nano-spray)
Fragmentation Mode: high energy CID (y and b ions)
MS Scan Mode: FT-ICR/Orbitrap
MS/MS Scan Mode: FT-ICR/Orbitrap

Carbamidomethylation: 57.02
Variable Modifications:
Deamidation (NQ): 0.98
Acetylation (N-term): 42.01
Oxidation (M): 15.99
Max Variable PTM Per Peptide: 3
Database: PF_all
Taxon: All
Searched Entry: 1941073
FDR Estimation: Enabled
Merge Options: no merge
Precursor Options: corrected
Charge Options: no correction
Filter Options: no filter
Process: true

1. Notes

2. Result Statistics

Figure 1. False discovery rate (FDR) curve. X axis is the number of peptide-spectrum matches (PSM) being kept. Y axis is the corresponding FDR.

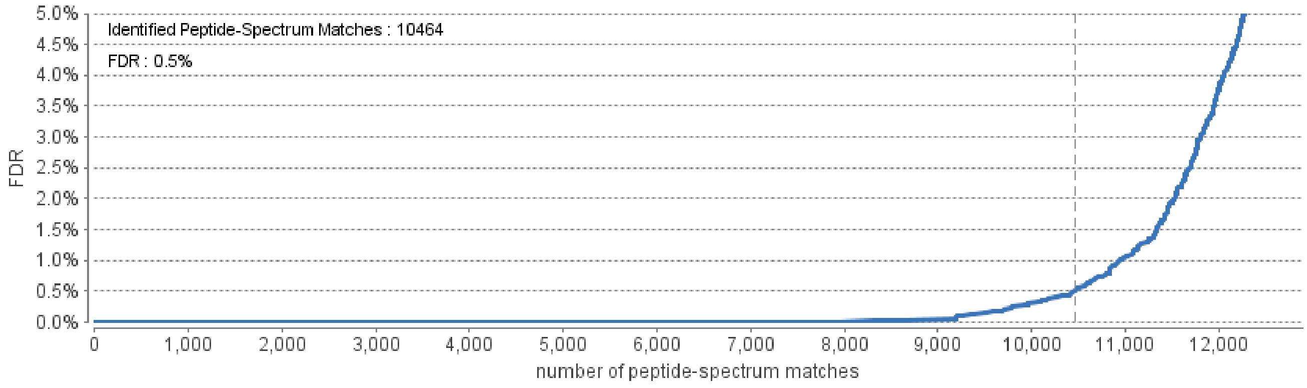

Figure 2. PSM score distribution. **(a)** Distribution of PEAKS peptide score; **(b)** Scatterplot of PEAKS peptide score versus precursor mass error.

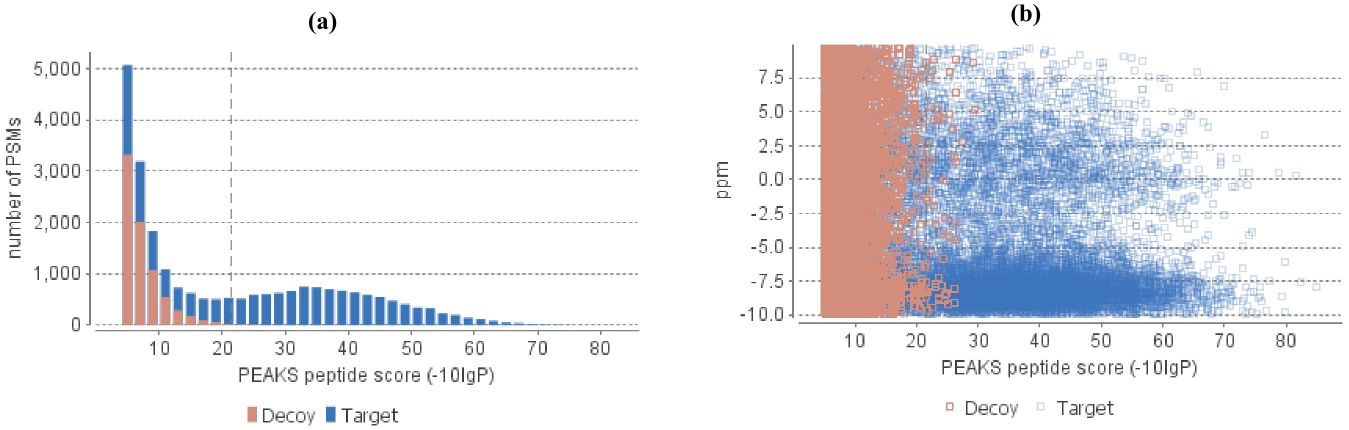

Figure 3. De novo result validation. Distribution of residue local confidence: **(a)** Residues in de novo sequences validated by confident database peptide assignment; **(b)** Residues in "de novo only" sequences.

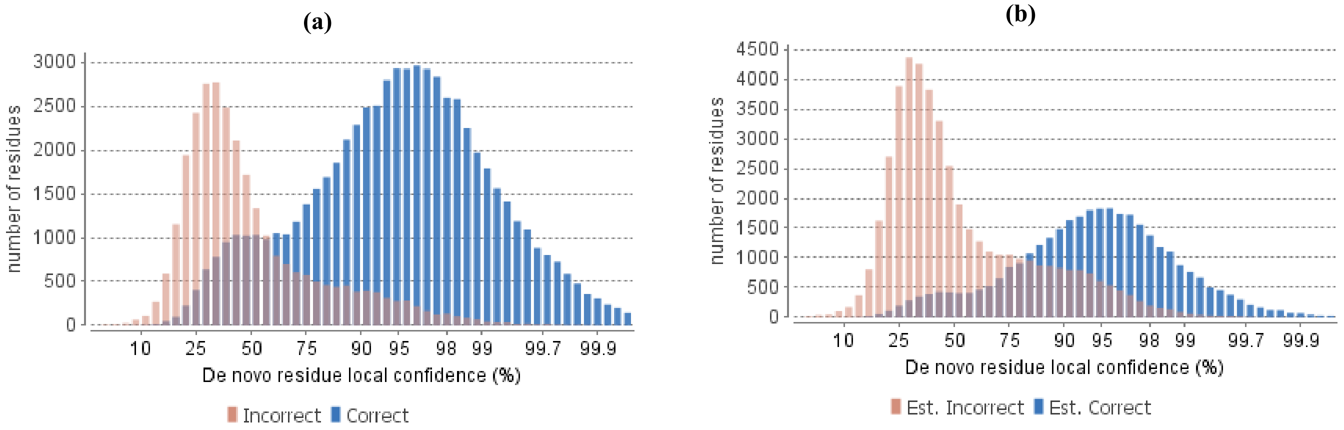

Table 1. Statistics of data.

# of MS scans	8493

Table 4. PTM profile.

Name	ΔMass	Position	#PSM	-10lgP	Area	AScor[illegible]

# of MS/MS scans 44798

Deamidation	.98	NQ	2855	79.77		0.0(
Oxidation	15.99	M	900	75.78	3.23E6	1000.0(
Acetylation	42.01	N-term	218	65.47		1000.0(
Carbamidomethyl	57.02	C	43	65.93	4.57E6	1000.0(

Table 2. Result filtration parameters.

Peptide -10lgP	≥21.5
Peptide Ascore	≥0
Protein -10lgP	≥20
Proteins unique peptides	≥0
De novo ALC Score	≥50%

Table 3. Statistics of filtered result.

Peptide-Spectrum Matches	10464
Peptide sequences	7703
Protein groups	1408
Proteins	3026
Proteins (#Unique Peptides)	1046 (>2); 433 (=2); 1354 (=1);
FDR (Peptide-Spectrum Matches)	0.5%
FDR (Peptide Sequences)	0.6%
De Novo Only Spectra	6706

3. Experiment Control

Figure 4. Precursor mass error of peptide-spectrum matches (PSM) in filtered result. **(a)** Distribution of precursor mass error in ppm; **(b)** Scatterplot of precursor m/z versus precursor mass error in ppm.

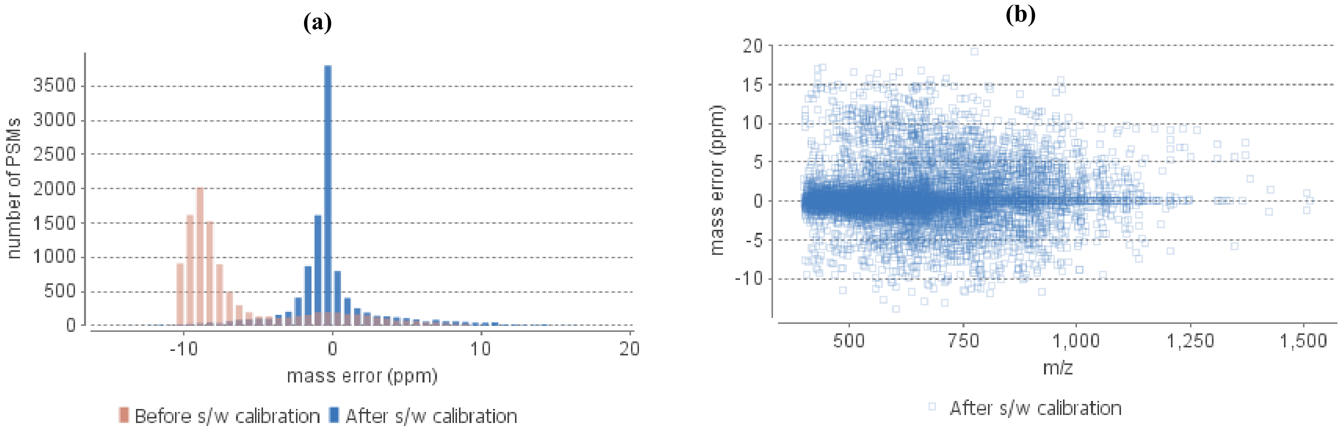

Table 5. Number of identified peptides in each sample by the number of missed cleavages

Missed Cleavages	0	1	2	3	4+
AZ014_02	6676	952	75	0	0

4. Other Information

Table 6. Search parameters.
Search Engine Name: PEAKS
Parent Mass Error Tolerance: 10.0 ppm
Fragment Mass Error Tolerance: 0.05 Da
Precursor Mass Search Type: monoisotopic
Enzyme: Trypsin
Max Missed Cleavages: 2
Non-specific Cleavage: one
Fixed Modifications:
  Carbamidomethylation: 57.02
Variable Modifications:
  Deamidation (NQ): 0.98

Table 7. Instrument parameters.
Fractions: AZ014_01.raw, AZ014_02.raw, AZ014_03.raw
Ion Source: ESI(nano-spray)
Fragmentation Mode: high energy CID (y and b ions)
MS Scan Mode: FT-ICR/Orbitrap
MS/MS Scan Mode: FT-ICR/Orbitrap

Acetylation (N-term): 42.01
Oxidation (M): 15.99
Max Variable PTM Per Peptide: 3
Database: PF_all
Taxon: All
Searched Entry: 1941073
FDR Estimation: Enabled
Merge Options: no merge
Precursor Options: corrected
Charge Options: no correction
Filter Options: no filter
Process: true

1. Notes

2. Result Statistics

Figure 1. False discovery rate (FDR) curve. X axis is the number of peptide-spectrum matches (PSM) being kept. Y axis is the corresponding FDR.

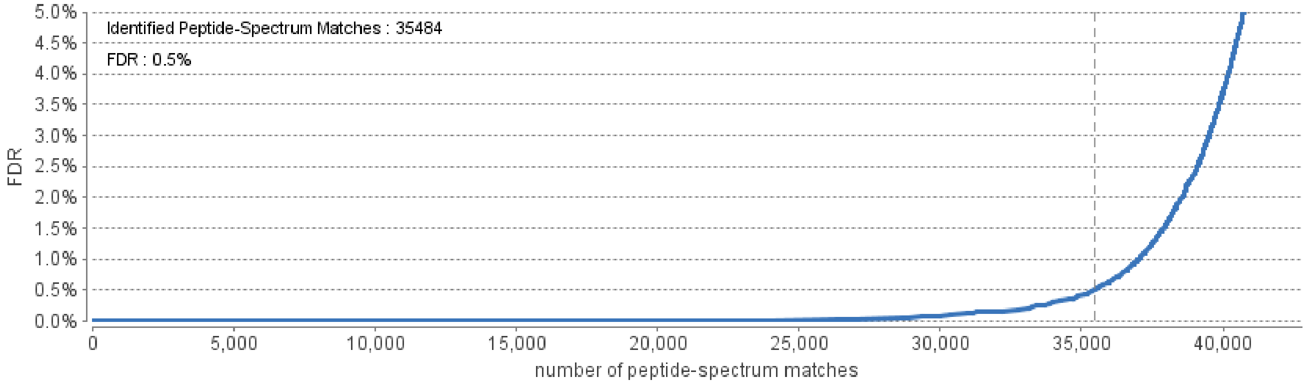

Figure 2. PSM score distribution. **(a)** Distribution of PEAKS peptide score; **(b)** Scatterplot of PEAKS peptide score versus precursor mass error.

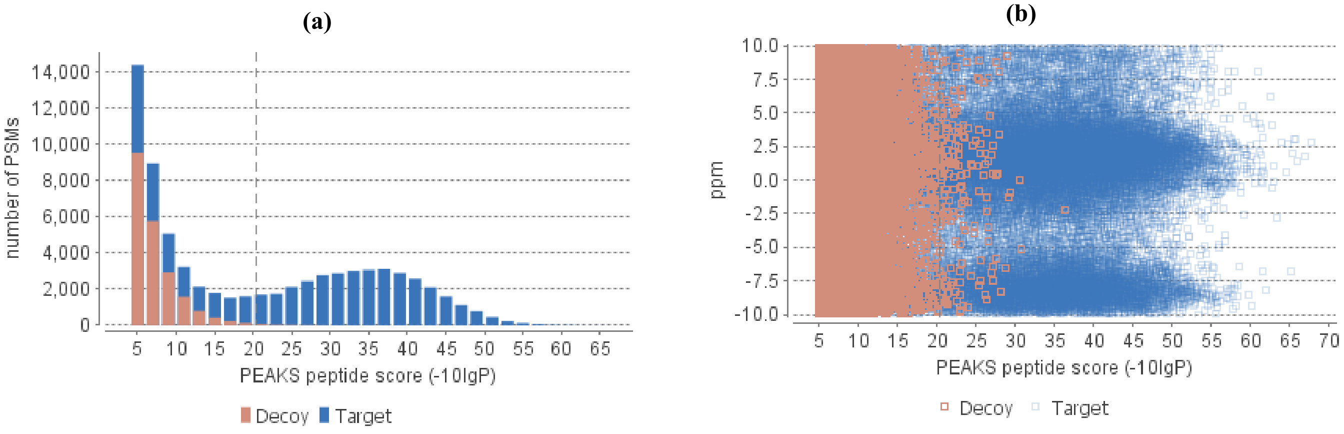

Figure 3. De novo result validation. Distribution of residue local confidence: **(a)** Residues in de novo sequences validated by confident database peptide assignment; **(b)** Residues in "de novo only" sequences.

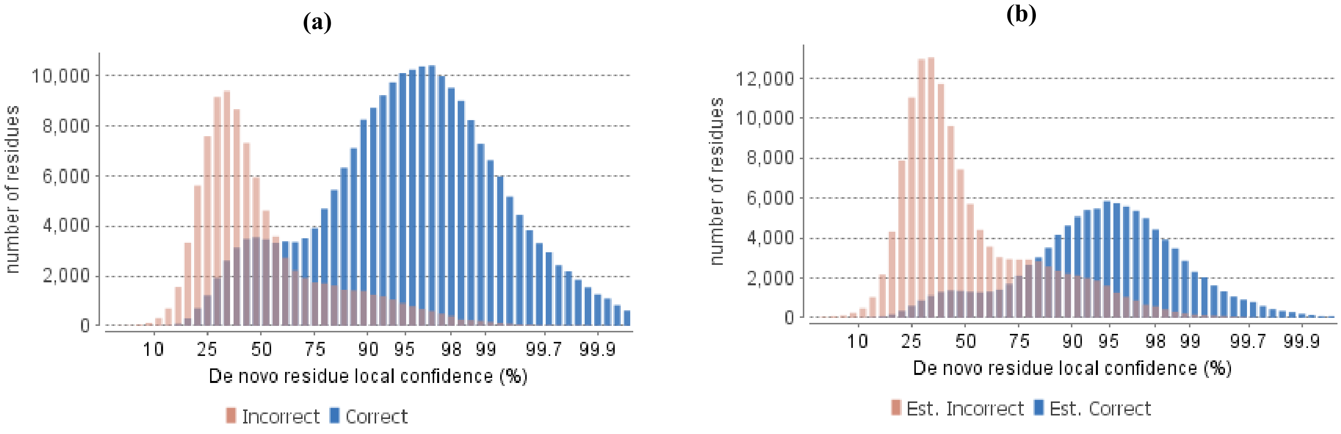

Table 1. Statistics of data.

# of MS scans	25538

Table 4. PTM profile.

Name	ΔMass	Position	#PSM	-10lgP	Area	AScore

# of MS/MS scans 134956

Deamidation	.98	NQ	7396	67.70	1.86E7	26.5[illegible]
Oxidation	15.99	M	3453	63.34		1000.0[illegible]
Acetylation	42.01	N-term	796	57.38		1000.0[illegible]
Carbamidomethyl	57.02	C	110	56.35	5.59E6	1000.0[illegible]

Table 2. Result filtration parameters.

Peptide -10lgP	≥20.3
Peptide Ascore	≥0
Protein -10lgP	≥20
Proteins unique peptides	≥0
De novo ALC Score	≥50%

Table 3. Statistics of filtered result.

Peptide-Spectrum Matches	35484
Peptide sequences	13532
Protein groups	1975
Proteins	3475
Proteins (#Unique Peptides)	1529 (>2); 433 (=2); 1170 (=1);
FDR (Peptide-Spectrum Matches)	0.5%
FDR (Peptide Sequences)	1.1%
De Novo Only Spectra	19180

3. Experiment Control

Figure 4. Precursor mass error of peptide-spectrum matches (PSM) in filtered result. **(a)** Distribution of precursor mass error in ppm; **(b)** Scatterplot of precursor m/z versus precursor mass error in ppm.

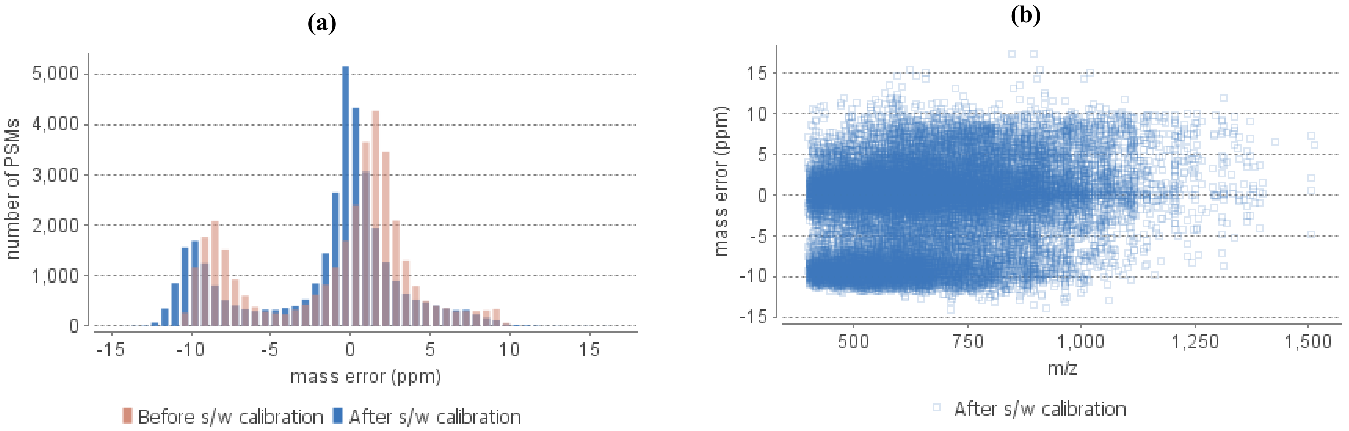

Table 5. Number of identified peptides in each sample by the number of missed cleavages

Missed Cleavages	0	1	2	3	4+
AZ015_01	3937	571	57	0	0
AZ015_02	4201	602	60	0	0
AZ015_03	3523	531	50	0	0

4. Other Information

Table 6. Search parameters.

Search Engine Name: PEAKS
Parent Mass Error Tolerance: 10.0 ppm
Fragment Mass Error Tolerance: 0.05 Da
Precursor Mass Search Type: monoisotopic
Enzyme: Trypsin
Max Missed Cleavages: 2
Non-specific Cleavage: one
Fixed Modifications:

Table 7. Instrument parameters.

Fractions: AZ015_01.raw, AZ015_02.raw, AZ015_03.raw
Ion Source: ESI(nano-spray)
Fragmentation Mode: high energy CID (y and b ions)
MS Scan Mode: FT-ICR/Orbitrap
MS/MS Scan Mode: FT-ICR/Orbitrap

Carbamidomethylation: 57.02
Variable Modifications:
Deamidation (NQ): 0.98
Acetylation (N-term): 42.01
Oxidation (M): 15.99
Max Variable PTM Per Peptide: 3
Database: PF_all
Taxon: All
Searched Entry: 1941073
FDR Estimation: Enabled
Merge Options: no merge
Precursor Options: corrected
Charge Options: no correction
Filter Options: no filter
Process: true

1. Notes

2. Result Statistics

Figure 1. False discovery rate (FDR) curve. X axis is the number of peptide-spectrum matches (PSM) being kept. Y axis is the corresponding FDR.

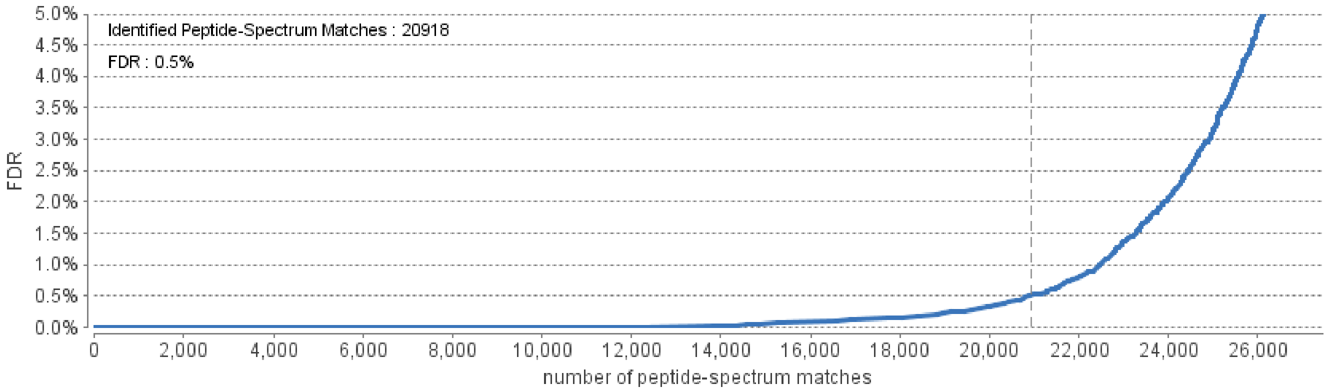

Figure 2. PSM score distribution. **(a)** Distribution of PEAKS peptide score; **(b)** Scatterplot of PEAKS peptide score versus precursor mass error.

(a)

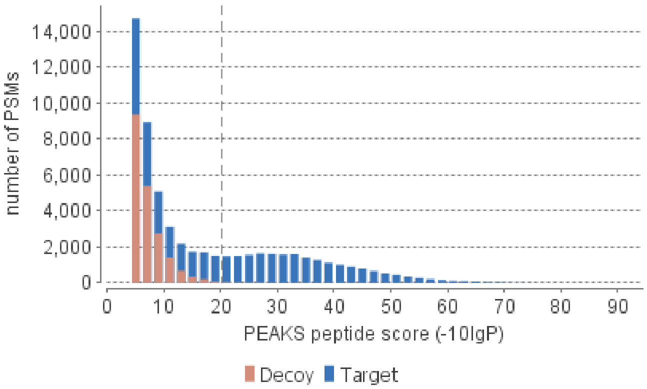

(b)

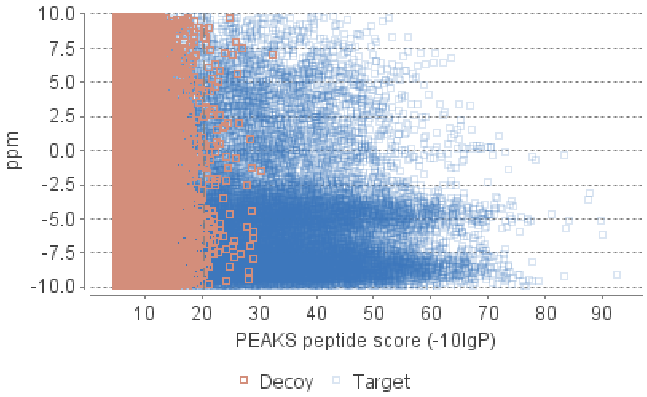

Figure 3. De novo result validation. Distribution of residue local confidence: **(a)** Residues in de novo sequences validated by confident database peptide assignment; **(b)** Residues in "de novo only" sequences.

(a)

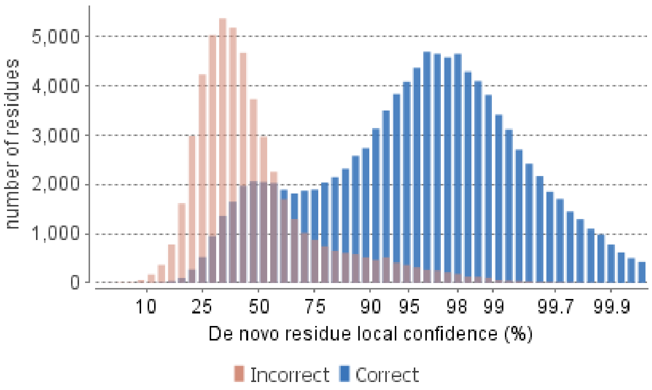

(b)

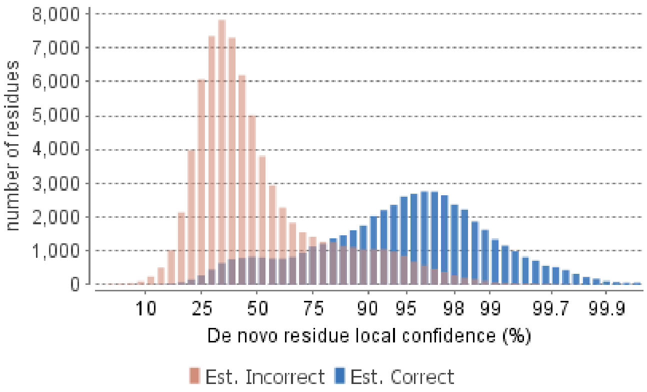

Table 1. Statistics of data.

Table 4. PTM profile.

# of MS scans	29997
# of MS/MS scans	118394

Table 2. Result filtration parameters.

Peptide -10lgP	≥20.3
Peptide Ascore	≥0
Protein -10lgP	≥20
Proteins unique peptides	≥0
De novo ALC Score	≥50%

Table 3. Statistics of filtered result.

Peptide-Spectrum Matches	20918
Peptide sequences	8166
Protein groups	1221
Proteins	2251
Proteins (#Unique Peptides)	882 (>2); 282 (=2); 827 (=1);
FDR (Peptide-Spectrum Matches)	0.5%
FDR (Peptide Sequences)	1.1%
De Novo Only Spectra	12487

Name	ΔMass	Position	#PSM	-10lgP	Area	AScore
Deamidation	.98	NQ	4599	87.64	6.77E6	145.41
Oxidation	15.99	M	1882	92.46	1.23E7	1000.00
Acetylation	42.01	N-term	289	61.63		1000.00
Carbamidomethyl	57.02	C	41	52.31	9.13E6	1000.00

3. Experiment Control

Figure 4. Precursor mass error of peptide-spectrum matches (PSM) in filtered result. **(a)** Distribution of precursor mass error in ppm; **(b)** Scatterplot of precursor m/z versus precursor mass error in ppm.

(a) **(b)**

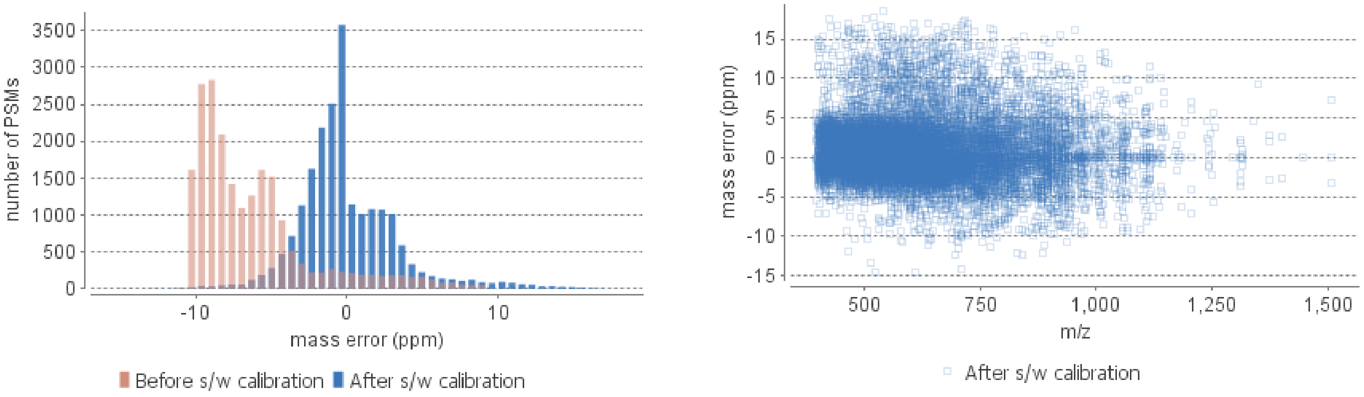

Table 5. Number of identified peptides in each sample by the number of missed cleavages

Missed Cleavages	0	1	2	3	4+
AZ017_01	2333	316	22	0	0
AZ017_02	2813	335	19	0	0
AZ017_03	2064	249	15	0	0

4. Other Information

Table 6. Search parameters.

Search Engine Name: PEAKS
Parent Mass Error Tolerance: 10.0 ppm
Fragment Mass Error Tolerance: 0.05 Da
Precursor Mass Search Type: monoisotopic
Enzyme: Trypsin
Max Missed Cleavages: 2

Table 7. Instrument parameters.

Fractions: AZ017_01.raw, AZ017_02.raw, AZ017_03.raw
Ion Source: ESI(nano-spray)
Fragmentation Mode: high energy CID (y and b ions)
MS Scan Mode: FT-ICR/Orbitrap
MS/MS Scan Mode: FT-ICR/Orbitrap

Non-specific Cleavage: one
Fixed Modifications:
  Carbamidomethylation: 57.02
Variable Modifications:
  Deamidation (NQ): 0.98
  Acetylation (N-term): 42.01
  Oxidation (M): 15.99
Max Variable PTM Per Peptide: 3
Database: PF_all
Taxon: All
Searched Entry: 1941073
FDR Estimation: Enabled
Merge Options: no merge
Precursor Options: corrected
Charge Options: no correction
Filter Options: no filter
Process: true

1. Notes

2. Result Statistics

Figure 1. False discovery rate (FDR) curve. X axis is the number of peptide-spectrum matches (PSM) being kept. Y axis is the corresponding FDR.

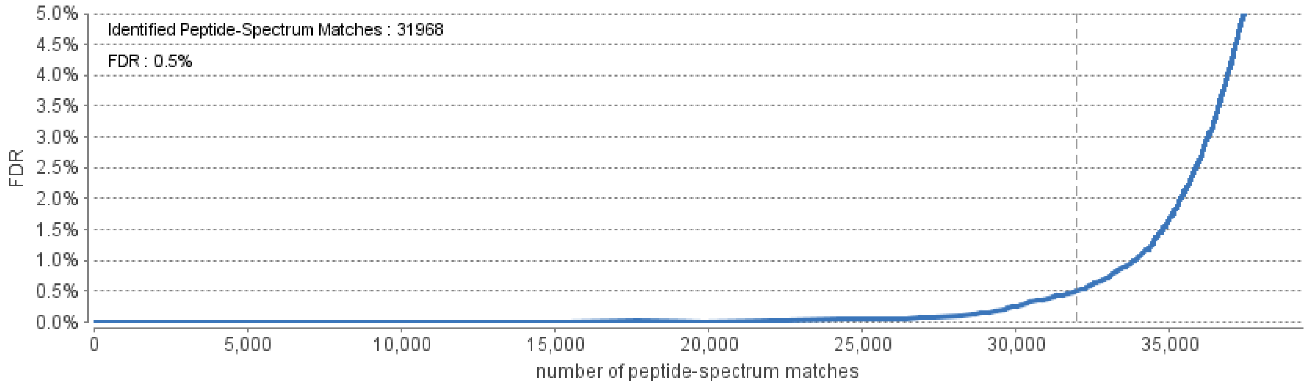

Figure 2. PSM score distribution. **(a)** Distribution of PEAKS peptide score; **(b)** Scatterplot of PEAKS peptide score versus precursor mass error.

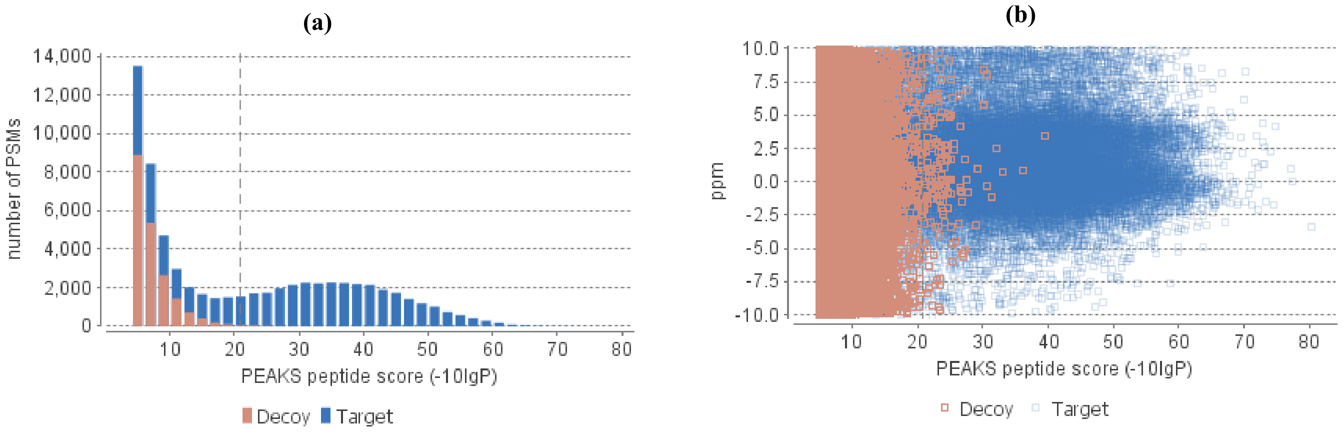

Figure 3. De novo result validation. Distribution of residue local confidence: **(a)** Residues in de novo sequences validated by confident database peptide assignment; **(b)** Residues in "de novo only" sequences.

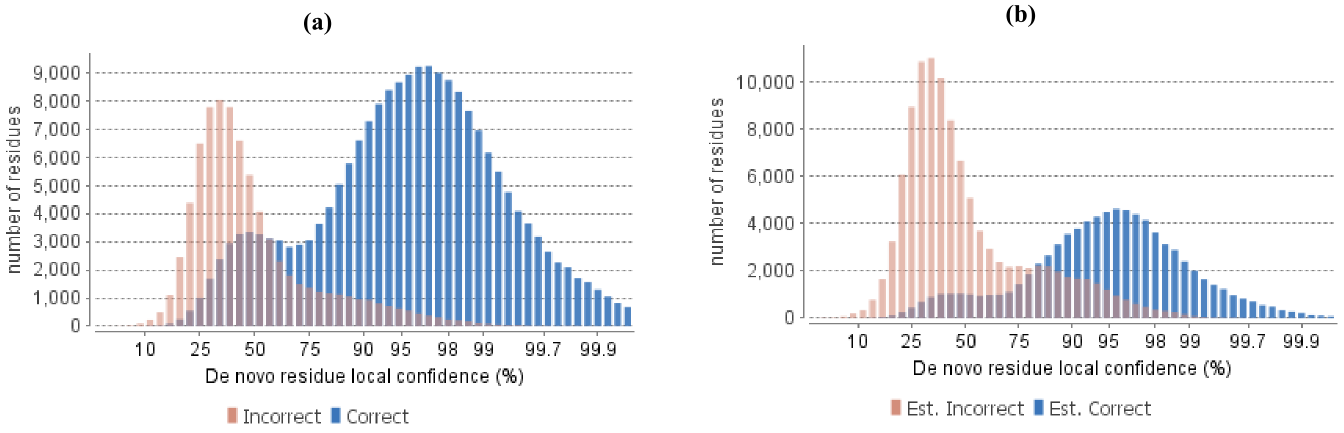

Table 1. Statistics of data.

# of MS scans	27458

Table 4. PTM profile.

Name	ΔMass	Position	#PSM	-10lgP	Area	AScor

# of MS/MS scans 127852

Deamidation	.98	NQ	5620	80.20	1.63E6	47.0[illegible]
Oxidation	15.99	M	3494	72.99	1.09E8	1000.0[illegible]
Acetylation	42.01	N-term	574	56.97		1000.0[illegible]
Carbamidomethyl	57.02	C	64	63.93		1000.0[illegible]

Table 2. Result filtration parameters.

Peptide -10lgP	≥20.8
Peptide Ascore	≥0
Protein -10lgP	≥20
Proteins unique peptides	≥0
De novo ALC Score	≥50%

Table 3. Statistics of filtered result.

Peptide-Spectrum Matches	31968
Peptide sequences	12128
Protein groups	1801
Proteins	3373
Proteins (#Unique Peptides)	1381 (>2); 463 (=2); 1259 (=1);
FDR (Peptide-Spectrum Matches)	0.5%
FDR (Peptide Sequences)	1.0%
De Novo Only Spectra	15781

3. Experiment Control

Figure 4. Precursor mass error of peptide-spectrum matches (PSM) in filtered result. **(a)** Distribution of precursor mass error in ppm; **(b)** Scatterplot of precursor m/z versus precursor mass error in ppm.

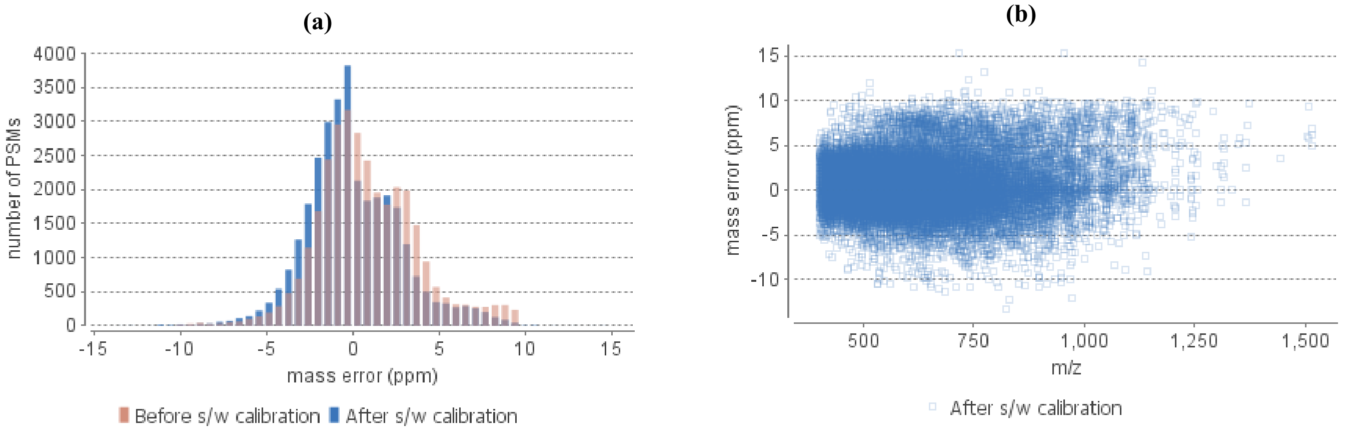

Table 5. Number of identified peptides in each sample by the number of missed cleavages

Missed Cleavages	0	1	2	3	4+
AZ018_01	3251	548	38	0	0
AZ018_02	3118	500	56	0	0
AZ018_03	3935	622	60	0	0

4. Other Information

Table 6. Search parameters.

Search Engine Name: PEAKS
Parent Mass Error Tolerance: 10.0 ppm
Fragment Mass Error Tolerance: 0.05 Da
Precursor Mass Search Type: monoisotopic
Enzyme: Trypsin
Max Missed Cleavages: 2
Non-specific Cleavage: one
Fixed Modifications:

Table 7. Instrument parameters.

Fractions: AZ018_01.raw, AZ018_02.raw, AZ018_03.raw
Ion Source: ESI(nano-spray)
Fragmentation Mode: high energy CID (y and b ions)
MS Scan Mode: FT-ICR/Orbitrap
MS/MS Scan Mode: FT-ICR/Orbitrap

Carbamidomethylation: 57.02
Variable Modifications:
Deamidation (NQ): 0.98
Acetylation (N-term): 42.01
Oxidation (M): 15.99
Max Variable PTM Per Peptide: 3
Database: PF_all
Taxon: All
Searched Entry: 1941073
FDR Estimation: Enabled
Merge Options: no merge
Precursor Options: corrected
Charge Options: no correction
Filter Options: no filter
Process: true

1. Notes

2. Result Statistics

Figure 1. False discovery rate (FDR) curve. X axis is the number of peptide-spectrum matches (PSM) being kept. Y axis is the corresponding FDR.

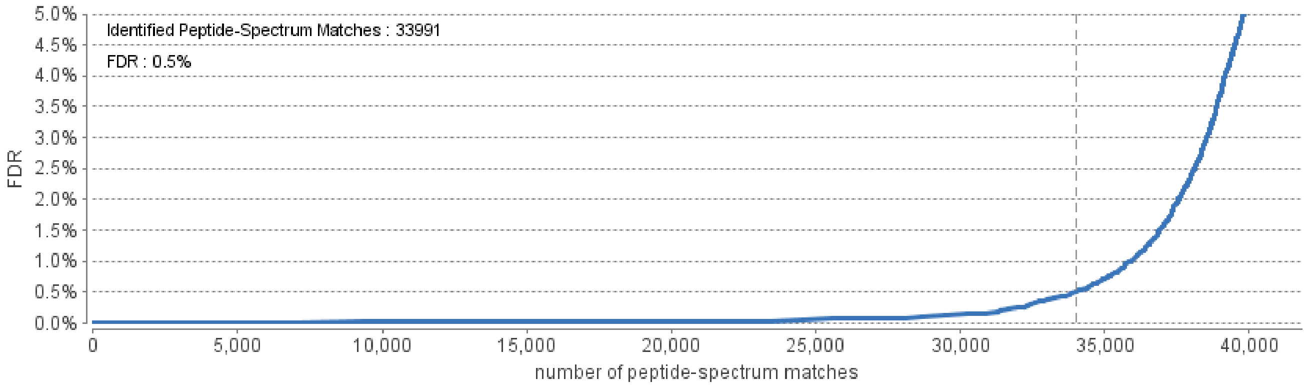

Figure 2. PSM score distribution. **(a)** Distribution of PEAKS peptide score; **(b)** Scatterplot of PEAKS peptide score versus precursor mass error.

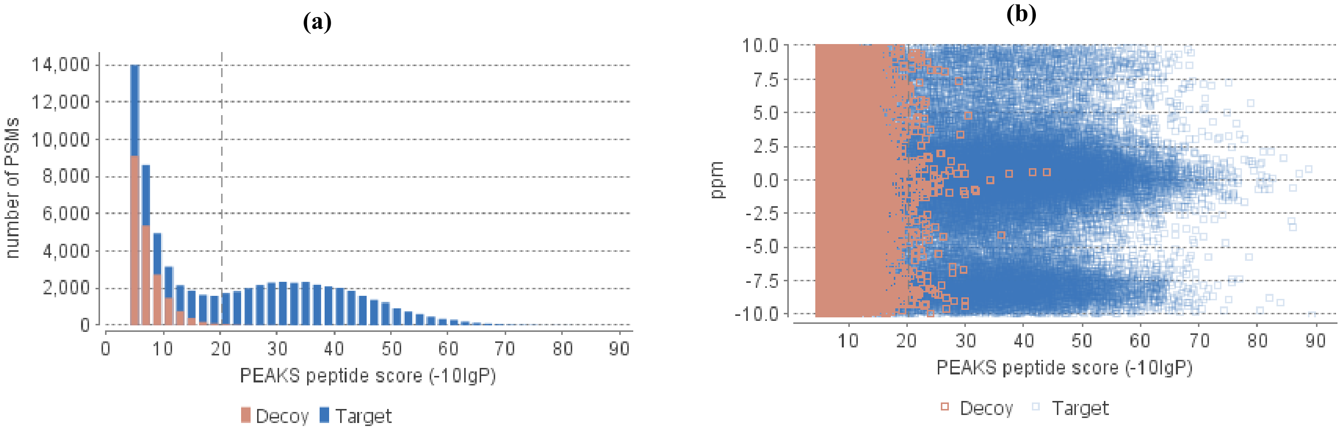

Figure 3. De novo result validation. Distribution of residue local confidence: **(a)** Residues in de novo sequences validated by confident database peptide assignment; **(b)** Residues in "de novo only" sequences.

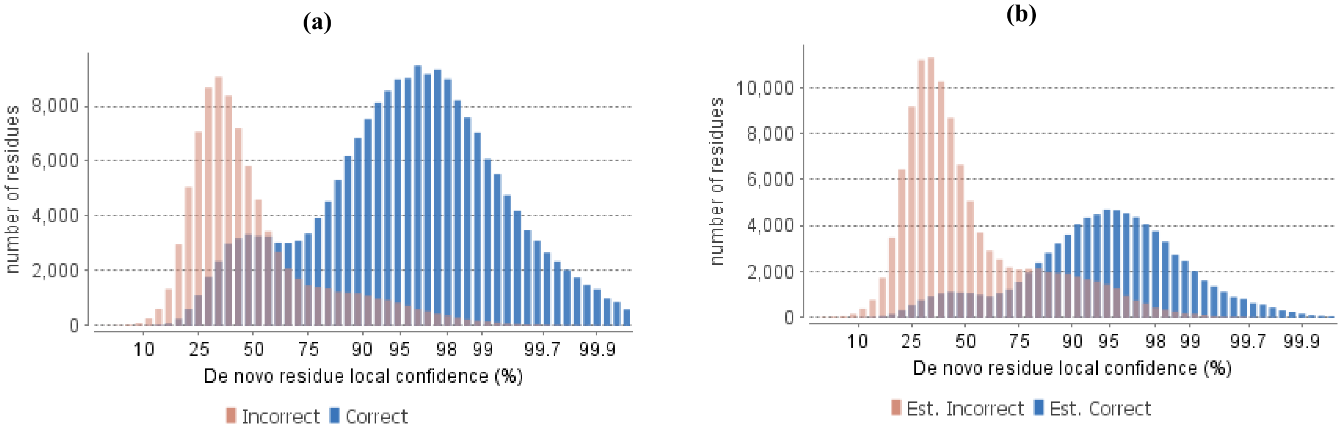

Table 1. Statistics of data.

# of MS scans	26882

Table 4. PTM profile.

Name	ΔMass	Position	#PSM	-10lgP	Area	AScore

# of MS/MS scans 130106

Deamidation	.98	NQ	7526	88.78	5.4E6	47.0[illegible]
Oxidation	15.99	M	2840	80.11	8.61E7	1000.0[illegible]
Acetylation	42.01	N-term	709	67.61		1000.0[illegible]
Carbamidomethyl	57.02	C	102	55.81	2.41E7	1000.0[illegible]

Table 2. Result filtration parameters.

Peptide -10lgP	≥20.2
Peptide Ascore	≥0
Protein -10lgP	≥20
Proteins unique peptides	≥0
De novo ALC Score	≥50%

Table 3. Statistics of filtered result.

Peptide-Spectrum Matches	33991
Peptide sequences	12759
Protein groups	1817
Proteins	3215
Proteins (#Unique Peptides)	1395 (>2); 411 (=2); 1107 (=1);
FDR (Peptide-Spectrum Matches)	0.5%
FDR (Peptide Sequences)	1.1%
De Novo Only Spectra	16464

3. Experiment Control

Figure 4. Precursor mass error of peptide-spectrum matches (PSM) in filtered result. **(a)** Distribution of precursor mass error in ppm; **(b)** Scatterplot of precursor m/z versus precursor mass error in ppm.

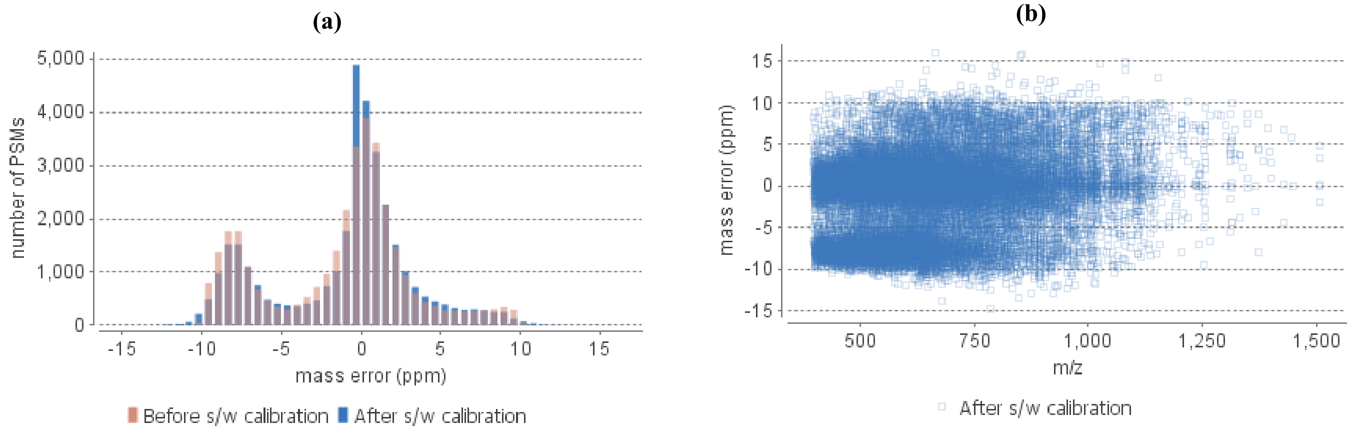

Table 5. Number of identified peptides in each sample by the number of missed cleavages

Missed Cleavages	0	1	2	3	4+
AZ020_01	3665	611	48	0	0
AZ020_02	4192	571	39	0	0
AZ020_03	3092	499	42	0	0

4. Other Information

Table 6. Search parameters.

Search Engine Name: PEAKS
Parent Mass Error Tolerance: 10.0 ppm
Fragment Mass Error Tolerance: 0.05 Da
Precursor Mass Search Type: monoisotopic
Enzyme: Trypsin
Max Missed Cleavages: 2
Non-specific Cleavage: one
Fixed Modifications:

Table 7. Instrument parameters.

Fractions: AZ020_01.raw, AZ020_02.raw, AZ020_03.raw
Ion Source: ESI(nano-spray)
Fragmentation Mode: high energy CID (y and b ions)
MS Scan Mode: FT-ICR/Orbitrap
MS/MS Scan Mode: FT-ICR/Orbitrap

Carbamidomethylation: 57.02
Variable Modifications:
Deamidation (NQ): 0.98
Acetylation (N-term): 42.01
Oxidation (M): 15.99
Max Variable PTM Per Peptide: 3
Database: PF_all
Taxon: All
Searched Entry: 1941073
FDR Estimation: Enabled
Merge Options: no merge
Precursor Options: corrected
Charge Options: no correction
Filter Options: no filter
Process: true

1. Notes

2. Result Statistics

Figure 1. False discovery rate (FDR) curve. X axis is the number of peptide-spectrum matches (PSM) being kept. Y axis is the corresponding FDR.

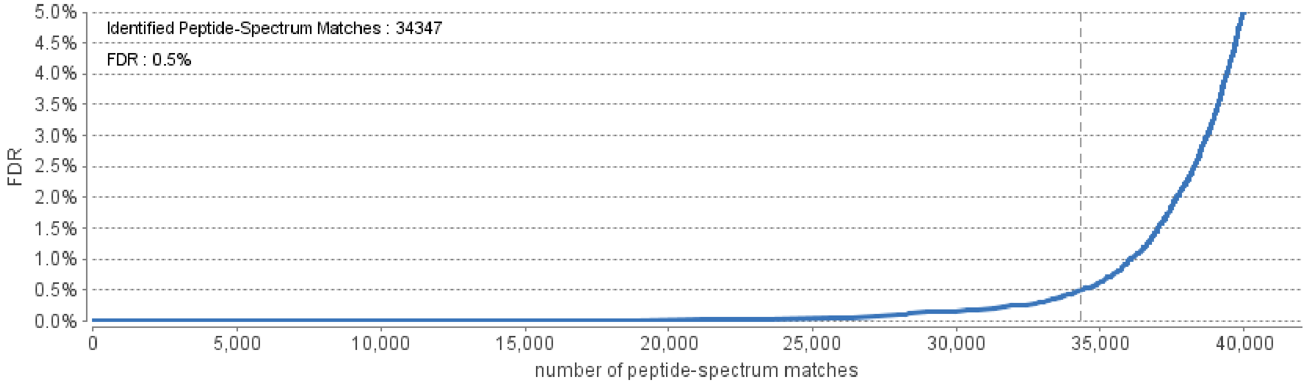

Figure 2. PSM score distribution. **(a)** Distribution of PEAKS peptide score; **(b)** Scatterplot of PEAKS peptide score versus precursor mass error.

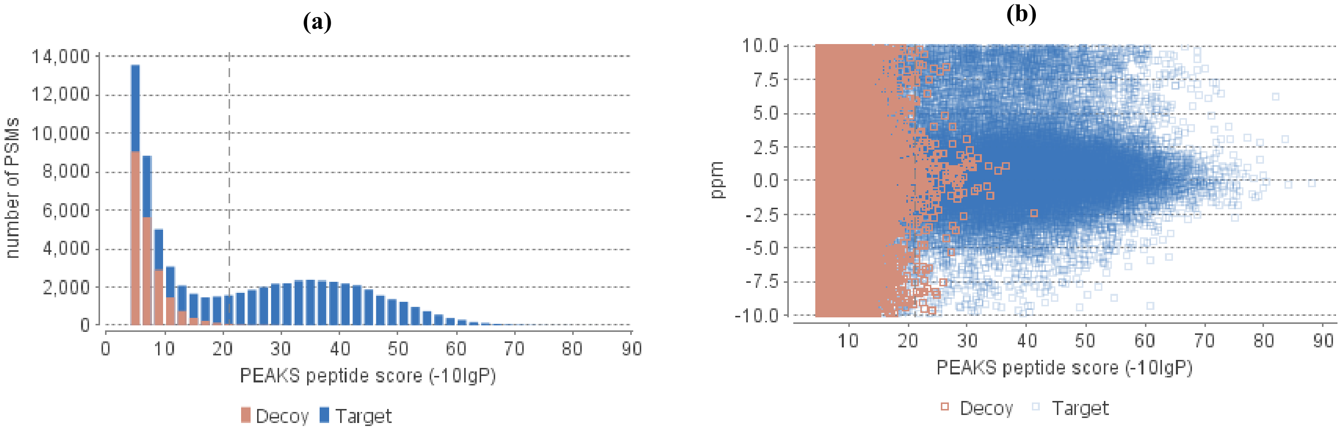

Figure 3. De novo result validation. Distribution of residue local confidence: **(a)** Residues in de novo sequences validated by confident database peptide assignment; **(b)** Residues in "de novo only" sequences.

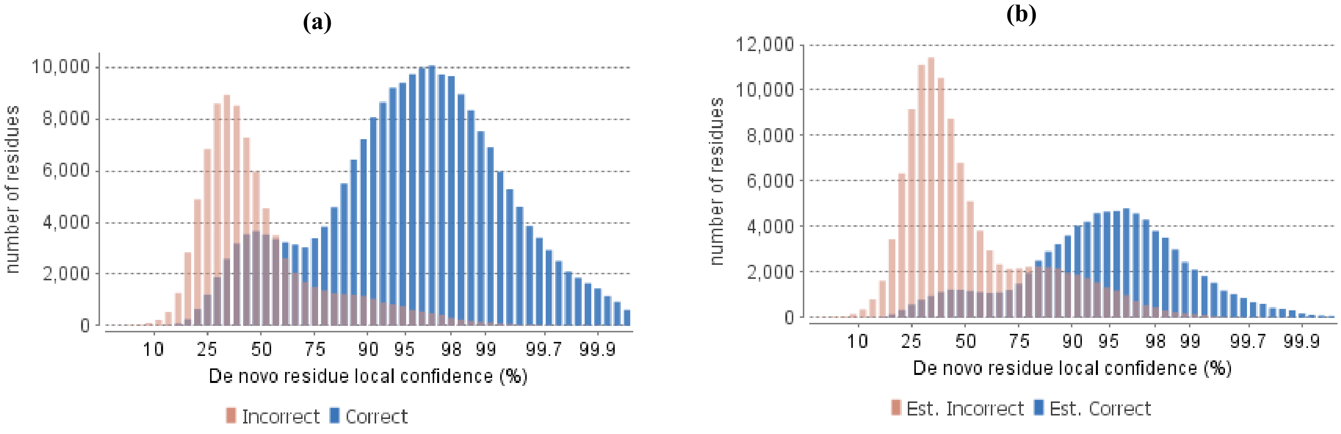

Table 1. Statistics of data.

# of MS scans	26843

Table 4. PTM profile.

Name	ΔMass	Position	#PSM	-10lgP	Area	AScore

# of MS/MS scans 130687

Table 2. Result filtration parameters.

Peptide -10lgP	≥21.1
Peptide Ascore	≥0
Protein -10lgP	≥20
Proteins unique peptides	≥0
De novo ALC Score	≥50%

Table 3. Statistics of filtered result.

Peptide-Spectrum Matches	34347
Peptide sequences	13272
Protein groups	1912
Proteins	3340
Proteins (#Unique Peptides)	1487 (>2); 411 (=2); 1126 (=1);
FDR (Peptide-Spectrum Matches)	0.5%
FDR (Peptide Sequences)	0.8%
De Novo Only Spectra	16641

Deamidation	.98	NQ	6298	88.08	4.45E6	47.0[illegible]
Oxidation	15.99	M	2654	88.08	4.45E6	1000.0[illegible]
Acetylation	42.01	N-term	732	60.87		1000.0[illegible]
Carbamidomethyl	57.02	C	85	71.15	3.66E6	1000.0[illegible]

3. Experiment Control

Figure 4. Precursor mass error of peptide-spectrum matches (PSM) in filtered result. **(a)** Distribution of precursor mass error in ppm; **(b)** Scatterplot of precursor m/z versus precursor mass error in ppm.

(a)

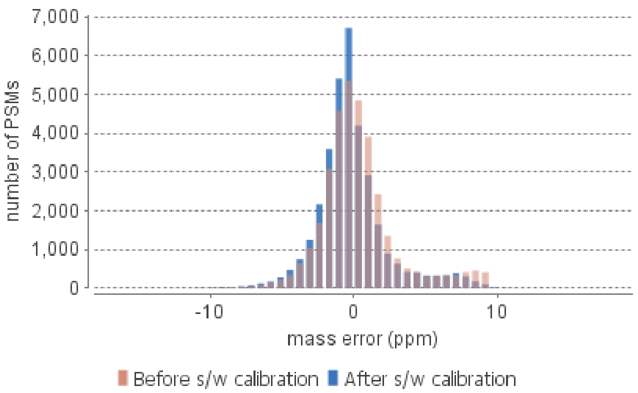

(b)

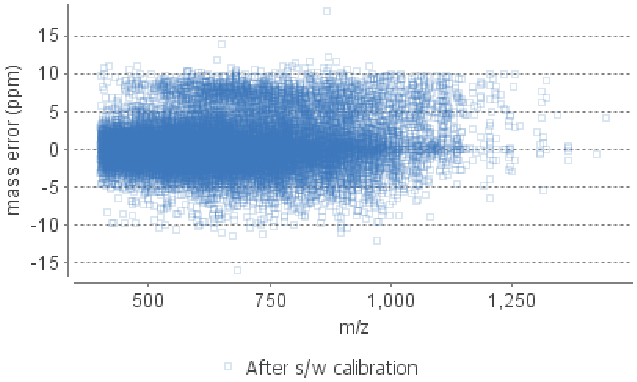

Table 5. Number of identified peptides in each sample by the number of missed cleavages

Missed Cleavages	0	1	2	3	4+
AZ021_01	4286	650	56	0	0
AZ021_02	3824	563	45	0	0
AZ021_03	3314	497	37	0	0

4. Other Information

Table 6. Search parameters.

Search Engine Name: PEAKS
Parent Mass Error Tolerance: 10.0 ppm
Fragment Mass Error Tolerance: 0.05 Da
Precursor Mass Search Type: monoisotopic
Enzyme: Trypsin
Max Missed Cleavages: 2
Non-specific Cleavage: one
Fixed Modifications:

Table 7. Instrument parameters.

Fractions: AZ021_01.raw, AZ021_02.raw, AZ021_03.raw
Ion Source: ESI(nano-spray)
Fragmentation Mode: high energy CID (y and b ions)
MS Scan Mode: FT-ICR/Orbitrap
MS/MS Scan Mode: FT-ICR/Orbitrap

Carbamidomethylation: 57.02
Variable Modifications:
Deamidation (NQ): 0.98
Acetylation (N-term): 42.01
Oxidation (M): 15.99
Max Variable PTM Per Peptide: 3
Database: PF_all
Taxon: All
Searched Entry: 1941073
FDR Estimation: Enabled
Merge Options: no merge
Precursor Options: corrected
Charge Options: no correction
Filter Options: no filter
Process: true

1. Notes

2. Result Statistics

Figure 1. False discovery rate (FDR) curve. X axis is the number of peptide-spectrum matches (PSM) being kept. Y axis is the corresponding FDR.

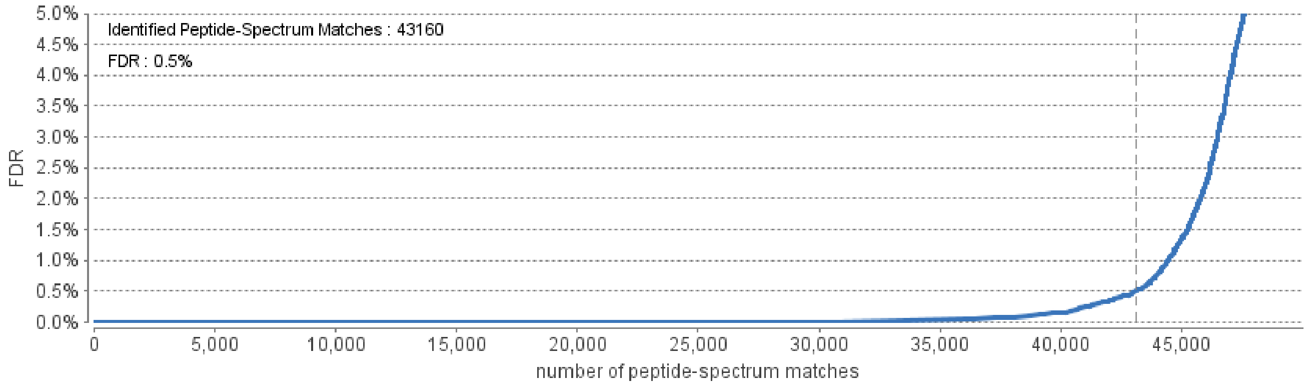

Figure 2. PSM score distribution. **(a)** Distribution of PEAKS peptide score; **(b)** Scatterplot of PEAKS peptide score versus precursor mass error.

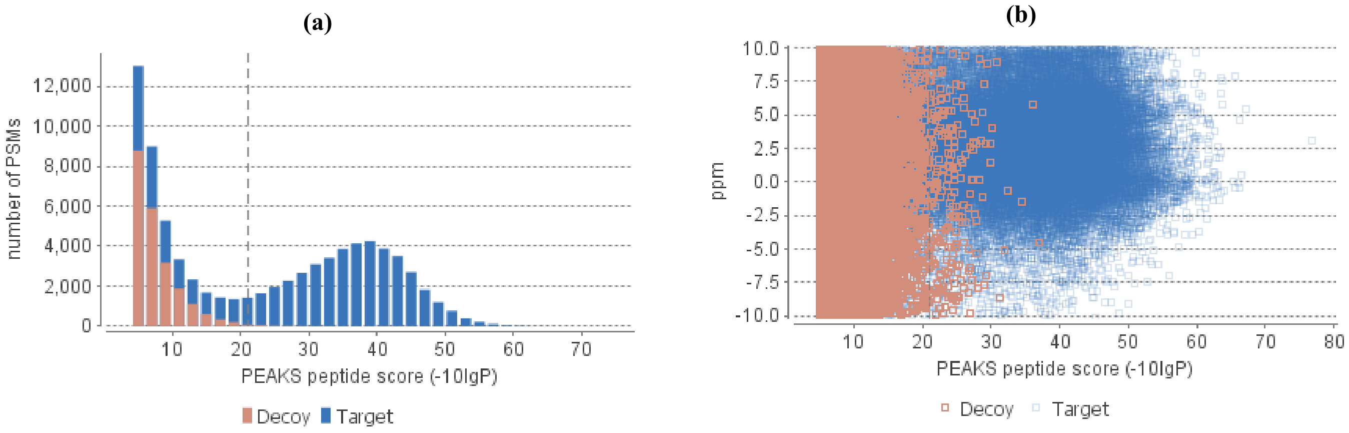

Figure 3. De novo result validation. Distribution of residue local confidence: **(a)** Residues in de novo sequences validated by confident database peptide assignment; **(b)** Residues in "de novo only" sequences.

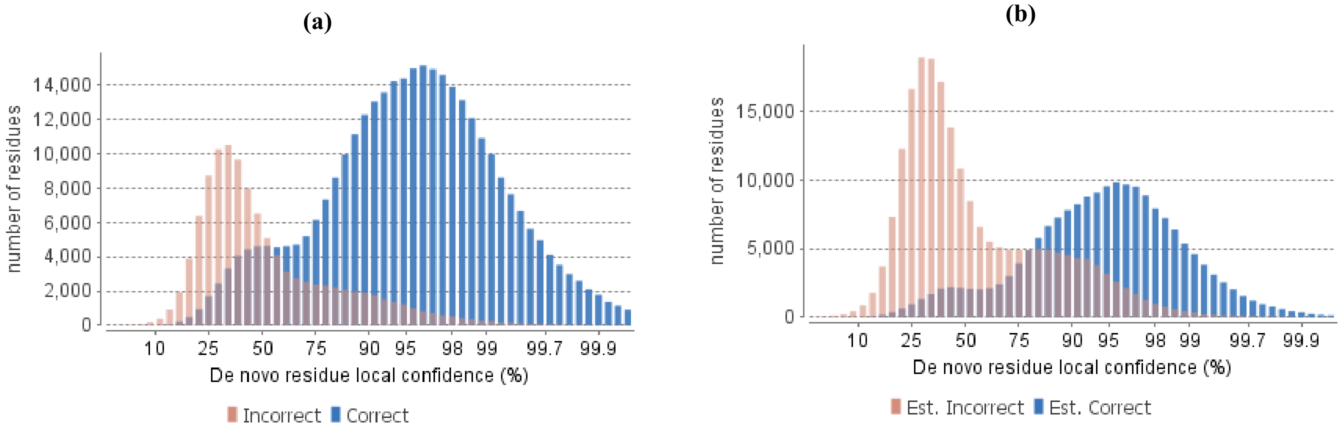

Table 1. Statistics of data.

# of MS scans	23506

Table 4. PTM profile.

Name	ΔMass	Position	#PSM	-10lgP	Area	AScor

# of MS/MS scans 143118

Deamidation	.98	NQ	9275	65.51		49.2[illegible]
Oxidation	15.99	M	4709	63.64		1000.0[illegible]
Acetylation	42.01	N-term	1247	66.22		1000.0[illegible]
Carbamidomethyl	57.02	C	252	52.15	4.92E6	1000.0[illegible]

Table 2. Result filtration parameters.

Peptide -10lgP	≥21
Peptide Ascore	≥0
Protein -10lgP	≥20
Proteins unique peptides	≥0
De novo ALC Score	≥50%

Table 3. Statistics of filtered result.

Peptide-Spectrum Matches	43160
Peptide sequences	15979
Protein groups	2266
Proteins	3647
Proteins (#Unique Peptides)	1592 (>2); 448 (=2); 1234 (=1);
FDR (Peptide-Spectrum Matches)	0.5%
FDR (Peptide Sequences)	0.9%
De Novo Only Spectra	28295

3. Experiment Control

Figure 4. Precursor mass error of peptide-spectrum matches (PSM) in filtered result. **(a)** Distribution of precursor mass error in ppm; **(b)** Scatterplot of precursor m/z versus precursor mass error in ppm.

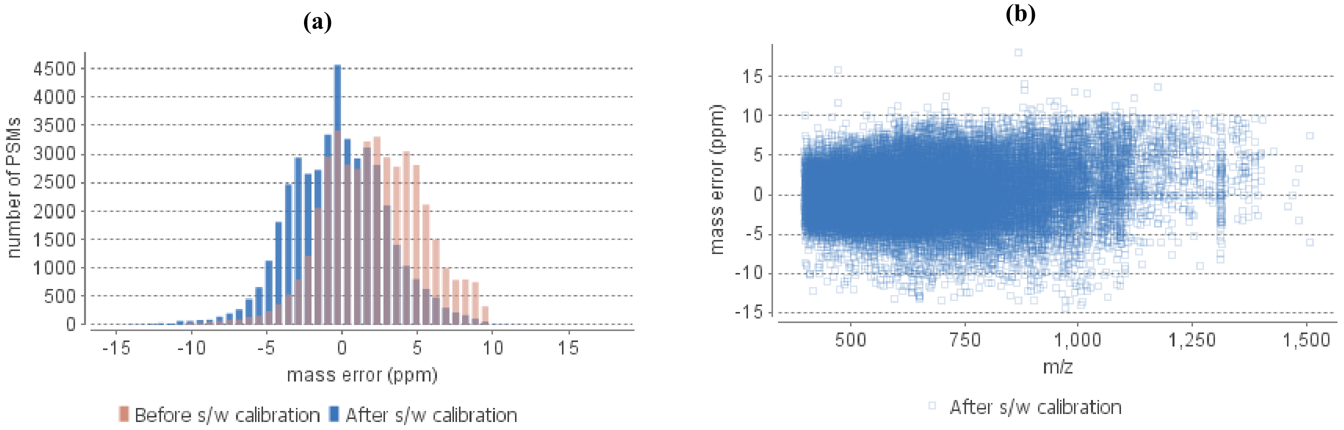

Table 5. Number of identified peptides in each sample by the number of missed cleavages

Missed Cleavages	0	1	2	3	4+
AZ022_01	4722	974	148	0	0
AZ022_02	4556	921	132	0	0
AZ022_03	3744	690	92	0	0

4. Other Information

Table 6. Search parameters.

Search Engine Name: PEAKS
Parent Mass Error Tolerance: 10.0 ppm
Fragment Mass Error Tolerance: 0.05 Da
Precursor Mass Search Type: monoisotopic
Enzyme: Trypsin
Max Missed Cleavages: 2
Non-specific Cleavage: one
Fixed Modifications:

Table 7. Instrument parameters.

Fractions: AZ022_01.raw, AZ022_02.raw, AZ022_03.raw
Ion Source: ESI(nano-spray)
Fragmentation Mode: high energy CID (y and b ions)
MS Scan Mode: FT-ICR/Orbitrap
MS/MS Scan Mode: FT-ICR/Orbitrap

Carbamidomethylation: 57.02
Variable Modifications:
Deamidation (NQ): 0.98
Acetylation (N-term): 42.01
Oxidation (M): 15.99
Max Variable PTM Per Peptide: 3
Database: PF_all
Taxon: All
Searched Entry: 1941073
FDR Estimation: Enabled
Merge Options: no merge
Precursor Options: corrected
Charge Options: no correction
Filter Options: no filter
Process: true

1. Notes

2. Result Statistics

Figure 1. False discovery rate (FDR) curve. X axis is the number of peptide-spectrum matches (PSM) being kept. Y axis is the corresponding FDR.

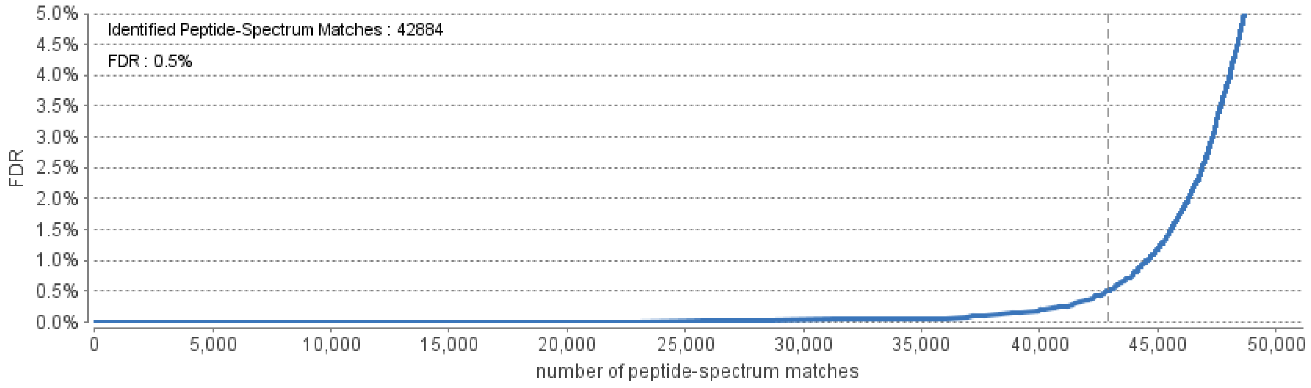

Figure 2. PSM score distribution. **(a)** Distribution of PEAKS peptide score; **(b)** Scatterplot of PEAKS peptide score versus precursor mass error.

(a)

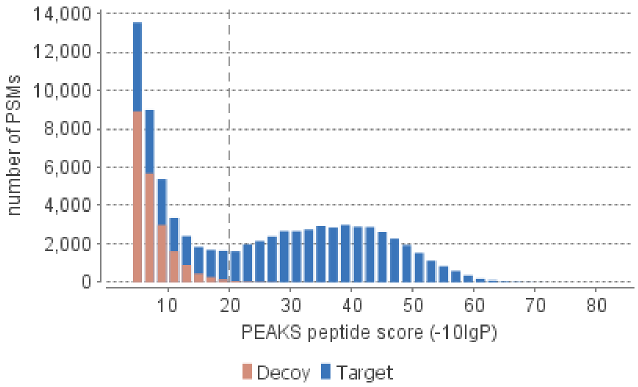

(b)

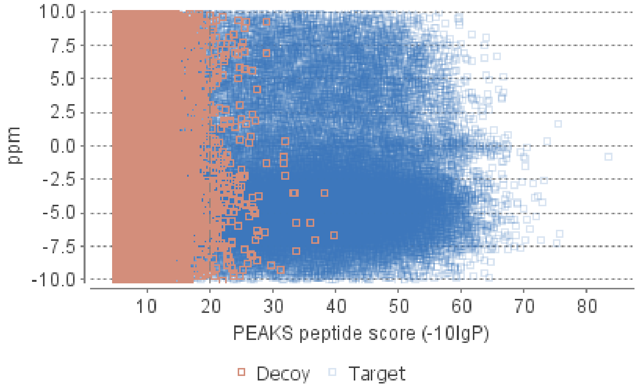

Figure 3. De novo result validation. Distribution of residue local confidence: **(a)** Residues in de novo sequences validated by confident database peptide assignment; **(b)** Residues in "de novo only" sequences.

(a)

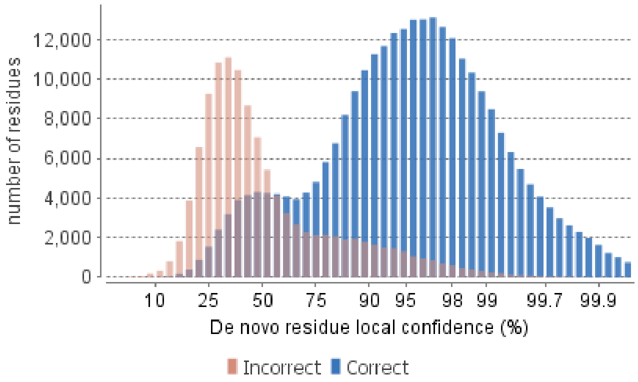

(b)

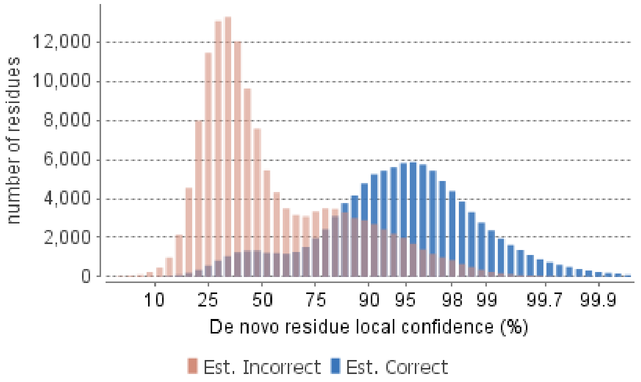

Table 1. Statistics of data.

# of MS scans	25107

Table 4. PTM profile.

Name	ΔMass	Position	#PSM	-10lgP	Area	AScor

# of MS/MS scans 137078

Deamidation	.98	NQ	10659	83.42		47.0[illegible]
Oxidation	15.99	M	5063	70.46	4.51E6	1000.0[illegible]
Acetylation	42.01	N-term	1135	66.42	3.92E6	1000.0[illegible]
Carbamidomethyl	57.02	C	187	59.87	1.08E7	1000.0[illegible]

Table 2. Result filtration parameters.

Peptide -10lgP	≥20.1
Peptide Ascore	≥0
Protein -10lgP	≥20
Proteins unique peptides	≥0
De novo ALC Score	≥50%

Table 3. Statistics of filtered result.

Peptide-Spectrum Matches	42884
Peptide sequences	16044
Protein groups	2011
Proteins	3514
Proteins (#Unique Peptides)	1568 (>2); 427 (=2); 1218 (=1);
FDR (Peptide-Spectrum Matches)	0.5%
FDR (Peptide Sequences)	1.0%
De Novo Only Spectra	19574

3. Experiment Control

Figure 4. Precursor mass error of peptide-spectrum matches (PSM) in filtered result. **(a)** Distribution of precursor mass error in ppm; **(b)** Scatterplot of precursor m/z versus precursor mass error in ppm.

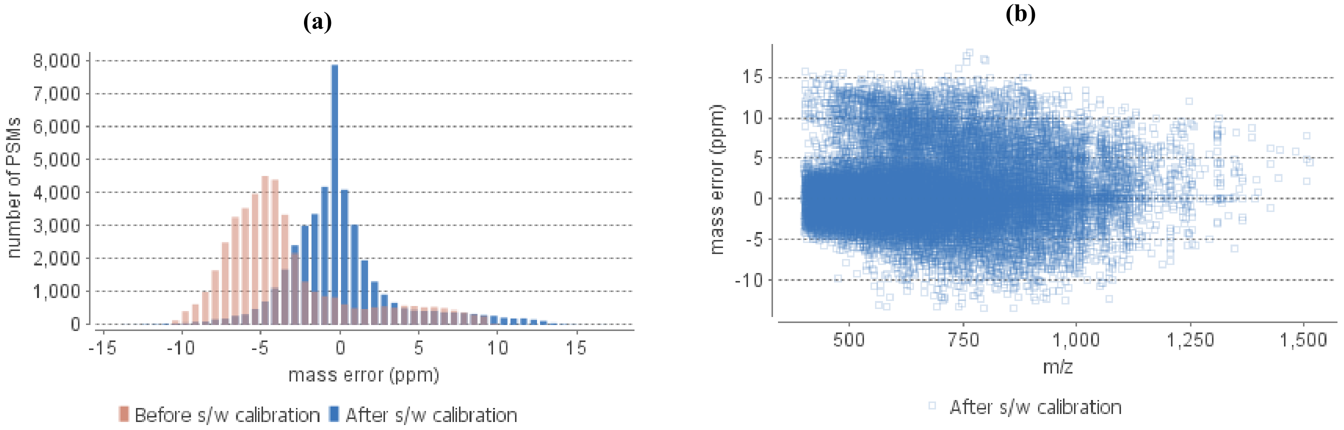

Table 5. Number of identified peptides in each sample by the number of missed cleavages

Missed Cleavages	0	1	2	3	4+
AZ025_01	5451	931	70	0	0
AZ025_02	4568	735	73	0	0
AZ025_03	3616	564	36	0	0

4. Other Information

Table 6. Search parameters.

Search Engine Name: PEAKS
Parent Mass Error Tolerance: 10.0 ppm
Fragment Mass Error Tolerance: 0.05 Da
Precursor Mass Search Type: monoisotopic
Enzyme: Trypsin
Max Missed Cleavages: 2
Non-specific Cleavage: one
Fixed Modifications:

Table 7. Instrument parameters.

Fractions: AZ025_01.raw, AZ025_02.raw, AZ025_03.raw
Ion Source: ESI(nano-spray)
Fragmentation Mode: high energy CID (y and b ions)
MS Scan Mode: FT-ICR/Orbitrap
MS/MS Scan Mode: FT-ICR/Orbitrap

Carbamidomethylation: 57.02
Variable Modifications:
Deamidation (NQ): 0.98
Acetylation (N-term): 42.01
Oxidation (M): 15.99
Max Variable PTM Per Peptide: 3
Database: PF_all
Taxon: All
Searched Entry: 1941073
FDR Estimation: Enabled
Merge Options: no merge
Precursor Options: corrected
Charge Options: no correction
Filter Options: no filter
Process: true

1. Notes

2. Result Statistics

Figure 1. False discovery rate (FDR) curve. X axis is the number of peptide-spectrum matches (PSM) being kept. Y axis is the corresponding FDR.

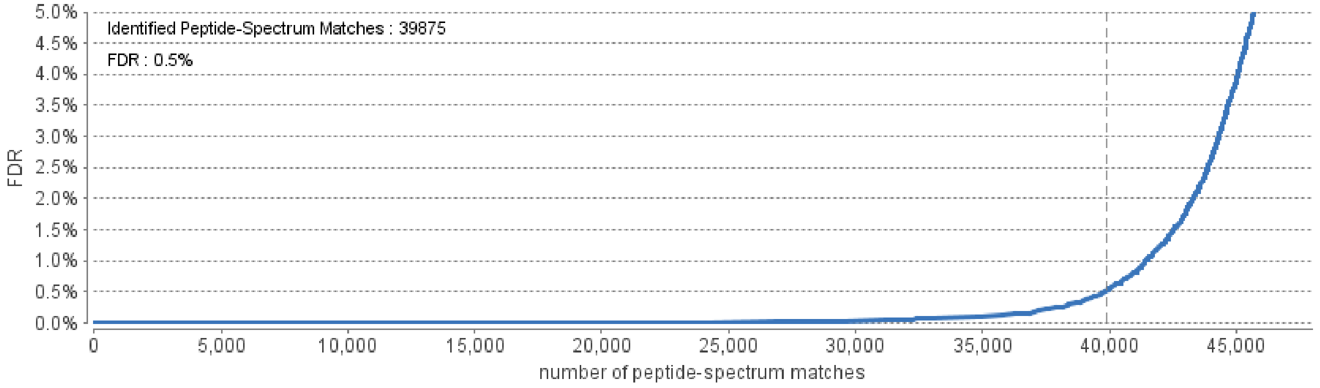

Figure 2. PSM score distribution. **(a)** Distribution of PEAKS peptide score; **(b)** Scatterplot of PEAKS peptide score versus precursor mass error.

(a) **(b)**

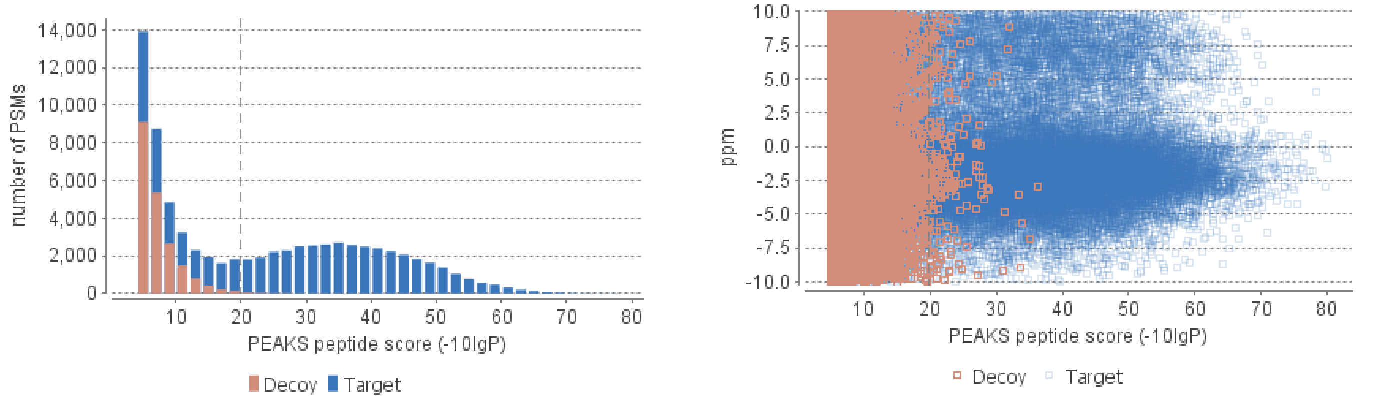

Figure 3. De novo result validation. Distribution of residue local confidence: **(a)** Residues in de novo sequences validated by confident database peptide assignment; **(b)** Residues in "de novo only" sequences.

(a) **(b)**

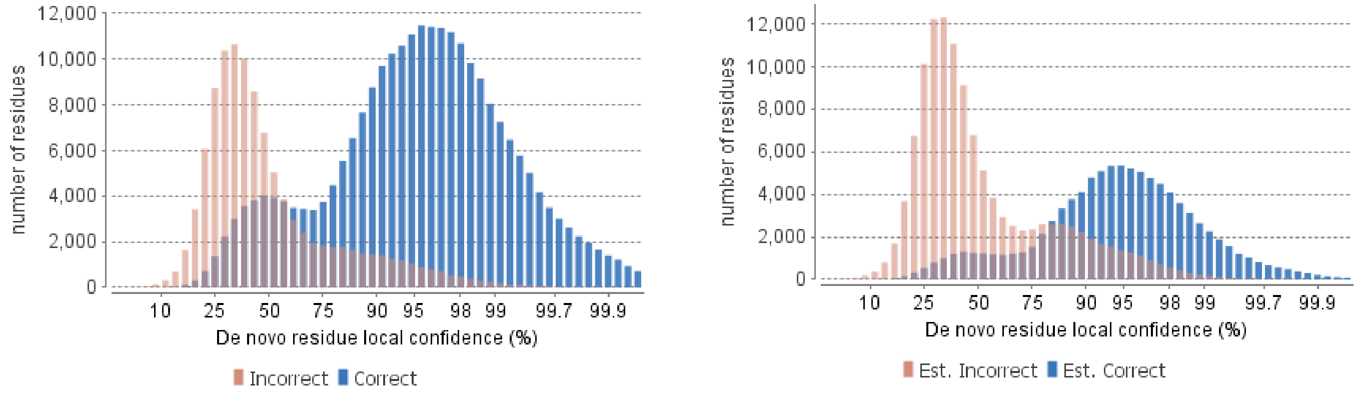

Table 1. Statistics of data.

Table 4. PTM profile.

# of MS scans	25893
# of MS/MS scans	134022

Table 2. Result filtration parameters.

Peptide -10lgP	≥19.8
Peptide Ascore	≥0
Protein -10lgP	≥20
Proteins unique peptides	≥0
De novo ALC Score	≥50%

Table 3. Statistics of filtered result.

Peptide-Spectrum Matches	39875
Peptide sequences	15316
Protein groups	2014
Proteins	3397
Proteins (#Unique Peptides)	1606 (>2); 554 (=2); 959 (=1);
FDR (Peptide-Spectrum Matches)	0.5%
FDR (Peptide Sequences)	1.1%
De Novo Only Spectra	18027

Name	ΔMass	Position	#PSM	-10lgP	Area	AScore
Deamidation	.98	NQ	8786	79.96	3.7E7	115.60
Oxidation	15.99	M	4441	76.18	9.01E7	1000.00
Acetylation	42.01	N-term	962	65.67		1000.00
Carbamidomethyl	57.02	C	98	66.15	4.72E6	1000.00

3. Experiment Control

Figure 4. Precursor mass error of peptide-spectrum matches (PSM) in filtered result. **(a)** Distribution of precursor mass error in ppm; **(b)** Scatterplot of precursor m/z versus precursor mass error in ppm.

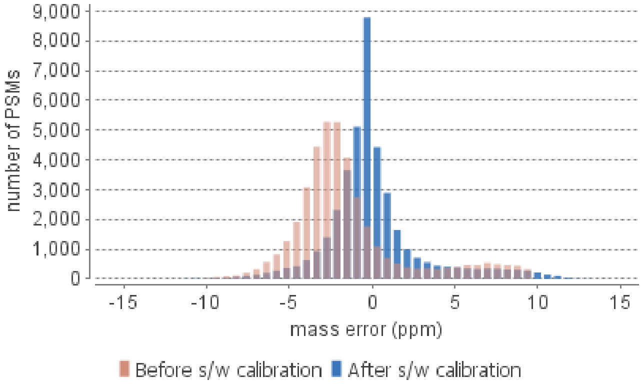

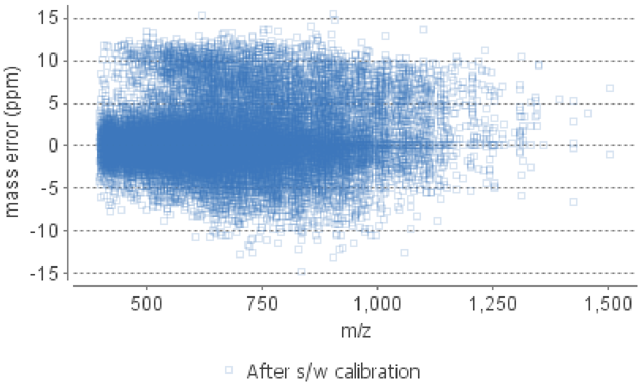

Table 5. Number of identified peptides in each sample by the number of missed cleavages

Missed Cleavages	0	1	2	3	4+
AZ028_01	4533	708	55	0	0
AZ028_02	4168	594	41	0	0
AZ028_03	4519	647	51	0	0

4. Other Information

Table 6. Search parameters.
Search Engine Name: PEAKS
Parent Mass Error Tolerance: 10.0 ppm
Fragment Mass Error Tolerance: 0.05 Da
Precursor Mass Search Type: monoisotopic
Enzyme: Trypsin
Max Missed Cleavages: 2
Non-specific Cleavage: one

Table 7. Instrument parameters.
Fractions: AZ028_01.raw, AZ028_02.raw, AZ028_03.raw
Ion Source: ESI(nano-spray)
Fragmentation Mode: high energy CID (y and b ions)
MS Scan Mode: FT-ICR/Orbitrap
MS/MS Scan Mode: FT-ICR/Orbitrap

Fixed Modifications:
  Carbamidomethylation: 57.02
Variable Modifications:
  Deamidation (NQ): 0.98
  Acetylation (N-term): 42.01
  Oxidation (M): 15.99
Max Variable PTM Per Peptide: 3
Database: PF_all
Taxon: All
Searched Entry: 1941073
FDR Estimation: Enabled
Merge Options: no merge
Precursor Options: corrected
Charge Options: no correction
Filter Options: no filter
Process: true

1. Notes

2. Result Statistics

Figure 1. False discovery rate (FDR) curve. X axis is the number of peptide-spectrum matches (PSM) being kept. Y axis is the corresponding FDR.

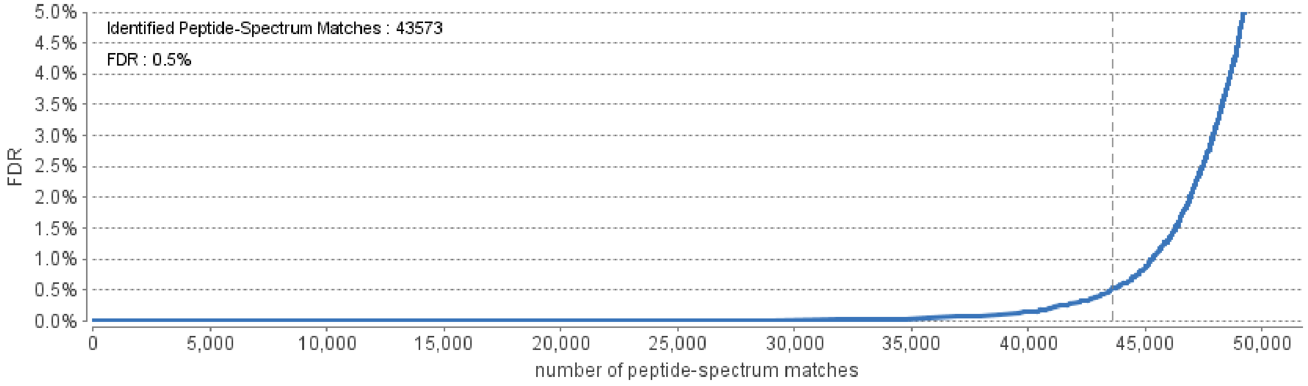

Figure 2. PSM score distribution. **(a)** Distribution of PEAKS peptide score; **(b)** Scatterplot of PEAKS peptide score versus precursor mass error.

(a) (b)

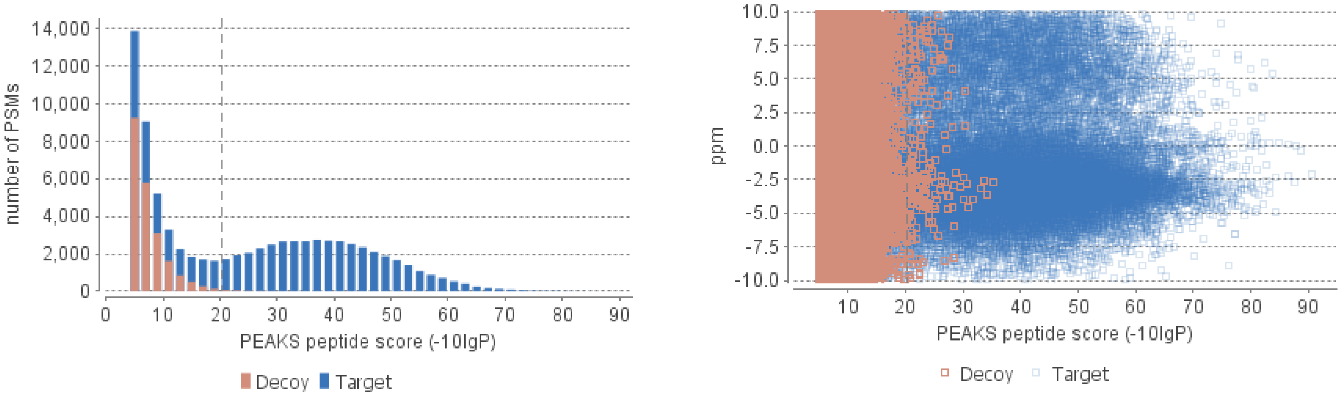

Figure 3. De novo result validation. Distribution of residue local confidence: **(a)** Residues in de novo sequences validated by confident database peptide assignment; **(b)** Residues in "de novo only" sequences.

(a) (b)

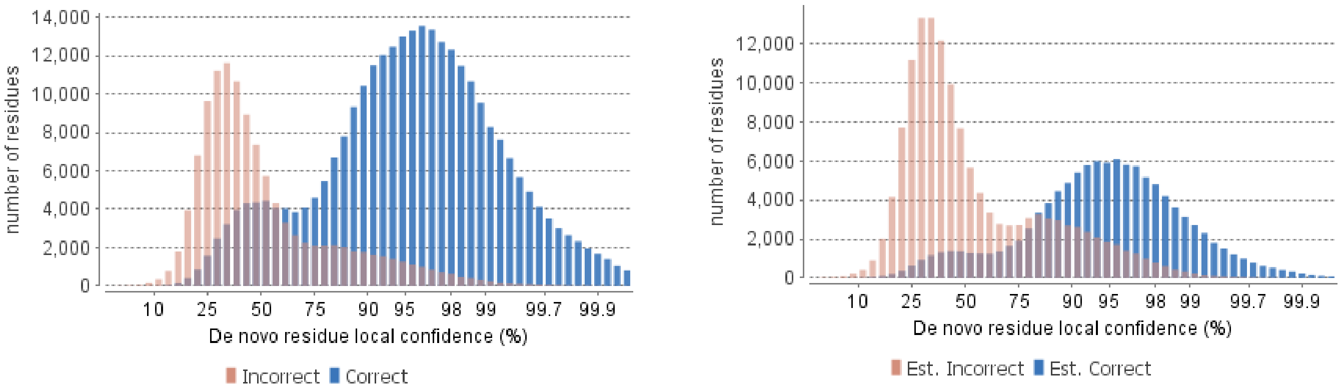

Table 1. Statistics of data.

# of MS scans	24939

Table 4. PTM profile.

Name	ΔMass	Position	#PSM	-10lgP	Area	AScor

# of MS/MS scans 137717

Deamidation	.98	NQ	11152	90.57	2.97E7	32.9[illegible]
Oxidation	15.99	M	5284	81.85		1000.0[illegible]
Acetylation	42.01	N-term	1121	69.02	4.18E6	1000.0[illegible]
Carbamidomethyl	57.02	C	136	61.00	2.22E7	1000.0[illegible]

Table 2. Result filtration parameters.

Peptide -10lgP	≥20.2
Peptide Ascore	≥0
Protein -10lgP	≥20
Proteins unique peptides	≥0
De novo ALC Score	≥50%

Table 3. Statistics of filtered result.

Peptide-Spectrum Matches	43573
Peptide sequences	16495
Protein groups	2045
Proteins	3554
Proteins (#Unique Peptides)	1677 (>2); 399 (=2); 1176 (=1);
FDR (Peptide-Spectrum Matches)	0.5%
FDR (Peptide Sequences)	1.0%
De Novo Only Spectra	20136

3. Experiment Control

Figure 4. Precursor mass error of peptide-spectrum matches (PSM) in filtered result. **(a)** Distribution of precursor mass error in ppm; **(b)** Scatterplot of precursor m/z versus precursor mass error in ppm.

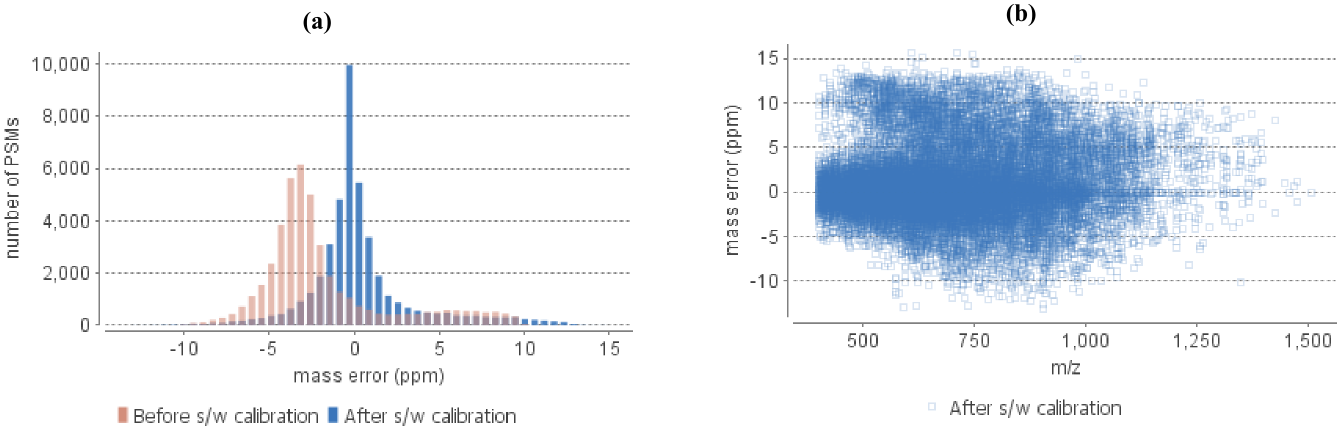

Table 5. Number of identified peptides in each sample by the number of missed cleavages

Missed Cleavages	0	1	2	3	4+
AZ030_01	4986	849	76	0	0
AZ030_02	4438	697	84	0	0
AZ030_03	4612	707	46	0	0

4. Other Information

Table 6. Search parameters.

Search Engine Name: PEAKS
Parent Mass Error Tolerance: 10.0 ppm
Fragment Mass Error Tolerance: 0.05 Da
Precursor Mass Search Type: monoisotopic
Enzyme: Trypsin
Max Missed Cleavages: 2
Non-specific Cleavage: one
Fixed Modifications:

Table 7. Instrument parameters.

Fractions: AZ030_01.raw, AZ030_02.raw, AZ030_03.raw
Ion Source: ESI(nano-spray)
Fragmentation Mode: high energy CID (y and b ions)
MS Scan Mode: FT-ICR/Orbitrap
MS/MS Scan Mode: FT-ICR/Orbitrap

Carbamidomethylation: 57.02
Variable Modifications:
Deamidation (NQ): 0.98
Acetylation (N-term): 42.01
Oxidation (M): 15.99
Max Variable PTM Per Peptide: 3
Database: PF_all
Taxon: All
Searched Entry: 1941073
FDR Estimation: Enabled
Merge Options: no merge
Precursor Options: corrected
Charge Options: no correction
Filter Options: no filter
Process: true

1. Notes

2. Result Statistics

Figure 1. False discovery rate (FDR) curve. X axis is the number of peptide-spectrum matches (PSM) being kept. Y axis is the corresponding FDR.

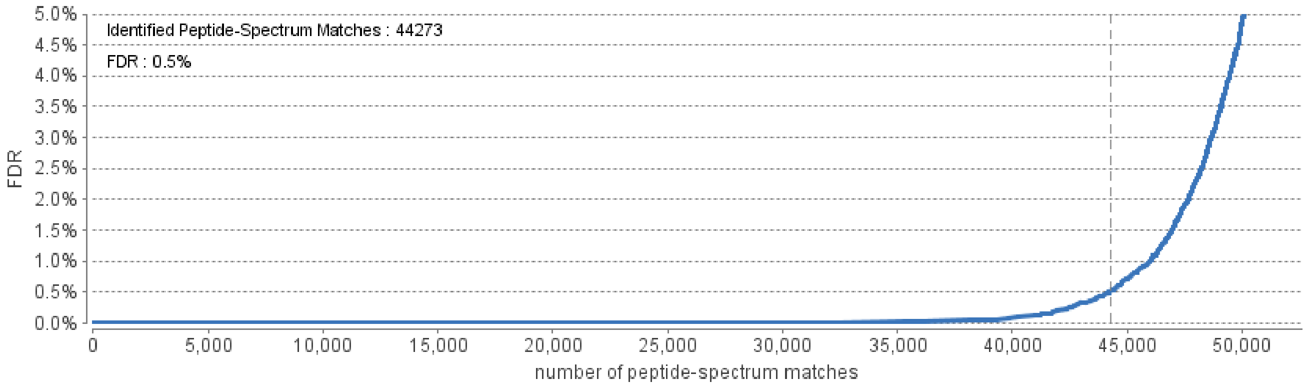

Figure 2. PSM score distribution. **(a)** Distribution of PEAKS peptide score; **(b)** Scatterplot of PEAKS peptide score versus precursor mass error.

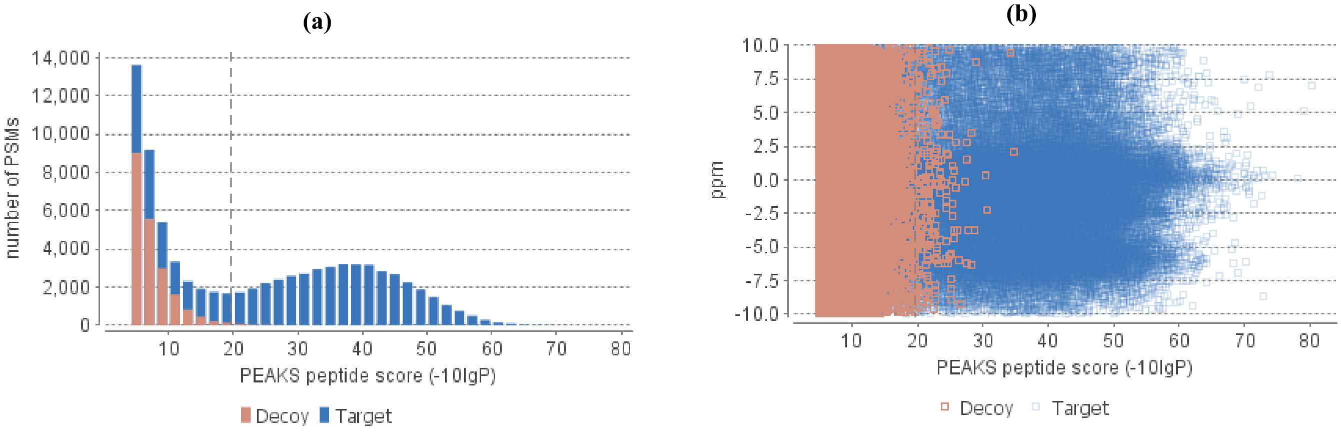

Figure 3. De novo result validation. Distribution of residue local confidence: **(a)** Residues in de novo sequences validated by confident database peptide assignment; **(b)** Residues in "de novo only" sequences.

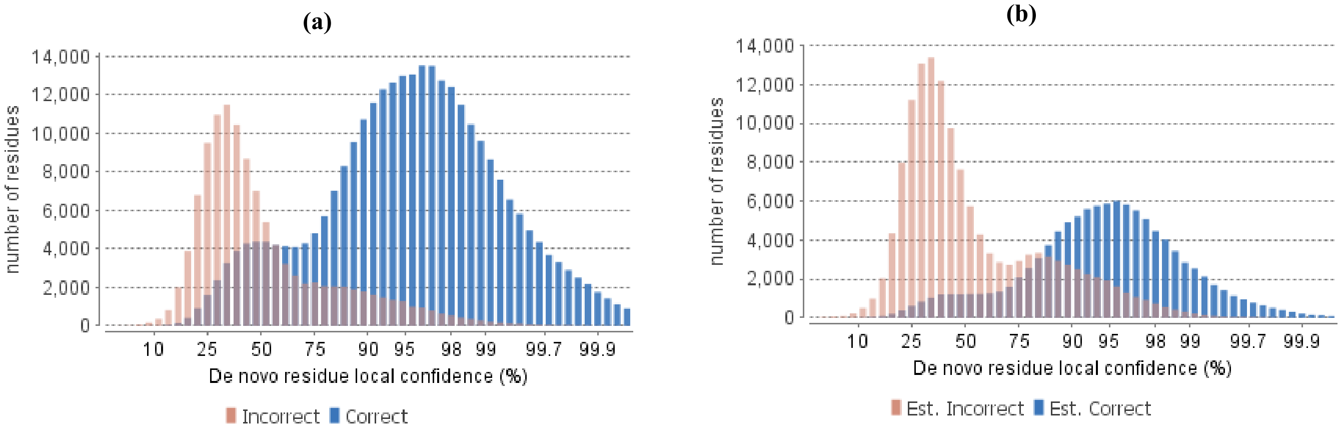

Table 1. Statistics of data.

# of MS scans	24825

Table 4. PTM profile.

Name	ΔMass	Position	#PSM	-10lgP	Area	AScore

# of MS/MS scans 138473

Table 2. Result filtration parameters.

Peptide -10lgP	≥19.6
Peptide Ascore	≥0
Protein -10lgP	≥20
Proteins unique peptides	≥0
De novo ALC Score	≥50%

Table 3. Statistics of filtered result.

Peptide-Spectrum Matches	44273
Peptide sequences	16230
Protein groups	2028
Proteins	3410
Proteins (#Unique Peptides)	1556 (>2); 428 (=2); 1109 (=1);
FDR (Peptide-Spectrum Matches)	0.5%
FDR (Peptide Sequences)	1.1%
De Novo Only Spectra	19187

Deamidation	.98	NQ	10285	80.22		76.1[illegible]
Oxidation	15.99	M	4207	78.97		1000.0[illegible]
Acetylation	42.01	N-term	1185	58.14	9.78E6	1000.0[illegible]
Carbamidomethyl	57.02	C	250	62.27	4.39E6	1000.0[illegible]

3. Experiment Control

Figure 4. Precursor mass error of peptide-spectrum matches (PSM) in filtered result. **(a)** Distribution of precursor mass error in ppm; **(b)** Scatterplot of precursor m/z versus precursor mass error in ppm.

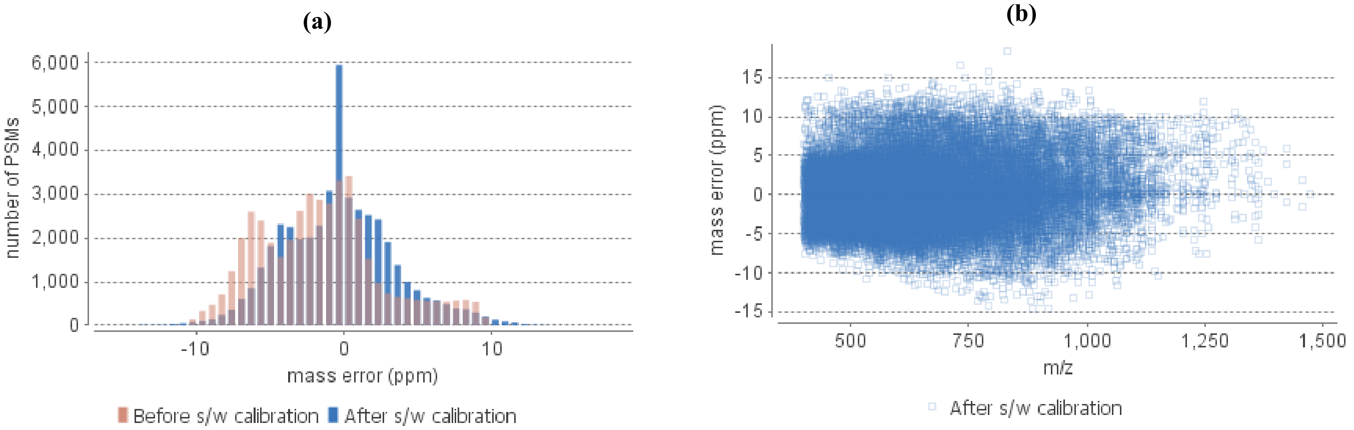

Table 5. Number of identified peptides in each sample by the number of missed cleavages

Missed Cleavages	0	1	2	3	4+
AZ031_01	5332	916	102	0	0
AZ031_02	3683	719	62	0	0
AZ031_03	4542	796	78	0	0

4. Other Information

Table 6. Search parameters.

Search Engine Name: PEAKS
Parent Mass Error Tolerance: 10.0 ppm
Fragment Mass Error Tolerance: 0.05 Da
Precursor Mass Search Type: monoisotopic
Enzyme: Trypsin
Max Missed Cleavages: 2
Non-specific Cleavage: one
Fixed Modifications:

Table 7. Instrument parameters.

Fractions: AZ031_01.raw, AZ031_02.raw, AZ031_03.raw
Ion Source: ESI(nano-spray)
Fragmentation Mode: high energy CID (y and b ions)
MS Scan Mode: FT-ICR/Orbitrap
MS/MS Scan Mode: FT-ICR/Orbitrap

  Carbamidomethylation: 57.02
Variable Modifications:
  Deamidation (NQ): 0.98
  Acetylation (N-term): 42.01
  Oxidation (M): 15.99
Max Variable PTM Per Peptide: 3
Database: PF_all
Taxon: All
Searched Entry: 1941073
FDR Estimation: Enabled
Merge Options: no merge
Precursor Options: corrected
Charge Options: no correction
Filter Options: no filter
Process: true

1. Notes

2. Result Statistics

Figure 1. False discovery rate (FDR) curve. X axis is the number of peptide-spectrum matches (PSM) being kept. Y axis is the corresponding FDR.

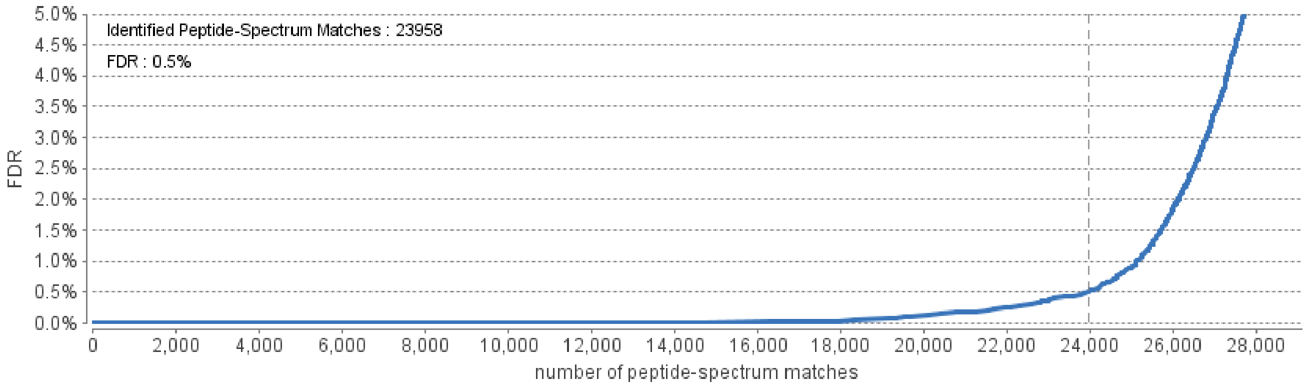

Figure 2. PSM score distribution. **(a)** Distribution of PEAKS peptide score; **(b)** Scatterplot of PEAKS peptide score versus precursor mass error.

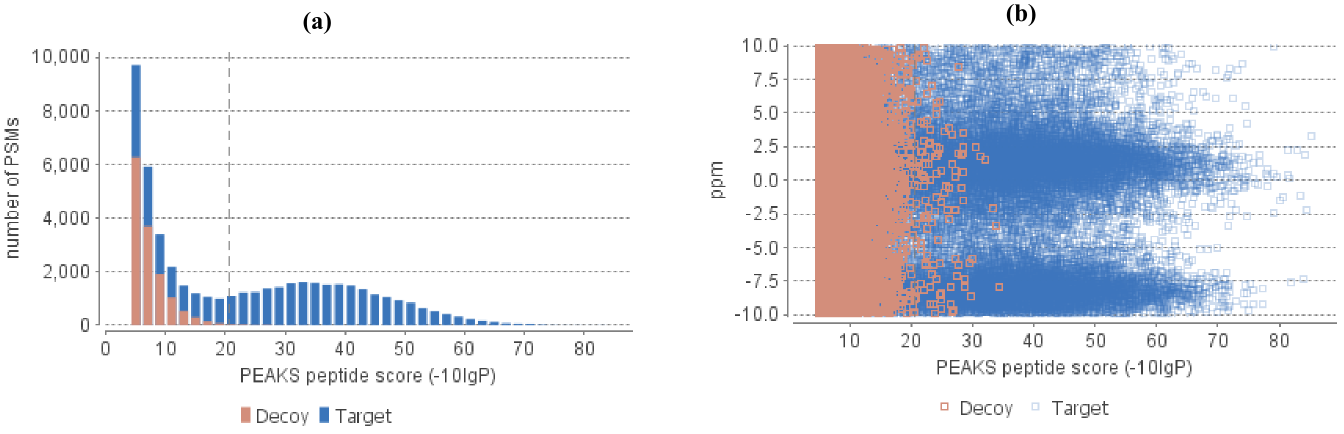

Figure 3. De novo result validation. Distribution of residue local confidence: **(a)** Residues in de novo sequences validated by confident database peptide assignment; **(b)** Residues in "de novo only" sequences.

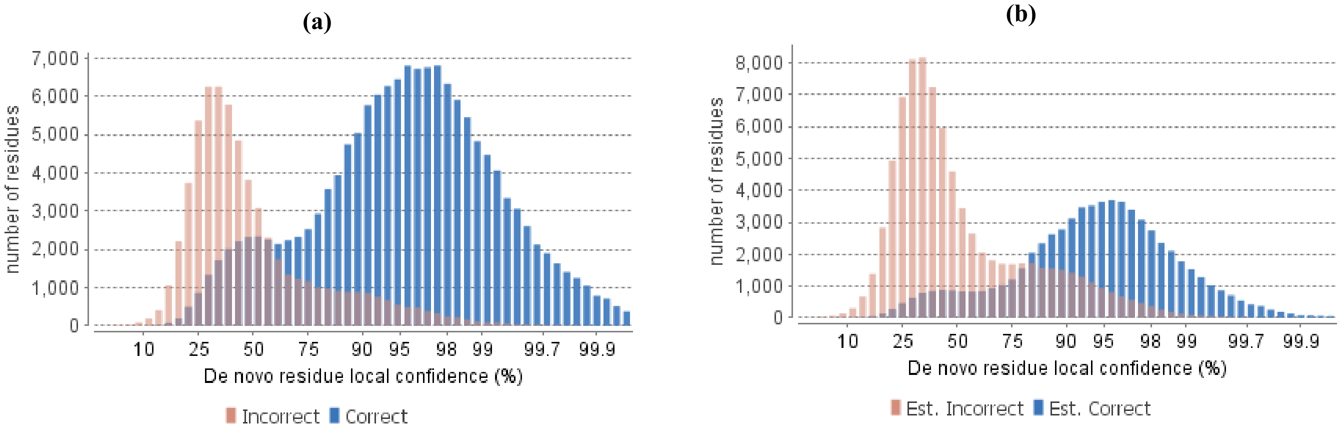

Table 1. Statistics of data.

# of MS scans	17757

Table 4. PTM profile.

Name	ΔMass	Position	#PSM	-10lgP	Area	AScore

# of MS/MS scans 87954

Deamidation	.98	NQ	5460	85.18	3.29E7	105.1
Oxidation	15.99	M	1828	84.13	4.52E7	1000.0
Acetylation	42.01	N-term	469	64.53		1000.0
Carbamidomethyl	57.02	C	84	70.26	2.27E6	1000.0

Table 2. Result filtration parameters.

Peptide -10lgP	≥20.6
Peptide Ascore	≥0
Protein -10lgP	≥20
Proteins unique peptides	≥0
De novo ALC Score	≥50%

Table 3. Statistics of filtered result.

Peptide-Spectrum Matches	23958
Peptide sequences	12045
Protein groups	1786
Proteins	3167
Proteins (#Unique Peptides)	1411 (>2); 393 (=2); 1081 (=1);
FDR (Peptide-Spectrum Matches)	0.5%
FDR (Peptide Sequences)	0.8%
De Novo Only Spectra	12330

3. Experiment Control

Figure 4. Precursor mass error of peptide-spectrum matches (PSM) in filtered result. **(a)** Distribution of precursor mass error in ppm; **(b)** Scatterplot of precursor m/z versus precursor mass error in ppm.

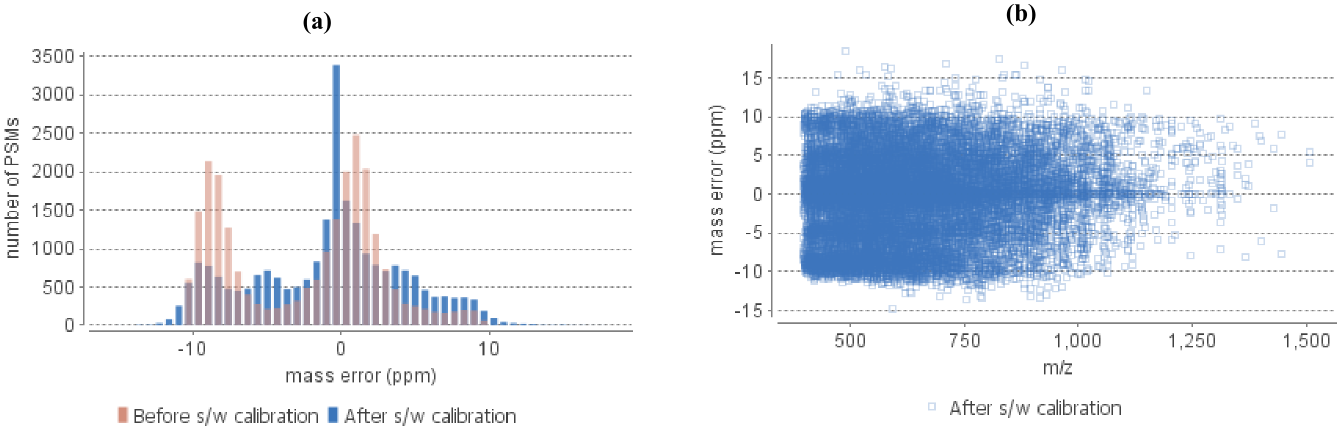

Table 5. Number of identified peptides in each sample by the number of missed cleavages

Missed Cleavages	0	1	2	3	4+
AZ038_01	4807	687	52	0	0
AZ038_03	5631	826	42	0	0

4. Other Information

Table 6. Search parameters.

Search Engine Name: PEAKS
Parent Mass Error Tolerance: 10.0 ppm
Fragment Mass Error Tolerance: 0.05 Da
Precursor Mass Search Type: monoisotopic
Enzyme: Trypsin
Max Missed Cleavages: 2
Non-specific Cleavage: one
Fixed Modifications:
  Carbamidomethylation: 57.02
Variable Modifications:

Table 7. Instrument parameters.

Fractions: AZ038_01.raw, AZ038_02.raw, AZ038_03.raw
Ion Source: ESI(nano-spray)
Fragmentation Mode: high energy CID (y and b ions)
MS Scan Mode: FT-ICR/Orbitrap
MS/MS Scan Mode: FT-ICR/Orbitrap

Deamidation (NQ): 0.98
Acetylation (N-term): 42.01
Oxidation (M): 15.99

Max Variable PTM Per Peptide: 3
Database: PF_all
Taxon: All
Searched Entry: 1941073
FDR Estimation: Enabled
Merge Options: no merge
Precursor Options: corrected
Charge Options: no correction
Filter Options: no filter
Process: true

1. Notes

2. Result Statistics

Figure 1. False discovery rate (FDR) curve. X axis is the number of peptide-spectrum matches (PSM) being kept. Y axis is the corresponding FDR.

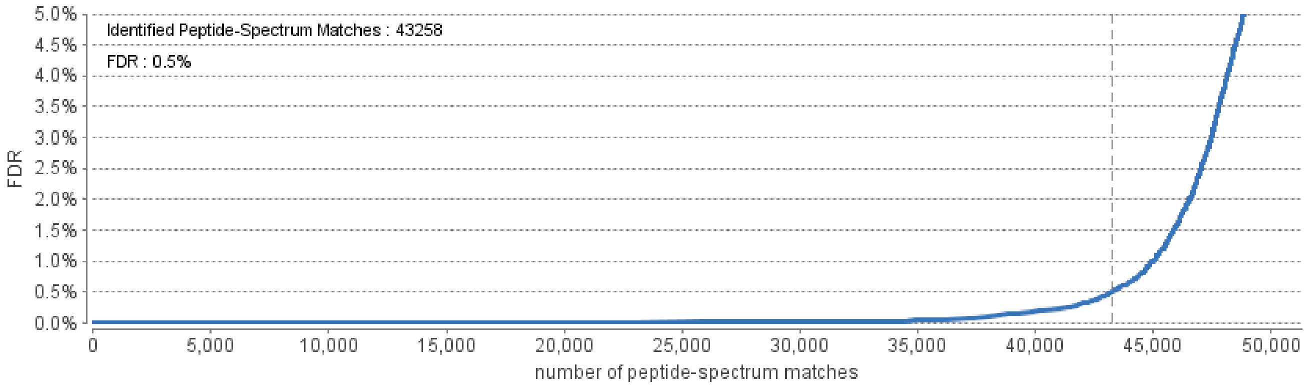

Figure 2. PSM score distribution. **(a)** Distribution of PEAKS peptide score; **(b)** Scatterplot of PEAKS peptide score versus precursor mass error.

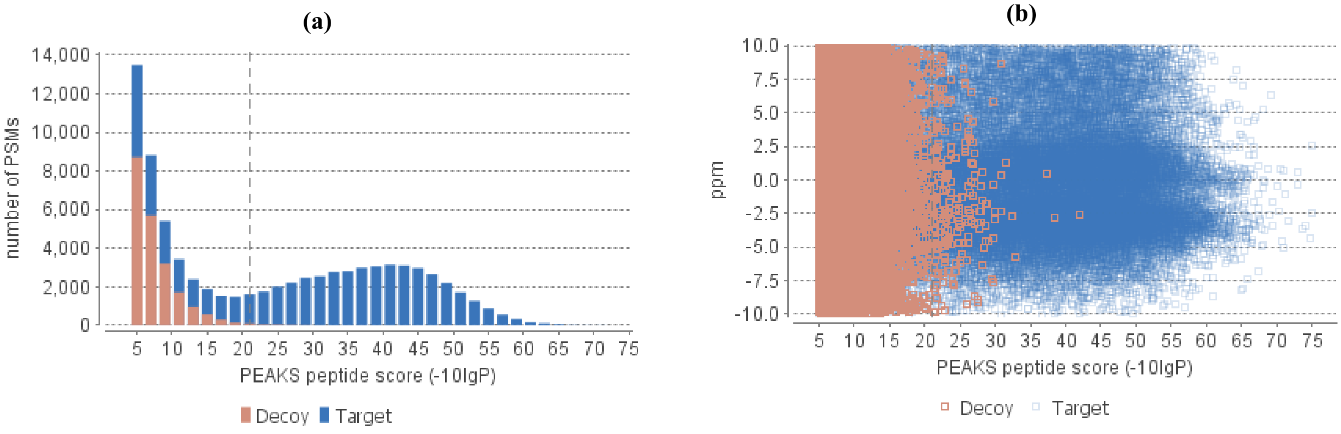

Figure 3. De novo result validation. Distribution of residue local confidence: **(a)** Residues in de novo sequences validated by confident database peptide assignment; **(b)** Residues in "de novo only" sequences.

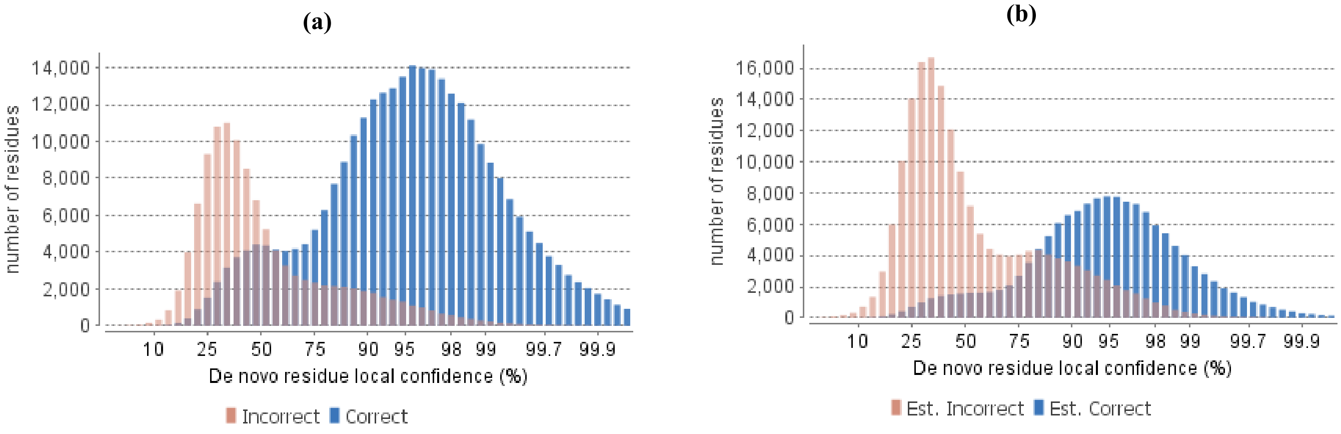

Table 1. Statistics of data.

# of MS scans	24358

Table 4. PTM profile.

Name	ΔMass	Position	#PSM	-10lgP	Area	AScore

# of MS/MS scans 140011

Table 2. Result filtration parameters.

Peptide -10lgP	≥21
Peptide Ascore	≥0
Protein -10lgP	≥20
Proteins unique peptides	≥0
De novo ALC Score	≥50%

Table 3. Statistics of filtered result.

Peptide-Spectrum Matches	43258
Peptide sequences	16058
Protein groups	2177
Proteins	3622
Proteins (#Unique Peptides)	1630 (>2); 487 (=2); 1181 (=1);
FDR (Peptide-Spectrum Matches)	0.5%
FDR (Peptide Sequences)	1.0%
De Novo Only Spectra	24453

Deamidation	.98	NQ	9737	74.94	4.29E7	32.9[illegible]
Oxidation	15.99	M	4212	70.74	1.55E8	1000.0[illegible]
Acetylation	42.01	N-term	1174	64.60	4.43E6	1000.0[illegible]
Carbamidomethyl	57.02	C	195	55.07		1000.0[illegible]

3. Experiment Control

Figure 4. Precursor mass error of peptide-spectrum matches (PSM) in filtered result. **(a)** Distribution of precursor mass error in ppm; **(b)** Scatterplot of precursor m/z versus precursor mass error in ppm.

(a) (b)

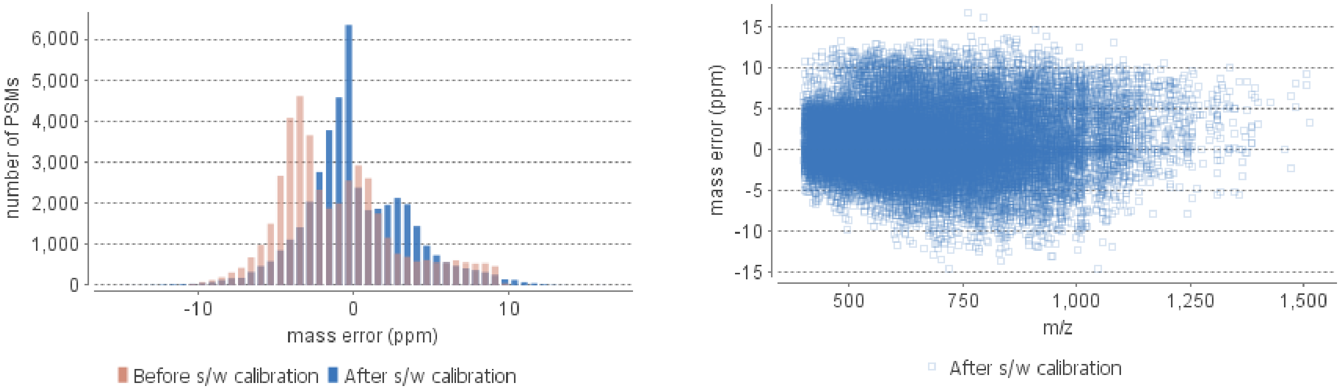

Table 5. Number of identified peptides in each sample by the number of missed cleavages

Missed Cleavages	0	1	2	3	4+
AZ040_01	4240	799	65	0	0
AZ040_02	4191	653	59	0	0
AZ040_03	5193	785	73	0	0

4. Other Information

Table 6. Search parameters.

Search Engine Name: PEAKS
Parent Mass Error Tolerance: 10.0 ppm
Fragment Mass Error Tolerance: 0.05 Da
Precursor Mass Search Type: monoisotopic
Enzyme: Trypsin
Max Missed Cleavages: 2
Non-specific Cleavage: one
Fixed Modifications:

Table 7. Instrument parameters.

Fractions: AZ040_01.raw, AZ040_02.raw, AZ040_03.raw
Ion Source: ESI(nano-spray)
Fragmentation Mode: high energy CID (y and b ions)
MS Scan Mode: FT-ICR/Orbitrap
MS/MS Scan Mode: FT-ICR/Orbitrap

Carbamidomethylation: 57.02
Variable Modifications:
Deamidation (NQ): 0.98
Acetylation (N-term): 42.01
Oxidation (M): 15.99
Max Variable PTM Per Peptide: 3
Database: PF_all
Taxon: All
Searched Entry: 1941073
FDR Estimation: Enabled
Merge Options: no merge
Precursor Options: corrected
Charge Options: no correction
Filter Options: no filter
Process: true

1. Notes

2. Result Statistics

Figure 1. False discovery rate (FDR) curve. X axis is the number of peptide-spectrum matches (PSM) being kept. Y axis is the corresponding FDR.

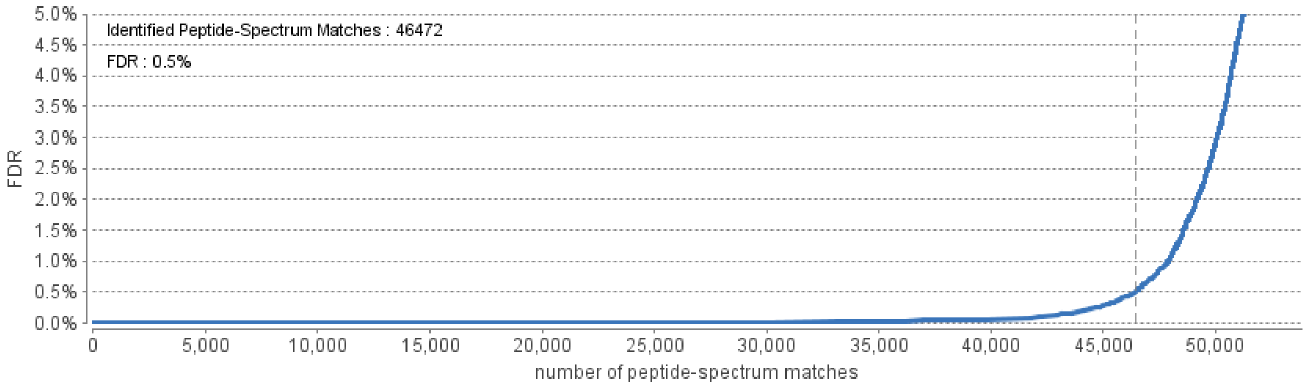

Figure 2. PSM score distribution. **(a)** Distribution of PEAKS peptide score; **(b)** Scatterplot of PEAKS peptide score versus precursor mass error.

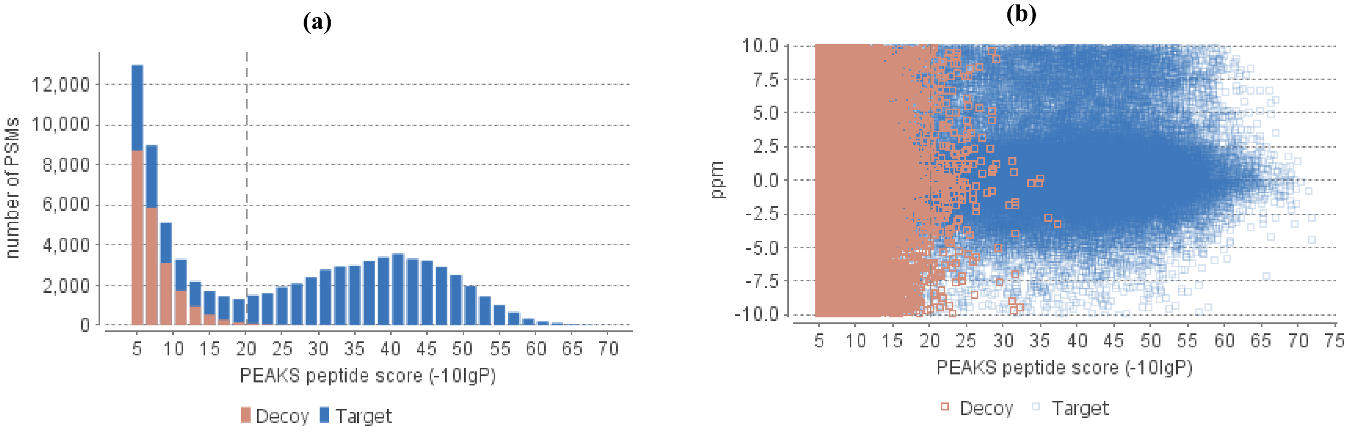

Figure 3. De novo result validation. Distribution of residue local confidence: **(a)** Residues in de novo sequences validated by confident database peptide assignment; **(b)** Residues in "de novo only" sequences.

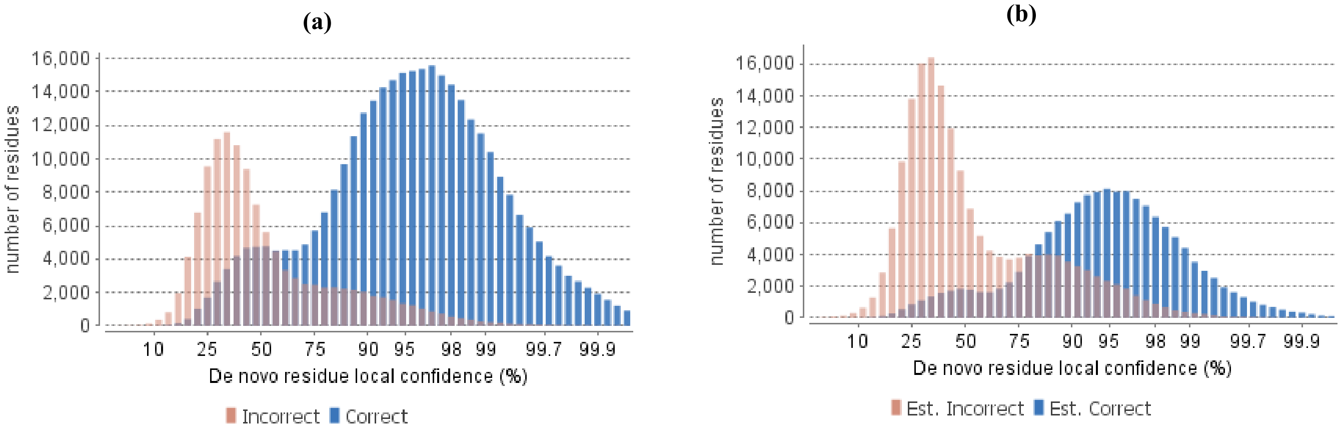

Table 1. Statistics of data.

# of MS scans	24204

Table 4. PTM profile.

Name	ΔMass	Position	#PSM	-10lgP	Area	AScore

# of MS/MS scans 140846

Table 2. Result filtration parameters.

Peptide -10lgP	≥20.2
Peptide Ascore	≥0
Protein -10lgP	≥20
Proteins unique peptides	≥0
De novo ALC Score	≥50%

Table 3. Statistics of filtered result.

Peptide-Spectrum Matches	46472
Peptide sequences	17512
Protein groups	2142
Proteins	3579
Proteins (#Unique Peptides)	1758 (>2); 402 (=2); 1117 (=1);
FDR (Peptide-Spectrum Matches)	0.5%
FDR (Peptide Sequences)	1.0%
De Novo Only Spectra	24171

Deamidation	.98	NQ	10637	69.67	4.47E7	8.6[illegible]
Oxidation	15.99	M	4476	69.17	2.02E8	1000.0[illegible]
Acetylation	42.01	N-term	1270	63.12	3.4E6	1000.0[illegible]
Carbamidomethyl	57.02	C	255	60.34	1.09E7	1000.0[illegible]

3. Experiment Control

Figure 4. Precursor mass error of peptide-spectrum matches (PSM) in filtered result. **(a)** Distribution of precursor mass error in ppm; **(b)** Scatterplot of precursor m/z versus precursor mass error in ppm.

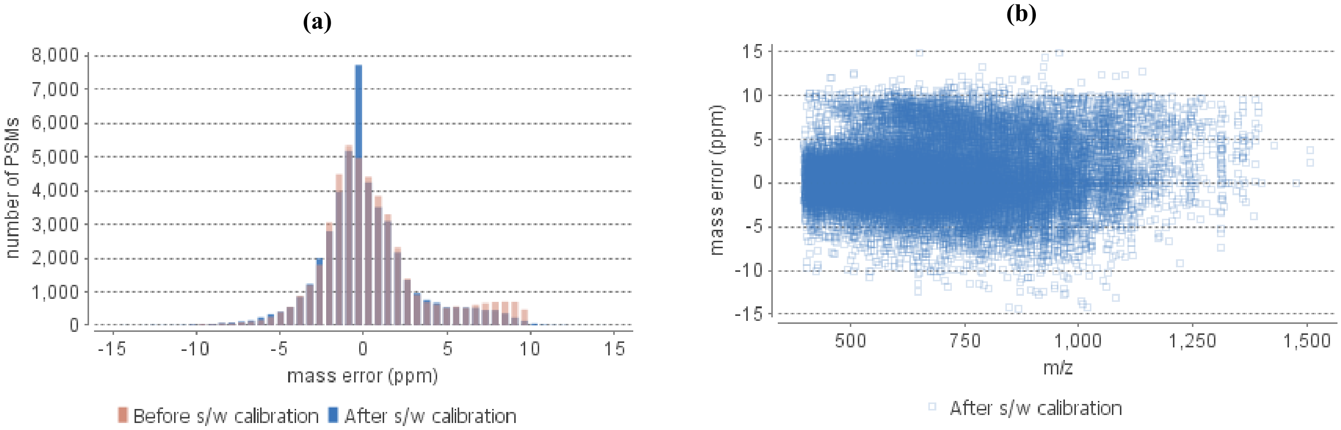

Table 5. Number of identified peptides in each sample by the number of missed cleavages

Missed Cleavages	0	1	2	3	4+
AZ042_01	4966	1027	98	0	0
AZ042_02	4903	851	98	0	0
AZ042_03	4592	890	87	0	0

4. Other Information

Table 6. Search parameters.

Search Engine Name: PEAKS
Parent Mass Error Tolerance: 10.0 ppm
Fragment Mass Error Tolerance: 0.05 Da
Precursor Mass Search Type: monoisotopic
Enzyme: Trypsin
Max Missed Cleavages: 2
Non-specific Cleavage: one
Fixed Modifications:

Table 7. Instrument parameters.

Fractions: AZ042_01.raw, AZ042_02.raw, AZ042_03.raw
Ion Source: ESI(nano-spray)
Fragmentation Mode: high energy CID (y and b ions)
MS Scan Mode: FT-ICR/Orbitrap
MS/MS Scan Mode: FT-ICR/Orbitrap

Carbamidomethylation: 57.02
Variable Modifications:
Deamidation (NQ): 0.98
Acetylation (N-term): 42.01
Oxidation (M): 15.99
Max Variable PTM Per Peptide: 3
Database: PF_all
Taxon: All
Searched Entry: 1941073
FDR Estimation: Enabled
Merge Options: no merge
Precursor Options: corrected
Charge Options: no correction
Filter Options: no filter
Process: true

1. Notes

2. Result Statistics

Figure 1. False discovery rate (FDR) curve. X axis is the number of peptide-spectrum matches (PSM) being kept. Y axis is the corresponding FDR.

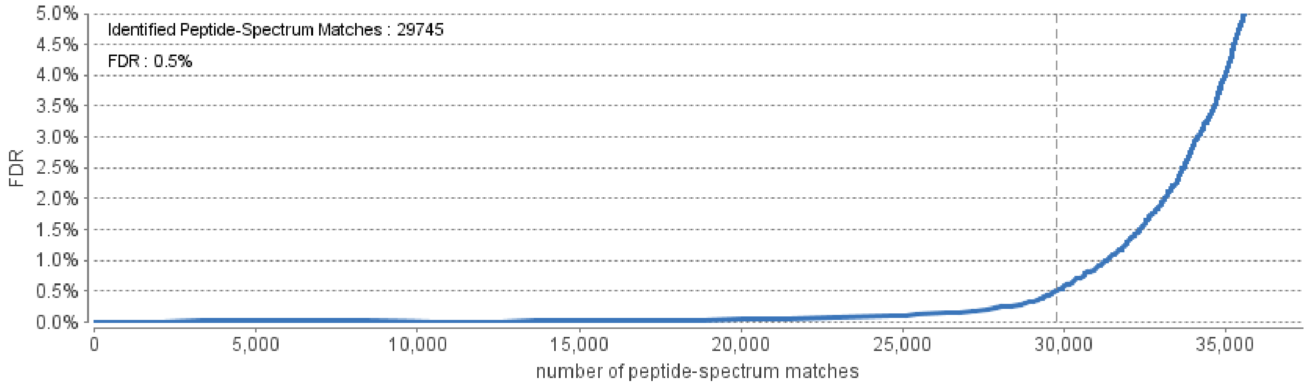

Figure 2. PSM score distribution. **(a)** Distribution of PEAKS peptide score; **(b)** Scatterplot of PEAKS peptide score versus precursor mass error.

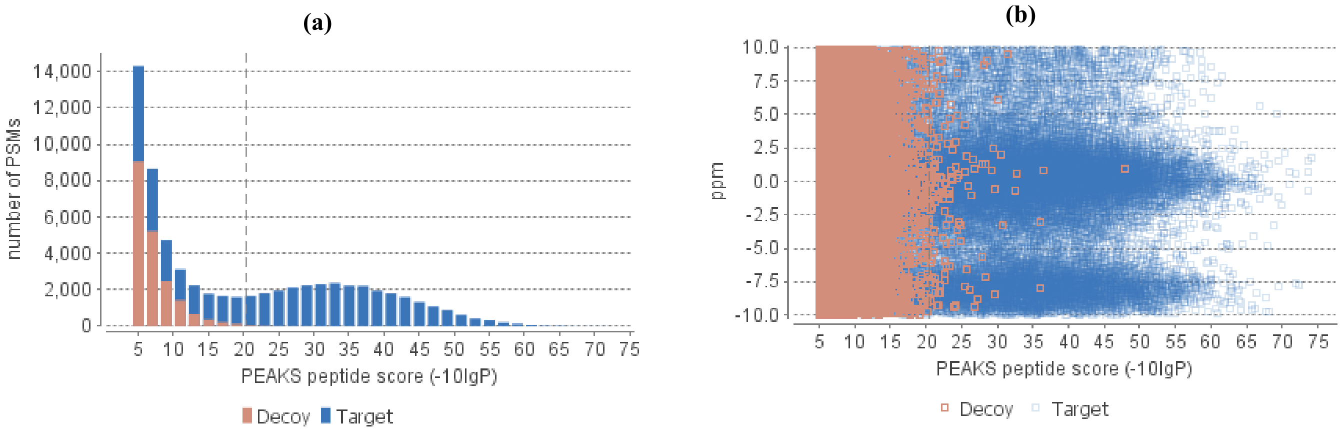

Figure 3. De novo result validation. Distribution of residue local confidence: **(a)** Residues in de novo sequences validated by confident database peptide assignment; **(b)** Residues in "de novo only" sequences.

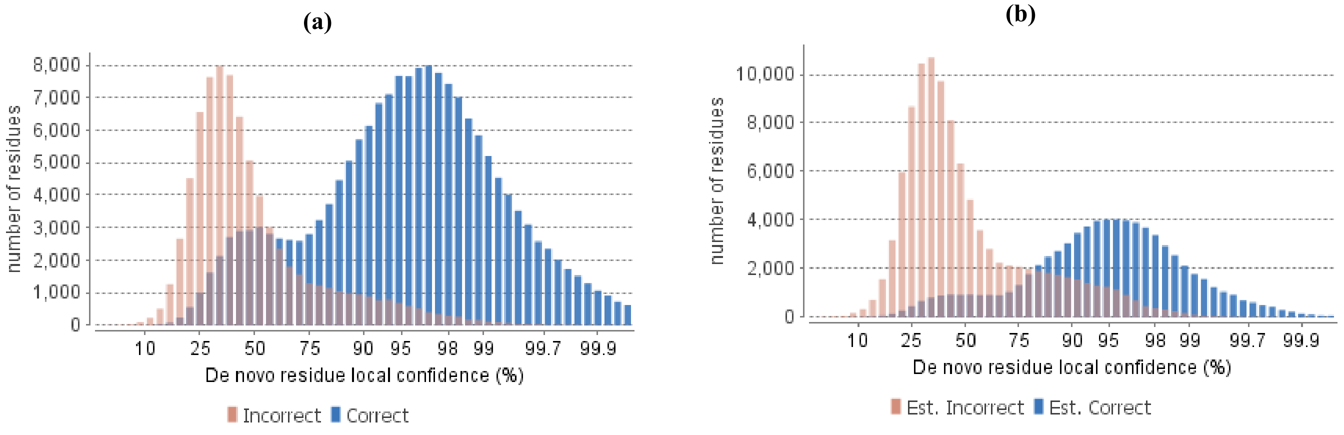

Table 1. Statistics of data.

# of MS scans	28120

Table 4. PTM profile.

Name	ΔMass	Position	#PSM	-10lgP	Area	AScor[illegible]

# of MS/MS scans 125892

Deamidation	.98	NQ	5889	72.36	2.58E7	29.3[illegible]
Oxidation	15.99	M	2476	69.45	6.66E6	1000.0[illegible]
Acetylation	42.01	N-term	630	55.18		1000.0[illegible]
Carbamidomethyl	57.02	C	83	58.65	4.4E6	1000.0[illegible]

Table 2. Result filtration parameters.

Peptide -10lgP	≥20.4
Peptide Ascore	≥0
Protein -10lgP	≥20
Proteins unique peptides	≥0
De novo ALC Score	≥50%

Table 3. Statistics of filtered result.

Peptide-Spectrum Matches	29745
Peptide sequences	11188
Protein groups	1728
Proteins	3303
Proteins (#Unique Peptides)	1330 (>2); 332 (=2); 1370 (=1);
FDR (Peptide-Spectrum Matches)	0.5%
FDR (Peptide Sequences)	1.0%
De Novo Only Spectra	15386

3. Experiment Control

Figure 4. Precursor mass error of peptide-spectrum matches (PSM) in filtered result. **(a)** Distribution of precursor mass error in ppm; **(b)** Scatterplot of precursor m/z versus precursor mass error in ppm.

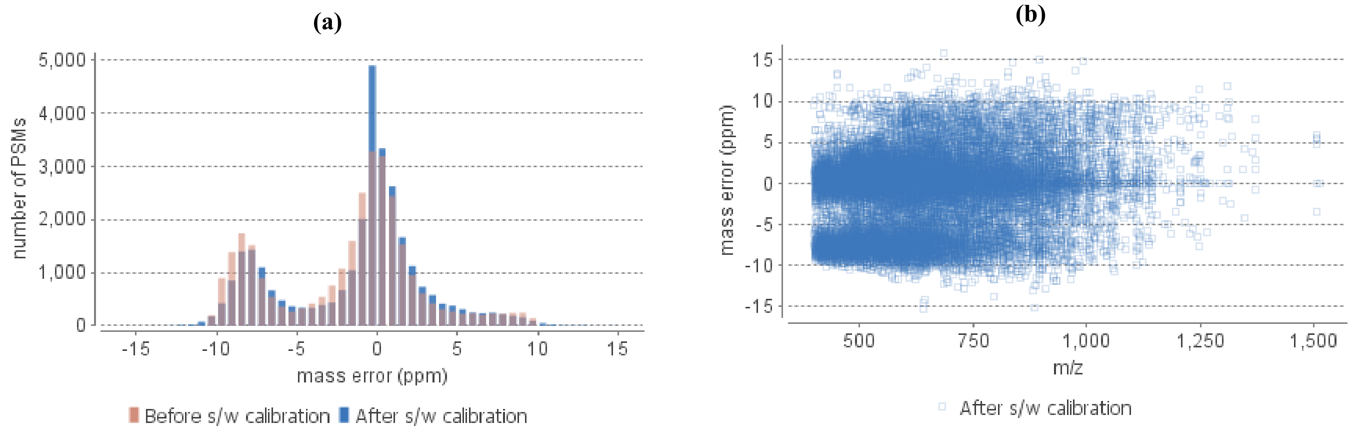

Table 5. Number of identified peptides in each sample by the number of missed cleavages

Missed Cleavages	0	1	2	3	4+
AZ043_01	3114	402	25	0	0
AZ043_02	3479	491	36	0	0
AZ043_03	3115	475	51	0	0

4. Other Information

Table 6. Search parameters.

Search Engine Name: PEAKS
Parent Mass Error Tolerance: 10.0 ppm
Fragment Mass Error Tolerance: 0.05 Da
Precursor Mass Search Type: monoisotopic
Enzyme: Trypsin
Max Missed Cleavages: 2
Non-specific Cleavage: one
Fixed Modifications:

Table 7. Instrument parameters.

Fractions: AZ043_01.raw, AZ043_02.raw, AZ043_03.raw
Ion Source: ESI(nano-spray)
Fragmentation Mode: high energy CID (y and b ions)
MS Scan Mode: FT-ICR/Orbitrap
MS/MS Scan Mode: FT-ICR/Orbitrap

Carbamidomethylation: 57.02
Variable Modifications:
Deamidation (NQ): 0.98
Acetylation (N-term): 42.01
Oxidation (M): 15.99
Max Variable PTM Per Peptide: 3
Database: PF_all
Taxon: All
Searched Entry: 1941073
FDR Estimation: Enabled
Merge Options: no merge
Precursor Options: corrected
Charge Options: no correction
Filter Options: no filter
Process: true

1. Notes

2. Result Statistics

Figure 1. False discovery rate (FDR) curve. X axis is the number of peptide-spectrum matches (PSM) being kept. Y axis is the corresponding FDR.

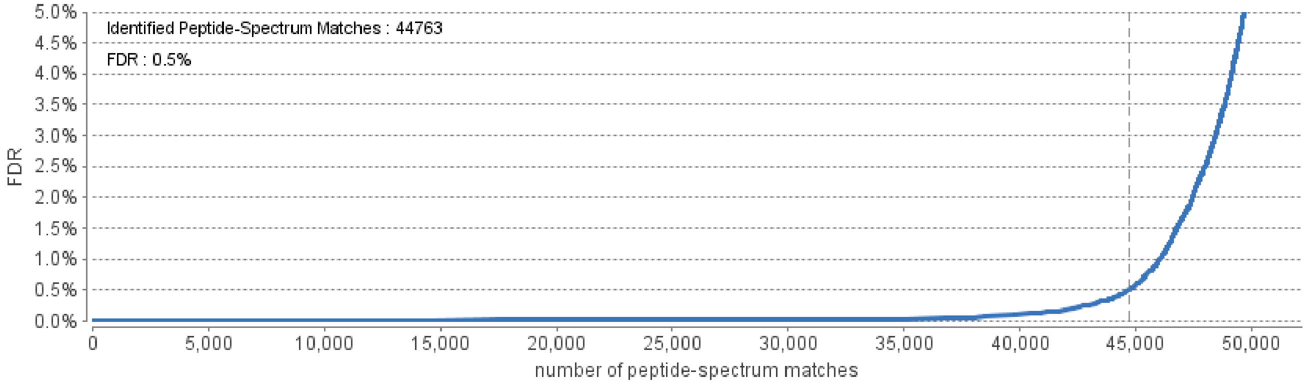

Figure 2. PSM score distribution. **(a)** Distribution of PEAKS peptide score; **(b)** Scatterplot of PEAKS peptide score versus precursor mass error.

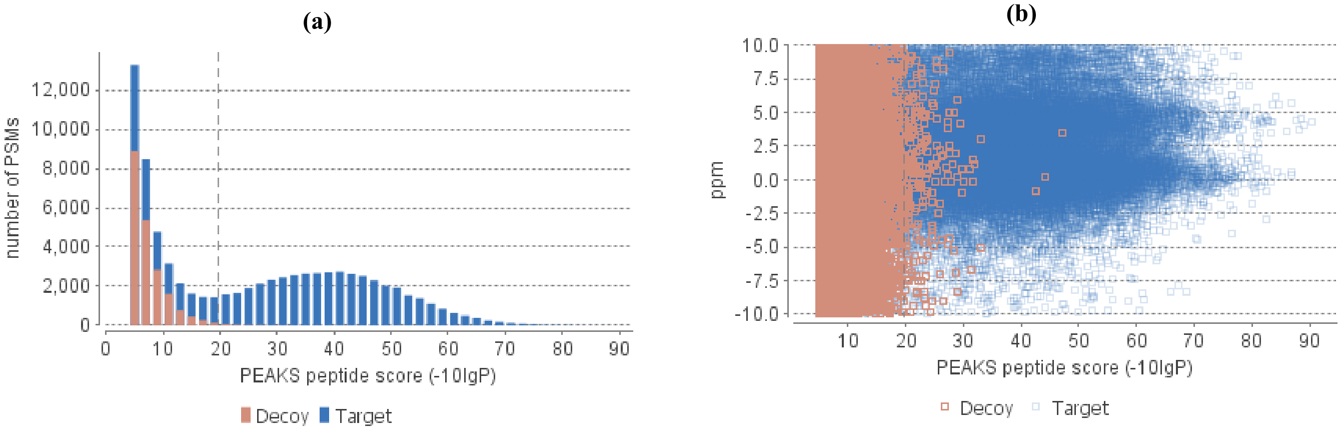

Figure 3. De novo result validation. Distribution of residue local confidence: **(a)** Residues in de novo sequences validated by confident database peptide assignment; **(b)** Residues in "de novo only" sequences.

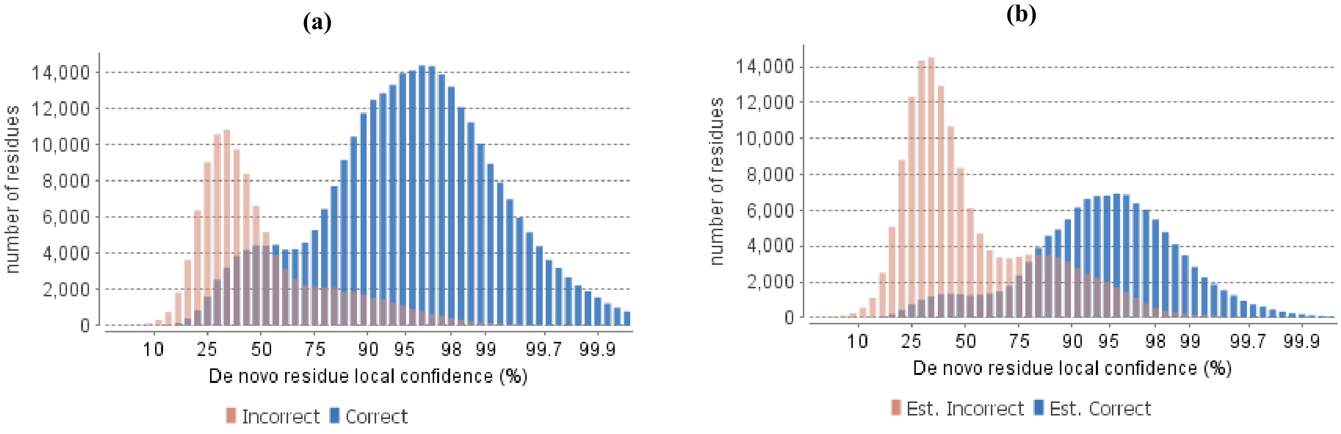

Table 1. Statistics of data.

# of MS scans	24989

Table 4. PTM profile.

Name	ΔMass	Position	#PSM	-10lgP	Area	AScore

# of MS/MS scans 138026

Deamidation	.98	NQ	8273	90.34	5.66E7	32.97
Oxidation	15.99	M	4406	86.90	6.64E6	1000.0(
Acetylation	42.01	N-term	1046	68.97		1000.0(
Carbamidomethyl	57.02	C	159	54.39		1000.0(

Table 2. Result filtration parameters.

Peptide -10lgP	≥19.8
Peptide Ascore	≥0
Protein -10lgP	≥20
Proteins unique peptides	≥0
De novo ALC Score	≥50%

Table 3. Statistics of filtered result.

Peptide-Spectrum Matches	44763
Peptide sequences	16981
Protein groups	2119
Proteins	3614
Proteins (#Unique Peptides)	1800 (>2); 412 (=2); 1146 (=1);
FDR (Peptide-Spectrum Matches)	0.5%
FDR (Peptide Sequences)	1.1%
De Novo Only Spectra	21255

3. Experiment Control

Figure 4. Precursor mass error of peptide-spectrum matches (PSM) in filtered result. **(a)** Distribution of precursor mass error in ppm; **(b)** Scatterplot of precursor m/z versus precursor mass error in ppm.

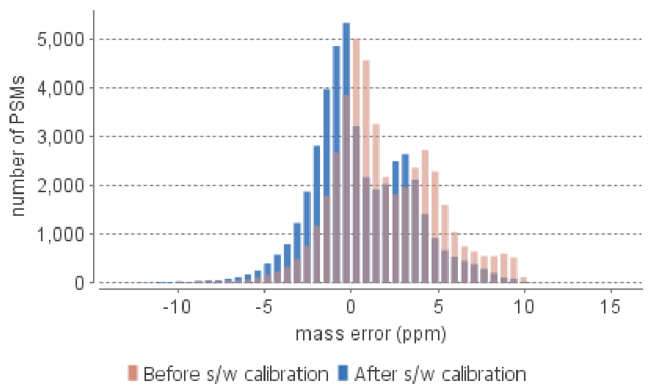

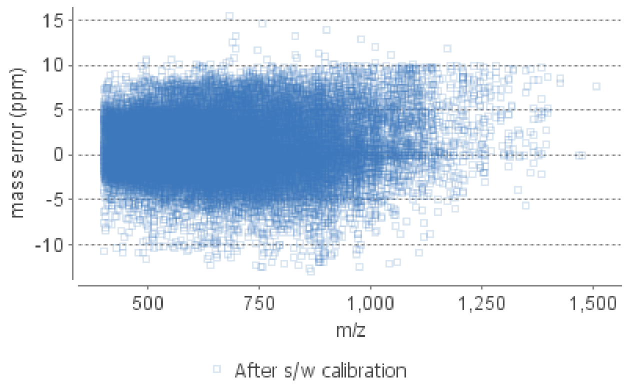

Table 5. Number of identified peptides in each sample by the number of missed cleavages

Missed Cleavages	0	1	2	3	4+
AZ044_01	5247	915	105	0	0
AZ044_02	4720	872	78	0	0
AZ044_03	4178	788	78	0	0

4. Other Information

Table 6. Search parameters.

Search Engine Name: PEAKS
Parent Mass Error Tolerance: 10.0 ppm
Fragment Mass Error Tolerance: 0.05 Da
Precursor Mass Search Type: monoisotopic
Enzyme: Trypsin
Max Missed Cleavages: 2
Non-specific Cleavage: one
Fixed Modifications:

Table 7. Instrument parameters.

Fractions: AZ044_01.raw, AZ044_02.raw, AZ044_03.raw
Ion Source: ESI(nano-spray)
Fragmentation Mode: high energy CID (y and b ions)
MS Scan Mode: FT-ICR/Orbitrap
MS/MS Scan Mode: FT-ICR/Orbitrap

Carbamidomethylation: 57.02
Variable Modifications:
Deamidation (NQ): 0.98
Acetylation (N-term): 42.01
Oxidation (M): 15.99
Max Variable PTM Per Peptide: 3
Database: PF_all
Taxon: All
Searched Entry: 1941073
FDR Estimation: Enabled
Merge Options: no merge
Precursor Options: corrected
Charge Options: no correction
Filter Options: no filter
Process: true

1. Notes

2. Result Statistics

Figure 1. False discovery rate (FDR) curve. X axis is the number of peptide-spectrum matches (PSM) being kept. Y axis is the corresponding FDR.

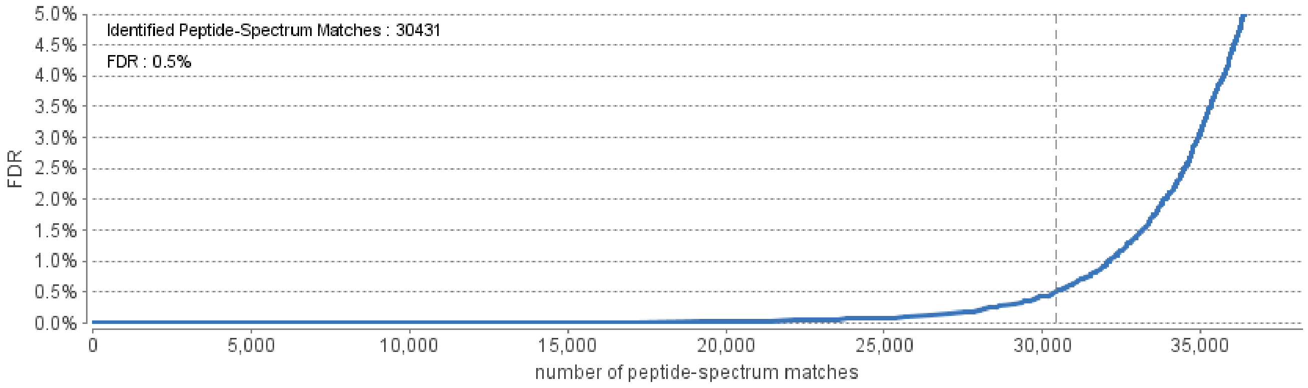

Figure 2. PSM score distribution. **(a)** Distribution of PEAKS peptide score; **(b)** Scatterplot of PEAKS peptide score versus precursor mass error.

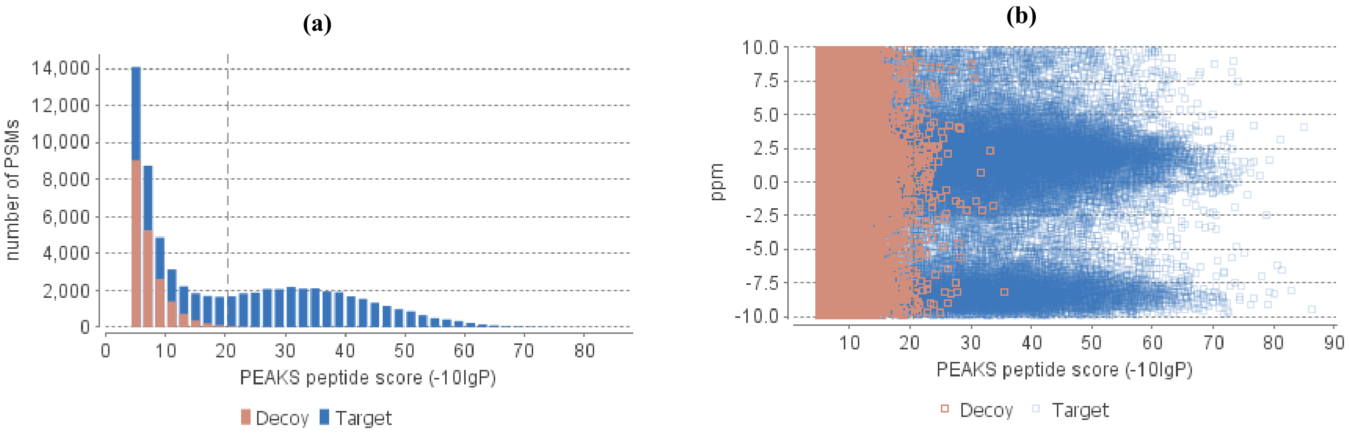

Figure 3. De novo result validation. Distribution of residue local confidence: **(a)** Residues in de novo sequences validated by confident database peptide assignment; **(b)** Residues in "de novo only" sequences.

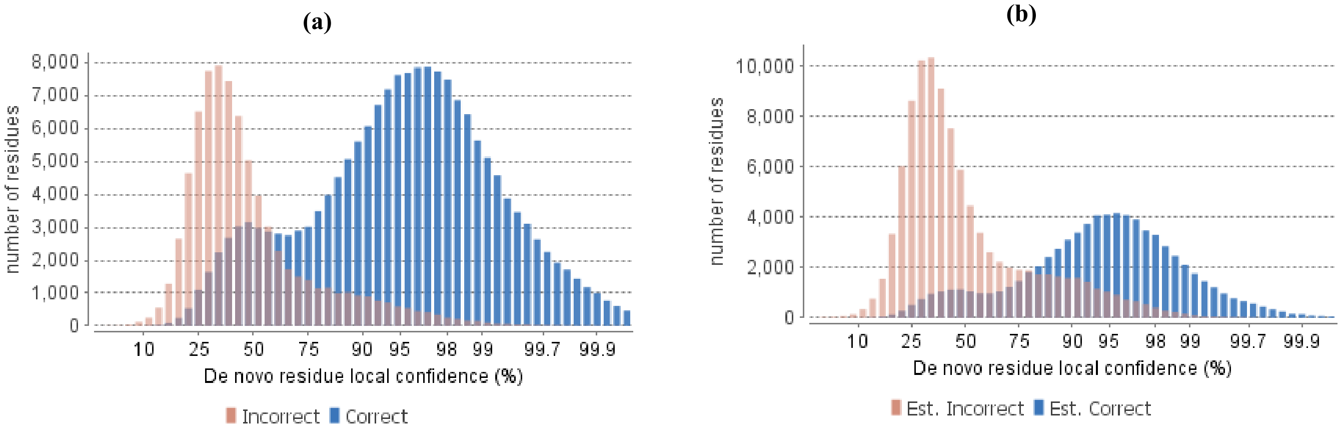

Table 1. Statistics of data.

# of MS scans	28023

Table 4. PTM profile.

Name	ΔMass	Position	#PSM	-10lgP	Area	AScor

# of MS/MS scans 126480

Table 2. Result filtration parameters.

Peptide -10lgP	≥20.5
Peptide Ascore	≥0
Protein -10lgP	≥20
Proteins unique peptides	≥0
De novo ALC Score	≥50%

Table 3. Statistics of filtered result.

Peptide-Spectrum Matches	30431
Peptide sequences	12102
Protein groups	1804
Proteins	3346
Proteins (#Unique Peptides)	1454 (>2); 451 (=2); 1170 (=1);
FDR (Peptide-Spectrum Matches)	0.5%
FDR (Peptide Sequences)	1.1%
De Novo Only Spectra	15249

Deamidation	.98	NQ	5533	86.26	6.99E6	32.9[illegible]
Oxidation	15.99	M	2639	78.07	1.48E7	1000.0[illegible]
Acetylation	42.01	N-term	589	64.81	2.52E7	1000.0[illegible]
Carbamidomethyl	57.02	C	37	50.53		1000.0[illegible]

3. Experiment Control

Figure 4. Precursor mass error of peptide-spectrum matches (PSM) in filtered result. **(a)** Distribution of precursor mass error in ppm; **(b)** Scatterplot of precursor m/z versus precursor mass error in ppm.

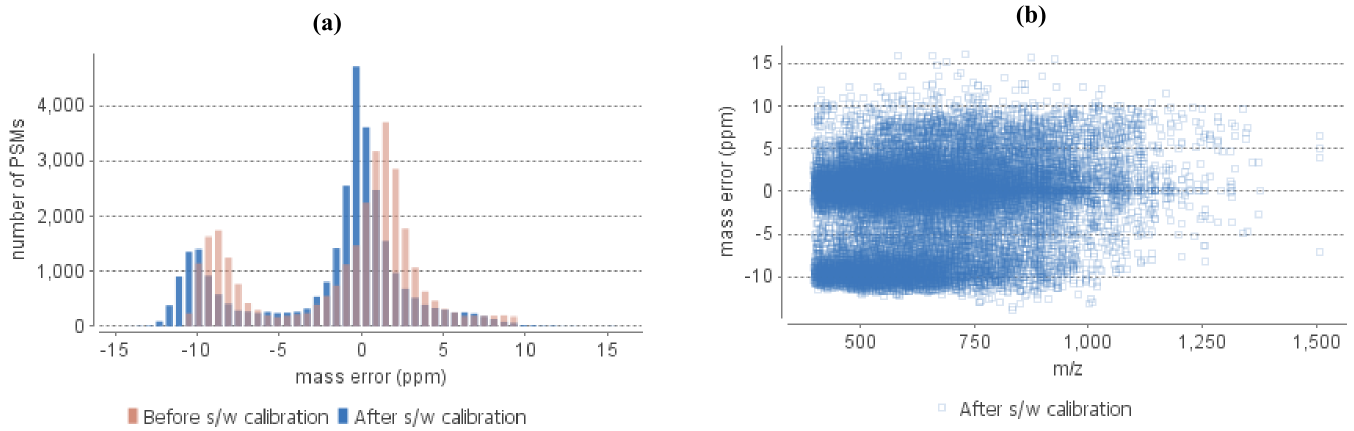

Table 5. Number of identified peptides in each sample by the number of missed cleavages

Missed Cleavages	0	1	2	3	4+
AZ046_01	3942	496	35	0	0
AZ046_02	3253	484	35	0	0
AZ046_03	3356	476	25	0	0

4. Other Information

Table 6. Search parameters.

Search Engine Name: PEAKS
Parent Mass Error Tolerance: 10.0 ppm
Fragment Mass Error Tolerance: 0.05 Da
Precursor Mass Search Type: monoisotopic
Enzyme: Trypsin
Max Missed Cleavages: 2
Non-specific Cleavage: one
Fixed Modifications:

Table 7. Instrument parameters.

Fractions: AZ046_01.raw, AZ046_02.raw, AZ046_03.raw
Ion Source: ESI(nano-spray)
Fragmentation Mode: high energy CID (y and b ions)
MS Scan Mode: FT-ICR/Orbitrap
MS/MS Scan Mode: FT-ICR/Orbitrap

Carbamidomethylation: 57.02
Variable Modifications:
Deamidation (NQ): 0.98
Acetylation (N-term): 42.01
Oxidation (M): 15.99
Max Variable PTM Per Peptide: 3
Database: PF_all
Taxon: All
Searched Entry: 1941073
FDR Estimation: Enabled
Merge Options: no merge
Precursor Options: corrected
Charge Options: no correction
Filter Options: no filter
Process: true

1. Notes

2. Result Statistics

Figure 1. False discovery rate (FDR) curve. X axis is the number of peptide-spectrum matches (PSM) being kept. Y axis is the corresponding FDR.

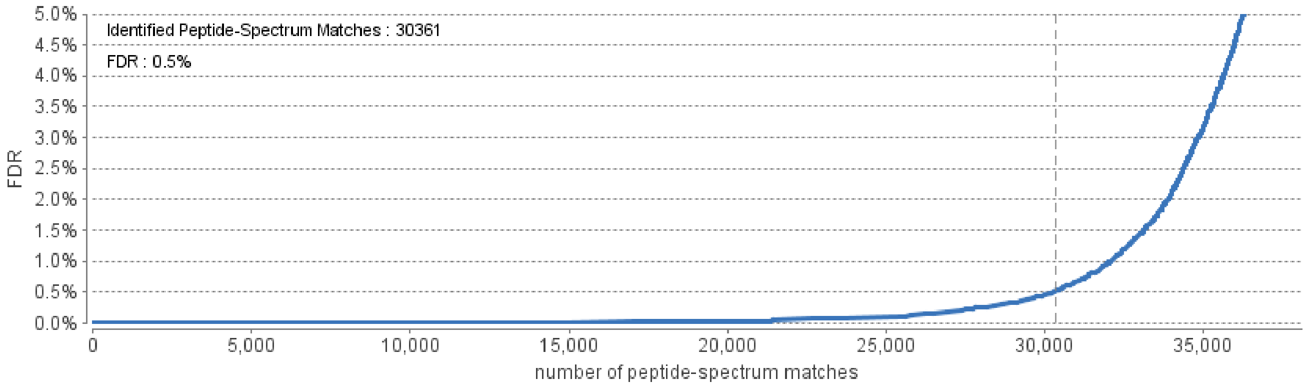

Figure 2. PSM score distribution. **(a)** Distribution of PEAKS peptide score; **(b)** Scatterplot of PEAKS peptide score versus precursor mass error.

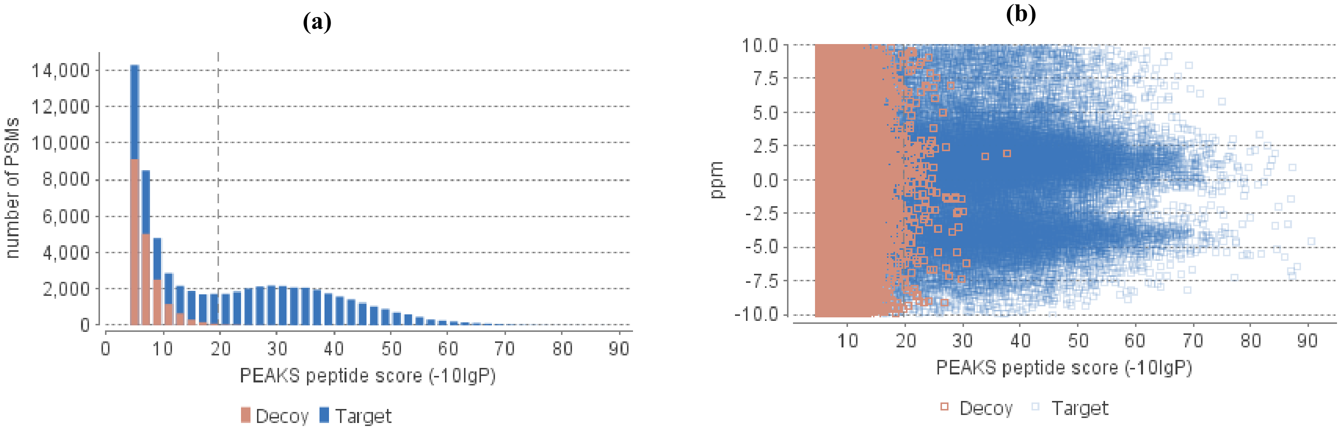

Figure 3. De novo result validation. Distribution of residue local confidence: **(a)** Residues in de novo sequences validated by confident database peptide assignment; **(b)** Residues in "de novo only" sequences.

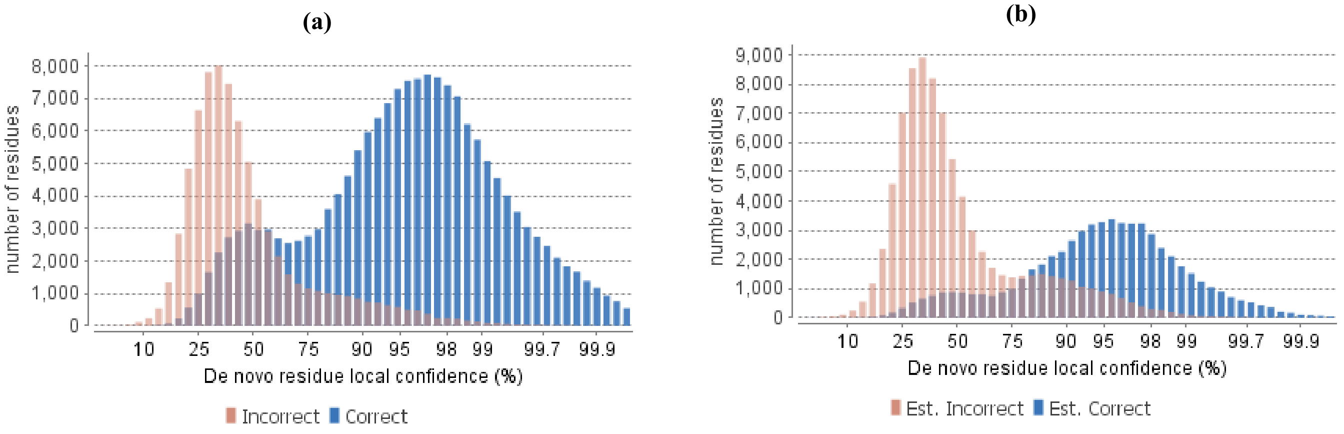

Table 1. Statistics of data.

# of MS scans	28419

Table 4. PTM profile.

Name	ΔMass	Position	#PSM	-10lgP	Area	AScore

# of MS/MS scans 124862

Deamidation	.98	NQ	5762	90.51	3.12E7	32.9[illegible]
Oxidation	15.99	M	2277	84.48	1.41E7	1000.0[illegible]
Acetylation	42.01	N-term	452	57.78		1000.0[illegible]
Carbamidomethyl	57.02	C	74	67.18		1000.0[illegible]

Table 2. Result filtration parameters.

Peptide -10lgP	≥19.7
Peptide Ascore	≥0
Protein -10lgP	≥20
Proteins unique peptides	≥0
De novo ALC Score	≥50%

Table 3. Statistics of filtered result.

Peptide-Spectrum Matches	30361
Peptide sequences	10930
Protein groups	1587
Proteins	3087
Proteins (#Unique Peptides)	1229 (>2); 388 (=2); 1193 (=1);
FDR (Peptide-Spectrum Matches)	0.5%
FDR (Peptide Sequences)	1.1%
De Novo Only Spectra	12762

3. Experiment Control

Figure 4. Precursor mass error of peptide-spectrum matches (PSM) in filtered result. **(a)** Distribution of precursor mass error in ppm; **(b)** Scatterplot of precursor m/z versus precursor mass error in ppm.

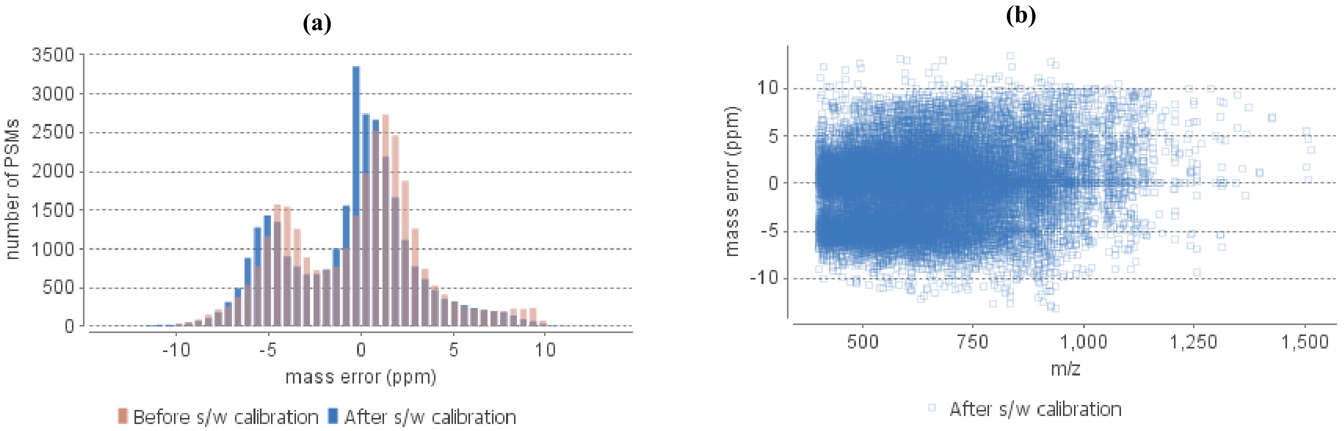

Table 5. Number of identified peptides in each sample by the number of missed cleavages

Missed Cleavages	0	1	2	3	4+
AZ047_01	4049	635	45	0	0
AZ047_02	2862	431	39	0	0
AZ047_03	2447	393	29	0	0

4. Other Information

Table 6. Search parameters.

Search Engine Name: PEAKS
Parent Mass Error Tolerance: 10.0 ppm
Fragment Mass Error Tolerance: 0.05 Da
Precursor Mass Search Type: monoisotopic
Enzyme: Trypsin
Max Missed Cleavages: 2
Non-specific Cleavage: one
Fixed Modifications:

Table 7. Instrument parameters.

Fractions: AZ047_01.raw, AZ047_02.raw, AZ047_03.raw
Ion Source: ESI(nano-spray)
Fragmentation Mode: high energy CID (y and b ions)
MS Scan Mode: FT-ICR/Orbitrap
MS/MS Scan Mode: FT-ICR/Orbitrap

Carbamidomethylation: 57.02
Variable Modifications:
Deamidation (NQ): 0.98
Acetylation (N-term): 42.01
Oxidation (M): 15.99
Max Variable PTM Per Peptide: 3
Database: PF_all
Taxon: All
Searched Entry: 1941073
FDR Estimation: Enabled
Merge Options: no merge
Precursor Options: corrected
Charge Options: no correction
Filter Options: no filter
Process: true

1. Notes

2. Result Statistics

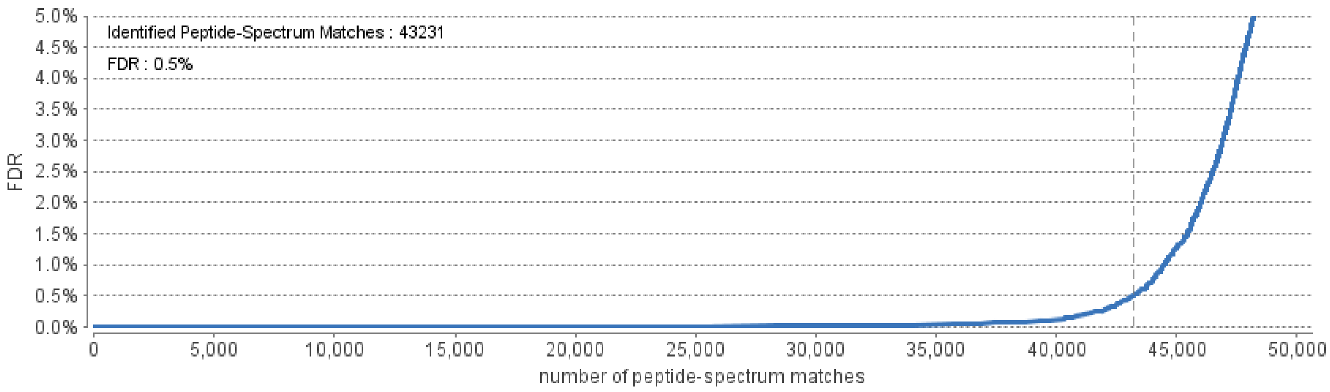

Figure 1. False discovery rate (FDR) curve. X axis is the number of peptide-spectrum matches (PSM) being kept. Y axis is the corresponding FDR.

Figure 2. PSM score distribution. **(a)** Distribution of PEAKS peptide score; **(b)** Scatterplot of PEAKS peptide score versus precursor mass error.

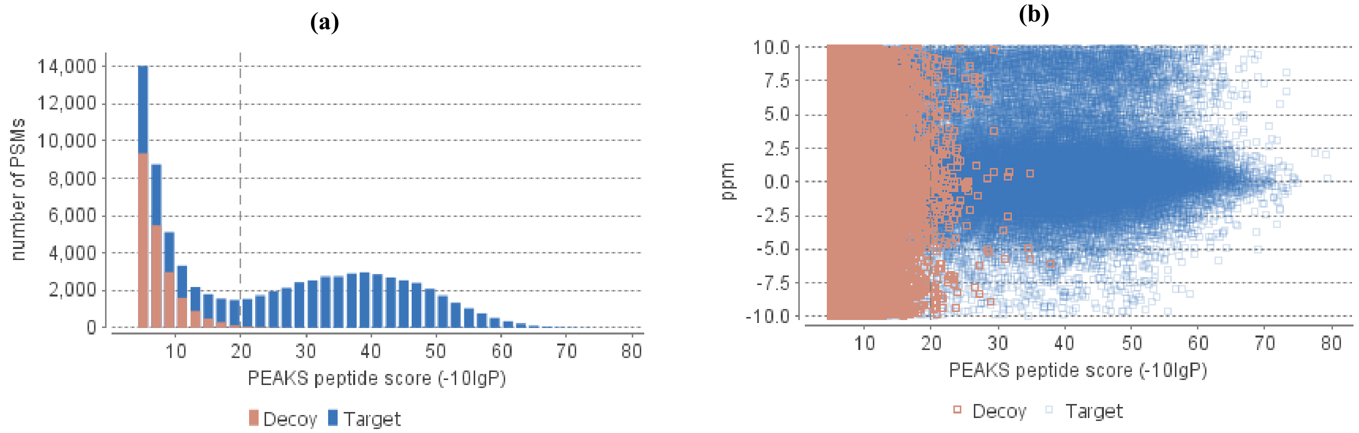

Figure 3. De novo result validation. Distribution of residue local confidence: **(a)** Residues in de novo sequences validated by confident database peptide assignment; **(b)** Residues in "de novo only" sequences.

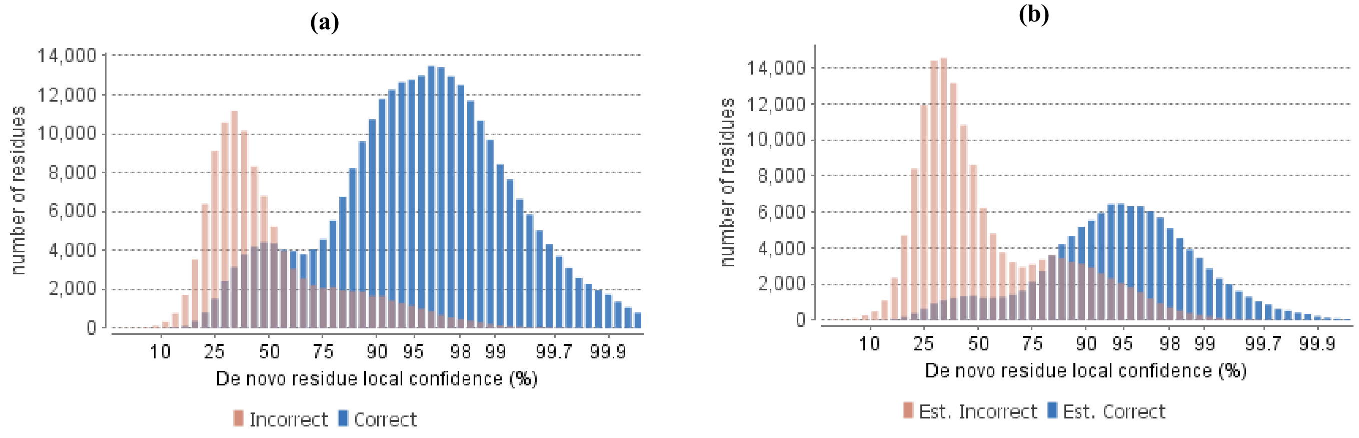

Table 1. Statistics of data.

Table 4. PTM profile.

# of MS scans	24976
# of MS/MS scans	138174

Table 2. Result filtration parameters.

Peptide -10lgP	≥19.9
Peptide Ascore	≥0
Protein -10lgP	≥20
Proteins unique peptides	≥0
De novo ALC Score	≥50%

Table 3. Statistics of filtered result.

Peptide-Spectrum Matches	43231
Peptide sequences	16459
Protein groups	2129
Proteins	3426
Proteins (#Unique Peptides)	1741 (>2); 399 (=2); 979 (=1);
FDR (Peptide-Spectrum Matches)	0.5%
FDR (Peptide Sequences)	1.0%
De Novo Only Spectra	21084

Name	ΔMass	Position	#PSM	-10lgP	Area	AScore
Deamidation	.98	NQ	8774	79.13	1.89E7	151.14
Oxidation	15.99	M	4343	74.29	7.6E6	1000.00
Acetylation	42.01	N-term	1182	65.15		1000.00
Carbamidomethyl	57.02	C	188	59.87	6.5E6	1000.00

3. Experiment Control

Figure 4. Precursor mass error of peptide-spectrum matches (PSM) in filtered result. **(a)** Distribution of precursor mass error in ppm; **(b)** Scatterplot of precursor m/z versus precursor mass error in ppm.

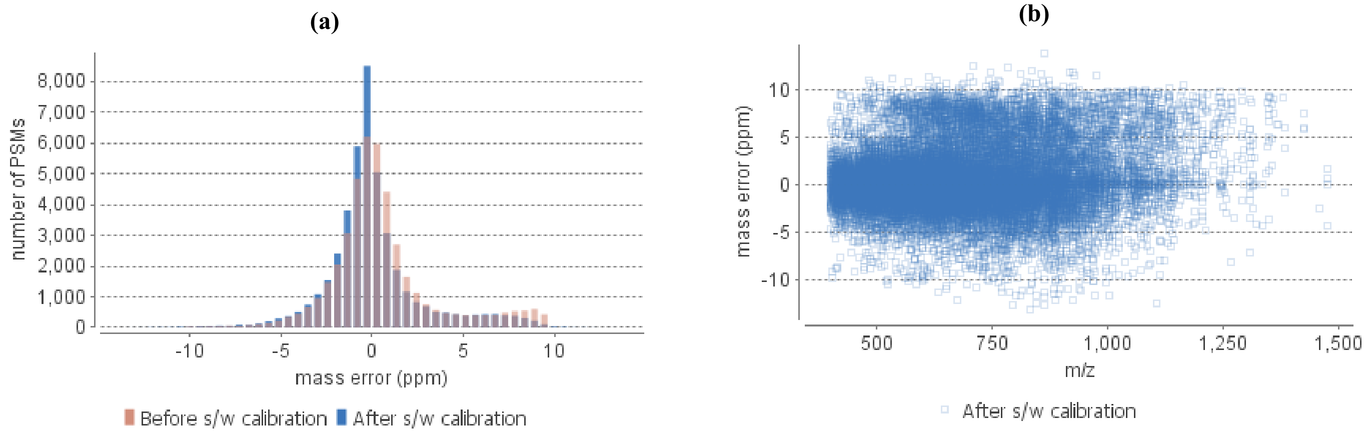

Table 5. Number of identified peptides in each sample by the number of missed cleavages

Missed Cleavages	0	1	2	3	4+
AZ048_01	4790	877	98	0	0
AZ048_02	4993	805	68	0	0
AZ048_03	4050	705	73	0	0

4. Other Information

Table 6. Search parameters.

Search Engine Name: PEAKS
Parent Mass Error Tolerance: 10.0 ppm
Fragment Mass Error Tolerance: 0.05 Da
Precursor Mass Search Type: monoisotopic
Enzyme: Trypsin
Max Missed Cleavages: 2
Non-specific Cleavage: one

Table 7. Instrument parameters.

Fractions: AZ048_01.raw, AZ048_02.raw, AZ048_03.raw
Ion Source: ESI(nano-spray)
Fragmentation Mode: high energy CID (y and b ions)
MS Scan Mode: FT-ICR/Orbitrap
MS/MS Scan Mode: FT-ICR/Orbitrap

Fixed Modifications:
  Carbamidomethylation: 57.02
Variable Modifications:
  Deamidation (NQ): 0.98
  Acetylation (N-term): 42.01
  Oxidation (M): 15.99
Max Variable PTM Per Peptide: 3
Database: PF_all
Taxon: All
Searched Entry: 1941073
FDR Estimation: Enabled
Merge Options: no merge
Precursor Options: corrected
Charge Options: no correction
Filter Options: no filter
Process: true

1. Notes

2. Result Statistics

Figure 1. False discovery rate (FDR) curve. X axis is the number of peptide-spectrum matches (PSM) being kept. Y axis is the corresponding FDR.

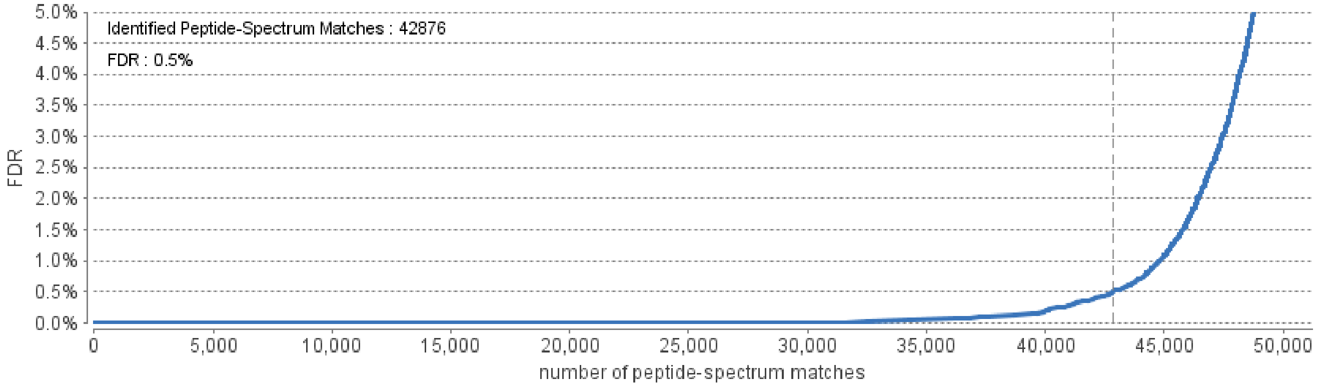

Figure 2. PSM score distribution. **(a)** Distribution of PEAKS peptide score; **(b)** Scatterplot of PEAKS peptide score versus precursor mass error.

(a)

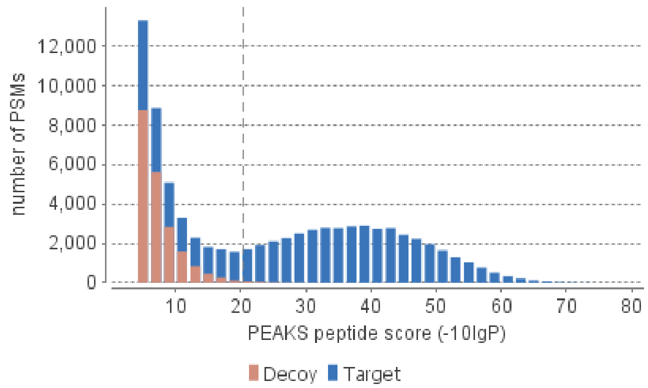

(b)

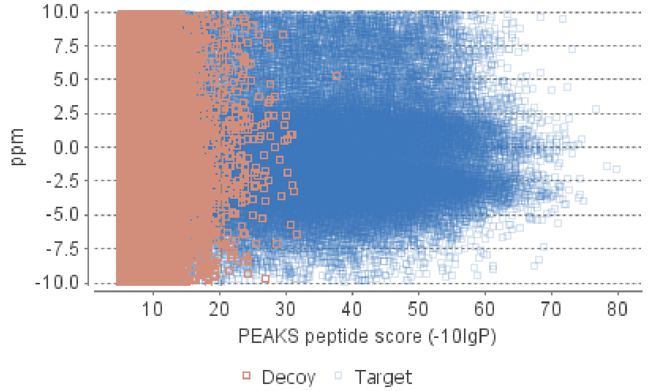

Figure 3. De novo result validation. Distribution of residue local confidence: **(a)** Residues in de novo sequences validated by confident database peptide assignment; **(b)** Residues in "de novo only" sequences.

(a)

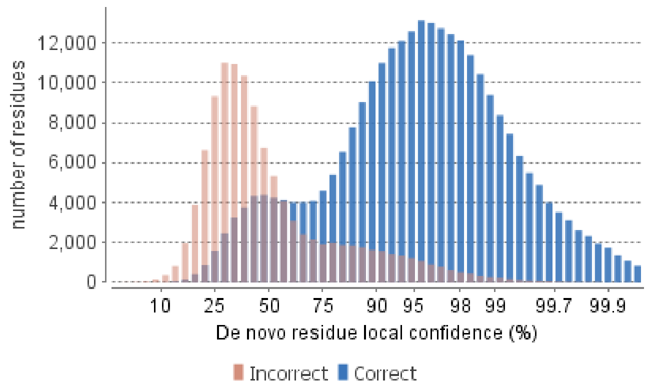

(b)

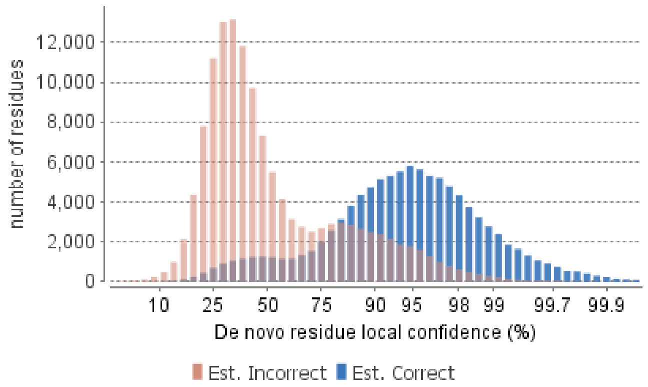

Table 1. Statistics of data.

Table 4. PTM profile.

# of MS scans	25410
# of MS/MS scans	136203

Name	ΔMass	Position	#PSM	-10lgP	Area	AScore
Deamidation	.98	NQ	9274	74.29	3.82E7	9.34
Oxidation	15.99	M	4325	72.27	1.7E8	1000.00
Acetylation	42.01	N-term	1017	63.56	3.19E6	1000.00
Carbamidomethyl	57.02	C	203	64.71		1000.00

Table 2. Result filtration parameters.

Peptide -10lgP	≥20.3
Peptide Ascore	≥0
Protein -10lgP	≥20
Proteins unique peptides	≥0
De novo ALC Score	≥50%

Table 3. Statistics of filtered result.

Peptide-Spectrum Matches	42876
Peptide sequences	15863
Protein groups	2004
Proteins	3410
Proteins (#Unique Peptides)	1621 (>2); 470 (=2); 964 (=1);
FDR (Peptide-Spectrum Matches)	0.5%
FDR (Peptide Sequences)	1.0%
De Novo Only Spectra	18567

3. Experiment Control

Figure 4. Precursor mass error of peptide-spectrum matches (PSM) in filtered result. **(a)** Distribution of precursor mass error in ppm; **(b)** Scatterplot of precursor m/z versus precursor mass error in ppm.

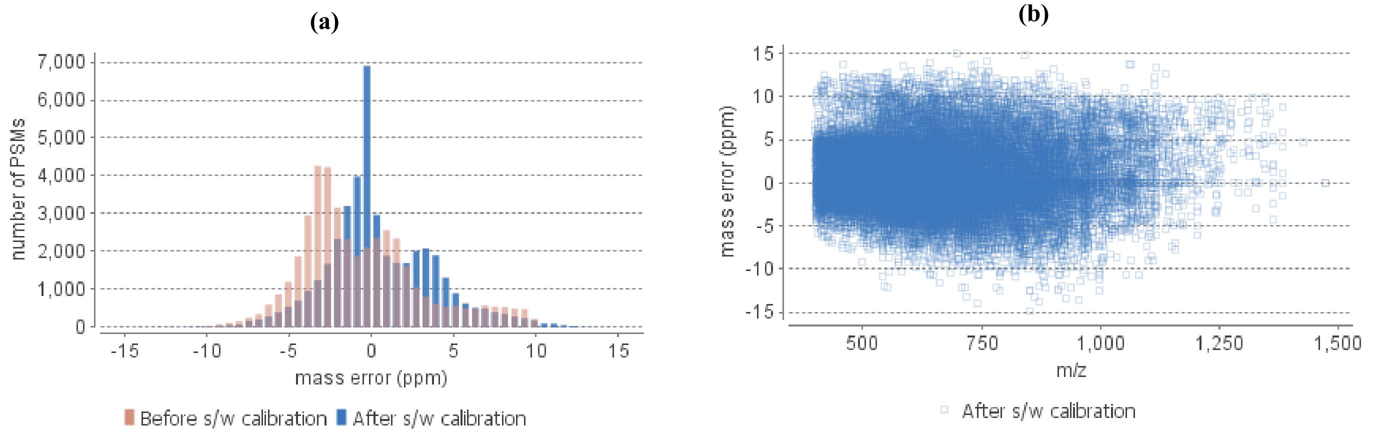

Table 5. Number of identified peptides in each sample by the number of missed cleavages

Missed Cleavages	0	1	2	3	4+
AZ050_01	4449	784	73	0	0
AZ050_02	4427	749	57	0	0
AZ050_03	4550	716	58	0	0

4. Other Information

Table 6. Search parameters.

Search Engine Name: PEAKS
Parent Mass Error Tolerance: 10.0 ppm
Fragment Mass Error Tolerance: 0.05 Da
Precursor Mass Search Type: monoisotopic
Enzyme: Trypsin
Max Missed Cleavages: 2
Non-specific Cleavage: one

Table 7. Instrument parameters.

Fractions: AZ050_01.raw, AZ050_02.raw, AZ050_03.raw
Ion Source: ESI(nano-spray)
Fragmentation Mode: high energy CID (y and b ions)
MS Scan Mode: FT-ICR/Orbitrap
MS/MS Scan Mode: FT-ICR/Orbitrap

Fixed Modifications:
  Carbamidomethylation: 57.02
Variable Modifications:
  Deamidation (NQ): 0.98
  Acetylation (N-term): 42.01
  Oxidation (M): 15.99
Max Variable PTM Per Peptide: 3
Database: PF_all
Taxon: All
Searched Entry: 1941073
FDR Estimation: Enabled
Merge Options: no merge
Precursor Options: corrected
Charge Options: no correction
Filter Options: no filter
Process: true

1. Notes

2. Result Statistics

Figure 1. False discovery rate (FDR) curve. X axis is the number of peptide-spectrum matches (PSM) being kept. Y axis is the corresponding FDR.

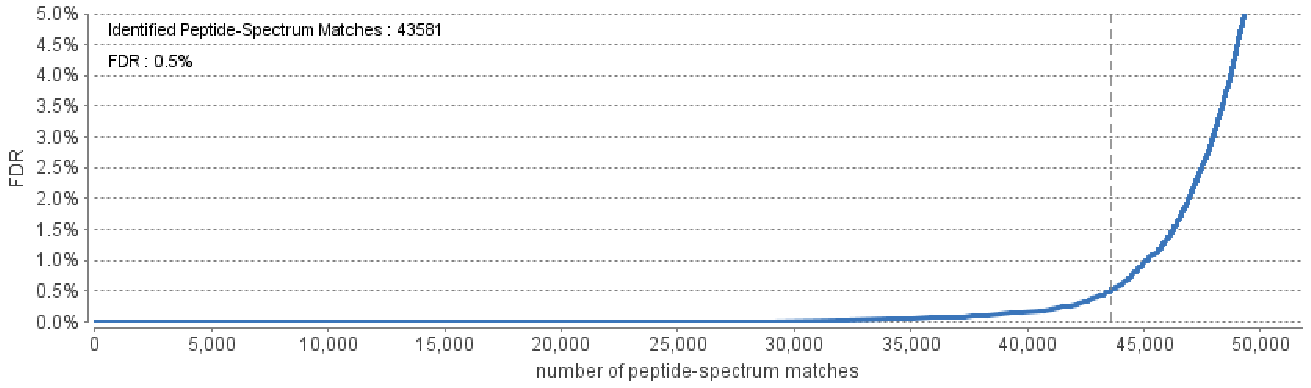

Figure 2. PSM score distribution. **(a)** Distribution of PEAKS peptide score; **(b)** Scatterplot of PEAKS peptide score versus precursor mass error.

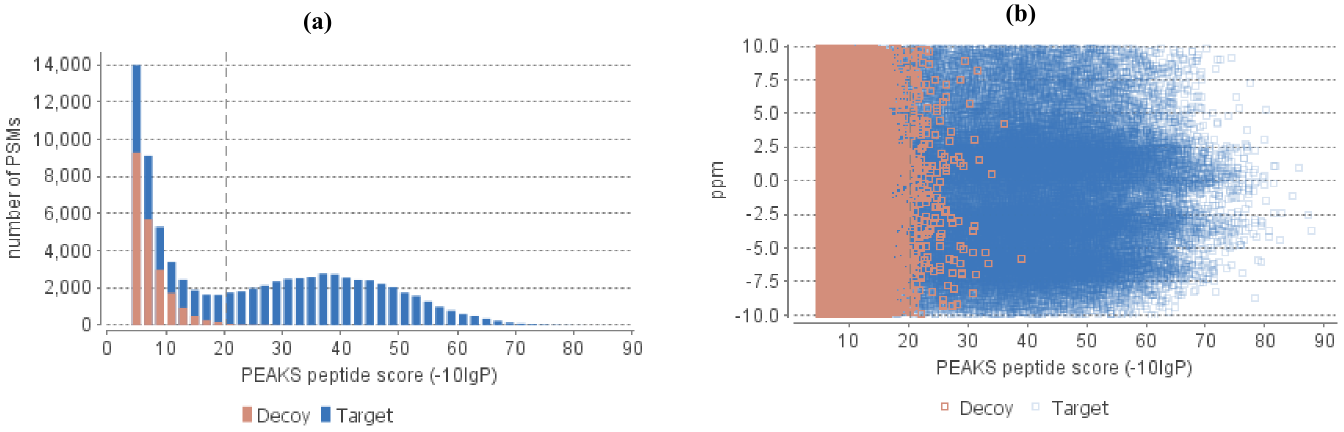

Figure 3. De novo result validation. Distribution of residue local confidence: **(a)** Residues in de novo sequences validated by confident database peptide assignment; **(b)** Residues in "de novo only" sequences.

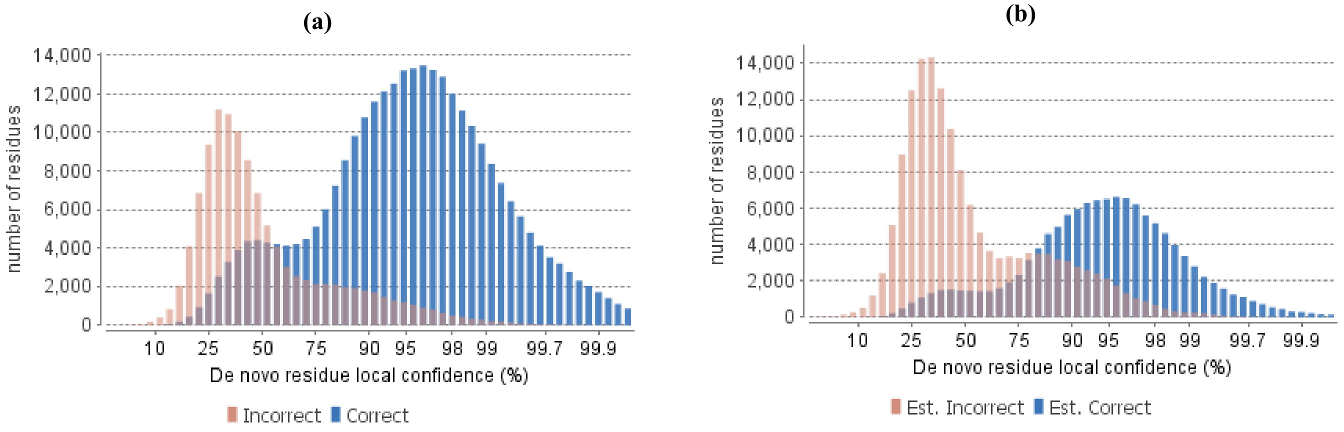

Table 1. Statistics of data.

# of MS scans	24618

Table 4. PTM profile.

Name	ΔMass	Position	#PSM	-10lgP	Area	AScor[illegible]

# of MS/MS scans 139456

Deamidation	.98	NQ	10487	87.75	6.35E7	38.1(
Oxidation	15.99	M	4387	80.71	1.94E8	1000.0(
Acetylation	42.01	N-term	1093	74.48	1.99E6	1000.0(
Carbamidomethyl	57.02	C	196	65.77		1000.0(

Table 2. Result filtration parameters.

Peptide -10lgP	≥20.3
Peptide Ascore	≥0
Protein -10lgP	≥20
Proteins unique peptides	≥0
De novo ALC Score	≥50%

Table 3. Statistics of filtered result.

Peptide-Spectrum Matches	43581
Peptide sequences	15954
Protein groups	2097
Proteins	3473
Proteins (#Unique Peptides)	1614 (>2); 485 (=2); 1079 (=1);
FDR (Peptide-Spectrum Matches)	0.5%
FDR (Peptide Sequences)	1.1%
De Novo Only Spectra	21305

3. Experiment Control

Figure 4. Precursor mass error of peptide-spectrum matches (PSM) in filtered result. **(a)** Distribution of precursor mass error in ppm; **(b)** Scatterplot of precursor m/z versus precursor mass error in ppm.

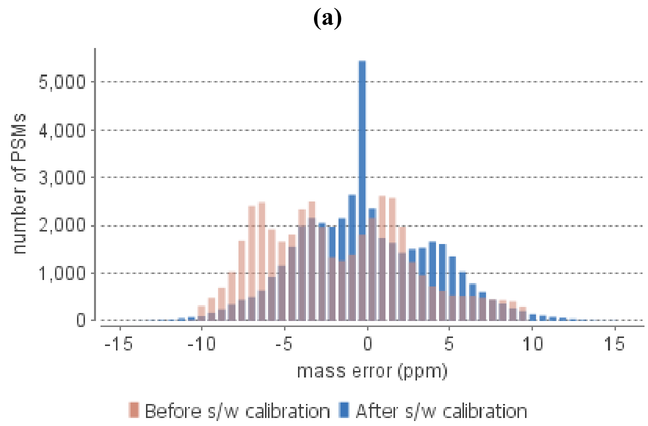

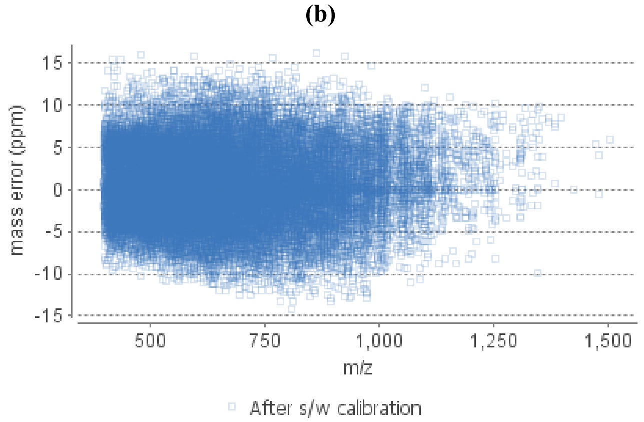

Table 5. Number of identified peptides in each sample by the number of missed cleavages

Missed Cleavages	0	1	2	3	4+
AZ051_01	4833	854	84	0	0
AZ051_02	4665	750	73	0	0
AZ051_03	3898	735	62	0	0

4. Other Information

Table 6. Search parameters.
Search Engine Name: PEAKS
Parent Mass Error Tolerance: 10.0 ppm
Fragment Mass Error Tolerance: 0.05 Da
Precursor Mass Search Type: monoisotopic
Enzyme: Trypsin
Max Missed Cleavages: 2
Non-specific Cleavage: one
Fixed Modifications:

Table 7. Instrument parameters.
Fractions: AZ051_01.raw, AZ051_02.raw, AZ051_03.raw
Ion Source: ESI(nano-spray)
Fragmentation Mode: high energy CID (y and b ions)
MS Scan Mode: FT-ICR/Orbitrap
MS/MS Scan Mode: FT-ICR/Orbitrap

Carbamidomethylation: 57.02
Variable Modifications:
Deamidation (NQ): 0.98
Acetylation (N-term): 42.01
Oxidation (M): 15.99
Max Variable PTM Per Peptide: 3
Database: PF_all
Taxon: All
Searched Entry: 1941073
FDR Estimation: Enabled
Merge Options: no merge
Precursor Options: corrected
Charge Options: no correction
Filter Options: no filter
Process: true

1. Notes

2. Result Statistics

Figure 1. False discovery rate (FDR) curve. X axis is the number of peptide-spectrum matches (PSM) being kept. Y axis is the corresponding FDR.

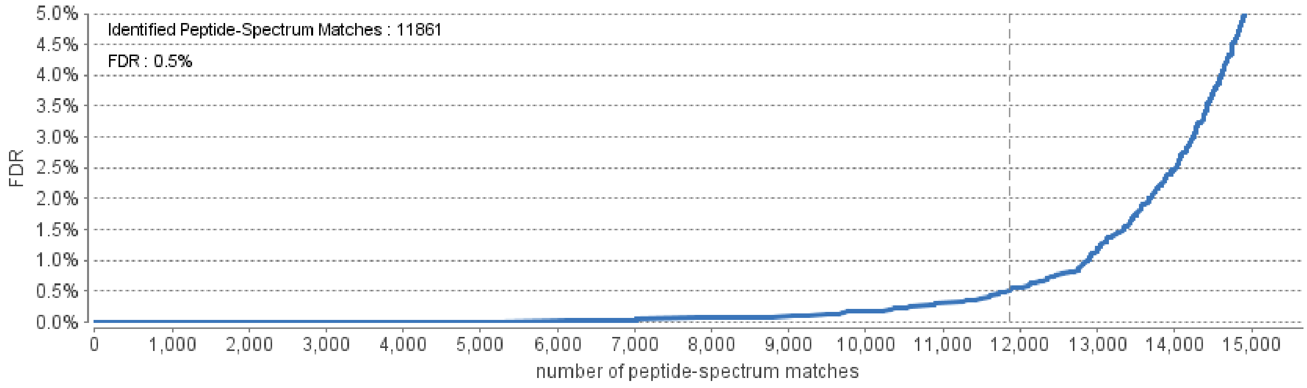

Figure 2. PSM score distribution. **(a)** Distribution of PEAKS peptide score; **(b)** Scatterplot of PEAKS peptide score versus precursor mass error.

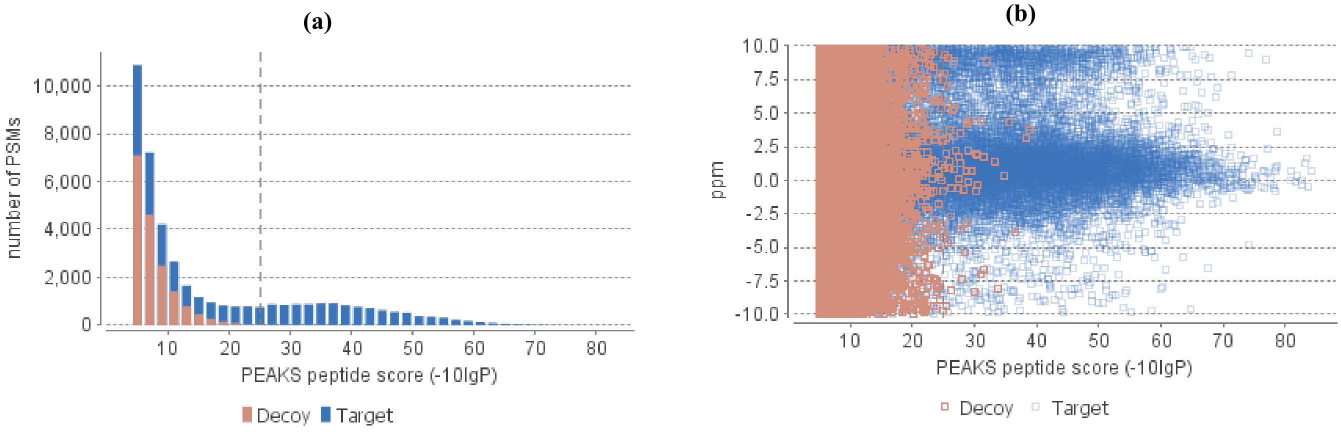

Figure 3. De novo result validation. Distribution of residue local confidence: **(a)** Residues in de novo sequences validated by confident database peptide assignment; **(b)** Residues in "de novo only" sequences.

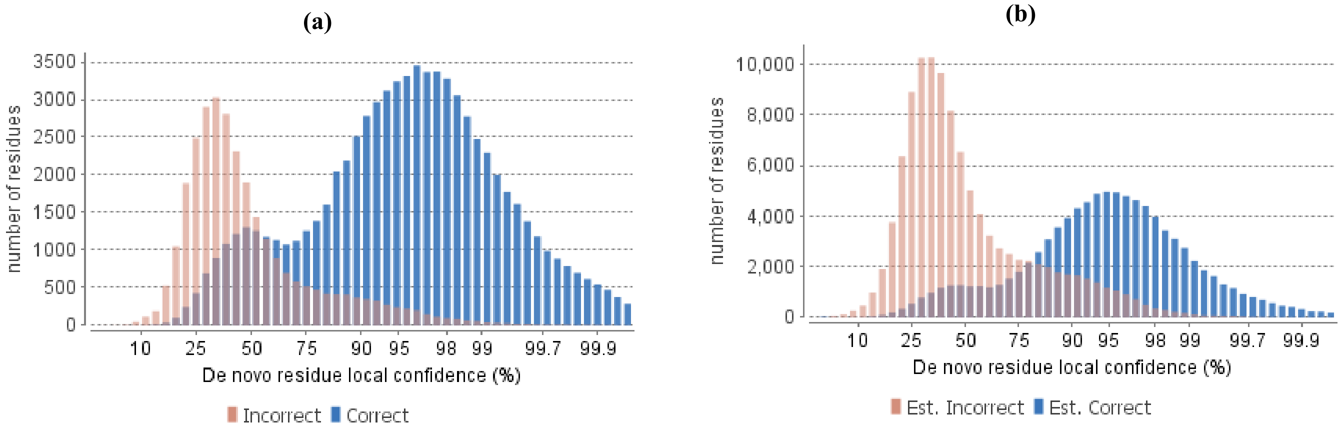

Table 1. Statistics of data.

# of MS scans	17635

Table 4. PTM profile.

Name	ΔMass	Position	#PSM	-10lgP	Area	AScor[illegible]

# of MS/MS scans 88589

Deamidation	.98	NQ	2116	84.12	2.69E7	32.9[illegible]
Oxidation	15.99	M	999	80.81	7.84E7	1000.0[illegible]
Acetylation	42.01	N-term	245	62.76	2.16E6	1000.0[illegible]
Carbamidomethyl	57.02	C	28	54.50		1000.0[illegible]

Table 2. Result filtration parameters.

Peptide -10lgP	≥25
Peptide Ascore	≥0
Protein -10lgP	≥20
Proteins unique peptides	≥0
De novo ALC Score	≥50%

Table 3. Statistics of filtered result.

Peptide-Spectrum Matches	11861
Peptide sequences	7817
Protein groups	1686
Proteins	3264
Proteins (#Unique Peptides)	1079 (>2); 440 (=2); 1315 (=1);
FDR (Peptide-Spectrum Matches)	0.5%
FDR (Peptide Sequences)	0.7%
De Novo Only Spectra	17511

3. Experiment Control

Figure 4. Precursor mass error of peptide-spectrum matches (PSM) in filtered result. **(a)** Distribution of precursor mass error in ppm; **(b)** Scatterplot of precursor m/z versus precursor mass error in ppm.

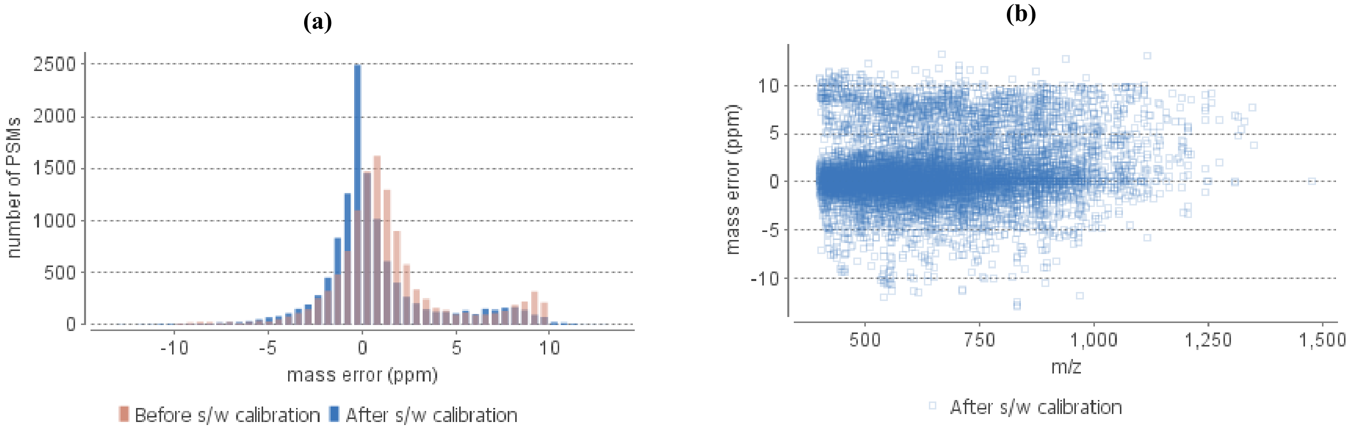

Table 5. Number of identified peptides in each sample by the number of missed cleavages

Missed Cleavages	0	1	2	3	4+
AZ053_01	6248	767	53	0	0
AZ053_03	587	142	20	0	0

4. Other Information

Table 6. Search parameters.

Search Engine Name: PEAKS
Parent Mass Error Tolerance: 10.0 ppm
Fragment Mass Error Tolerance: 0.05 Da
Precursor Mass Search Type: monoisotopic
Enzyme: Trypsin
Max Missed Cleavages: 2
Non-specific Cleavage: one
Fixed Modifications:
  Carbamidomethylation: 57.02
Variable Modifications:

Table 7. Instrument parameters.

Fractions: AZ053_01.raw, AZ053_02.raw, AZ053_03.raw
Ion Source: ESI(nano-spray)
Fragmentation Mode: high energy CID (y and b ions)
MS Scan Mode: FT-ICR/Orbitrap
MS/MS Scan Mode: FT-ICR/Orbitrap

Deamidation (NQ): 0.98
Acetylation (N-term): 42.01
Oxidation (M): 15.99
Max Variable PTM Per Peptide: 3
Database: PF_all
Taxon: All
Searched Entry: 1941073
FDR Estimation: Enabled
Merge Options: no merge
Precursor Options: corrected
Charge Options: no correction
Filter Options: no filter
Process: true

1. Notes

2. Result Statistics

Figure 1. False discovery rate (FDR) curve. X axis is the number of peptide-spectrum matches (PSM) being kept. Y axis is the corresponding FDR.

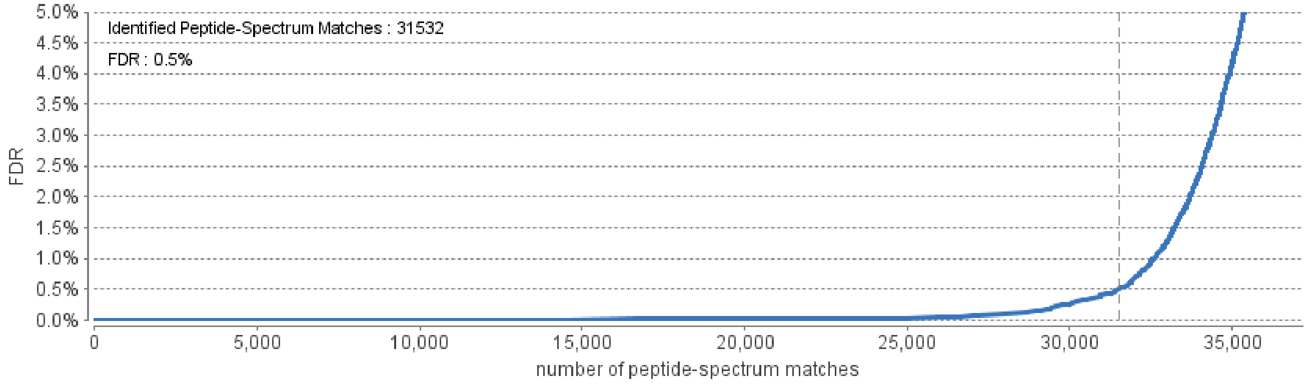

Figure 2. PSM score distribution. **(a)** Distribution of PEAKS peptide score; **(b)** Scatterplot of PEAKS peptide score versus precursor mass error.

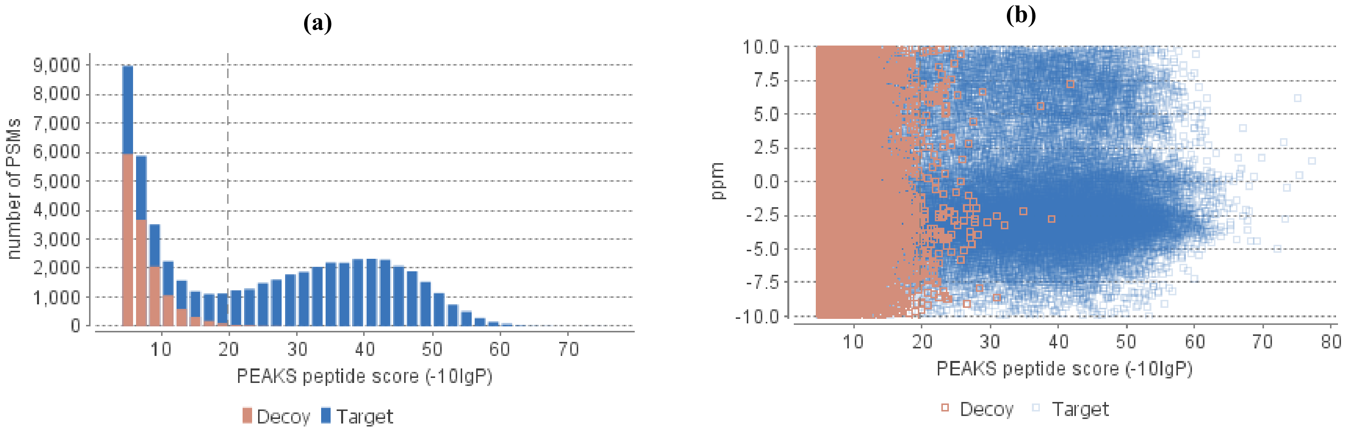

Figure 3. De novo result validation. Distribution of residue local confidence: **(a)** Residues in de novo sequences validated by confident database peptide assignment; **(b)** Residues in "de novo only" sequences.

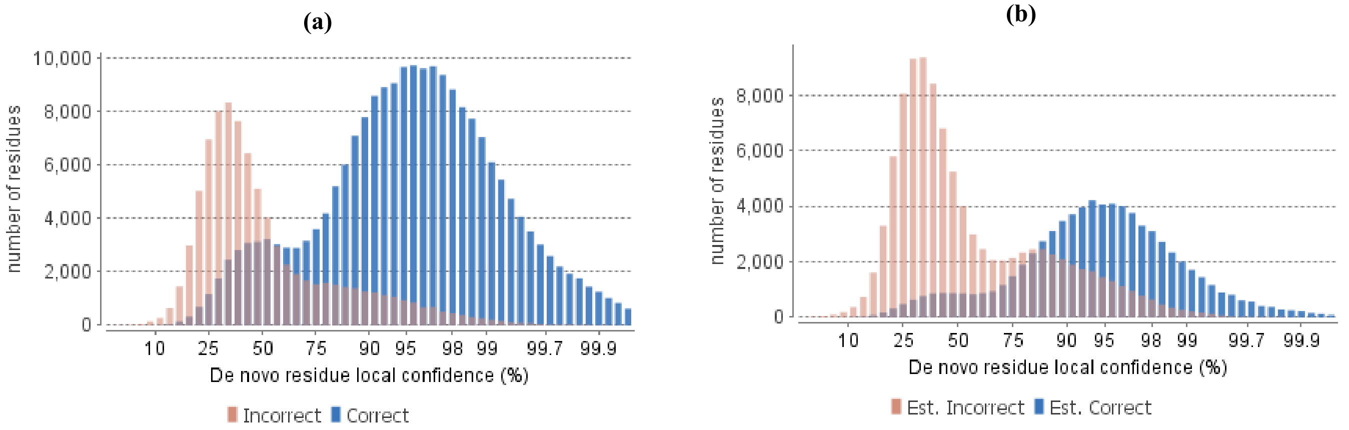

Table 1. Statistics of data.

# of MS scans	16347

Table 4. PTM profile.

Name	ΔMass	Position	#PSM	-10lgP	Area	AScor

# of MS/MS scans 93312

Deamidation	.98	NQ	7742	77.13		38.1[illegible]
Oxidation	15.99	M	2673	77.13		1000.0[illegible]
Acetylation	42.01	N-term	777	59.68	4.56E6	1000.0[illegible]
Carbamidomethyl	57.02	C	196	61.11	4.83E6	1000.0[illegible]

Table 2. Result filtration parameters.

Peptide -10lgP	≥19.8
Peptide Ascore	≥0
Protein -10lgP	≥20
Proteins unique peptides	≥0
De novo ALC Score	≥50%

Table 3. Statistics of filtered result.

Peptide-Spectrum Matches	31532
Peptide sequences	14916
Protein groups	1918
Proteins	3349
Proteins (#Unique Peptides)	1606 (>2); 399 (=2); 1090 (=1);
FDR (Peptide-Spectrum Matches)	0.5%
FDR (Peptide Sequences)	0.8%
De Novo Only Spectra	13473

3. Experiment Control

Figure 4. Precursor mass error of peptide-spectrum matches (PSM) in filtered result. **(a)** Distribution of precursor mass error in ppm; **(b)** Scatterplot of precursor m/z versus precursor mass error in ppm.

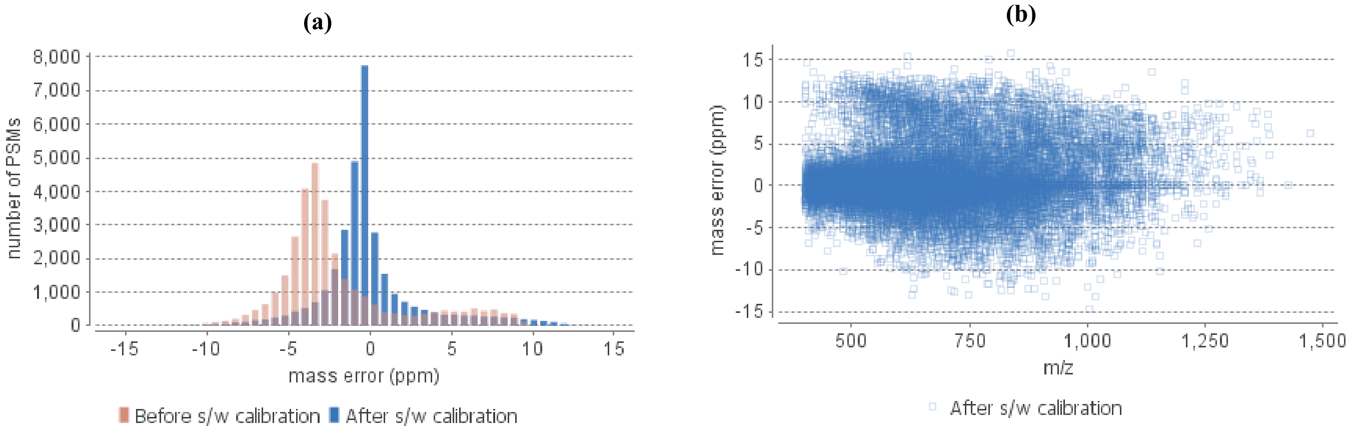

Table 5. Number of identified peptides in each sample by the number of missed cleavages

Missed Cleavages	0	1	2	3	4+
AZ055_01	7372	1139	95	0	0
AZ055_02	5309	913	88	0	0

4. Other Information

Table 6. Search parameters.

Search Engine Name: PEAKS
Parent Mass Error Tolerance: 10.0 ppm
Fragment Mass Error Tolerance: 0.05 Da
Precursor Mass Search Type: monoisotopic
Enzyme: Trypsin
Max Missed Cleavages: 2
Non-specific Cleavage: one
Fixed Modifications:
  Carbamidomethylation: 57.02
Variable Modifications:

Table 7. Instrument parameters.

Fractions: AZ055_01.raw, AZ055_02.raw, AZ055_03.raw
Ion Source: ESI(nano-spray)
Fragmentation Mode: high energy CID (y and b ions)
MS Scan Mode: FT-ICR/Orbitrap
MS/MS Scan Mode: FT-ICR/Orbitrap

Deamidation (NQ): 0.98
Acetylation (N-term): 42.01
Oxidation (M): 15.99
Max Variable PTM Per Peptide: 3
Database: PF_all
Taxon: All
Searched Entry: 1941073
FDR Estimation: Enabled
Merge Options: no merge
Precursor Options: corrected
Charge Options: no correction
Filter Options: no filter
Process: true

1. Notes

2. Result Statistics

Figure 1. False discovery rate (FDR) curve. X axis is the number of peptide-spectrum matches (PSM) being kept. Y axis is the corresponding FDR.

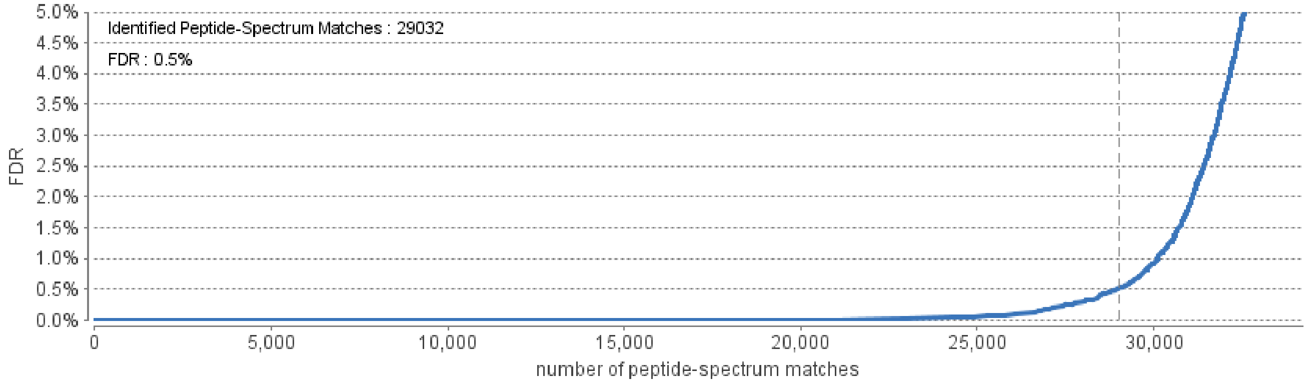

Figure 2. PSM score distribution. **(a)** Distribution of PEAKS peptide score; **(b)** Scatterplot of PEAKS peptide score versus precursor mass error.

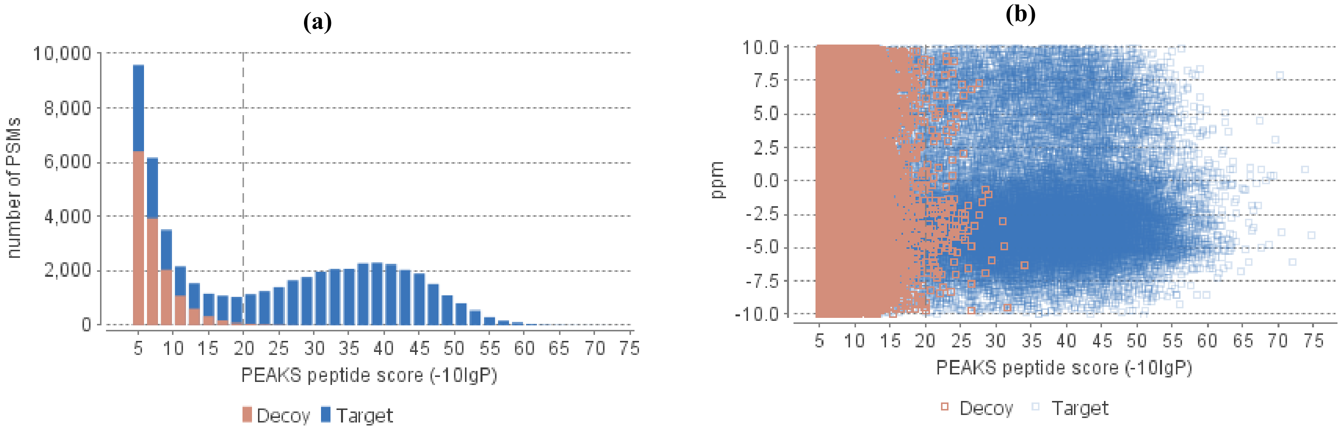

Figure 3. De novo result validation. Distribution of residue local confidence: **(a)** Residues in de novo sequences validated by confident database peptide assignment; **(b)** Residues in "de novo only" sequences.

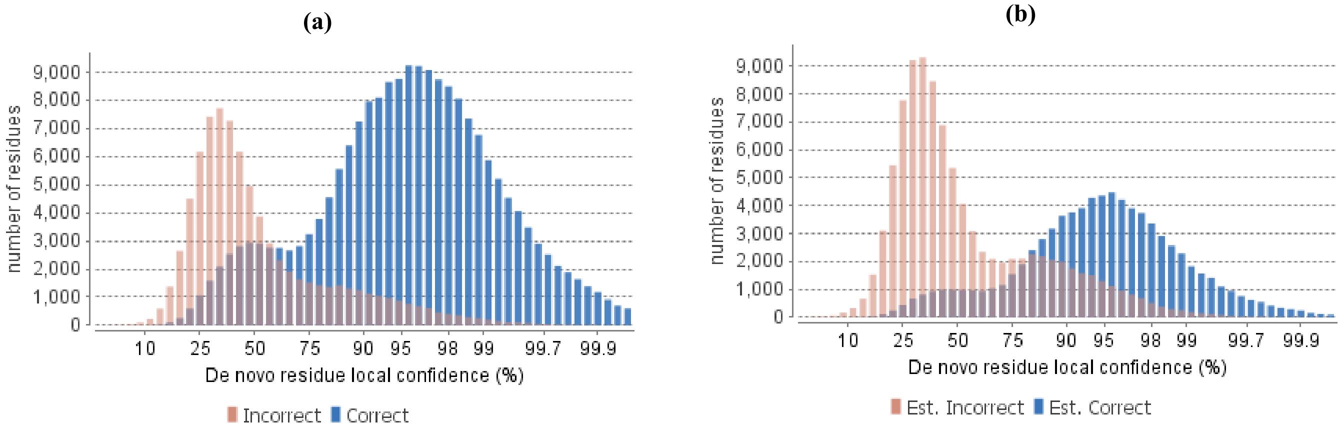

Table 1. Statistics of data.

# of MS scans	16070

Table 4. PTM profile.

Name	ΔMass	Position	#PSM	-10lgP	Area	AScor[illegible]

# of MS/MS scans 93946

Deamidation	.98	NQ	7544	74.63	3.88E6	38.1[illegible]
Oxidation	15.99	M	3067	74.63	3.88E6	1000.0[illegible]
Acetylation	42.01	N-term	731	59.82	2.78E6	1000.0[illegible]
Carbamidomethyl	57.02	C	163	56.58	6.97E6	1000.0[illegible]

Table 2. Result filtration parameters.

Peptide -10lgP	≥20
Peptide Ascore	≥0
Protein -10lgP	≥20
Proteins unique peptides	≥0
De novo ALC Score	≥50%

Table 3. Statistics of filtered result.

Peptide-Spectrum Matches	29032
Peptide sequences	14337
Protein groups	1928
Proteins	3416
Proteins (#Unique Peptides)	1492 (>2); 433 (=2); 1238 (=1);
FDR (Peptide-Spectrum Matches)	0.5%
FDR (Peptide Sequences)	0.8%
De Novo Only Spectra	13926

3. Experiment Control

Figure 4. Precursor mass error of peptide-spectrum matches (PSM) in filtered result. **(a)** Distribution of precursor mass error in ppm; **(b)** Scatterplot of precursor m/z versus precursor mass error in ppm.

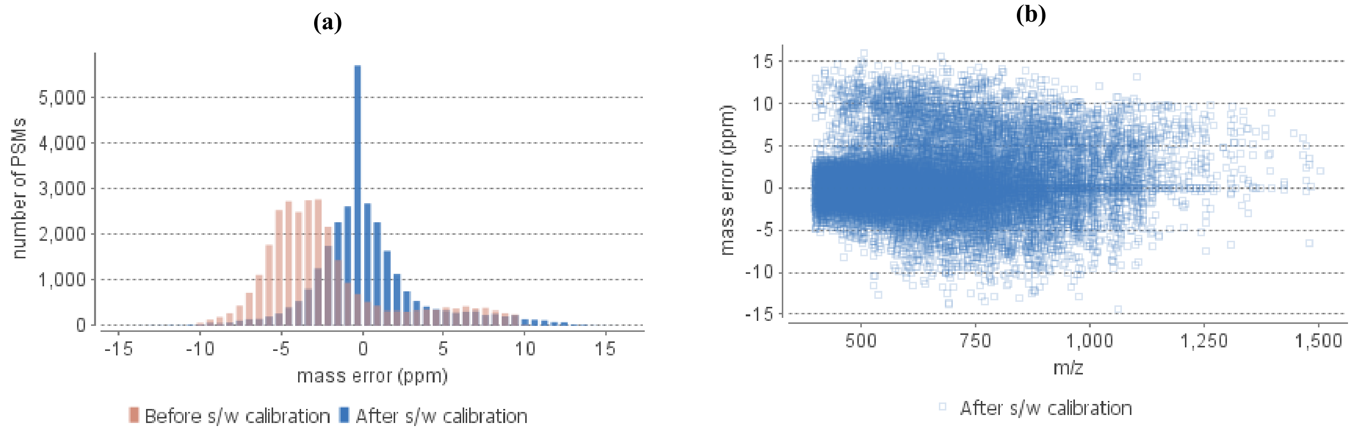

Table 5. Number of identified peptides in each sample by the number of missed cleavages

Missed Cleavages	0	1	2	3	4+
AZ057_01	6499	1064	99	0	0
AZ057_03	5739	872	64	0	0

4. Other Information

Table 6. Search parameters.

Search Engine Name: PEAKS
Parent Mass Error Tolerance: 10.0 ppm
Fragment Mass Error Tolerance: 0.05 Da
Precursor Mass Search Type: monoisotopic
Enzyme: Trypsin
Max Missed Cleavages: 2
Non-specific Cleavage: one
Fixed Modifications:
  Carbamidomethylation: 57.02
Variable Modifications:

Table 7. Instrument parameters.

Fractions: AZ057_01.raw, AZ057_02.raw, AZ057_03.raw
Ion Source: ESI(nano-spray)
Fragmentation Mode: high energy CID (y and b ions)
MS Scan Mode: FT-ICR/Orbitrap
MS/MS Scan Mode: FT-ICR/Orbitrap

Deamidation (NQ): 0.98
Acetylation (N-term): 42.01
Oxidation (M): 15.99
Max Variable PTM Per Peptide: 3
Database: PF_all
Taxon: All
Searched Entry: 1941073
FDR Estimation: Enabled
Merge Options: no merge
Precursor Options: corrected
Charge Options: no correction
Filter Options: no filter
Process: true

1. Notes

2. Result Statistics

Figure 1. False discovery rate (FDR) curve. X axis is the number of peptide-spectrum matches (PSM) being kept. Y axis is the corresponding FDR.

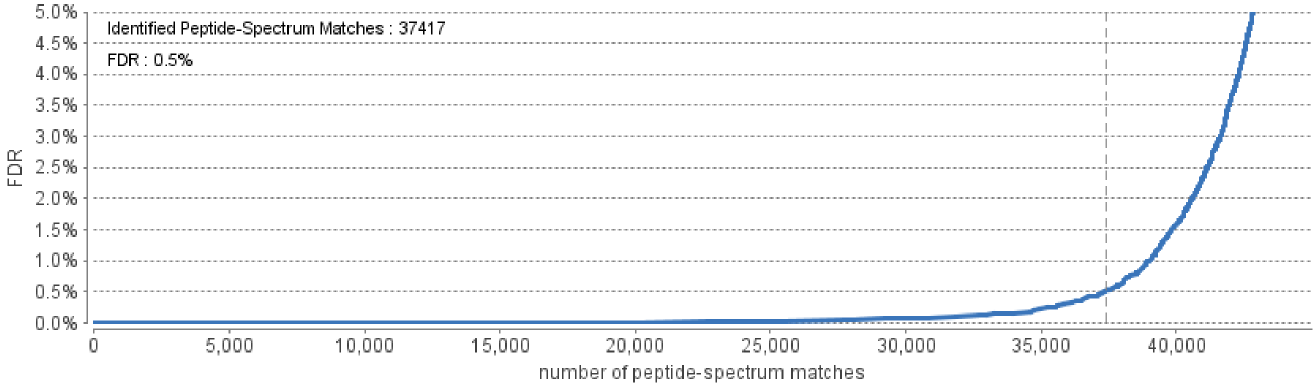

Figure 2. PSM score distribution. **(a)** Distribution of PEAKS peptide score; **(b)** Scatterplot of PEAKS peptide score versus precursor mass error.

(a) **(b)**

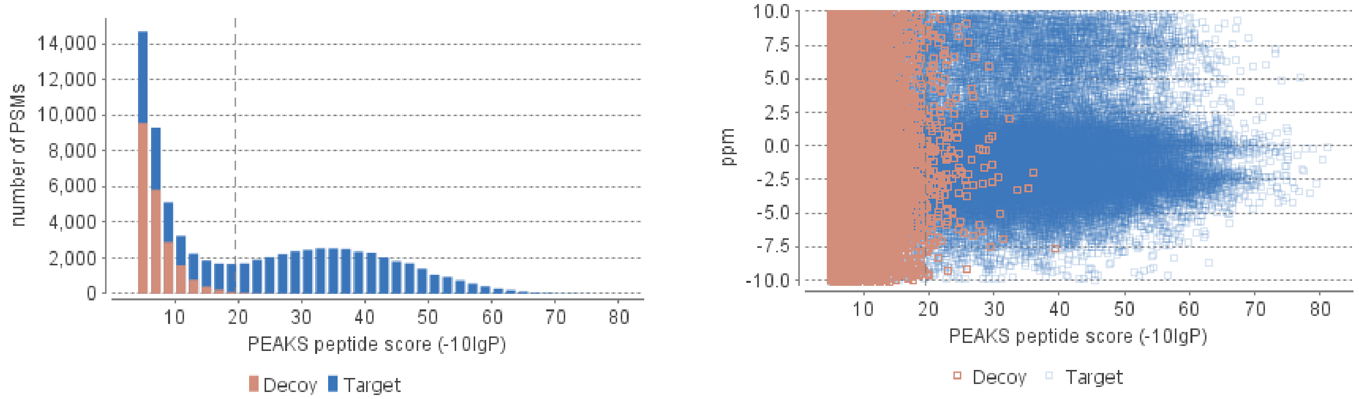

Figure 3. De novo result validation. Distribution of residue local confidence: **(a)** Residues in de novo sequences validated by confident database peptide assignment; **(b)** Residues in "de novo only" sequences.

(a) **(b)**

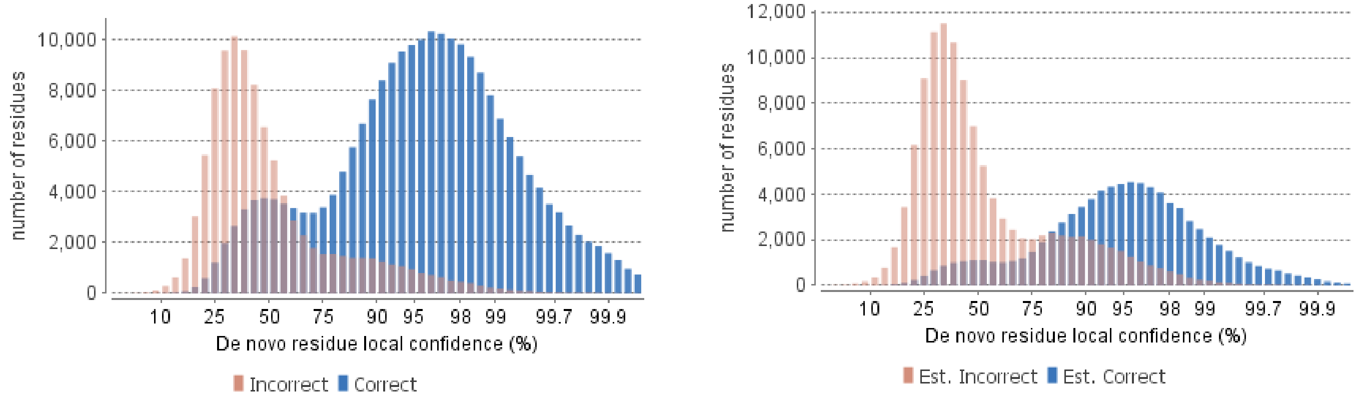

Table 1. Statistics of data.

Table 4. PTM profile.

# of MS scans	25095
# of MS/MS scans	138042

Table 2. Result filtration parameters.

Peptide -10lgP	≥19.5
Peptide Ascore	≥0
Protein -10lgP	≥20
Proteins unique peptides	≥0
De novo ALC Score	≥50%

Table 3. Statistics of filtered result.

Peptide-Spectrum Matches	37417
Peptide sequences	14145
Protein groups	1863
Proteins	3126
Proteins (#Unique Peptides)	1482 (>2); 389 (=2); 975 (=1);
FDR (Peptide-Spectrum Matches)	0.5%
FDR (Peptide Sequences)	1.0%
De Novo Only Spectra	16204

Name	ΔMass	Position	#PSM	-10lgP	Area	AScore
Deamidation	.98	NQ	8320	79.38	2.59E7	9.34
Oxidation	15.99	M	3592	76.97		1000.00
Acetylation	42.01	N-term	806	66.40	2.03E6	1000.00
Carbamidomethyl	57.02	C	134	65.56	3.68E6	1000.00

3. Experiment Control

Figure 4. Precursor mass error of peptide-spectrum matches (PSM) in filtered result. **(a)** Distribution of precursor mass error in ppm; **(b)** Scatterplot of precursor m/z versus precursor mass error in ppm.

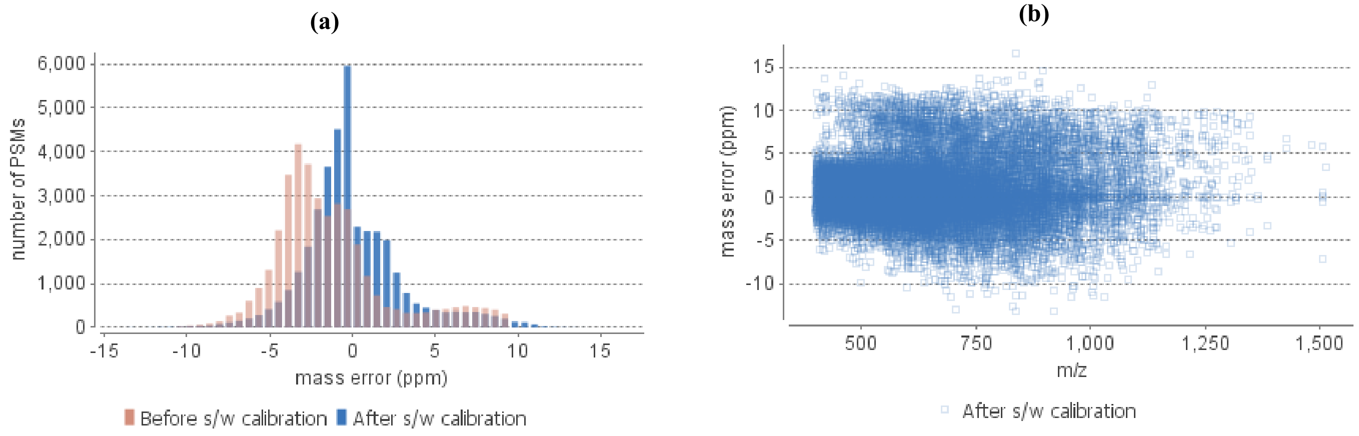

Table 5. Number of identified peptides in each sample by the number of missed cleavages

Missed Cleavages	0	1	2	3	4+
AZ058_01	4808	709	48	0	0
AZ058_02	3727	548	50	0	0
AZ058_03	3682	535	38	0	0

4. Other Information

Table 6. Search parameters.

Search Engine Name: PEAKS
Parent Mass Error Tolerance: 10.0 ppm
Fragment Mass Error Tolerance: 0.05 Da
Precursor Mass Search Type: monoisotopic
Enzyme: Trypsin
Max Missed Cleavages: 2
Non-specific Cleavage: one

Table 7. Instrument parameters.

Fractions: AZ058_01.raw, AZ058_02.raw, AZ058_03.raw
Ion Source: ESI(nano-spray)
Fragmentation Mode: high energy CID (y and b ions)
MS Scan Mode: FT-ICR/Orbitrap
MS/MS Scan Mode: FT-ICR/Orbitrap

Fixed Modifications:
  Carbamidomethylation: 57.02
Variable Modifications:
  Deamidation (NQ): 0.98
  Acetylation (N-term): 42.01
  Oxidation (M): 15.99
Max Variable PTM Per Peptide: 3
Database: PF_all
Taxon: All
Searched Entry: 1941073
FDR Estimation: Enabled
Merge Options: no merge
Precursor Options: corrected
Charge Options: no correction
Filter Options: no filter
Process: true

1. Notes

2. Result Statistics

Figure 1. False discovery rate (FDR) curve. X axis is the number of peptide-spectrum matches (PSM) being kept. Y axis is the corresponding FDR.

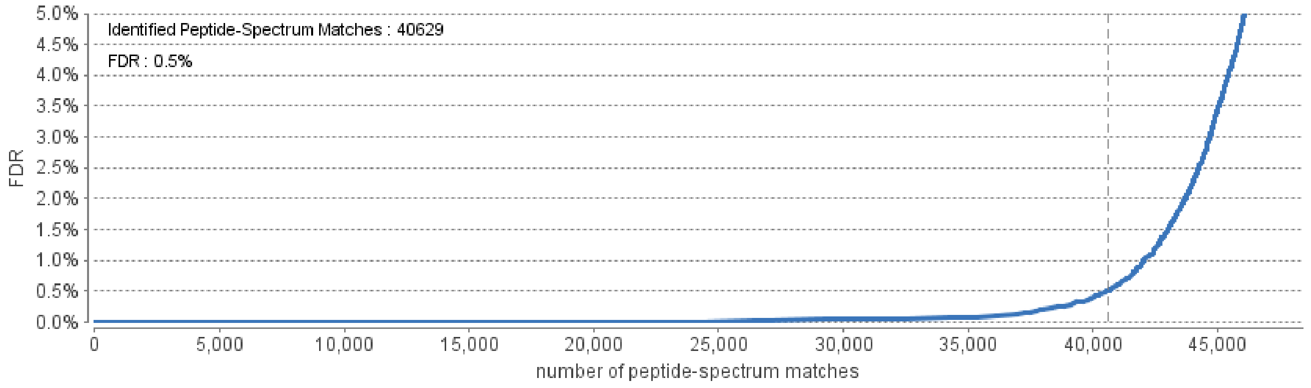

Figure 2. PSM score distribution. **(a)** Distribution of PEAKS peptide score; **(b)** Scatterplot of PEAKS peptide score versus precursor mass error.

(a)

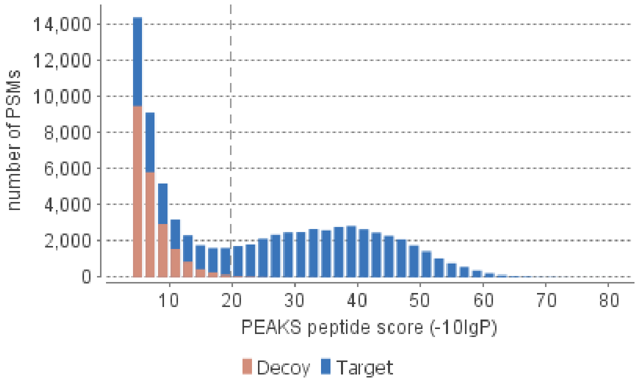

(b)

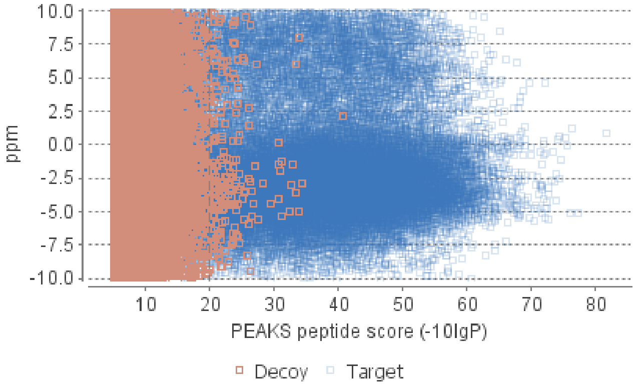

Figure 3. De novo result validation. Distribution of residue local confidence: **(a)** Residues in de novo sequences validated by confident database peptide assignment; **(b)** Residues in "de novo only" sequences.

(a)

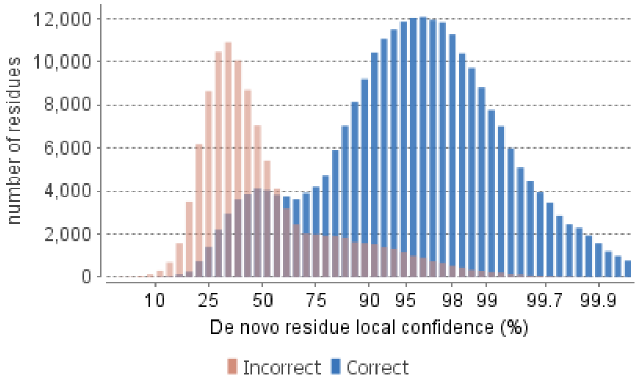

(b)

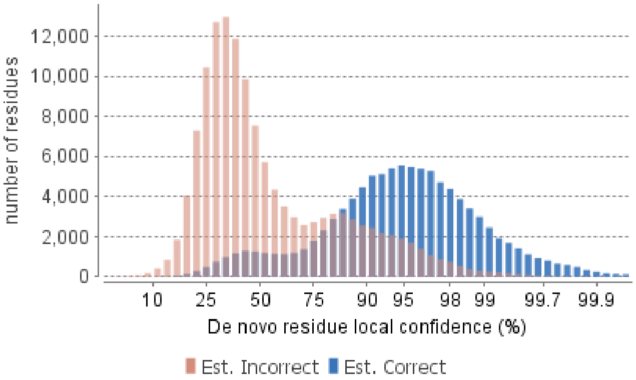

Table 1. Statistics of data.

# of MS scans	24993

Table 4. PTM profile.

Name	ΔMass	Position	#PSM	-10lgP	Area	AScor

# of MS/MS scans 138155

Table 2. Result filtration parameters.

Peptide -10lgP	≥19.9
Peptide Ascore	≥0
Protein -10lgP	≥20
Proteins unique peptides	≥0
De novo ALC Score	≥50%

Table 3. Statistics of filtered result.

Peptide-Spectrum Matches	40629
Peptide sequences	15407
Protein groups	1918
Proteins	3485
Proteins (#Unique Peptides)	1553 (>2); 456 (=2); 1248 (=1);
FDR (Peptide-Spectrum Matches)	0.5%
FDR (Peptide Sequences)	1.0%
De Novo Only Spectra	19175

Deamidation	.98	NQ	9784	81.63		32.9[illegible]
Oxidation	15.99	M	4653	75.47		1000.0[illegible]
Acetylation	42.01	N-term	942	60.40		1000.0[illegible]
Carbamidomethyl	57.02	C	143	67.38	5.96E6	1000.0[illegible]

3. Experiment Control

Figure 4. Precursor mass error of peptide-spectrum matches (PSM) in filtered result. **(a)** Distribution of precursor mass error in ppm; **(b)** Scatterplot of precursor m/z versus precursor mass error in ppm.

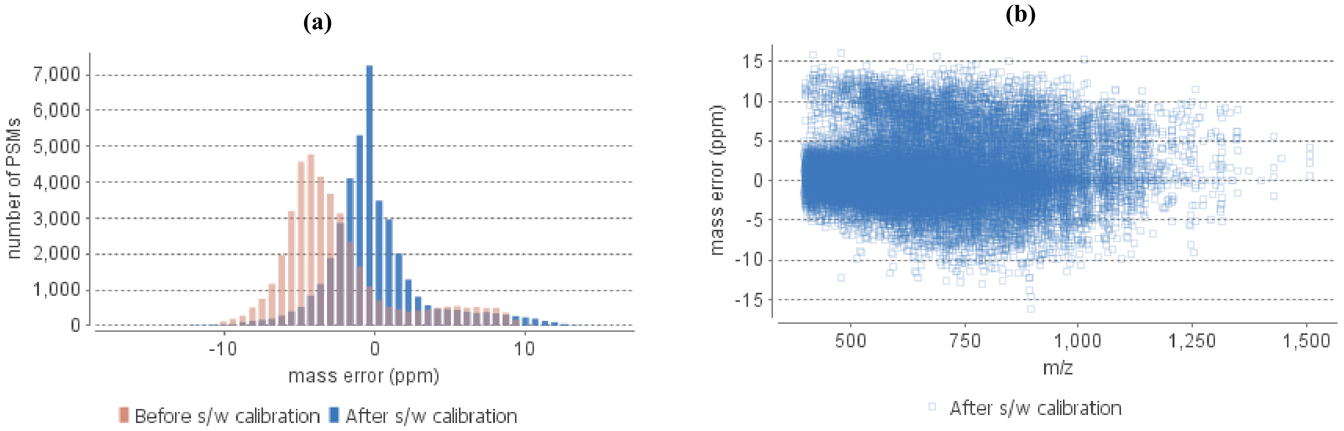

Table 5. Number of identified peptides in each sample by the number of missed cleavages

Missed Cleavages	0	1	2	3	4+
AZ059_01	4337	694	58	0	0
AZ059_02	4526	780	55	0	0
AZ059_03	4251	657	49	0	0

4. Other Information

Table 6. Search parameters.

Search Engine Name: PEAKS
Parent Mass Error Tolerance: 10.0 ppm
Fragment Mass Error Tolerance: 0.05 Da
Precursor Mass Search Type: monoisotopic
Enzyme: Trypsin
Max Missed Cleavages: 2
Non-specific Cleavage: one
Fixed Modifications:

Table 7. Instrument parameters.

Fractions: AZ059_01.raw, AZ059_02.raw, AZ059_03.raw
Ion Source: ESI(nano-spray)
Fragmentation Mode: high energy CID (y and b ions)
MS Scan Mode: FT-ICR/Orbitrap
MS/MS Scan Mode: FT-ICR/Orbitrap

Carbamidomethylation: 57.02
Variable Modifications:
Deamidation (NQ): 0.98
Acetylation (N-term): 42.01
Oxidation (M): 15.99
Max Variable PTM Per Peptide: 3
Database: PF_all
Taxon: All
Searched Entry: 1941073
FDR Estimation: Enabled
Merge Options: no merge
Precursor Options: corrected
Charge Options: no correction
Filter Options: no filter
Process: true

1. Notes

2. Result Statistics

Figure 1. False discovery rate (FDR) curve. X axis is the number of peptide-spectrum matches (PSM) being kept. Y axis is the corresponding FDR.

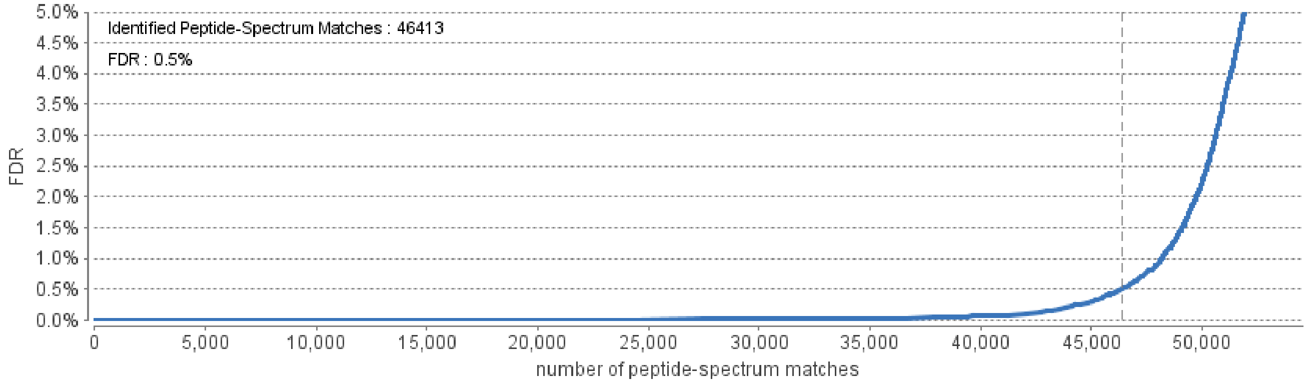

Figure 2. PSM score distribution. **(a)** Distribution of PEAKS peptide score; **(b)** Scatterplot of PEAKS peptide score versus precursor mass error.

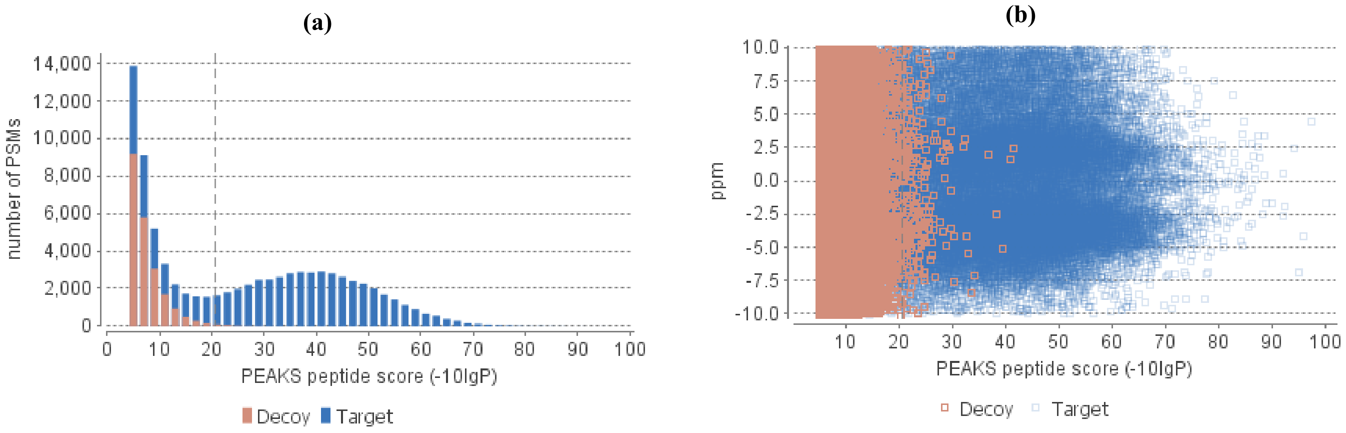

Figure 3. De novo result validation. Distribution of residue local confidence: **(a)** Residues in de novo sequences validated by confident database peptide assignment; **(b)** Residues in "de novo only" sequences.

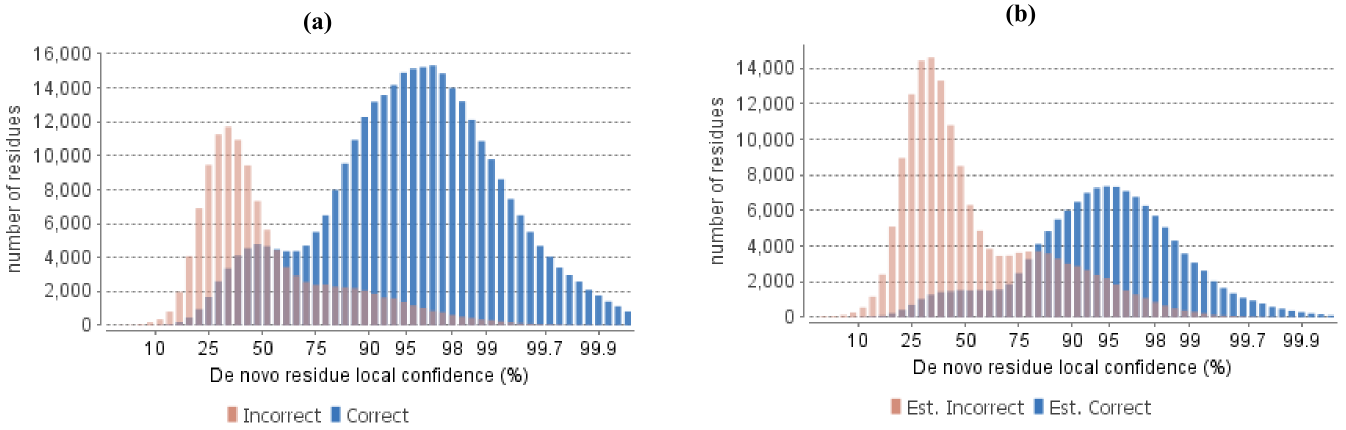

Table 1. Statistics of data.

# of MS scans	23880

Table 4. PTM profile.

Name	ΔMass	Position	#PSM	-10lgP	Area	AScor

# of MS/MS scans 141814

Deamidation	.98	NQ	11059	97.39		47.0
Oxidation	15.99	M	4967	94.14		1000.0
Acetylation	42.01	N-term	1172	75.74	6.76E6	1000.0
Carbamidomethyl	57.02	C	196	60.17	5.77E7	1000.0

Table 2. Result filtration parameters.

Peptide -10lgP	≥20.6
Peptide Ascore	≥0
Protein -10lgP	≥20
Proteins unique peptides	≥0
De novo ALC Score	≥50%

Table 3. Statistics of filtered result.

Peptide-Spectrum Matches	46413
Peptide sequences	17879
Protein groups	2116
Proteins	3481
Proteins (#Unique Peptides)	1799 (>2); 381 (=2); 1033 (=1);
FDR (Peptide-Spectrum Matches)	0.5%
FDR (Peptide Sequences)	0.9%
De Novo Only Spectra	22522

3. Experiment Control

Figure 4. Precursor mass error of peptide-spectrum matches (PSM) in filtered result. **(a)** Distribution of precursor mass error in ppm; **(b)** Scatterplot of precursor m/z versus precursor mass error in ppm.

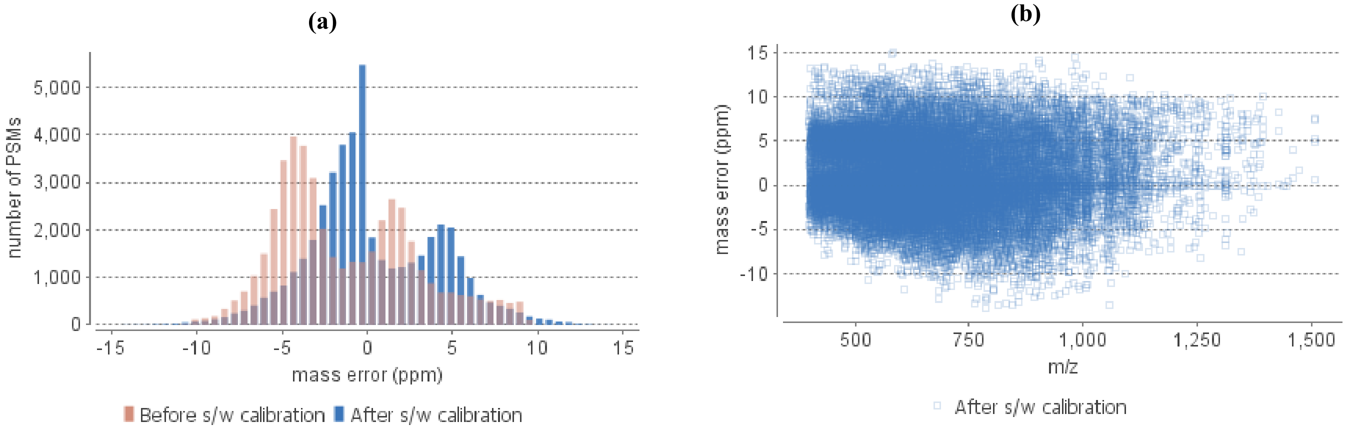

Table 5. Number of identified peptides in each sample by the number of missed cleavages

Missed Cleavages	0	1	2	3	4+
AZ061_01	4631	691	58	0	0
AZ061_02	4722	823	59	0	0
AZ061_03	5857	948	90	0	0

4. Other Information

Table 6. Search parameters.

Search Engine Name: PEAKS
Parent Mass Error Tolerance: 10.0 ppm
Fragment Mass Error Tolerance: 0.05 Da
Precursor Mass Search Type: monoisotopic
Enzyme: Trypsin
Max Missed Cleavages: 2
Non-specific Cleavage: one
Fixed Modifications:

Table 7. Instrument parameters.

Fractions: AZ061_01.raw, AZ061_02.raw, AZ061_03.raw
Ion Source: ESI(nano-spray)
Fragmentation Mode: high energy CID (y and b ions)
MS Scan Mode: FT-ICR/Orbitrap
MS/MS Scan Mode: FT-ICR/Orbitrap

Carbamidomethylation: 57.02
Variable Modifications:
Deamidation (NQ): 0.98
Acetylation (N-term): 42.01
Oxidation (M): 15.99
Max Variable PTM Per Peptide: 3
Database: PF_all
Taxon: All
Searched Entry: 1941073
FDR Estimation: Enabled
Merge Options: no merge
Precursor Options: corrected
Charge Options: no correction
Filter Options: no filter
Process: true

1. Notes

2. Result Statistics

Figure 1. False discovery rate (FDR) curve. X axis is the number of peptide-spectrum matches (PSM) being kept. Y axis is the corresponding FDR.

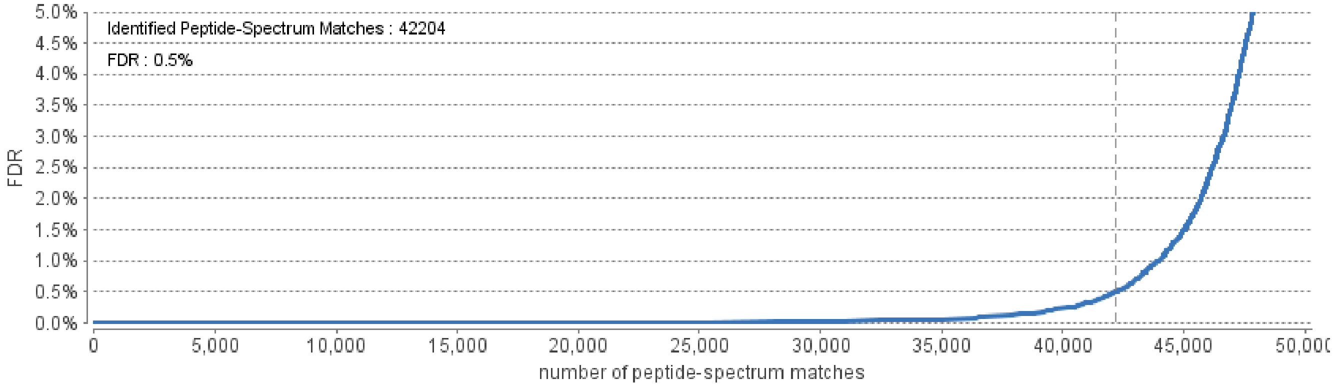

Figure 2. PSM score distribution. **(a)** Distribution of PEAKS peptide score; **(b)** Scatterplot of PEAKS peptide score versus precursor mass error.

(a)

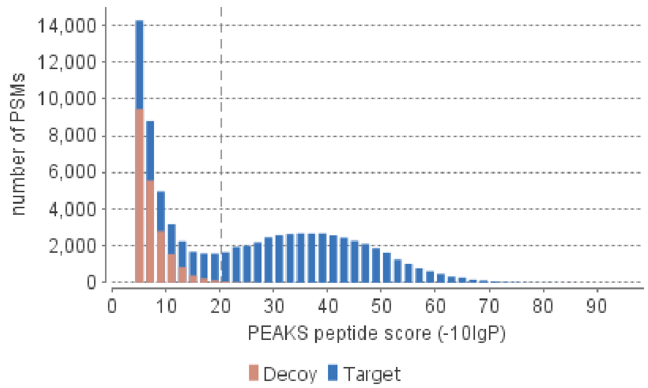

(b)

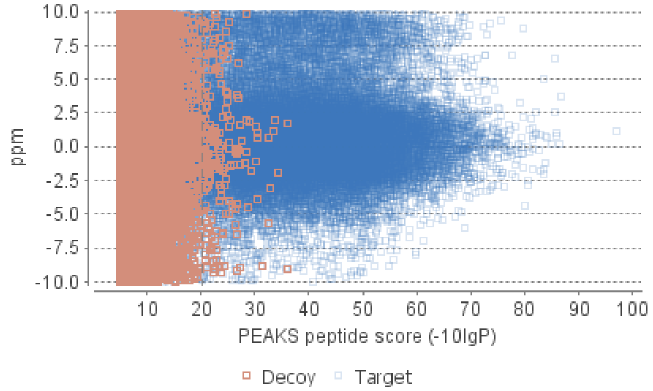

Figure 3. De novo result validation. Distribution of residue local confidence: **(a)** Residues in de novo sequences validated by confident database peptide assignment; **(b)** Residues in "de novo only" sequences.

(a)

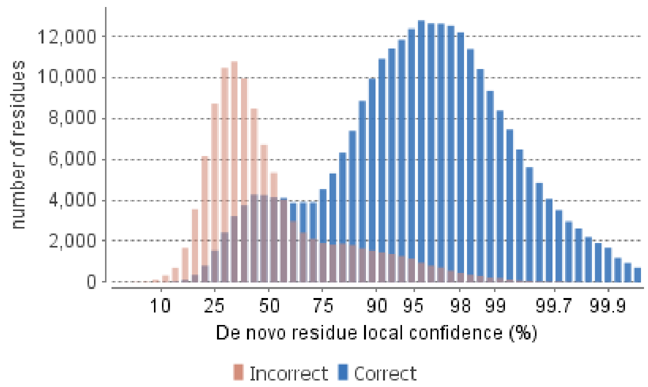

(b)

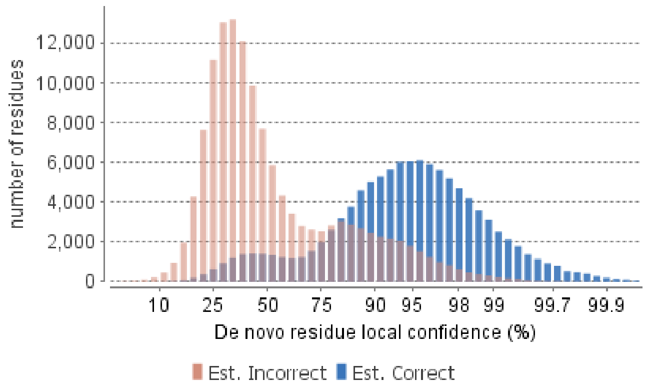

Table 1. Statistics of data.

Table 4. PTM profile.

# of MS scans	24761
# of MS/MS scans	138843

Table 2. Result filtration parameters.

Peptide -10lgP	≥20.2
Peptide Ascore	≥0
Protein -10lgP	≥20
Proteins unique peptides	≥0
De novo ALC Score	≥50%

Table 3. Statistics of filtered result.

Peptide-Spectrum Matches	42204
Peptide sequences	16601
Protein groups	2004
Proteins	3311
Proteins (#Unique Peptides)	1733 (>2); 356 (=2); 947 (=1);
FDR (Peptide-Spectrum Matches)	0.5%
FDR (Peptide Sequences)	1.0%
De Novo Only Spectra	19680

Name	ΔMass	Position	#PSM	-10lgP	Area	AScore
Deamidation	.98	NQ	8448	86.76		28.36
Oxidation	15.99	M	3557	82.00	8.8E7	1000.00
Acetylation	42.01	N-term	1063	73.46	4.03E6	1000.00
Carbamidomethyl	57.02	C	103	60.50	7.43E6	1000.00

3. Experiment Control

Figure 4. Precursor mass error of peptide-spectrum matches (PSM) in filtered result. **(a)** Distribution of precursor mass error in ppm; **(b)** Scatterplot of precursor m/z versus precursor mass error in ppm.

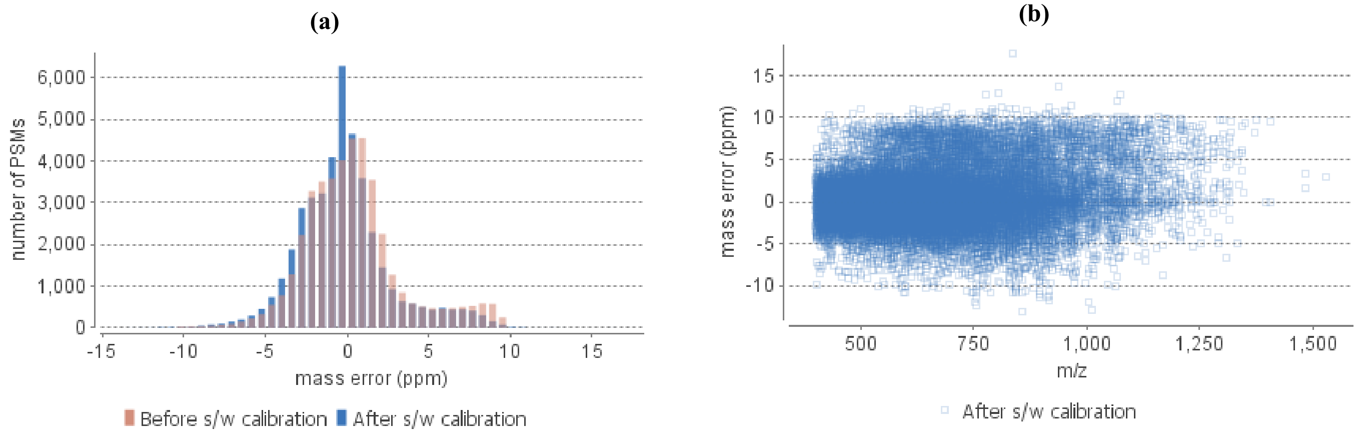

Table 5. Number of identified peptides in each sample by the number of missed cleavages

Missed Cleavages	0	1	2	3	4+
AZ062_01	5265	842	71	0	0
AZ062_02	4000	635	55	0	0
AZ062_03	4872	793	68	0	0

4. Other Information

Table 6. Search parameters.

Search Engine Name: PEAKS
Parent Mass Error Tolerance: 10.0 ppm
Fragment Mass Error Tolerance: 0.05 Da
Precursor Mass Search Type: monoisotopic
Enzyme: Trypsin
Max Missed Cleavages: 2
Non-specific Cleavage: one

Table 7. Instrument parameters.

Fractions: AZ062_01.raw, AZ062_02.raw, AZ062_03.raw
Ion Source: ESI(nano-spray)
Fragmentation Mode: high energy CID (y and b ions)
MS Scan Mode: FT-ICR/Orbitrap
MS/MS Scan Mode: FT-ICR/Orbitrap

Fixed Modifications:
  Carbamidomethylation: 57.02
Variable Modifications:
  Deamidation (NQ): 0.98
  Acetylation (N-term): 42.01
  Oxidation (M): 15.99
Max Variable PTM Per Peptide: 3
Database: PF_all
Taxon: All
Searched Entry: 1941073
FDR Estimation: Enabled
Merge Options: no merge
Precursor Options: corrected
Charge Options: no correction
Filter Options: no filter
Process: true
