# Supplementary material for: Mutant Proteomics of Lung Adenocarcinomas Harboring Different EGFR Mutations
Source: Front Oncol. 2020 Aug 25;10:1494. doi: 10.3389/fonc.2020.01494 (PMC7477350; doi:10.3389/fonc.2020.01494)
Supplement: Figure S1 — Geneontology (GO) analysis of the identified proteins to the three traits-M1 (L858R), M2 (Ex19del), and NM (no L858R/Ex19del). (A) GO Biological process. 1, developmental process (GO:0032502); 2, multicellular organismal process (GO:0032501); 3, cellular process (GO:0009987); 4, reproduction (GO:0000003); 5, cell population proliferation (GO:0008283); 6, localization (GO:0051179); 7, reproductive process (GO:0022414); 8, multiorganism process (GO:0051704); 9, biological adhesion (GO:0022610); 10, immune system process (GO:0002376); 11, cellular component organization or biogenesis (GO:0071840); 12, biological regulation (GO:0065007); 13, growth (GO:0040007); 14, signaling (GO:0023052); 15, metabolic process (GO:0008152); 16, response to stimulus (GO:0050896); 17, pigmentation (GO:0043473); 18, behavior (GO:0007610); 19, locomotion (GO:0040011). (B) GO Molecular function. 1, translation regulator activity (GO:0045182); 2, transcription regulator activity (GO:0140110); 3, molecular transducer activity (GO:0060089); 4, binding (GO:0005488); 5, structural molecule activity (GO:0005198); 6, molecular function regulator (GO:0098772); 7, catalytic activity (GO:0003824); 8, transporter activity (GO:0005215). (C) GO Cellular component. 1, synapse part (GO:0044456); 2, membrane part (GO:0044425); 3, membrane (GO:0016020); 4, synapse (GO:0045202); 5, organelle part (GO:0044422); 6, extracellular region part (GO:0044421); 7, cell junction (GO:0030054); 8, membrane-enclosed lumen (GO:0031974); 9, protein-containing complex (GO:0032991); 10, supramolecular complex (GO:0099080); 11, extracellular region (GO:0005576); 12, cell (GO:0005623); 13, cell part (GO:0044464); 14, organelle (GO:0043226). (D) GO Protein class. 1, extracellular matrix protein (PC00102); 2, cytoskeletal protein (PC00085); 3, transporter (PC00227); 4, scaffold/adaptor protein (PC00226); 5, cell adhesion molecule (PC00069); 6, nucleic acid binding protein (PC00171); 7, intercellular signal molecule (PC00207); 8, [file Data_Sheet_3.pdf]

Supplementary Information File 3

Mutant Proteomics of Lung Adenocarcinomas Harboring Different EGFR Mutations

Toshihide Nishimura, Ákos Végvári, Haruhiko Nakamura, Harubumi Kato, Hisashi Saji

Figure S1

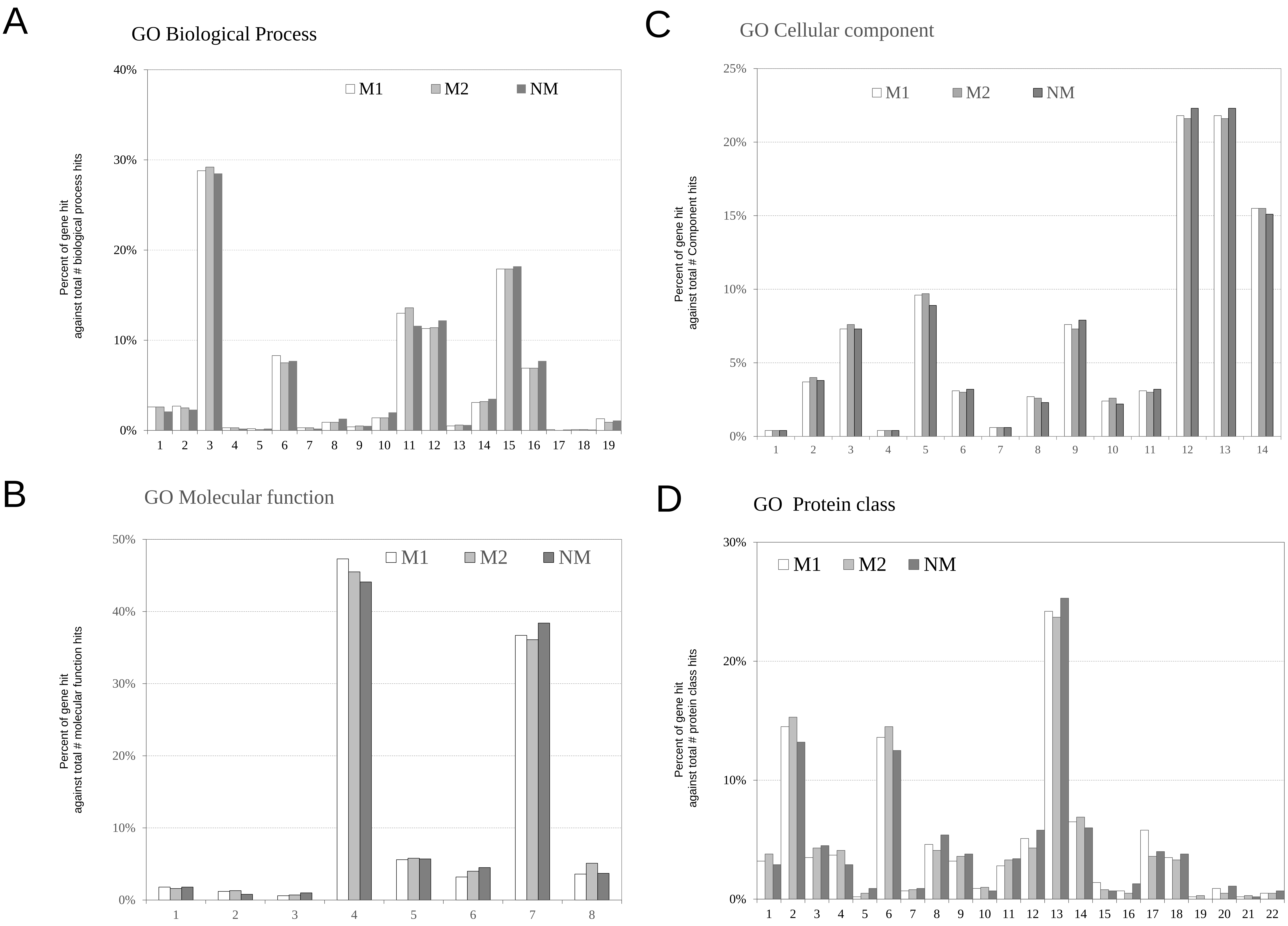
A
GO Biological Process
M1
M2
NM
Percent of gene hit against total # biological process hits
0%
10%
20%
30%
40%
1 2 3 4 5 6 7 8 9 10 11 12 13 14 15 16 17 18 19
B
GO Molecular function
M1
M2
NM
Percent of gene hit against total # molecular function hits
0%
10%
20%
30%
40%
50%
1 2 3 4 5 6 7 8
C
GO Cellular component
M1
M2
NM
Percent of gene hit against total # Component hits
0%
5%
10%
15%
20%
25%
1 2 3 4 5 6 7 8 9 10 11 12 13 14
D
GO Protein class
M1
M2
NM
Percent of gene hit against total # protein class hits
0%
10%
20%
30%
1 2 3 4 5 6 7 8 9 10 11 12 13 14 15 16 17 18 19 20 21 22

Figure S2.

Relationship between module eigen proteins and the L858R and Ex19del mutations in the *EGFR* gene. Each row in the embedded table represents weighted gene co-expression network analysis results for each module. The first and second columns in the table represent module ID and colour name of the module. The third column represents the number of proteins in each module. The fourth, fifth and sixth (seventh, eighth and nineth) columns indicate the correlation coefficients (*p*-values of the correlation coefficients) between the corresponding modules and the clinical traits. The table is colour-coded by correlation coefficient according to the colour legend on the right side of the figure. The intensity and direction of the correlations are indicated on the right side of the heatmap (red, positive correlation; blue, negative correlation). *p*-values (< 0.10) are highlighted in red.

Module ID	Module colour	# of mutant proeins in the module	Module trait relationship					
			trait M1 (correlation)	trait M2 (correlation)	trait NM (correlation)	trait M1 (*p*-value)	trait M2 (*p*-value)	trait NM (*p*-value)
WM1	darkgreen	32	0.1065	-0.1390	0.0281	0.5363	0.4187	0.8706
WM2	yellow	63	0.1971	0.1282	-0.2817	0.2492	0.4561	0.0960
WM3	black	55	0.1631	0.0680	-0.2001	0.3418	0.6937	0.2419
WM4	red	58	-0.0976	-0.0976	0.1690	0.5712	0.5712	0.3244
WM5	cyan	38	-0.1372	0.1920	-0.0475	0.4251	0.2619	0.7832
WM6	blue	77	-0.1372	0.4115	-0.2376	0.4251	0.0127	0.1630
WM7	pink	54	0.0510	-0.1335	0.0714	0.7675	0.4378	0.6791
WM8	green	63	-0.1636	0.1443	0.0167	0.3405	0.4010	0.9232
WM9	tan	41	-0.0976	-0.0976	0.1690	0.5712	0.5712	0.3244
WM10	turquoise	81	0.2928	-0.0976	-0.1690	0.0831	0.5712	0.3244
WM11	salmon	39	-0.0976	-0.0976	0.1690	0.5712	0.5712	0.3244
WM12	royalblue	34	0.2928	-0.0976	-0.1690	0.0831	0.5712	0.3244
WM13	purple	46	-0.0976	-0.0976	0.1690	0.5712	0.5712	0.3244
WM14	midnightblue	37	0.2928	-0.0976	-0.1690	0.0831	0.5712	0.3244
WM15	magenta	48	-0.0976	-0.0976	0.1690	0.5712	0.5712	0.3244
WM16	lightyellow	35	-0.0976	-0.0976	0.1690	0.5712	0.5712	0.3244
WM17	lightgreen	36	-0.0976	0.2928	-0.1690	0.5712	0.0831	0.3244
WM18	lightcyan	37	-0.0976	-0.0976	0.1690	0.5712	0.5712	0.3244
WM19	grey60	37	-0.0976	-0.0976	0.1690	0.5712	0.5712	0.3244
WM20	greenyellow	44	-0.0976	-0.0976	0.1690	0.5712	0.5712	0.3244
WM21	darkturquoise	31	-0.0976	-0.0976	0.1690	0.5712	0.5712	0.3244
WM22	brown	74	0.2928	-0.0976	-0.1690	0.0831	0.5712	0.3244
WM23	darkred	32	-0.0976	-0.0976	0.1690	0.5712	0.5712	0.3244

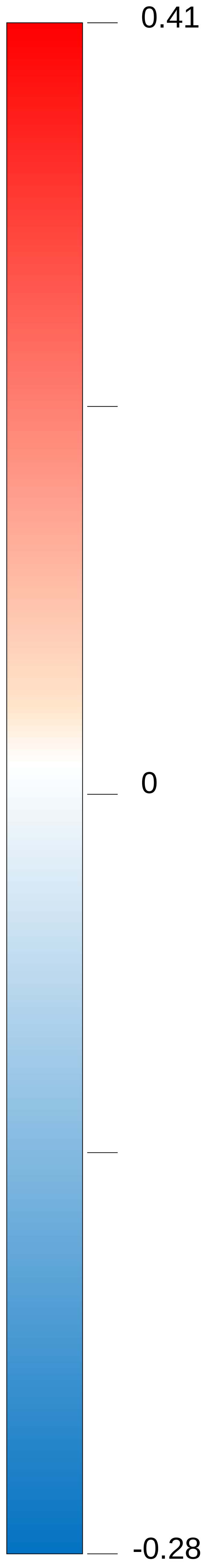

Figure S3 R_{SC} values between M1 and M2 calculated for proteins identified (X-axis). Mutant proteins upregurlated with twice fold changes for M1 ($R_{SC} \geq 1$) and M2 ($R_{SC} \leq -1$) are denoted.

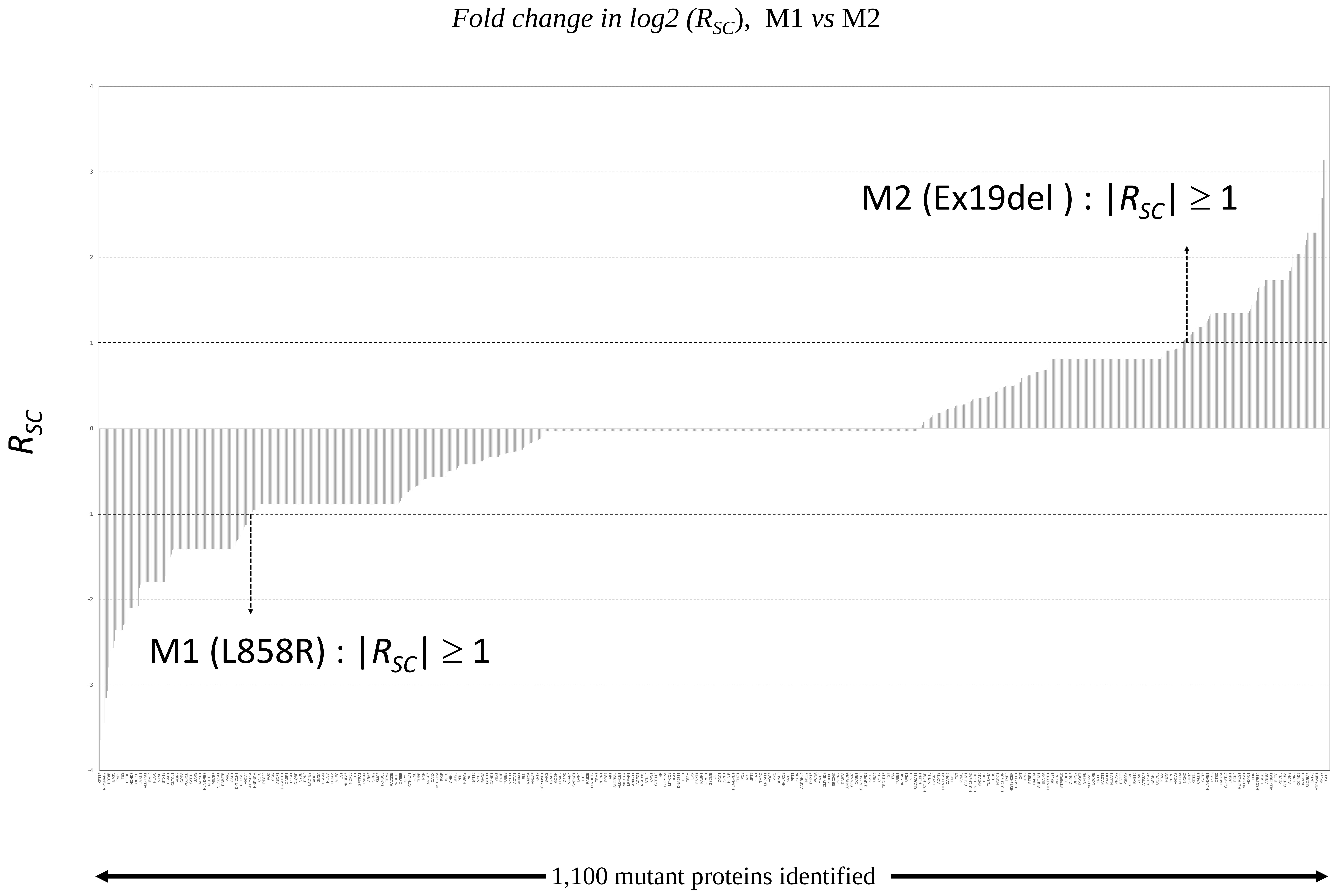

Table S1. The comparative analysis results of causal networks predicted by IPA for mutant proteins expressed commonly (see Venn map in Fig. 2A). *MNK1/2*, *Max-Myc*, *MYC*, *XBP1*, *BTG2*, *F8*, *STK11*, and *RAD21* were highly activated (z-score > 2.5) and differentially under M1 (L858R).

**Causal Networks**	**Depth**	**Activation *z*-score**		
		**M1 (L858R)**	**M2 (Ex19del)**	**NM (no L858R/Ex19del)**
MNK1/2	2	3.727	1.091	0.853
Max-Myc	3	3.077	0.302	0.832
MYC	2	2.73	-0.164	-0.438
XBP1	1	2.646	1.342	0
BTG2	2	2.53	0.816	1.134
F8	2	2.53	0.447	1.342
STK11	1	2.53	0.447	1.414
RAD21	2	2.53	0.655	0.626
bardoxolone methyl	2	2.38	1.257	1
CAB39	2	2.309	0	1
BIRC5	2	2.216	1.061	-0.149
PCGEM1	3	2.151	-0.493	-0.729
tamoxifen	2	2.138	1.029	1.372
PCGEM1	2	2.03	0.447	0
MXD1	2	-2.111	0	0.426
bivalirudin	2	-2.138	-1.414	-1.414
argatroban	2	-2.138	-1.414	-1.414
PFDN5	3	-2.157	0	-0.802
FBXO32	2	-2.188	-0.784	-1.177
macitentan	2	-2.236	-1.342	-1.342
N-Ac-leucyl-leucyl-methioninal	2	-2.236	-0.816	-0.447
ciprofloxacin	1	-2.236	-1	-1
FBXL14	3	-2.278	-0.143	1.336
IgG	1	-2.333	-0.447	-1.342
PUF60	2	-2.343	-0.447	-0.218
LONP1	1	-2.449	-1.414	-1.414
FBXL14	2	-2.534	-0.816	-0.2
PUF60	3	-2.593	0.164	0.438
dihydromorphine	3	-2.654	-0.392	-1.225
SGI 1776	2	-2.777	-1.091	-0.626
PPP2R2B	3	-3.444	-0.762	-0.283
